# Impact of Metacognition on Health-Related Behavior: A Mediation Model Study

**DOI:** 10.1155/2023/6547804

**Published:** 2023-01-31

**Authors:** Yue Li, Jiaxing Tang, Xiaoran Ma, Xiaomin Zhang, Yansong Xue, Xiang Zhao

**Affiliations:** ^1^School of Physical Education, Huaibei Normal University, Huaibei, China; ^2^School of Physical Education, Shanghai University of Sport, Shanghai, China; ^3^School of Physical Education, Xi'an Physical Education University, Xi'an, China; ^4^School of Electronic and Information Engineering, Huaibei Institute of Technology, Huaibei, China; ^5^Qihe No. 1 Middle School, Dezhou, China

## Abstract

**Objective:**

The study aims to explore the correlation mechanism among metacognition, attitude toward physical exercise, and health-related behavior in high school students.

**Methods:**

A total of 869 students (17 ± 1.70) from Anhui, Zhejiang, Shandong, and Fujian provinces were selected by stratified sampling to complete the Metacognition Questionnaire, Health-Related Behavior Self-Rating Scale, Attitude Toward Physical Exercise Scale, and Depression-Anxiety-Stress Scale (Simplified Chinese version, DASS-21).

**Results:**

(1) Metacognition was negatively predictive of attitude toward physical exercise and health-related behavior (*β* = −0.236, *P* < 0.01; *β* = −0.239, *P* < 0.01) but positively predictive of negative emotion (*β* = 0.496, *P* < 0.01); (2) attitude toward physical exercise was positively predictive of health-related behavior (*β* = 0.533, *P* < 0.01) but negatively predictive of negative emotion (*β* = −0.336, *P* < 0.01); and (3) negative emotion was negatively predictive of health-related behavior (*β* = −0.389, *P* < 0.01).

**Conclusions:**

Metacognition not only has a directly predictive effect on health-related behavior but also predicts it through attitude toward physical exercise. Negative emotion also mediates the relationship between metacognition and attitude toward physical exercise.

## 1. Introduction

Health-related behavior refers to the illness-prevention activities undertaken by individuals to protect, promote, and maintain health [1]. Health-related behavior is not only a significant indicator of health level but also a determinant factor in the social development of human beings. In May 2022, the General Office of the State Council of the People's Republic of China issued “The 14th Five-Year National Health Plan,” which called for health promotion, health education, and healthy lifestyle cultivation among Chinese people and for comprehensive interventions on Health-related issues and influential factors [2]. In adolescence, individuals undergo tremendous physical and psychological changes. The imbalance of brain structure and function development also means that adolescents are more likely to be involved in behaviors that put their health at risk [3]. The incidence rate of health-risk behaviors among Chinese adolescents is as high as 30% [4]. Adolescence is a critical period in the development of health-related behavior. Health-related behavior patterns and habits developed in adolescence will persist into adulthood [5]. They are highly correlated with health levels in adulthood [6]. Therefore, it is highly significant to prevent and change health-risk behaviors and promote health-related behavior patterns and habits among adolescents. This study aims to explore the influential factors and mechanisms of health-related behavior among high school students, thereby providing a theoretical and empirical basis for promoting health-related behaviors.

## 2. Theoretical Background

### 2.1. Metacognition and Health-Related Behavior

Adolescents' health-related behavior is affected by many factors, including socioeconomic status, personality, social support, and cognition [7]. Among these elements, cognitive factors have received the most attention. Cognition is the process of obtaining and applying information, including perception, memory, thinking, imagination, and language. Cognition is a complex process with multidimensional, associative, and holistic characteristics [8]. Individuals obtain information through cognition, convert it into internal psychological activities, and then control their own behavior. Cognitive behavior modification theory holds that a person's behavior depends on their cognitive assessment of the world around them. Most of the problematic behaviors of adolescent students stem from a lack of correct cognition. The problematic behaviors of adolescent students are directly related to their cognitive errors. Therefore, it is necessary to change the cognitive process and quality of adolescent students to promote their cognitive development and address their health-damaging behaviors [9]. Metacognition is an individual's self-awareness and self-regulation of cognitive activities. It is the monitoring system of human thinking. Metacognition is the ability of individuals to plan, monitor, regulate, and evaluate themselves during cognitive activities [10]. The development of metacognitive ability is conducive to improving the self-cognition ability of adolescent students [11]. In the process of self-cognition, the lower level of self-cognition produces cognitive bias toward society or other people. Mental health problems are also addressed through self-control and self-regulation. A research study indicated that metacognition could affect behavior through individual monitoring and self-regulation of cognitive function, thereby preventing illness and maintaining a healthy state [12]. Metacognition beliefs can predict depression, anxiety, and dysfunctional beliefs related to health [13]. In a study investigating the influence of metacognition on stress perception, depression, anxiety symptoms, and quality of life, metacognition skills were shown to play a role in physical and mental health promotion [14] and in preventing the occurrence of health risk behaviors [15, 16]. The first hypothesis of the current study is that the metacognition of high school students can positively predict health-related behavior.

### 2.2. The Mediating Effect of Attitude toward Physical Exercise

In this study, attitude toward physical exercise mediates between metacognition and health-related behavior. Attitudes have been identified as the “core concept” of social psychology [17]. An attitude is a psychological tendency manifested in the positive or negative evaluation of a specific object. Attitudes involving cognition, emotion, and behavioral tendencies are acquired psychological entities [18]. Research has shown that attitudes strongly interact with cognition and behavior, enabling them to guide attention, interpretation, and memory and promote behavior change [19]. Metacognition has a judgment role in adjusting the influence of attitudes on subsequent cognition and behavior [20]. According to the theory that attitude influences metacognition [21], the subjective judgment or belief inherent in an individual's attitude can moderate the influence of attitude on subsequent cognition and behavior and play an important role in the process of behavior change [22]. Attitude-related metacognition can promote health-related behavior by changing stereotypes [23]. Physical exercise has been viewed as the “medicine” of health maintenance and illness prevention. It is attitude that decides the likelihood of participation in physical exercise. Numerous studies have shown that attitude toward physical exercise is closely correlated with health-related behavior. A recent study on college students indicated that a healthy diet and an attitude toward physical exercise can promote the cultivation of a healthy lifestyle [24]. College students should be fully aware of the potential health threats of poor eating habits and being overweight. They should improve their attitude toward exercise to prevent overweight-related diseases [25]. Obesity and sleep deprivation in adolescents are associated with negative exercise attitudes [26]. Attitude toward physical exercise and motivation are important predictors of physical activity intervention in adolescents [27]. Therefore, the second hypothesis of the current study is that attitude toward physical exercise has a mediating role between metacognition and health-related behavior.

### 2.3. The Regulating Effect of Negative Emotion

Emotion is a psychological phenomenon mediated by the needs and desires of the subject. Emotion has three unique components: physiological arousal, subjective experience, and external expression. Satisfaction of needs and desires will induce positive emotions, and vice versa [28]. A recent study found that metacognitive beliefs and repetitive negative thinking were related to emotional levels, and individuals with high levels of positive emotion were better able to regulate and monitor their behaviors [29]. Another study explored the role of emotion in physical exercise, showing that participants with negative emotions also had negative attitudes toward physical exercise [30]. Positive or negative emotions may affect attitudes toward physical exercise [31]. Emotion is also an important factor affecting the influence of metacognition on attitudes [32]. It is easier to maintain a positive attitude in a happy state than in a sad state, and this leads to positive behavior [21]. Therefore, the third hypothesis of the current study is that negative emotion could regulate the relationship between metacognition and attitude toward physical exercise.

**Figure 1:**
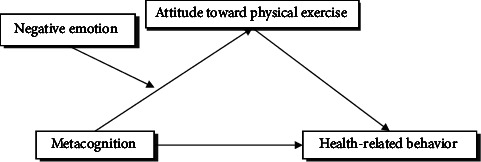
Hypothesized model of the effect of metacognition on health-related behavior.

The current study conducted a deep analysis of the influential factors in health-related behavior. This study not only investigated the direct effect of metacognition on adolescents' health-related behaviors but also investigated the effect of attitude toward physical exercises on adolescents' health-related behaviors and the regulating effect of emotion between metacognition and attitude toward physical exercises based on attitude metacognition theory (Figure 1). This study also elucidates why metacognition affects adolescents' health-related behavior and how to intervene to promote health-relevant behaviors. Our study provides a psychological, theoretical, and empirical basis for preventing and reducing adolescents' health risk behaviors and promoting the development of health-related behavior.

## 3. Materials and Methods

### 3.1. Participants

Stratified sampling was used to select one high school in an urban area and one in a rural area in Shandong, Anhui, Zhejiang, and Fujian provinces in China. A total of eight schools were selected. Then, one class of students from each grade at each school was randomly selected. A total of 1,024 enrolled students in the 24 classes participated in the study. Informed consent was collected from school leaders, teachers, parents, and students. The survey was anonymous and confidential, and students' participation was voluntary. This study was approved by the Ethics Committee of Huaibei Normal University. Questionnaires were sent out and collected by e-mail. The questionnaires were issued in May 2021 to avoid the influence of accommodations and examinations at the beginning and end of an academic year. A total of 926 questionnaires were collected one week later (recovery rate = 90%). After invalid questionnaires (items missing) were eliminated, 869 questionnaires were included for analysis (efficiency rate = 93.8%). There were 396 (45.6%) male students and 473 (54.4%) female students. The average age of the included students is 17 ± 1.7 (Mean ± Standard Deviation, *M* ± SD) years old. Of the students, 278 were in grade 10, 300 in grade 11, and 291 in grade 12. There was no significant difference in the number of students regarding gender and grade.

### 3.2. Research Tools

#### 3.2.1. Metacognition Scale

The revised Chinese version of the Metacognition Questionnaire (McQ-30) was used [33]. Previous research revealed that it was suitable for assessing Chinese students [34, 35]. The questionnaire has 30 items, grouped into six domains: cognitive confidence, positive beliefs, cognitive self-consciousness, uncontrollability, danger, and need to control thoughts. A 4-point evaluation was used, in which 1 represents “Strongly agree” and 4 represents “Strongly Disagree.” The McQ-30 correlates with anxiety symptoms and levels of worry, with higher scores indicating higher levels of anxiety beliefs. The *α* coefficient of internal consistency of the scale was 0.924, and the fitting index of confirmatory factor analysis was as follows: *χ*^2^/d*f* = 3.50, RMSEA = 0.054, NFI = 0.907, RFI = 0.894, and TLI = 0.892, CFI = 0.893.

#### 3.2.2. Health-Related Behavior Scale

The Health Behavior Self-Rating Scale (senior high school version) [36] was used. A previous study showed that the questionnaire was appropriate for a sample of high school students [37]. The questionnaire contains 13 items, grouped into three dimensions: physical exercise, lifestyle, and bad habits avoidance. A 5-point evaluation was used, in which 1 represents “Strongly disagree” and 5 represents “Strongly agree.” Higher scores represent higher levels of physical fitness. The internal consistency coefficient of the scale was 0.889, and the fitting index of confirmatory factor analysis was as follows: *χ*^2^/d*f* = 4.881, RMSEA = 0.040, NFI = 0.920, RFI = 0.898, and TLI = 0.908, CFI = 0.928.

#### 3.2.3. Attitude toward Physical Exercise Scale

The attitude toward physical exercise scale developed by Mao [38] was used, and it showed good applicability in a sample of high school students in a study [39]. The scale contains 70 items and is divided into eight dimensions: behavioral attitude, goal attitude, behavioral cognition, behavioral habits, behavioral intention, emotional experience, behavioral control, and subjective criteria. Among the items, questions 1, 7, 9, 10, 15, 18, 25, 31, 33, 34, 39, 41, 45, 47, 49, 55, 57, 58, 62, 63, 66, 69, and 70 were the reverse scoring questions. A 5-point evaluation was used, in which 1 represents “Strongly match” and 5 represents “Strongly mismatch.” The higher the score (forward treatment to the reverse scoring questions), the better the attitude toward physical exercise. The *α* coefficient of internal consistency of the scale was 0.901, and the fitting index of confirmatory factor analysis was as follows: *χ*^2^/d*f* = 2.660, RMSEA = 0.065, NFI = 0.937, RFI = 0.928, TLI = 0.934, and CFI = 0.942.

#### 3.2.4. Depression-Anxiety-Stress Scale

The Depression-Anxiety-Stress Scale (simplified Chinese version) (DASS-21) [40] which was revised by Gong et al. [41] was used. A previous study [42] showed that the scale was appropriate for secondary school students. The scale contains 21 items and is divided into three dimensions: depression, anxiety, and stress. A 4-point evaluation was used, in which 0 represents “Strong match” and 3 represents “Strong mismatch.” The internal consistency coefficient *α* of the scale was 0.964. The fitting index of the confirmatory factor analysis was as follows: *χ*^2^/d*f* = 3.502, RMSEA = 0.043, NFI = 0.869, RFI = 0.851, TLI = 0.862, and CFI = 0.878.

#### 3.2.5. Test for Multicollinearity

The VIF value was used to evaluate multicollinearity among the variables, with a value of >10 representing high multicollinearity [43]. SPSS 23.0 was used to perform the multicollinearity test. Results revealed that the VIF value of all variables was less than 5, indicating that no multicollinearity was found among variables.

### 3.3. Statistical Analysis

IBM SPSS23.0 statistical software and Hayes' (2013) Process plug-in were used for the statistical analysis of the data. A confirmatory factor analysis of all questionnaires was performed using Amos 21.0. First, IBM SPSS23.0 was used to test for common method bias and multicollinearity. Pearson correlation analysis was used to calculate the relationships among metacognition, attitude toward physical exercise, negative emotion, and health-related behaviors. Normally distributed continuous variables were expressed as *M* ± SD. Finally, to test the mediating effect of attitude toward physical exercise between metacognition and health-related behavior and the regulating effect of emotion, Model 7 in the SPSS macroprogram compiled by Hayes was applied to the collected data. The age and gender of the participants did not differ significantly and were therefore not considered as control variables [44]. The significance level was set at *P* < 0.01.

## 4. Results

### 4.1. Common Method Deviation Test

To avoid common method bias, the Harman single-factor test was used to perform the exploratory factor analysis on the sum of all the items from the four questionnaires. The results showed that the characteristic root values of 18 factors were greater than 1, and the variation explained by the first factor was 23.857%, less than the critical standard of 40% [45, 46]. Therefore, there was no obvious common method bias.

### 4.2. Descriptive Statistics and Correlation Analysis

As Table 1 shows, metacognition is negatively correlated with attitude toward physical exercise and health-related behavior (*β* = −0.236, *p* < 0.01;*β* = −0.239, *p* < 0.01) and is positively correlated with negative emotions (*β* = 0.496, *p* < 0.01). Attitude toward physical exercise is positively correlated with health-related behavior (*β* = 0.533, *p* < 0.01) and negatively correlated with negative emotions (*β* = −0.336, *p* < 0.01). Negative emotion is negatively correlated with health-related behaviors (*β* = −0.389, *p* < 0.01).

### 4.3. Test of Moderated Mediation Model

**Figure 2:**
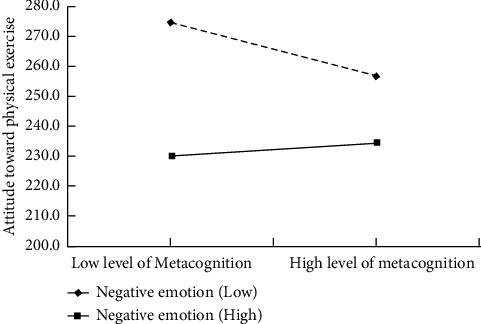
Regulating effect of negative emotion on metacognition and attitude toward physical exercise.

The test of the mediating effect of attitude toward physical exercise showed that metacognition has a significant negative predictive effect on health-related behavior (*β* = −0.067, *p* < 0.01); metacognition has a significant negative predictive effect on attitude toward physical exercise (*β* = −1.027, *p* < 0.01); and attitude toward physical exercise has a significant positive predictive effect on health-related behavior (*β* = 0.111, *p* < 0.01). The mediating effect value of attitude toward physical exercise was −0.073; the upper limit of the bootstrap 95% confidence interval was −0.088; and the lower limit was −0.163 (barring 0). This indicates that attitude toward physical exercise mediates between metacognition and health-related behavior.  Table 2 shows the regulating effect of negative emotion. After adding negative emotion to the model, the product terms of metacognition and negative emotion had a significant positive predictive effect on attitude toward physical exercise (*β* = 0.020, *p* < 0.01. Therefore, negative emotion plays a moderating role in the metacognition prediction of attitude toward physical exercise, and attitude toward physical exercise has a significant positive predictive role in health-related behaviors, *β* = 0.111, *p* < 0.01).  Table 3 shows the mediating effect of attitude toward physical exercise with different levels of negative emotion. When the level of negative emotion is low (*M* − 1SD), the mediating effect value of attitude toward physical exercise is −0.062, and its bootstrap 95% confidence interval does not include 0, which is statistically significant. When the level of negative emotion is high (*M* + 1SD), the mediating effect value of attitude toward physical exercise is 0.005, and its bootstrap 95% confidence interval includes 0, which is not statistically significant.  Figure 2 shows the regulating effect of negative emotions between metacognition and attitude toward physical exercise. For participants with low negative emotion (*M* − 1SD), metacognition had a significant negative predictive effect on attitude toward physical exercise (simple slope = −0.539, *t* = −4.686, *P* < 0.01). However, for participants with higher negative emotions (*M* + 1SD), metacognition had no significant predictive effect on attitude toward physical exercise (simple slope = 0.105, *t* = −0.863, *P* > 0.05). Based on the above results, the moderated mediation model proposed in this study is valid. Metacognition not only has a direct predictive effect on health-related behavior but also predicts health-related behavior through the mediating effect of attitude toward physical exercise. The first stage of the mediating effect is regulated by emotion.

## 5. Discussion

### 5.1. Metacognition and Health-Related Behavior

Our study found that metacognition has a significant negative predictive effect on health-related behavior, thereby verifying hypothesis 1. This finding is consistent with a previous study [47]. The higher the McQ-30 score, the higher the individuals' anxiety level, and the more likely they are to suffer from metacognitive disorder [41]. The result is in line with the metacognitive model of psychological disorders proposed by Wells [48]. Unhealthy behaviors tend to be more common in psychologically vulnerable adolescents, especially in females with low self-esteem and high anxiety, and metacognitive disorders might be an underlying factor in psychological vulnerability [49]. A research study [50] indicated that metacognitive beliefs about worry and anxiety could produce psychological disorders and aggravate a series of health-damaging behaviors. This may reflect the fact that metacognitive disorder leads to generalized anxiety [48]. Repeated anxiety and worry, as well as repeated negative thinking may amplify the aversion to negative emotional reactions and motivate individuals to engage in maladaptive coping behaviors [51], such as smoking, drinking, or excessive eating. Therefore, attention should be paid to adolescents' mental health problems, and the influence of negative metacognition on health-related behavior should be avoided in daily life and studies. Guiding students to adopt metacognitive strategies to maintain a positive attitude will also help them overcome poor behaviors and develop healthy behavior habits.

### 5.2. Mediating Effect of Attitude toward Physical Exercise

Our study found that attitude toward physical exercise has a positive predictive effect on health-related behavior. This finding is consistent with the results of a previous study [52]. Our study further proves the positive effect of metacognition on health-related behavior through attitude toward physical exercise, thereby verifying hypothesis 2. The health belief model emphasizes the use of individual attitudes and beliefs to explain and predict various health-related behaviors [53]. The change and maintenance of attitudes toward physical exercises play an important role in health-related behaviors. A positive attitude toward physical exercise reduces the generation of negative expectations (for example, it will cost more time and money to persist in physical exercise) and brings about positive outcome expectations (for example, it will reduce the chance of getting sick) [54]. A positive attitude toward physical exercise is conducive to developing physical exercise habits. The increase in physical exercise time will reduce sedentary behavior. It will increase nutrient intake and foster the development of regular sleep habits [55]. Attitude metacognition theory also reveals that attitude metacognition can affect subsequent cognition and behavior and plays an important role in the process of attitude change [21]. Negative metacognition enhances the degree of anxiety and worry and produces a negative attitude toward physical exercise. Meanwhile, the negative expectation and evaluation of physical exercise change the original cognition. Eventually, this leads to behaviors harmful to health. When individuals become aware that unhealthy behaviors result in disease, passive metacognition will be forced to take effect. The result is that attention is paid to physical exercise, and a gradual change in attitude toward physical exercise occurs. The change in attitude toward physical exercise will stimulate the development of health-related behaviors (e.g., regular sleep and eating, controlled smoking, and drinking). The result is consistent with previous research [56, 57], in which people with positive attitudes toward physical exercise tended to be more disciplined and had fewer unhealthy behaviors. In short, maintaining a positive attitude toward physical exercise is of great significance in cultivating awareness of health-related behaviors.

### 5.3. The Regulating Effect of Negative Emotion

Our study found that the mediating effect of attitude toward physical exercise between metacognition and health-related behavior is regulated by emotion. The regulating effect was significant in the group with low negative emotion levels but not in the group with high negative emotion levels, thereby verifying hypothesis 3. This finding is in line with previous research [58]. Specifically, metacognition has a more significant effect on attitudes toward physical exercise when the negative emotion level is lower. The results of a simple effect analysis showed that the predictive effect of metacognition on attitude toward physical exercise decreased significantly with the increase in negative emotion level, consistent with the cumulative ecological risk model [59]. The model explains that the accumulation of multiple risk factors (metacognition and negative emotion) can strengthen an individual's negative attitude and behavior. Physical education teachers should pay more attention to students with metacognitive disorders and negative emotions, arouse their enthusiasm in the physical education class, follow the principle of teaching from easy to difficult, and increase the interesting nature of the teaching content so that students can experience more enjoyment and develop a better attitude toward physical exercise. However, individuals with a low level of negative emotion bear a significantly more positive attitude toward physical exercise than individuals with a high level of negative emotion, indicating that positive emotion may improve students' attitude toward physical exercise. For this to happen, the intervention on health-related behavior of high school students should not only avoid the influence of students' negative and tired attitudes (induced by negative emotions) toward physical exercise but also consider the potential health-risk behavior caused by metacognition disorder. Therefore, for individuals with low levels of negative emotion, cultivating a good attitude toward physical exercise should be considered as a way to develop good health-related behavior. Improving metacognition should also be considered as a way of avoiding health-related behavior habits or lifestyles.

## 6. Limitations and Prospects

This study used a moderated mediation model to investigate the relationships among metacognition, attitude toward physical exercises, emotions, and health-related behaviors. It revealed the correlation mechanism between metacognition and the health-related behavior of high school students. It provided important theoretical and practical value for training the health-related behaviors of high school students and provided a basis for studying the relationship between or among relevant variables. However, the causal relationship among the variables cannot be illustrated. In the future, experimental intervention or longitudinal follow-up should be conducted to better explain the effects of metacognition on health-related behavior. In addition, only attitude toward physical exercise and mood were considered influential factors in this study. However, there are other mediating and moderating variables, such as internal (personality, self-esteem, etc.) and external environmental factors, which need to be further explored. This study used a sample of high school students. Therefore, the model's applicability to other age groups needs to be investigated. Future research should include different samples. Moreover, only physical exercise and emotion were considered influential factors in our study. Some other mediating and regulating variables need to be explored further, such as intrapersonal factors (personality, self-esteem, etc.), family status, regional culture, and external environment.

## 7. Conclusions

Metacognition has a significant negative predictive effect on health-related behavior. Attitude toward physical exercise plays a mediating role between metacognition and health-related behavior, and negative emotion plays a regulating role between metacognition and attitude toward physical exercise. Metacognition has a significant negative predictive effect on attitude toward physical exercise in individuals with a low level of negative emotion. Nevertheless, metacognition has no significant negative predictive effect on attitude toward physical exercise in individuals with a high level of negative emotion.

## Figures and Tables

**Table 1:** Descriptive statistics and correlation analysis.

	*M*	SD	1	2	3	4
(1) Metacognition	79.112	16.488	1			
(2) Attitude toward physical exercise	251.381	43.891	−0.236^*∗∗*^	1		
(3) Negative emotion	38.062	15.122	0.496^*∗∗*^	−0.336^*∗∗*^	1	
(4) Health-related behavior	48.562	9.686	−0.239^*∗∗*^	0.533^*∗∗*^	−0.389^*∗∗*^	1

*N* = 869; ^*∗∗*^*p* < 0.01; ^*∗*^*p* < 0.05.

**Table 2:** Test of the moderated mediation model.

Regression equation (*n* = 869)		Adjustment of fitting index			Coefficient significance	
Outcome variable	Predictable variable	*R* ^*2*^	*R* ^*2*^	*F*	*β*	*t*
Attitude toward physical exercise		0.158	0.151	26.895^*∗∗*^		
	Metacognition				−1.027	−5.371^*∗∗*^
	Negative emotions				−2.670	−6.295^*∗∗*^
	Metacognition and negative emotions				0.020	4.489^*∗∗*^

Health-related behaviors		0.333	0.328	86.225^*∗∗*^		
	Metacognition				−0.067	−3.949^*∗∗*^
	Attitude toward physical exercise				0.111	17.304^*∗∗*^

All variables in the model are standardized.

**Table 3:** Mediating the effect of different negative emotion levels on attitude toward physical exercise.

Mediating variables	Level	Level value	Effect	BootSE	BootLLCI	BootULCI
Attitude toward physical exercise	*M *−* *1SD	22.940	−0.062	0.014	−0.089	−0.036
	*M*	38.062	−0.028	0.012	−0.052	−0.004
	*M + *1SD	53.185	0.005	0.016	−0.027	0.037

BootLLCI refers to the 95% lower limit of bootstrap sampling. BootULCI refers to the 95% upper limit of bootstrap sampling.

## Data Availability

The original contributions presented in the study are included in the article/Supplementary Materials, further inquiries can be directed to the corresponding authors.
