# Supplementary material for: Impact of Metacognition on Health-Related Behavior: A Mediation Model Study
Source: J Environ Public Health. 2023 Jan 31;2023:6547804. doi: 10.1155/2023/6547804 (PMC9904901; doi:10.1155/2023/6547804)
Supplement: Supplementary Materials — The Supplementary Material for this article can be found online. [file 6547804.f1.pdf]

serial nu	Gender	Grede	Age	A1	A2	A3	A4	A5
1	2	3	18	4	4	3	2	4
2	2	3	17	4	3	4	4	3
3	1	3	18	3	3	3	3	3
4	2	3	17	1	2	3	2	3
5	2	3	18	4	4	4	4	4
6	1	2	17	3	4	2	3	4
7	2	2	17	3	3	2	4	3
8	2	2	17	3	3	4	1	2
9	1	3	17	4	1	4	1	4
10	1	2	18	4	3	4	2	4
11	2	2	18	4	2	3	4	2
12	1	2	18	3	2	4	3	3
13	2	3	18	4	2	4	4	4
14	1	2	18	4	4	4	4	4
15	2	2	18	4	2	3	3	2
16	2	3	18	4	3	4	1	1
17	1	2	18	3	3	3	2	2
18	2	2	18	4	4	4	4	4
19	2	3	18	3	2	4	1	3
20	2	2	18	4	1	4	4	2
21	2	2	18	3	2	2	2	3
22	2	2	18	4	1	3	2	1
23	2	2	18	4	3	4	1	4
24	1	2	18	3	3	2	3	2
25	2	2	18	1	3	3	4	4
26	1	2	18	3	4	4	4	3
27	2	3	18	3	2	3	4	4
28	2	3	18	3	2	3	3	3
29	1	3	18	1	3	4	4	3
30	2	3	18	4	4	4	3	4
31	2	2	18	4	3	4	3	3
32	2	2	18	4	3	4	1	4
33	2	3	18	2	2	3	2	3
34	1	3	18	3	3	3	3	3
35	1	3	18	4	1	4	4	2
36	2	3	18	4	4	1	4	4
37	2	3	18	4	3	4	3	4
38	1	3	18	4	4	4	4	4
39	1	3	18	4	2	3	4	2
40	1	3	18	4	4	4	4	4
41	2	3	18	2	1	3	1	3
42	2	3	18	3	1	3	1	3
43	1	3	18	3	3	3	3	3
44	1	3	18	3	3	3	3	3
45	2	3	18	4	1	1	1	1
46	2	3	18	4	4	4	4	4
47	1	3	18	3	3	1	1	4
48	1	3	18	4	4	4	4	4
49	1	3	18	1	1	1	1	2
50	1	3	18	4	4	4	4	3
51	1	3	18	4	4	4	4	4
52	1	3	18	3	1	2	1	4
53	1	3	18	2	2	3	2	4

54	1	3	18	3	3	4	3	3
55	1	3	18	4	3	4	3	3
56	1	2	18	4	4	4	4	4
57	1	1	16	2	2	2	1	1
58	1	1	16	4	4	3	2	2
59	1	1	16	4	4	4	1	4
60	1	1	16	3	1	1	1	2
61	1	1	16	1	1	1	1	1
62	1	1	16	4	2	4	3	3
63	1	2	18	3	3	4	4	3
64	2	1	16	2	3	4	3	3
65	2	1	16	4	2	4	2	3
66	1	1	16	4	1	4	1	4
67	1	1	16	1	1	4	1	4
68	1	1	15	4	1	4	1	3
69	1	1	15	4	1	4	1	4
70	2	1	15	1	1	1	1	1
71	2	1	15	4	4	3	2	4
72	1	1	15	1	3	4	1	4
73	2	1	15	4	3	3	3	3
74	2	1	15	3	3	3	2	3
75	2	1	15	4	2	4	4	4
76	1	1	15	3	3	2	4	2
77	2	1	18	4	3	2	4	4
78	2	1	18	1	1	3	1	3
79	1	1	18	3	4	4	3	4
80	2	1	18	1	1	3	2	4
81	1	1	18	3	3	3	3	3
82	2	1	18	2	4	1	4	1
83	2	1	18	4	2	4	1	1
84	2	1	18	3	1	3	2	2
85	2	1	18	2	4	2	4	4
86	1	1	18	3	2	3	2	3
87	2	1	18	3	4	4	4	2
88	1	1	18	3	3	4	3	2
89	2	1	18	4	1	3	1	4
90	2	1	18	4	1	4	1	4
91	2	1	18	3	2	2	3	4
92	1	1	18	4	3	4	2	4
93	2	1	18	4	2	4	2	4
94	1	1	18	3	3	3	3	3
95	1	1	18	3	2	3	2	3
96	1	1	18	4	4	1	4	1
97	2	1	18	4	3	4	1	3
98	1	1	18	2	3	4	2	4
99	1	1	18	4	3	4	3	3
100	1	2	18	2	3	3	2	3
101	1	1	18	4	1	3	3	4
102	2	1	18	4	1	4	1	4
103	1	1	18	3	1	4	1	4
104	2	1	17	2	1	3	1	3
105	2	1	17	4	1	4	1	4
106	2	1	17	4	2	3	3	1
107	1	1	17	3	3	3	4	3

108	1	1	17	4	3	3	3	3
109	1	1	17	3	3	3	3	4
110	2	1	17	4	3	3	2	3
111	2	1	17	4	4	4	4	4
112	2	2	17	4	2	4	3	4
113	2	2	17	4	4	4	3	4
114	2	1	16	4	3	4	1	3
115	1	2	17	4	4	4	1	4
116	2	1	16	4	3	3	1	4
117	1	2	17	3	4	4	4	4
118	1	2	17	3	4	3	4	3
119	1	2	17	3	1	2	2	2
120	1	2	17	4	2	3	3	3
121	2	1	16	4	3	4	3	4
122	2	1	16	4	1	3	3	4
123	2	1	16	3	3	3	4	3
124	2	1	16	4	3	3	3	2
125	1	2	17	2	1	3	1	4
126	2	2	17	3	3	4	3	4
127	1	1	16	4	4	4	4	4
128	1	1	16	3	2	3	2	3
129	1	1	16	4	4	4	4	4
130	2	1	16	4	4	4	4	4
131	2	1	16	4	4	4	4	3
132	2	2	17	4	3	3	2	3
133	2	2	17	3	3	2	3	2
134	2	2	17	2	3	3	2	3
135	2	1	16	4	2	3	3	3
136	2	2	17	3	4	2	4	3
137	1	2	17	4	4	4	4	4
138	2	1	16	4	1	4	4	4
139	2	2	17	1	1	4	1	3
140	1	1	16	3	3	3	3	3
141	2	2	17	1	1	4	2	2
142	1	1	16	4	1	3	1	4
143	2	2	17	3	3	3	1	4
144	2	1	16	1	1	1	1	1
145	2	1	16	2	1	1	1	3
146	2	2	17	4	4	3	1	3
147	1	2	17	2	1	4	2	2
148	2	1	16	3	4	4	3	4
149	2	1	16	4	4	3	3	2
150	1	1	16	4	3	1	4	4
151	2	2	17	3	2	2	3	3
152	1	2	17	1	1	2	1	4
153	1	2	17	3	2	3	2	4
154	2	1	16	3	3	2	4	4
155	2	1	16	4	2	3	4	4
156	2	1	16	3	3	3	2	4
157	1	1	16	3	2	3	4	2
158	1	2	17	1	4	4	1	2
159	1	1	16	4	2	4	4	4
160	1	2	17	2	2	2	4	3
161	2	1	16	4	3	2	2	4

162	2	2	17	4	2	3	1	4
163	1	1	16	4	3	2	2	2
164	2	1	16	4	4	4	3	4
165	2	1	16	3	3	3	2	3
166	2	2	17	1	4	4	4	4
167	1	2	17	1	4	4	1	4
168	2	1	16	3	2	3	2	3
169	2	3	16	4	2	3	1	3
170	2	2	17	2	4	2	4	3
171	1	1	16	3	2	3	4	4
172	2	1	16	4	3	4	2	4
173	2	1	16	1	1	3	3	3
174	2	1	16	4	3	3	2	3
175	1	1	16	4	1	4	1	4
176	2	1	16	2	2	3	2	2
177	2	2	17	4	1	4	1	4
178	1	1	16	3	2	2	2	3
179	1	1	16	3	2	3	2	2
180	1	2	17	3	4	4	4	4
181	1	1	16	4	2	3	1	4
182	1	1	16	4	3	3	2	3
183	2	2	17	4	2	4	1	4
184	2	1	16	3	1	3	3	3
185	2	1	16	1	1	3	1	3
186	2	1	16	1	4	4	1	1
187	1	2	17	3	2	4	2	3
188	2	2	17	4	2	4	3	3
189	1	1	16	4	4	4	3	3
190	1	1	16	1	4	2	3	2
191	2	2	17	4	4	4	4	4
192	2	2	17	3	1	3	1	2
193	2	2	17	4	4	4	1	4
194	1	2	17	4	2	3	2	3
195	2	1	16	4	2	2	3	4
196	1	2	17	4	3	3	3	2
197	1	2	18	3	3	3	3	3
198	2	2	18	1	2	2	3	3
199	2	1	16	3	1	4	4	4
200	1	2	18	4	2	3	2	3
201	1	1	16	4	3	4	4	3
202	2	1	16	4	4	4	4	4
203	2	2	18	3	3	3	3	3
204	1	2	18	4	1	4	4	3
205	2	1	16	3	2	3	2	4
206	2	2	18	1	1	3	3	2
207	2	2	18	3	3	3	2	3
208	1	2	18	4	2	2	2	4
209	2	2	18	1	4	4	1	4
210	1	2	18	4	3	4	3	4
211	1	1	16	1	1	4	4	4
212	1	2	18	4	1	1	1	4
213	1	1	16	3	2	4	1	3
214	1	2	18	4	2	4	1	4
215	1	1	16	4	3	3	4	4

216	2	1	16	4	4	4	3	4
217	1	2	18	4	4	4	4	4
218	1	2	18	4	4	4	4	4
219	2	2	18	2	3	2	2	2
220	2	1	16	3	2	3	3	3
221	1	1	16	4	4	4	4	4
222	2	2	18	4	4	4	4	4
223	2	2	18	1	1	4	2	4
224	2	2	18	4	3	4	4	4
225	2	2	18	3	2	3	2	3
226	2	2	18	4	4	4	1	4
227	1	1	16	4	2	4	4	4
228	2	2	18	1	1	4	2	4
229	2	1	16	1	2	4	2	3
230	1	2	18	4	2	4	1	4
231	1	3	18	4	3	4	3	4
232	1	1	16	4	4	3	3	3
233	1	2	18	3	1	3	1	3
234	2	2	18	3	2	3	1	3
235	2	2	18	3	2	3	3	3
236	1	2	18	3	3	3	2	4
237	2	2	18	3	4	4	4	3
238	2	2	18	4	2	3	3	4
239	1	2	18	3	4	2	1	2
240	1	2	18	4	4	3	4	4
241	1	2	16	4	2	3	3	4
242	2	2	16	2	2	2	2	2
243	2	2	16	4	4	4	2	4
244	2	2	16	4	4	4	4	4
245	2	2	16	3	1	4	1	3
246	2	2	16	2	2	2	2	2
247	2	1	16	2	4	4	4	1
248	1	3	18	3	4	3	3	3
249	1	2	16	4	4	4	4	4
250	2	1	16	4	1	4	1	3
251	2	1	16	4	4	4	3	3
252	2	2	16	4	2	4	1	4
253	1	2	16	4	3	2	4	4
254	1	2	16	4	4	4	4	4
255	1	1	16	4	1	4	1	4
256	1	1	16	4	2	3	3	4
257	1	1	16	3	3	4	3	3
258	1	2	16	1	2	4	4	2
259	1	2	16	4	4	4	4	4
260	2	1	16	4	4	3	4	3
261	2	2	16	4	3	3	3	4
262	1	1	16	3	3	3	4	4
263	1	1	16	4	4	4	3	3
264	1	1	16	4	4	4	3	3
265	1	1	16	4	1	4	2	3
266	1	2	16	4	1	4	2	3
267	1	1	16	1	1	4	1	4
268	2	2	16	3	2	3	1	3
269	2	2	16	3	2	2	2	2

270	2	2	16	4	1	3	2	4
271	1	2	16	4	3	4	1	4
272	1	1	16	3	2	1	1	3
273	1	1	16	3	3	3	3	3
274	1	2	16	3	2	3	2	4
275	1	2	16	2	3	3	3	3
276	1	2	16	3	3	3	3	3
277	1	2	16	4	4	4	2	2
278	1	1	16	2	1	4	4	1
279	2	2	16	2	2	2	3	3
280	2	1	16	4	2	3	1	3
281	2	2	16	4	4	4	3	3
282	2	1	16	4	3	4	4	4
283	1	1	16	1	1	3	1	3
284	1	1	16	4	4	4	2	4
285	1	1	16	4	3	3	3	3
286	2	2	16	4	1	4	4	4
287	1	2	16	1	1	1	1	1
288	1	2	16	1	1	1	1	1
289	2	3	18	3	3	3	3	2
290	1	3	18	4	4	4	4	4
291	1	3	18	3	3	3	3	3
292	2	3	18	4	3	4	3	4
293	2	3	18	3	2	3	4	3
294	1	3	18	3	3	3	3	3
295	1	3	18	4	2	4	2	4
296	1	2	16	2	2	3	3	1
297	2	3	18	3	2	3	2	2
298	1	1	16	4	1	4	4	1
299	2	1	16	4	4	4	1	4
300	2	1	16	1	4	4	4	3
301	2	1	16	3	1	2	1	4
302	2	1	16	1	4	3	2	2
303	2	1	16	3	2	3	1	3
304	1	3	18	3	2	3	2	3
305	2	3	18	1	1	2	2	1
306	1	3	18	4	3	4	3	3
307	2	3	18	4	1	3	1	3
308	2	3	18	4	3	3	3	3
309	1	3	18	4	2	4	3	3
310	2	3	18	3	3	3	3	3
311	2	3	18	4	3	4	4	4
312	1	3	18	2	2	3	1	3
313	2	3	18	3	3	3	3	3
314	2	3	18	3	3	3	3	3
315	2	3	18	2	3	3	3	3
316	2	3	18	3	1	3	3	3
317	1	3	18	1	3	3	1	3
318	1	3	18	1	1	2	1	2
319	2	3	18	2	2	4	3	2
320	1	3	18	4	2	2	3	2
321	2	3	18	4	1	4	1	4
322	2	3	18	4	4	4	3	2
323	2	3	18	3	3	3	3	2

324	2	3	18	4	3	4	2	4
325	2	3	18	2	2	3	3	3
326	2	3	18	4	1	4	4	2
327	2	3	18	4	4	4	4	3
328	1	3	16	4	4	4	4	4
329	2	3	18	3	3	4	4	2
330	1	3	18	4	1	4	1	4
331	2	3	18	3	4	3	3	3
332	2	3	18	3	3	2	3	3
333	2	3	18	4	4	4	4	4
334	2	3	18	3	2	4	3	3
335	2	3	18	3	3	3	3	3
336	1	3	18	4	4	4	2	4
337	2	3	18	2	3	3	3	3
338	2	3	18	4	3	2	1	4
339	2	3	18	4	4	4	4	4
340	1	3	18	4	4	4	4	4
341	2	3	18	3	2	3	2	3
342	2	3	18	3	4	3	4	3
343	2	3	18	4	3	2	3	3
344	2	3	18	3	2	2	4	3
345	2	3	18	3	3	3	4	3
346	2	3	18	4	3	4	1	4
347	2	3	18	4	1	4	2	4
348	2	3	18	3	2	2	1	1
349	2	3	18	4	4	4	3	4
350	1	3	18	3	3	3	4	3
351	2	3	18	4	2	4	3	3
352	2	3	18	3	3	2	3	3
353	2	3	18	3	2	3	3	2
354	2	3	18	1	3	3	4	3
355	2	3	18	2	3	3	3	3
356	1	3	18	4	4	4	4	3
357	1	3	18	1	3	4	4	3
358	1	3	18	4	3	3	1	3
359	1	3	18	3	3	3	3	1
360	2	3	18	3	3	4	4	4
361	2	3	18	4	3	3	3	3
362	2	3	18	4	2	3	1	3
363	2	3	18	4	4	1	4	4
364	2	3	18	4	2	3	3	2
365	2	3	18	4	2	4	4	3
366	2	3	18	3	3	3	2	2
367	2	3	18	3	2	3	1	2
368	2	3	18	3	2	3	4	4
369	1	3	18	3	4	4	4	4
370	2	3	18	4	1	4	1	4
371	1	3	18	3	3	3	3	3
372	1	3	18	1	1	3	1	3
373	1	3	18	4	3	4	3	3
374	1	3	18	4	1	1	1	1
375	1	3	18	4	1	3	2	3
376	1	3	18	3	3	3	3	2
377	1	3	18	1	1	3	2	3

378	1	3	18	3	2	3	2	3
379	1	3	18	4	4	4	4	4
380	1	3	18	4	2	4	3	3
381	1	3	18	3	2	2	3	3
382	1	3	18	3	3	2	3	2
383	2	3	18	4	1	4	1	4
384	1	3	18	3	2	3	1	3
385	2	3	18	4	2	2	2	3
386	1	3	18	4	2	4	3	4
387	2	3	18	4	3	4	2	4
388	2	3	18	4	3	3	3	3
389	2	3	18	4	4	4	4	3
390	1	3	18	3	2	3	1	3
391	2	3	18	3	4	3	3	4
392	1	3	18	4	1	2	1	2
393	1	3	18	3	4	4	4	4
394	2	3	18	3	1	4	2	3
395	2	3	18	3	2	2	4	3
396	1	3	18	4	4	4	4	4
397	1	3	18	4	3	4	4	4
398	1	3	18	3	3	4	1	4
399	2	3	18	4	1	3	1	3
400	1	3	18	3	2	3	3	3
401	1	3	18	3	3	4	2	4
402	2	3	18	1	4	2	4	2
403	2	3	18	1	1	1	1	2
404	1	3	18	3	1	3	1	3
405	2	3	18	4	3	3	4	3
406	2	3	18	1	1	2	2	1
407	2	3	18	4	4	3	4	4
408	1	1	16	3	4	3	2	3
409	1	3	18	3	3	3	3	3
410	1	3	18	3	2	3	3	4
411	2	1	16	3	1	2	1	3
412	2	2	18	4	3	4	3	3
413	2	3	18	4	2	3	4	1
414	1	3	18	4	4	4	4	4
415	2	3	18	2	4	3	4	4
416	2	3	18	4	4	4	1	4
417	2	3	18	4	3	3	3	3
418	2	3	18	4	1	4	2	3
419	2	3	18	1	2	4	3	3
420	2	3	18	4	2	3	3	2
421	2	3	18	2	2	3	2	4
422	1	3	18	3	3	3	3	3
423	2	3	18	4	1	4	4	4
424	2	3	18	2	1	3	3	3
425	2	3	18	2	4	4	4	1
426	1	3	18	4	4	4	4	4
427	2	3	18	2	2	3	3	3
428	2	3	18	1	4	4	4	3
429	2	3	18	4	1	4	1	3
430	2	3	18	4	1	3	1	2
431	2	3	18	4	3	4	2	4

432	2	3	18	4	3	4	4	3
433	2	3	18	3	4	4	4	2
434	2	3	18	4	4	4	4	4
435	2	3	18	2	2	4	3	3
436	2	3	18	3	2	4	2	2
437	2	3	18	3	2	4	3	3
438	2	3	18	4	3	3	1	3
439	2	3	18	4	1	4	1	3
440	1	3	18	4	4	4	3	3
441	1	3	18	4	4	4	4	4
442	2	3	18	4	3	4	1	3
443	1	3	18	3	2	4	1	2
444	1	3	18	3	3	3	3	3
445	2	3	18	1	4	1	1	2
446	2	2	18	4	2	4	4	4
447	2	2	18	4	1	4	1	4
448	2	3	18	3	4	4	4	4
449	2	2	18	3	2	3	3	3
450	2	3	18	4	2	3	2	3
451	2	2	18	4	4	4	4	4
452	2	2	18	4	3	3	4	4
453	1	2	18	4	4	4	3	3
454	2	3	18	4	1	3	3	4
455	2	3	18	3	2	3	2	3
456	2	2	18	4	2	4	3	4
457	1	2	18	4	2	4	4	2
458	1	3	18	4	3	3	1	3
459	2	3	18	3	3	4	2	2
460	2	2	18	4	3	4	4	4
461	2	2	18	3	3	3	3	3
462	2	3	18	4	2	2	2	2
463	2	3	18	4	2	4	2	4
464	1	3	18	4	3	3	3	2
465	2	2	18	3	3	3	2	3
466	2	2	18	4	4	4	1	4
467	2	3	18	4	3	3	4	3
468	2	3	18	3	1	3	1	4
469	2	3	18	3	3	2	2	3
470	2	3	18	2	3	4	2	3
471	2	3	18	3	3	4	2	3
472	2	3	18	3	3	3	3	2
473	2	3	18	4	3	4	4	4
474	2	3	18	4	3	3	3	3
475	2	3	18	4	1	3	1	2
476	2	3	18	4	1	4	4	4
477	2	3	18	2	2	3	2	3
478	1	3	18	3	4	3	3	3
479	2	3	18	4	3	3	1	3
480	2	3	18	4	2	4	1	4
481	2	3	18	3	2	3	2	3
482	1	3	18	1	3	3	3	2
483	2	3	18	3	2	3	2	3
484	2	3	18	3	3	2	3	3
485	2	3	18	4	4	4	4	2

486	2	2	16	3	3	3	3	3
487	1	2	16	2	2	2	2	2
488	1	2	16	4	1	3	1	3
489	2	2	16	4	2	3	1	1
490	1	2	16	3	3	3	3	3
491	1	3	18	3	2	3	2	3
492	2	2	16	4	2	2	2	2
493	2	2	16	4	2	3	2	3
494	1	2	16	3	3	4	4	2
495	2	3	18	4	3	3	3	3
496	1	2	16	4	2	2	4	3
497	2	2	16	1	3	3	1	3
498	2	2	16	4	2	2	4	2
499	2	2	16	3	2	3	1	4
500	2	2	16	3	2	3	2	3
501	1	2	16	3	3	4	3	3
502	1	2	16	2	3	3	4	2
503	2	2	16	2	3	4	3	3
504	1	2	18	2	2	3	3	3
505	2	2	16	3	3	4	4	3
506	2	3	18	4	2	4	4	4
507	2	2	16	4	2	4	3	3
508	2	3	18	2	2	2	2	2
509	2	2	16	3	1	3	4	3
510	1	2	16	4	2	4	3	4
511	2	2	16	4	1	4	4	4
512	2	3	18	3	2	3	1	3
513	1	2	16	3	3	3	3	3
514	2	2	16	2	3	3	3	3
515	2	3	18	3	4	4	3	2
516	1	2	16	4	4	4	4	4
517	1	2	16	2	3	3	3	3
518	1	2	16	4	4	4	4	4
519	1	2	16	2	2	2	2	2
520	1	2	16	3	3	3	3	2
521	1	3	18	4	4	4	4	4
522	1	2	16	4	4	4	4	4
523	1	3	18	4	2	4	3	4
524	1	2	16	4	3	4	3	4
525	2	2	16	1	1	4	1	1
526	2	2	18	4	4	4	4	3
527	2	2	18	2	2	2	2	2
528	1	2	18	4	4	4	4	4
529	1	2	18	4	2	3	3	3
530	2	3	18	3	3	3	2	2
531	2	3	18	3	3	3	4	3
532	2	2	18	4	2	3	4	3
533	2	2	18	3	3	3	3	2
534	1	3	18	2	3	4	2	3
535	1	3	18	4	4	4	4	4
536	2	3	18	2	2	2	2	3
537	2	2	18	3	1	2	3	3
538	1	2	18	2	3	2	2	2
539	1	2	18	2	2	2	2	2

540	1	2	18	4	1	3	1	1
541	1	2	17	4	1	1	1	4
542	2	3	17	4	2	3	1	3
543	2	3	17	3	3	3	4	3
544	1	2	17	4	4	4	1	4
545	1	2	17	1	1	1	1	1
546	2	3	17	4	2	4	4	4
547	1	3	17	1	3	3	1	4
548	2	2	17	2	2	3	4	2
549	2	3	17	3	4	4	4	4
550	1	2	17	3	3	4	1	3
551	1	3	17	1	2	1	2	3
552	2	3	17	3	1	3	1	3
553	1	2	17	3	3	3	3	3
554	1	2	17	4	2	3	2	3
555	2	3	17	4	4	3	4	2
556	2	2	17	3	2	3	4	3
557	2	2	17	3	1	3	1	1
558	2	3	17	3	3	3	3	3
559	1	3	17	4	1	1	1	4
560	2	3	16	2	2	4	4	4
561	1	2	17	4	2	2	2	2
562	2	2	17	3	4	3	3	3
563	1	2	17	3	2	3	4	3
564	2	3	17	4	4	3	2	3
565	2	2	17	4	2	4	2	3
566	2	3	17	3	2	3	3	3
567	2	2	17	3	2	4	4	3
568	2	2	17	2	4	4	4	4
569	2	2	17	4	3	4	4	4
570	2	3	17	3	3	3	1	3
571	2	3	17	3	2	4	3	2
572	1	2	17	4	4	4	4	4
573	2	2	17	4	3	3	3	2
574	2	2	17	3	2	4	1	2
575	1	2	17	3	3	3	3	3
576	2	2	17	2	2	3	2	3
577	1	2	17	3	2	2	2	2
578	1	1	16	4	2	3	1	4
579	2	3	17	2	2	3	3	3
580	1	2	17	4	4	4	4	4
581	1	1	16	1	1	1	1	4
582	1	2	17	3	2	4	1	3
583	2	2	17	3	2	4	2	3
584	2	2	17	4	3	4	2	4
585	1	2	17	3	3	3	3	3
586	2	2	17	4	4	4	4	4
587	2	1	16	3	3	3	3	3
588	1	1	16	3	4	4	4	4
589	2	1	16	3	2	3	3	3
590	2	1	16	3	2	3	4	4
591	2	1	16	4	4	2	4	2
592	1	1	16	4	4	4	4	4
593	2	1	16	3	2	4	1	4

594	2	1	16	4	1	4	1	4
595	2	1	16	3	3	4	3	3
596	2	1	16	2	1	3	1	3
597	1	1	16	3	2	3	2	3
598	2	1	16	4	3	3	4	4
599	1	1	16	4	1	4	1	3
600	2	1	16	4	2	2	1	3
601	1	1	16	3	2	3	2	4
602	1	1	16	4	4	3	3	3
603	1	1	16	4	1	4	1	4
604	1	1	16	3	2	3	3	3
605	1	1	16	3	2	3	2	4
606	2	1	16	2	2	3	1	2
607	1	1	16	4	4	4	4	4
608	1	1	16	4	3	3	4	3
609	2	1	16	4	4	4	4	4
610	2	1	16	1	2	3	1	4
611	1	1	16	4	4	4	4	4
612	1	1	16	4	4	4	4	4
613	2	1	16	1	1	1	1	4
614	2	1	16	3	3	4	4	3
615	1	1	16	1	2	4	1	4
616	1	1	16	3	2	4	2	4
617	2	1	16	3	3	3	1	4
618	1	1	16	4	4	4	1	4
619	1	1	16	4	1	4	2	4
620	1	1	16	4	4	4	3	4
621	2	1	16	2	2	3	1	4
622	2	3	17	3	4	4	3	2
623	2	1	16	1	1	1	1	4
624	2	1	16	3	2	3	4	3
625	1	1	16	4	4	4	4	4
626	2	1	16	4	1	4	1	4
627	1	1	16	4	1	4	1	4
628	2	3	17	3	3	4	4	3
629	2	3	17	2	2	3	1	3
630	2	1	16	4	3	3	3	3
631	1	1	16	4	1	4	1	4
632	2	1	16	1	1	1	1	4
633	2	1	16	4	3	4	4	4
634	2	1	16	4	3	4	3	4
635	1	1	16	1	1	4	1	4
636	2	1	16	4	1	4	1	4
637	2	1	16	3	2	3	2	4
638	1	1	16	1	2	2	1	1
639	1	1	16	4	4	4	4	4
640	2	1	16	4	1	4	1	4
641	1	1	16	4	3	4	4	4
642	1	1	16	4	3	3	3	3
643	2	1	16	4	2	4	3	4
644	2	1	16	2	3	2	3	2
645	1	1	16	4	4	4	4	4
646	1	1	16	3	4	3	2	3
647	2	1	16	3	2	4	1	4

648	1	1	16	4	1	2	3	4
649	2	1	16	3	3	2	2	3
650	1	1	16	3	2	4	1	4
651	2	1	16	3	4	4	4	4
652	1	1	16	4	4	4	4	4
653	2	1	16	4	4	4	1	4
654	2	1	16	3	3	3	3	3
655	2	1	16	4	4	1	4	4
656	1	1	16	4	2	3	2	4
657	1	1	16	4	4	4	4	4
658	1	1	16	1	1	1	1	4
659	2	1	16	3	3	4	4	3
660	1	1	16	1	1	1	1	4
661	2	1	16	4	4	4	3	4
662	1	2	17	3	1	3	1	3
663	1	1	16	4	2	4	1	4
664	1	2	17	3	1	3	1	3
665	2	1	16	4	1	2	1	4
666	1	1	16	3	1	3	2	3
667	1	1	16	2	2	3	2	3
668	1	1	16	1	1	3	1	3
669	2	1	16	3	3	3	3	3
670	1	1	16	4	4	4	4	4
671	1	1	16	4	1	4	4	4
672	1	2	17	2	2	2	2	2
673	1	2	17	3	3	3	3	3
674	1	2	17	3	3	3	2	2
675	1	2	17	3	3	3	2	3
676	2	2	17	4	3	3	4	3
677	2	2	17	2	1	4	4	3
678	2	2	17	3	3	3	3	2
679	1	3	17	2	3	3	3	3
680	2	3	17	4	1	4	1	3
681	1	1	16	3	2	3	3	3
682	1	2	17	4	1	3	3	4
683	2	2	17	3	3	3	3	3
684	1	2	17	3	2	3	1	2
685	2	3	17	3	3	3	1	2
686	1	2	17	4	2	3	3	3
687	1	2	17	3	3	3	3	3
688	1	2	17	4	4	4	4	3
689	2	2	17	4	2	4	2	3
690	2	2	17	4	4	4	2	4
691	2	2	17	3	2	2	2	2
692	2	2	17	3	3	4	4	3
693	1	1	16	1	4	1	1	4
694	2	2	17	3	4	4	4	3
695	2	2	17	2	2	2	1	3
696	2	2	17	3	2	3	3	3
697	2	2	17	2	4	4	3	2
698	2	1	16	3	2	4	2	4
699	1	2	17	2	1	1	1	3
700	2	2	17	3	3	3	3	3
701	1	3	17	3	2	2	3	2

702	1	2	17	4	4	4	2	4
703	2	2	17	3	3	3	3	2
704	2	1	16	3	3	4	3	3
705	2	3	17	3	3	3	3	3
706	2	2	17	4	4	1	4	1
707	1	3	17	3	4	4	4	4
708	1	2	17	4	4	4	4	4
709	2	2	17	4	3	2	4	2
710	1	1	16	3	3	3	2	3
711	1	2	17	4	2	2	1	4
712	2	1	16	4	4	4	4	4
713	2	1	16	4	4	4	4	4
714	1	3	17	3	2	2	2	3
715	2	1	16	3	2	3	3	4
716	1	1	16	4	4	4	1	4
717	1	1	16	4	3	4	3	4
718	2	3	17	1	2	3	1	3
719	2	3	17	3	3	3	3	3
720	2	2	17	3	3	3	2	3
721	2	3	17	4	1	3	1	3
722	1	3	17	4	4	4	4	4
723	2	3	17	3	3	3	3	3
724	2	3	17	3	2	2	3	3
725	1	3	17	3	2	4	2	4
726	1	3	17	4	4	2	2	3
727	2	3	17	2	1	4	4	2
728	1	3	17	4	3	4	2	4
729	2	2	17	2	4	4	4	3
730	1	3	17	4	3	4	4	4
731	2	3	17	4	4	4	3	2
732	1	3	17	1	1	3	2	4
733	2	3	17	3	2	3	2	3
734	2	3	16	3	3	3	3	3
735	1	2	17	2	2	2	2	2
736	2	3	17	4	4	2	3	1
737	1	3	17	3	2	4	2	4
738	2	2	17	3	1	3	2	3
739	1	3	17	3	4	4	4	4
740	2	3	17	3	3	3	3	3
741	2	3	17	4	2	2	2	2
742	1	3	17	2	3	2	3	3
743	2	3	17	3	2	3	2	3
744	1	3	17	4	4	4	4	4
745	2	3	17	2	2	3	1	3
746	2	2	17	3	3	3	3	3
747	2	3	17	4	2	3	2	3
748	2	2	17	3	3	3	3	3
749	1	2	17	3	3	3	3	3
750	1	3	17	3	3	1	2	3
751	2	2	17	4	3	4	4	4
752	2	3	17	4	2	4	1	4
753	1	2	17	4	1	3	1	3
754	2	2	17	2	2	3	1	4
755	2	2	17	3	3	3	2	3

756	2	3	17	3	2	3	3	3
757	1	3	17	4	3	3	2	3
758	2	3	17	4	2	2	1	3
759	1	3	17	4	2	3	2	3
760	2	3	17	3	3	4	4	4
761	2	2	17	3	2	3	1	3
762	1	2	17	2	1	1	1	3
763	1	2	17	4	4	4	4	4
764	1	2	17	3	2	4	1	2
765	1	2	17	2	4	3	3	3
766	1	2	17	2	4	3	3	3
767	1	2	17	4	4	3	4	4
768	1	2	17	4	2	2	1	4
769	1	3	18	3	2	3	2	3
770	1	2	17	3	4	4	1	1
771	1	1	16	4	1	1	1	1
772	1	2	17	1	1	3	1	3
773	2	2	17	1	3	4	3	3
774	1	1	16	4	4	4	1	4
775	2	1	16	4	2	4	3	4
776	1	2	17	1	2	4	3	3
777	2	1	16	4	3	4	4	4
778	1	1	16	1	1	4	1	4
779	1	1	16	4	3	4	2	3
780	2	2	17	4	4	4	1	4
781	2	1	16	4	2	4	2	3
782	1	1	16	2	1	2	1	2
783	2	1	16	4	4	4	1	3
784	1	1	16	4	3	3	3	3
785	1	1	16	4	1	4	1	4
786	2	1	16	4	4	2	1	4
787	1	1	16	4	3	3	1	3
788	2	1	16	1	2	1	4	2
789	1	1	16	3	2	3	3	4
790	1	1	16	4	4	4	4	4
791	1	1	16	2	1	4	1	2
792	2	2	17	2	2	3	3	3
793	2	2	17	4	1	4	2	3
794	1	1	16	3	1	4	1	3
795	2	1	16	3	2	2	1	3
796	2	1	16	3	1	4	1	3
797	2	1	16	4	2	2	3	1
798	1	1	16	4	4	4	4	4
799	2	1	16	2	2	4	2	3
800	2	1	16	3	2	3	2	3
801	1	1	16	3	3	4	2	4
802	2	1	16	3	1	4	3	3
803	1	1	16	4	4	3	3	4
804	1	1	16	3	3	2	2	3
805	2	1	16	3	2	3	1	3
806	2	2	17	1	1	1	2	4
807	2	1	16	3	2	3	3	4
808	2	1	16	3	3	3	3	2
809	1	1	16	1	3	3	1	3

810	2	1	16	2	3	3	4	3
811	2	1	16	4	2	3	2	4
812	1	1	16	4	1	4	1	4
813	2	1	16	3	2	2	2	3
814	1	2	17	3	3	4	3	4
815	2	1	16	3	2	2	3	2
816	1	1	16	3	3	2	1	2
817	2	1	16	4	3	4	1	4
818	1	1	16	2	2	3	1	3
819	2	1	16	4	2	4	3	3
820	2	1	16	1	1	3	2	4
821	1	2	17	4	4	4	2	3
822	1	1	16	4	2	4	1	2
823	1	1	16	4	1	4	1	4
824	1	1	16	4	1	4	1	4
825	1	2	17	2	2	3	1	1
826	1	2	17	4	3	4	3	3
827	1	2	17	3	2	2	1	2
828	1	2	17	3	1	3	1	3
829	2	3	17	2	1	3	3	3
830	2	2	17	3	4	4	4	4
831	2	3	17	3	3	3	4	2
832	1	3	17	2	1	2	1	3
833	1	3	17	3	3	3	1	3
834	2	2	17	4	3	3	3	3
835	2	2	17	3	2	3	2	3
836	2	2	17	3	3	3	2	3
837	2	3	17	4	3	4	1	3
838	2	2	17	4	1	2	2	2
839	2	2	17	2	2	3	2	3
840	2	2	17	4	1	1	1	2
841	2	2	17	3	2	3	2	3
842	2	2	17	2	1	4	1	3
843	1	2	17	3	3	3	2	2
844	1	2	17	2	3	3	3	3
845	1	2	17	3	3	3	2	3
846	1	2	17	4	3	3	3	3
847	1	2	17	4	4	3	2	3
848	1	2	17	3	3	2	2	3
849	1	2	17	4	1	1	1	3
850	1	2	17	4	3	4	3	4
851	1	2	17	4	3	4	4	4
852	2	2	17	3	3	3	3	2
853	1	2	17	3	3	3	1	4
854	1	2	17	4	2	2	2	2
855	1	2	17	3	4	4	4	1
856	1	1	16	4	3	4	4	4
857	1	2	17	4	3	3	3	3
858	1	2	17	2	2	3	3	3
859	1	2	17	2	1	2	2	4
860	1	2	17	4	3	4	1	3
861	1	2	17	2	3	3	3	2
862	1	2	17	3	2	3	4	4
863	2	2	17	2	3	3	3	3

864	1	2	17	4	1	1	1	4
865	1	2	17	4	2	3	3	2
866	1	2	17	3	3	3	2	2
867	1	2	17	4	3	3	4	4
868	1	2	17	4	2	2	1	3
869	2	2	17	2	3	3	3	3

A6	A7	A8	A9	A10	A11	A12	A13	A14
3	3	2	2	3	2	4	3	3
1	4	4	4	4	4	4	3	4
2	3	1	2	3	3	3	1	4
3	2	2	3	3	2	3	2	3
4	4	1	1	4	1	4	4	4
4	2	2	1	1	1	4	1	3
3	2	4	3	2	3	3	1	4
1	3	3	1	4	2	4	2	3
2	4	3	1	3	1	4	4	4
3	3	1	1	4	1	4	4	1
3	3	3	4	3	4	4	2	2
2	3	3	1	3	1	3	3	4
3	4	3	1	4	1	4	4	3
4	4	4	4	4	4	4	1	4
2	2	3	3	3	3	2	2	3
1	4	3	1	4	1	4	4	3
3	3	3	1	3	1	3	3	3
3	4	4	4	4	4	4	4	4
2	3	2	2	4	2	3	3	1
1	3	1	4	3	4	3	4	4
1	2	1	2	3	2	3	2	3
2	2	3	3	3	3	4	3	4
1	4	1	1	4	1	4	4	4
2	2	2	2	3	2	3	2	3
3	2	3	3	2	3	3	3	3
4	4	4	2	4	1	3	3	4
3	2	4	2	2	2	3	3	3
2	2	2	2	3	2	3	2	3
1	2	2	1	1	1	3	3	4
2	2	3	3	3	3	3	2	3
3	3	3	3	4	3	3	2	4
4	4	1	1	4	1	4	4	3
1	1	2	2	2	1	4	1	4
3	3	3	3	3	3	3	3	3
2	3	1	2	1	2	3	4	4
4	4	4	4	4	4	4	3	3
2	4	2	1	4	1	4	4	4
4	4	4	4	4	4	4	4	4
4	4	2	2	3	3	3	3	3
4	4	4	4	4	4	4	4	4
1	1	1	1	4	1	4	4	3
3	3	3	3	3	2	3	3	3
3	3	3	3	3	3	3	3	3
3	3	3	3	3	3	3	3	3
4	1	1	1	4	1	4	4	4
4	4	4	4	4	3	4	4	4
2	2	3	3	2	2	2	2	2
4	4	4	4	4	4	4	4	4
2	2	2	2	2	1	2	2	2
3	3	3	3	3	3	3	3	3
4	4	4	4	4	4	4	4	4
3	4	1	1	4	1	4	3	3
3	3	2	2	2	2	3	3	3

2	3	4	4	3	3	4	3	4
1	3	2	2	4	3	4	3	1
4	4	4	4	4	4	4	4	4
1	1	1	1	1	1	1	1	1
4	4	4	2	4	2	4	3	4
1	3	2	1	4	2	4	3	3
1	3	1	1	3	1	3	2	2
1	1	1	1	1	1	1	1	1
2	4	4	4	4	4	3	4	4
3	4	2	2	4	3	3	2	2
3	2	2	3	1	3	2	1	2
4	3	2	2	3	3	4	2	4
1	4	1	1	4	1	4	4	2
1	1	1	1	1	1	1	1	1
1	3	3	1	3	1	3	3	3
4	3	1	1	4	1	4	4	1
1	1	1	1	1	1	1	1	1
3	3	3	1	1	1	4	4	1
2	4	1	2	3	1	4	4	3
1	3	2	2	3	2	3	3	3
3	3	2	2	3	2	3	3	4
2	3	4	1	3	3	4	3	3
3	2	3	2	2	2	3	3	3
4	4	4	4	4	4	3	2	4
1	1	2	3	2	2	1	2	1
1	3	1	1	3	2	2	3	2
1	3	2	1	3	1	3	4	3
4	3	3	3	3	2	2	2	3
1	1	1	3	2	2	3	2	1
1	1	1	1	1	1	1	1	1
2	3	4	3	3	3	2	2	3
2	1	4	4	3	4	2	4	1
2	2	2	2	2	1	3	3	3
1	1	1	1	2	1	1	2	4
1	4	3	3	3	3	2	3	2
1	1	1	1	4	1	4	4	1
2	3	1	1	4	1	4	1	3
2	2	3	1	2	1	3	3	4
3	4	4	4	4	2	4	4	2
2	3	2	1	4	3	3	4	4
3	3	2	3	2	3	3	2	3
3	3	2	2	3	3	3	3	3
1	4	4	4	1	4	1	1	4
1	2	1	1	3	1	4	4	2
3	4	4	2	4	1	2	3	4
2	2	3	3	2	3	3	3	2
3	3	2	3	3	2	2	3	2
1	3	1	1	4	1	4	3	2
1	1	1	1	4	1	4	4	4
1	1	2	2	2	1	4	2	3
1	3	2	2	2	3	2	2	2
4	4	4	4	4	4	4	4	4
1	2	1	1	1	1	1	1	1
3	3	1	2	3	3	2	2	3

2	3	3	3	3	3	3	3	4
2	2	4	3	2	3	2	2	4
1	1	4	1	4	2	4	4	4
4	4	4	4	4	4	4	4	4
2	3	3	2	3	2	3	3	3
2	3	3	1	4	1	4	2	3
1	1	4	2	3	3	3	4	2
1	4	1	1	4	1	4	4	1
1	3	1	1	4	1	4	3	3
4	4	4	4	1	4	1	1	2
2	3	2	4	2	4	2	2	4
2	3	2	1	3	2	3	3	3
4	3	3	2	4	4	4	4	3
4	3	3	4	4	3	3	2	4
3	3	3	3	4	2	4	3	3
1	2	2	3	3	3	3	4	3
2	3	2	2	4	3	3	2	3
1	1	1	1	4	1	4	4	4
2	1	2	2	3	1	4	3	3
4	4	4	4	4	4	4	4	4
3	3	2	2	3	2	3	3	2
4	4	4	4	4	4	4	4	4
4	4	4	4	4	4	4	3	4
1	2	1	1	4	3	3	1	1
3	1	1	2	2	2	2	2	3
3	1	1	3	1	3	1	1	1
3	2	2	2	2	2	2	3	3
1	2	2	2	4	2	3	1	3
2	2	1	2	2	3	3	3	2
1	4	1	1	4	1	4	1	4
4	4	2	1	4	4	4	4	4
2	1	3	1	1	1	3	3	4
3	3	3	3	3	3	3	3	3
1	3	2	2	4	4	2	1	3
2	4	1	1	4	1	4	4	1
3	2	1	3	1	2	4	4	2
1	1	1	1	1	1	1	1	1
1	1	1	1	1	1	1	2	1
1	1	1	1	4	1	4	4	4
1	1	2	2	2	2	2	2	2
3	4	3	3	4	3	4	4	3
1	2	3	4	1	3	4	3	2
2	1	1	2	3	3	3	3	4
3	3	3	2	2	3	2	2	3
1	1	1	1	4	1	4	4	1
2	4	4	1	4	2	4	3	2
1	3	4	4	1	4	3	1	4
3	1	4	3	3	2	3	2	4
2	3	4	2	4	2	4	4	3
4	4	4	4	3	2	3	2	4
1	1	1	1	4	1	4	4	3
1	3	4	3	4	4	4	4	4
1	1	3	4	3	2	4	3	3
2	3	1	1	4	1	4	3	2

1	3	1	2	4	2	4	3	2
3	2	2	3	2	3	3	3	3
4	3	4	3	4	4	4	4	4
3	3	3	3	3	3	3	3	3
1	1	1	1	1	1	4	3	4
3	2	1	1	1	1	4	4	2
2	3	2	2	4	1	2	3	2
3	3	2	1	3	1	4	3	3
4	2	4	4	2	4	2	1	4
4	4	4	2	1	1	2	1	4
4	4	2	1	4	2	4	3	4
2	1	2	2	1	3	3	4	3
2	2	2	2	3	2	3	3	2
3	4	1	1	2	1	4	4	3
2	3	2	2	2	2	3	3	3
1	3	3	1	4	1	4	2	4
1	2	2	1	3	1	1	2	3
2	1	3	2	1	2	3	3	3
4	4	4	4	4	4	4	4	4
4	3	2	2	3	1	4	2	1
2	4	1	1	4	1	4	4	1
3	3	2	2	4	1	4	3	4
2	2	3	4	3	4	3	2	3
1	4	1	1	3	2	4	2	2
1	1	1	1	1	4	3	1	3
2	2	2	2	4	2	3	2	2
2	2	3	3	3	3	4	3	4
4	4	4	4	3	4	4	4	4
3	1	2	3	2	2	2	1	4
2	4	4	3	3	1	4	2	3
1	3	1	2	3	1	3	2	3
1	4	1	1	4	1	4	3	3
2	3	2	1	3	1	4	4	1
4	4	2	3	4	4	1	2	4
3	4	2	2	4	2	2	4	3
2	2	3	2	3	2	3	2	3
1	1	2	1	1	1	4	4	2
3	4	3	2	1	2	3	2	3
2	3	3	2	3	1	1	3	3
3	4	4	3	4	3	4	4	3
4	4	4	4	4	4	4	4	4
3	3	3	3	3	3	3	3	3
4	3	1	3	4	3	4	4	4
2	2	2	1	3	1	3	2	3
1	3	3	3	3	3	3	2	3
2	3	3	3	3	2	3	3	3
4	4	4	4	4	4	4	4	4
1	1	1	1	1	1	4	4	4
3	4	1	4	4	3	3	4	3
4	4	2	1	4	1	4	2	2
4	4	3	4	4	1	4	4	3
2	2	3	1	3	1	3	3	3
4	4	4	1	4	1	4	4	4
1	4	2	2	3	2	4	1	3

4	4	3	2	4	2	4	3	3
3	4	3	3	4	4	4	4	4
4	4	4	4	4	4	4	4	1
3	2	3	2	2	2	3	2	3
2	1	4	3	4	2	4	4	3
4	4	4	4	4	4	4	4	4
4	4	4	4	4	4	4	4	4
4	3	1	3	4	3	3	3	3
1	1	2	1	2	1	4	2	3
2	2	3	3	3	3	3	2	3
1	4	2	3	4	1	4	4	4
4	4	4	4	4	3	4	4	4
4	3	1	3	4	3	3	3	3
3	3	2	1	3	1	3	2	2
1	4	1	4	4	1	4	3	3
4	4	4	3	4	2	4	3	3
3	3	3	3	3	3	3	3	3
3	3	3	3	3	3	3	3	3
2	4	4	3	3	3	3	3	4
3	3	3	3	3	3	3	2	4
4	4	4	3	2	2	3	4	4
4	3	1	4	2	4	3	2	4
1	1	2	1	4	1	4	1	3
1	1	1	1	1	1	1	4	1
3	2	3	2	3	1	4	4	3
4	2	4	2	3	2	3	4	4
2	2	2	2	2	2	2	2	2
2	4	1	2	4	4	4	4	2
4	4	4	4	4	4	4	4	4
2	1	2	1	3	1	4	3	4
2	2	3	2	2	2	2	2	2
1	1	4	3	1	4	4	1	4
3	2	3	3	3	3	3	3	3
4	4	4	4	4	4	4	4	2
1	3	3	1	4	1	3	2	4
2	3	3	2	3	2	3	4	3
1	2	3	1	3	1	4	2	2
4	4	1	1	4	1	3	4	4
4	4	4	4	4	4	4	4	4
1	4	2	4	4	1	3	2	3
4	4	3	4	4	4	4	4	4
2	3	3	3	4	2	2	2	1
4	3	4	4	2	2	3	2	4
4	4	4	4	4	1	4	4	1
4	3	3	4	1	4	2	1	4
1	2	1	2	4	2	3	3	3
2	3	2	4	3	2	3	2	2
2	2	3	1	2	1	3	2	4
4	4	4	3	4	4	4	4	4
2	2	3	3	3	2	3	3	2
2	2	3	3	3	2	3	3	2
4	3	1	1	4	1	4	4	1
2	4	3	1	3	1	3	3	3
2	2	2	3	2	2	3	2	3

1	4	3	3	4	2	4	2	3
2	2	2	2	3	2	3	2	2
3	4	1	1	4	1	4	4	4
4	4	3	2	3	3	3	2	3
3	3	2	4	4	4	4	4	4
2	2	2	2	2	2	3	3	3
3	3	3	3	3	3	3	3	3
2	4	2	2	2	2	2	4	2
1	1	2	4	3	2	3	1	4
2	3	3	2	2	2	3	3	2
2	2	3	1	3	1	4	4	4
4	4	4	3	4	4	4	4	4
2	3	3	4	4	4	4	4	4
1	1	2	1	2	1	2	2	3
4	3	2	1	3	1	4	4	3
3	3	3	3	3	3	3	3	3
4	4	3	3	4	4	3	4	3
1	1	1	1	1	1	4	1	2
1	1	1	1	1	1	4	4	3
2	3	3	4	3	4	3	2	3
4	4	3	4	4	2	4	3	3
1	2	4	3	3	3	3	1	3
1	1	1	2	3	3	4	4	3
4	2	3	3	3	3	3	3	3
3	3	3	3	3	3	3	3	3
2	4	2	1	4	1	4	4	3
4	2	2	2	3	3	2	2	2
2	3	2	3	2	3	2	2	2
3	4	4	1	4	1	4	3	2
1	3	2	1	4	2	4	3	3
3	1	2	1	1	4	1	1	3
1	2	1	1	2	1	4	3	3
1	1	3	1	1	1	1	1	1
2	4	4	3	3	3	3	3	4
3	3	2	2	3	2	3	2	3
1	4	3	1	4	3	3	3	3
4	4	4	1	4	2	4	4	4
2	3	2	1	4	1	3	4	3
2	3	2	2	3	3	3	3	3
3	4	3	3	3	3	3	3	3
2	2	3	3	3	2	2	2	4
2	4	3	1	4	3	3	4	2
1	3	2	1	3	1	4	4	3
3	3	3	3	3	3	3	3	3
3	3	3	2	4	3	3	3	3
2	2	3	2	2	3	2	2	3
1	3	3	2	2	2	3	3	2
2	3	1	1	1	1	3	1	1
1	2	2	1	3	2	3	2	2
1	3	3	3	2	3	3	2	3
2	2	3	1	4	2	2	2	3
1	1	1	1	4	1	4	4	2
1	2	2	2	3	2	4	2	3
2	2	4	4	2	2	2	2	4

2	3	3	2	4	1	4	4	4
2	2	3	2	2	1	3	2	1
1	3	1	4	3	4	3	4	4
4	4	4	4	4	4	4	3	4
4	4	4	4	4	4	4	4	4
3	3	2	3	3	3	3	3	3
2	3	3	1	3	1	3	4	3
4	2	4	2	2	3	2	3	3
2	2	2	2	3	2	3	2	3
4	4	4	4	4	4	4	4	4
2	4	2	1	3	1	4	4	3
2	2	2	2	3	2	3	2	2
4	4	1	1	3	2	3	3	3
2	3	3	2	3	2	3	3	3
2	2	3	2	2	2	4	3	3
4	4	4	4	4	4	4	4	4
4	4	4	4	4	4	4	4	4
3	1	1	1	3	2	4	3	3
2	3	3	3	3	3	3	2	3
3	3	2	2	2	3	2	2	3
2	3	3	1	2	1	4	3	3
3	4	2	3	4	3	2	3	4
2	3	2	1	4	1	4	4	3
1	2	1	1	3	2	3	3	4
2	1	2	1	3	1	3	2	4
4	4	1	1	4	1	4	4	3
3	3	3	3	3	3	3	3	3
4	4	3	1	3	1	3	3	4
1	1	2	3	2	3	3	2	3
3	2	2	2	3	2	3	2	3
2	2	1	1	4	3	3	1	4
2	2	3	2	2	2	2	2	2
3	3	3	3	3	2	3	3	3
3	3	4	3	1	4	3	2	4
3	2	2	1	1	1	4	3	3
1	1	3	3	3	1	2	2	2
3	2	4	4	2	4	3	4	4
3	2	3	3	3	3	3	1	4
2	2	2	2	3	2	3	2	2
4	4	4	4	1	4	1	1	4
2	4	1	1	4	2	4	2	3
4	4	2	2	4	4	4	4	4
2	3	4	4	3	3	2	2	3
2	2	2	1	3	1	4	3	3
2	4	3	4	2	4	2	1	4
3	3	3	2	2	2	4	4	3
4	4	4	1	4	1	3	1	4
3	3	3	3	3	3	3	3	3
3	3	2	1	3	1	3	2	2
3	3	3	3	3	3	3	3	3
4	1	1	1	4	1	4	4	4
1	3	3	3	2	3	3	3	3
3	3	3	2	2	3	3	2	3
2	3	2	2	2	2	3	3	3

3	2	2	2	3	2	3	3	1
4	4	4	4	4	1	4	4	2
3	4	3	3	3	3	3	3	3
2	3	1	3	3	2	2	2	3
2	2	2	2	3	2	3	2	3
1	1	1	1	4	1	4	1	4
2	1	1	1	1	1	4	3	3
3	2	3	1	4	2	2	2	4
3	3	1	1	4	1	4	3	1
1	3	3	1	3	1	3	2	4
2	3	2	2	3	2	3	2	3
3	2	3	4	3	4	2	3	4
2	3	3	2	3	2	3	1	2
2	3	2	2	3	2	4	2	3
2	2	2	1	4	1	3	3	1
4	4	4	3	4	4	4	4	4
2	3	1	1	2	1	4	2	3
4	4	3	3	3	3	2	2	2
1	4	4	4	4	1	4	4	4
4	4	4	4	4	4	4	4	4
4	3	2	3	2	2	2	2	2
1	2	3	1	4	1	4	2	2
3	3	3	3	2	3	3	2	3
2	2	2	1	3	2	4	4	2
1	1	1	2	1	2	2	2	4
2	2	2	2	2	1	2	2	2
2	3	1	3	4	1	3	3	3
4	4	4	3	4	4	2	4	4
1	2	1	2	1	1	4	4	2
4	4	4	4	4	4	4	2	4
3	2	1	1	4	1	3	4	3
3	3	3	2	3	2	3	3	2
3	2	3	2	3	2	3	3	3
1	1	3	1	1	2	3	1	3
2	3	1	2	3	2	2	2	3
1	1	1	1	4	2	3	1	4
4	4	4	4	4	4	3	3	4
2	1	2	3	2	3	3	2	3
1	1	2	1	4	1	4	1	4
3	4	4	3	4	3	3	3	4
4	2	4	1	4	1	4	4	3
2	4	3	2	4	2	2	3	2
1	1	1	3	2	3	4	3	4
3	3	2	2	2	2	3	3	3
3	3	3	3	3	3	3	3	3
2	3	3	2	4	2	3	4	4
1	2	1	1	3	1	3	3	1
2	3	3	4	3	2	4	4	4
4	4	4	4	4	4	4	4	4
2	3	1	2	3	2	3	3	4
2	2	3	2	3	3	3	3	4
4	4	1	2	3	1	4	4	4
1	3	1	1	4	3	3	1	3
3	4	1	1	3	1	4	3	2

3	4	1	1	4	2	3	4	1
1	4	1	3	4	4	3	1	1
4	4	4	4	4	4	4	4	4
2	1	2	2	1	3	4	2	3
1	1	4	3	1	2	3	1	4
1	2	1	1	3	1	3	2	1
2	3	2	1	4	1	3	4	1
4	4	1	2	3	1	4	4	4
4	4	1	1	4	1	4	4	2
4	4	4	4	4	4	4	4	4
2	3	3	2	4	1	4	2	4
3	2	1	1	4	2	3	3	1
2	2	1	2	2	2	4	4	1
1	1	3	1	1	1	4	3	3
1	3	1	1	4	1	4	2	3
1	3	1	1	4	1	4	4	1
4	4	4	4	4	4	4	3	3
2	4	3	2	3	1	3	2	3
3	3	2	3	4	2	3	3	1
4	4	4	1	4	2	4	4	3
3	4	3	4	4	4	4	2	3
3	3	1	1	4	1	4	3	3
3	3	3	3	4	2	4	3	3
4	4	2	1	3	1	3	2	3
3	4	3	2	4	2	4	3	4
4	4	1	4	4	4	4	3	3
2	2	1	1	3	1	3	3	3
1	2	2	2	3	2	3	3	3
4	4	4	4	4	4	4	4	4
2	3	2	3	2	3	3	3	3
2	4	4	2	4	2	3	3	3
2	4	4	4	4	4	4	4	1
2	3	3	2	3	2	3	4	4
1	3	2	2	3	1	3	3	3
1	1	1	1	1	1	4	4	1
2	3	4	2	2	3	2	3	4
1	1	2	1	3	1	4	4	3
2	2	2	2	2	1	4	2	2
3	3	3	3	3	3	3	3	3
2	3	1	2	3	2	4	4	4
3	2	3	2	2	3	2	2	3
3	4	4	2	4	3	4	4	4
3	4	3	2	3	3	4	2	3
1	4	1	1	3	1	3	3	1
1	3	1	1	4	1	4	2	1
2	3	3	2	3	2	3	2	3
4	2	4	2	2	3	2	3	3
3	2	2	1	4	1	4	3	2
2	3	1	1	4	1	4	4	2
3	3	2	2	3	2	3	3	3
1	1	3	2	3	1	3	2	3
3	1	3	4	2	4	2	2	2
4	3	2	3	3	3	3	3	4
4	4	1	4	1	4	4	4	4

3	3	3	3	3	3	3	3	3
2	2	4	4	1	4	2	1	4
1	1	1	1	3	1	4	3	3
1	1	1	1	1	2	2	3	4
3	3	3	3	3	2	3	3	3
2	2	3	2	4	1	3	2	3
2	1	3	1	1	1	2	3	1
2	4	4	2	4	2	3	2	4
2	1	4	2	2	2	2	1	3
2	2	2	2	2	2	2	2	3
4	2	2	2	3	2	2	3	4
1	1	1	1	2	2	3	2	3
2	3	3	2	3	2	2	1	3
3	2	2	1	3	1	4	3	4
1	1	1	1	2	1	3	3	2
2	3	4	4	3	3	4	3	4
2	3	3	3	3	2	3	2	3
2	1	2	1	1	1	3	2	3
2	3	1	2	3	2	3	3	4
1	4	1	3	4	4	3	3	4
1	3	1	1	4	1	4	2	3
3	3	2	2	3	2	3	3	3
2	2	2	2	2	2	2	2	2
3	3	3	3	3	2	2	3	3
2	2	2	3	3	2	4	4	3
4	1	2	4	4	1	4	1	4
1	1	4	1	1	2	2	4	4
2	2	3	3	3	2	2	2	4
3	2	3	3	3	4	2	3	4
1	4	1	1	4	1	3	2	3
4	4	4	4	4	4	4	4	4
2	2	3	2	2	2	2	2	2
4	4	4	4	4	4	4	4	4
3	3	3	3	3	3	3	3	3
3	2	3	2	2	2	3	3	3
4	4	4	4	4	4	4	4	4
4	4	4	4	4	4	4	4	4
2	3	1	1	4	1	4	4	2
3	3	3	4	3	3	4	3	4
1	2	1	1	1	1	4	3	3
3	3	3	3	3	3	3	3	4
2	2	2	2	2	2	2	2	2
4	4	4	4	4	4	4	4	4
2	2	2	2	3	2	3	3	2
3	3	3	2	2	2	4	3	4
2	2	3	1	3	2	3	3	4
3	3	3	3	3	3	3	3	3
2	3	3	2	2	1	3	3	3
3	2	3	2	2	1	4	3	3
4	4	4	4	4	4	4	4	4
2	2	2	2	2	2	2	2	2
2	2	2	2	2	1	3	3	3
2	2	3	2	2	2	3	3	3
2	2	2	2	2	2	2	2	2

1	1	1	1	1	1	2	1	1
4	4	4	2	4	1	4	4	4
2	1	2	1	3	1	3	2	2
2	3	3	3	3	3	3	3	3
2	4	4	3	3	2	3	4	3
1	1	1	1	1	2	4	3	2
4	4	4	2	4	1	4	2	4
3	2	1	1	3	1	3	3	2
1	1	3	2	3	3	2	2	4
3	2	3	4	2	3	3	4	4
2	3	2	1	2	1	4	4	3
2	2	2	2	2	3	2	2	2
3	3	1	1	2	2	3	3	3
3	3	2	2	3	2	3	3	3
3	3	2	3	3	3	3	3	3
2	3	3	4	3	4	3	3	3
3	3	2	3	2	3	3	2	3
3	2	1	2	3	1	1	4	3
2	3	3	2	3	2	3	2	3
1	1	1	1	1	1	3	3	3
3	2	3	2	3	2	3	3	3
2	2	2	2	2	2	2	3	1
3	4	3	3	3	3	3	3	3
3	3	3	3	2	4	2	3	3
2	4	3	2	3	2	4	2	4
2	4	2	3	4	2	3	2	3
2	2	3	3	3	3	3	2	3
2	2	3	3	3	3	3	2	4
4	4	2	4	3	4	4	4	4
2	4	3	4	4	4	2	2	4
1	2	3	2	2	1	3	2	2
2	1	1	2	2	2	3	4	2
4	4	4	4	4	4	4	4	4
1	3	2	2	4	2	3	3	4
2	3	1	2	4	2	3	4	2
3	3	2	2	3	2	3	3	3
2	2	1	2	1	1	2	1	3
2	2	2	2	2	2	2	2	2
3	2	1	1	3	1	4	3	1
3	3	3	2	3	2	3	2	3
4	4	4	4	4	4	4	4	4
2	1	2	1	1	1	4	1	4
2	1	2	1	1	1	3	3	3
2	2	2	2	4	2	3	2	2
3	3	1	1	4	1	4	4	1
3	3	4	3	2	4	2	1	4
3	2	4	3	1	4	1	4	4
3	3	3	3	3	3	3	3	3
4	4	4	4	1	4	1	1	2
3	3	3	4	4	3	3	4	2
3	4	3	3	3	3	2	2	4
1	1	3	1	3	1	3	3	2
4	4	4	4	4	4	4	4	4
2	2	3	1	4	3	4	4	3

1	1	4	1	1	1	4	4	4
3	3	3	3	3	3	3	3	3
1	2	2	2	2	2	4	1	4
3	3	2	2	3	2	3	3	3
4	4	4	3	4	4	4	2	4
2	4	1	1	4	1	4	4	3
1	1	2	2	1	2	1	2	3
3	2	3	3	3	3	3	3	3
2	3	4	4	4	4	4	4	4
1	1	1	1	4	1	4	1	4
3	2	3	2	3	3	2	2	2
3	2	3	3	3	3	3	3	3
2	2	2	3	3	3	2	2	3
4	4	4	4	4	4	4	4	4
4	3	4	3	3	3	4	2	3
1	4	3	2	2	2	1	4	3
2	4	1	2	3	2	4	1	4
4	4	4	4	4	4	4	4	4
4	4	4	4	4	4	3	4	1
2	2	1	1	4	1	4	4	1
4	1	1	1	1	3	2	3	2
3	3	2	2	4	1	4	1	3
4	3	1	1	4	1	4	4	3
3	3	3	3	3	3	3	3	3
4	4	3	2	4	1	4	4	2
2	4	2	1	4	1	4	4	4
2	4	2	1	4	1	4	2	3
1	2	3	2	3	3	4	3	3
2	2	2	3	3	3	3	2	3
2	2	1	1	4	1	4	4	1
2	3	1	1	3	1	4	4	3
4	4	4	4	4	4	4	4	4
1	4	1	1	4	1	4	4	1
2	4	4	1	4	2	4	3	4
2	1	1	2	2	2	4	4	3
2	2	1	1	1	1	3	3	3
3	3	3	4	4	3	3	3	3
4	4	1	1	4	1	4	4	1
1	4	4	1	4	1	4	1	4
2	3	3	3	3	4	4	2	3
2	2	2	2	3	2	3	3	2
1	1	1	1	4	1	4	4	3
1	4	4	1	4	1	4	4	1
1	3	2	1	3	1	4	4	3
1	1	1	1	1	1	1	1	2
4	4	4	4	4	4	4	4	4
1	1	4	1	1	1	4	4	4
1	3	3	1	4	1	4	2	4
3	3	3	3	3	3	2	2	3
3	3	1	1	4	1	4	3	1
3	2	3	2	3	2	3	2	3
4	4	4	4	4	4	4	4	4
2	2	3	1	3	1	3	4	3
1	1	4	4	4	4	3	1	4

1	1	1	1	4	1	3	4	3
1	3	2	3	3	3	3	2	1
1	4	1	1	3	1	4	3	3
4	2	3	3	3	3	3	2	2
4	4	4	4	4	4	4	4	4
1	4	1	1	4	1	4	4	1
2	3	4	3	4	3	3	3	3
4	4	1	4	4	4	1	1	4
1	4	2	2	4	1	4	4	4
4	4	4	4	4	4	1	4	4
4	1	1	1	1	1	4	4	3
2	2	3	3	4	2	3	4	4
4	1	1	1	1	1	4	4	3
4	4	4	4	4	4	4	3	3
4	2	4	3	3	1	3	2	4
4	4	1	1	4	1	4	3	3
4	2	4	3	3	1	3	2	4
1	4	4	2	4	1	4	3	1
2	3	1	1	3	1	3	4	1
2	3	2	2	4	2	3	2	3
1	1	1	1	1	1	3	4	1
3	3	3	3	3	3	3	3	3
4	4	4	4	4	4	4	4	4
4	4	1	4	4	1	4	1	1
2	2	2	2	2	2	2	2	2
3	3	3	3	3	3	3	3	3
2	2	1	1	3	2	4	4	2
2	3	3	2	3	2	3	3	3
4	4	4	3	4	4	2	4	4
4	2	3	4	1	4	3	2	3
2	2	2	2	2	2	3	3	3
2	2	2	2	2	2	3	3	3
1	3	4	1	4	1	4	4	4
3	3	2	2	3	2	3	3	2
3	3	3	3	4	2	4	3	3
2	2	3	2	3	3	3	2	3
1	3	3	2	2	1	3	3	3
2	1	1	1	2	1	2	2	2
3	3	1	2	3	2	3	2	3
2	3	3	3	3	3	3	3	3
4	4	3	4	4	2	4	4	4
1	4	3	3	4	1	3	3	2
1	4	2	1	4	1	4	2	2
2	3	2	2	3	2	2	3	3
3	3	4	4	4	4	4	4	4
4	4	1	1	1	1	4	4	4
4	4	2	3	3	3	4	3	2
1	3	1	1	3	1	3	2	2
1	1	1	1	3	2	3	3	1
2	2	1	2	2	2	3	3	2
2	2	3	2	3	1	3	2	2
1	1	1	1	1	1	3	4	3
3	3	3	3	3	3	3	3	3
2	3	3	3	3	3	3	3	3

3	4	2	1	4	1	4	4	3
3	3	4	3	2	3	2	1	3
2	2	4	3	3	4	3	3	3
3	2	3	2	3	1	3	3	3
4	4	1	1	1	1	1	1	4
1	1	1	1	3	3	4	2	1
1	4	4	1	4	1	4	3	2
2	3	3	4	2	3	1	1	3
3	3	3	3	3	3	3	3	3
2	2	2	2	4	2	4	2	1
4	4	4	4	4	4	1	4	4
4	4	4	4	4	4	4	4	4
2	2	3	2	3	2	2	3	3
3	2	3	2	3	2	3	4	3
1	4	4	1	4	1	4	4	1
4	1	4	1	4	4	4	2	4
2	3	3	2	3	2	3	2	3
3	3	3	3	2	4	3	2	4
2	2	3	2	3	2	3	2	2
1	2	3	1	3	1	3	3	3
4	4	4	4	4	4	4	4	4
3	3	3	3	3	3	3	3	3
2	2	2	2	2	2	3	3	2
1	3	1	1	4	1	4	4	1
3	3	3	3	3	2	4	3	3
4	4	2	4	4	4	4	2	3
2	4	1	1	4	1	4	4	1
4	4	3	4	4	4	2	2	4
3	3	4	4	4	3	4	4	2
2	2	2	3	1	1	3	1	1
1	3	2	1	3	1	3	4	3
2	3	3	2	3	3	3	3	3
3	3	3	3	3	3	3	3	3
2	2	2	3	3	3	3	2	2
1	1	4	4	1	4	4	1	4
1	3	1	1	4	1	4	4	1
2	2	3	3	3	2	3	3	3
2	3	1	2	1	2	4	3	2
1	2	2	3	3	3	3	3	4
2	2	4	2	3	2	2	2	2
3	3	3	3	3	3	3	3	3
2	3	3	2	3	2	3	2	3
4	4	4	4	4	4	4	4	4
1	3	2	1	3	1	4	4	3
3	3	1	2	3	3	3	3	1
3	3	2	1	4	1	3	1	1
3	4	3	3	3	3	3	3	2
3	3	3	3	3	3	3	3	3
1	3	3	3	2	3	3	1	4
4	4	4	3	4	3	4	3	3
2	3	3	1	3	1	4	4	3
2	3	1	1	4	1	4	3	1
1	1	2	1	2	1	4	2	1
3	3	1	1	3	1	3	2	2

3	2	3	1	2	1	3	2	3
3	3	3	3	3	2	3	2	2
1	2	1	2	3	1	3	2	2
2	3	1	1	3	1	3	3	1
2	3	3	3	3	3	2	2	3
3	2	3	2	3	2	3	3	4
1	1	1	1	1	1	1	2	1
4	4	4	4	4	4	4	4	4
1	2	3	1	4	1	4	4	4
3	2	2	3	2	2	3	3	3
3	2	2	3	2	2	3	3	3
4	3	4	4	3	3	4	4	4
3	1	4	2	1	1	4	2	3
1	1	1	1	3	1	3	3	3
1	4	3	1	4	1	4	4	4
1	1	1	1	4	1	4	1	1
2	1	3	1	3	1	3	4	2
3	1	3	2	1	1	4	4	2
1	3	4	3	3	4	4	4	4
1	2	3	1	1	2	3	2	4
2	3	3	2	3	1	4	4	3
4	3	2	4	3	4	4	3	3
1	1	1	1	1	1	1	4	1
3	4	2	2	4	1	4	4	1
2	3	2	1	4	1	4	1	3
1	2	2	1	2	1	4	4	3
2	2	2	2	3	2	3	3	3
1	1	3	2	1	1	1	2	3
3	3	3	3	3	3	3	3	3
4	4	1	1	4	1	4	4	1
4	2	1	1	3	1	1	1	3
1	3	4	1	4	1	3	3	2
4	4	3	2	4	2	2	1	4
2	1	3	1	1	1	3	4	3
4	4	1	3	3	4	4	4	1
1	4	1	1	3	1	3	4	2
2	2	2	3	2	3	3	2	3
2	3	1	4	4	3	2	4	3
1	4	1	1	4	1	4	4	4
2	3	2	1	3	1	4	3	3
1	3	1	1	2	1	3	3	2
2	3	3	2	3	2	3	2	3
4	4	1	1	4	1	4	4	1
2	2	2	2	3	2	3	3	3
2	3	2	1	3	1	2	3	3
1	3	2	2	3	1	3	3	4
1	1	1	1	3	2	3	3	1
2	4	3	3	4	3	3	3	2
2	3	1	1	3	1	3	3	3
1	3	2	1	2	1	1	4	3
1	1	1	1	4	1	4	4	1
1	1	1	1	4	1	4	1	1
2	2	1	2	2	1	4	4	3
4	2	2	2	1	1	3	3	2

1	3	4	3	2	3	1	2	4
2	4	3	3	4	3	4	4	3
1	1	3	1	4	1	4	4	1
3	3	1	2	3	2	3	2	3
2	2	3	2	4	2	3	2	3
2	2	3	3	2	3	3	2	4
1	3	3	1	2	1	2	1	3
1	4	1	1	4	1	4	3	3
2	2	1	2	3	2	3	3	3
3	3	3	2	3	1	4	4	4
1	2	3	1	3	2	4	2	3
4	2	2	1	3	1	4	4	3
1	3	2	3	4	1	3	3	2
3	4	3	1	4	1	4	4	1
3	4	3	1	4	1	4	4	1
3	4	2	2	2	1	3	2	1
3	3	3	3	3	3	3	3	3
1	1	1	1	3	2	3	2	2
2	3	3	2	3	1	4	3	1
1	4	3	3	3	1	4	4	4
3	3	3	4	4	4	3	4	4
3	2	3	3	2	3	3	3	3
3	1	1	1	1	1	1	1	1
3	3	3	3	3	3	3	3	3
2	3	3	2	3	2	3	2	3
2	2	3	2	3	2	3	2	3
2	2	3	2	2	2	2	2	3
1	3	1	1	4	1	4	1	1
2	2	2	2	2	2	2	2	2
1	2	2	1	2	1	3	3	2
2	2	2	2	2	2	2	2	3
1	1	2	1	3	2	3	3	3
1	1	1	1	2	1	4	4	3
1	3	1	1	3	1	3	3	2
3	3	2	2	2	2	3	3	2
2	3	3	2	3	2	3	3	3
3	2	3	3	3	3	3	3	3
3	3	1	1	4	1	4	4	1
2	2	2	3	3	4	2	2	4
3	2	2	2	2	1	4	4	4
3	4	2	3	4	2	4	4	1
2	4	1	1	4	1	3	4	3
3	3	3	3	2	3	2	3	3
1	2	2	1	3	2	3	3	3
2	2	2	2	4	2	3	2	4
4	1	4	4	1	4	4	4	1
2	4	1	1	4	1	3	4	3
2	2	3	2	4	2	3	3	3
3	3	3	2	3	2	3	3	2
3	1	1	1	3	1	4	3	2
2	3	2	1	4	2	3	2	2
3	3	2	2	3	3	2	1	3
4	2	1	1	1	1	4	4	3
3	1	2	2	2	2	3	2	3

1	2	1	1	4	1	4	3	1
3	3	3	2	3	2	3	3	3
3	3	2	2	3	2	3	2	3
4	4	4	4	4	4	3	4	3
2	2	2	2	3	1	3	4	3
3	1	2	2	2	2	3	2	3

A15	A16	A17	A18	A19	A20	A21	A22	A23
2	3	2	3	3	3	2	3	3
2	4	4	1	4	3	4	1	4
3	3	2	3	3	3	2	2	3
3	3	2	3	2	2	2	2	2
4	4	4	1	4	4	4	4	4
1	3	2	2	2	4	1	1	1
2	2	3	2	2	3	2	1	2
2	3	3	2	3	4	1	1	3
1	4	1	4	4	1	1	1	4
1	4	1	4	4	4	1	2	4
4	3	1	3	4	3	3	3	3
2	3	2	3	3	3	1	2	3
1	4	1	4	4	4	1	3	4
4	1	4	4	4	4	4	4	4
3	3	1	3	3	1	2	1	3
1	3	3	4	4	1	1	1	4
1	3	4	2	3	3	1	1	4
4	4	4	4	4	4	4	4	4
2	3	2	3	4	3	2	2	3
4	4	3	2	4	2	4	3	4
2	3	4	2	3	2	2	2	3
2	3	2	2	2	4	2	1	3
1	4	1	4	4	4	1	4	4
2	3	2	2	3	2	2	3	3
3	2	2	3	3	2	3	3	2
3	3	3	3	4	3	2	2	3
3	3	3	3	3	3	3	2	2
2	3	2	3	3	2	2	2	3
2	4	3	3	2	2	1	1	1
2	3	3	3	3	3	3	2	3
4	3	2	3	3	3	3	3	3
1	4	1	4	4	4	1	2	4
2	3	2	3	3	3	2	1	2
3	3	3	3	3	3	3	3	3
3	4	4	2	3	4	2	2	1
3	3	3	3	3	3	3	3	3
3	4	2	4	4	3	2	2	4
4	4	4	4	4	4	4	4	4
3	3	1	2	3	4	4	3	3
4	4	4	4	4	4	4	4	4
1	4	1	2	4	4	1	1	4
1	3	3	3	3	3	2	3	3
3	3	3	3	3	3	3	3	3
3	3	3	3	3	3	3	3	3
1	1	1	4	4	1	1	4	4
4	3	2	3	3	3	3	3	3
3	3	3	3	3	3	3	3	3
4	4	4	4	4	4	4	4	4
1	1	2	2	1	2	1	1	2
3	3	3	3	3	3	3	3	3
4	4	4	4	4	4	4	4	4
1	4	3	4	4	1	1	1	4
2	3	1	3	3	3	1	2	3

3	3	4	2	3	3	3	2	2
1	3	3	4	4	2	2	1	4
4	4	2	2	3	2	3	4	3
1	1	1	1	1	1	1	1	1
4	4	4	2	4	4	2	4	4
1	4	1	4	4	1	2	1	4
3	2	1	3	3	1	1	1	4
1	1	1	1	1	1	1	1	1
4	3	4	2	4	2	2	4	4
3	3	2	3	3	3	2	3	2
2	3	2	3	1	1	2	1	1
2	4	2	3	4	3	2	1	3
1	4	2	4	4	1	1	1	4
1	1	1	1	1	1	1	1	1
1	1	2	3	3	4	2	1	4
1	4	1	4	3	4	1	4	3
1	1	1	1	1	1	1	1	1
1	4	1	4	1	3	1	1	1
3	4	2	4	4	4	1	4	4
2	3	2	3	3	2	2	1	4
2	2	3	2	3	2	2	2	4
3	3	3	3	3	2	2	2	3
4	2	4	2	2	4	2	1	2
4	4	4	4	4	4	4	4	4
1	1	1	4	1	1	1	1	1
4	4	4	3	3	2	4	1	3
1	3	3	2	3	1	1	1	3
3	3	3	3	3	3	3	3	3
3	2	2	2	2	2	2	2	2
1	1	3	1	1	1	1	1	1
3	2	3	2	3	2	3	2	3
3	4	1	3	3	1	4	1	3
2	3	3	3	3	3	2	2	3
2	2	4	2	2	2	2	2	2
3	2	3	3	3	3	3	3	3
1	4	1	4	4	1	1	1	4
1	4	1	4	4	4	1	1	4
2	3	3	3	4	3	1	1	3
3	4	1	4	4	3	4	3	4
2	3	1	3	3	3	1	1	4
3	3	2	3	2	3	3	2	3
2	3	3	3	3	3	2	2	3
4	4	4	1	1	4	4	1	4
1	3	1	3	4	1	1	1	3
2	3	2	4	4	1	1	1	4
3	3	2	3	3	2	2	2	3
2	3	2	1	1	1	1	1	1
1	4	1	4	4	1	2	2	4
2	4	2	4	4	1	1	1	4
2	3	1	4	2	2	1	2	2
1	2	1	2	4	1	4	1	3
4	4	4	4	4	1	2	4	4
1	1	1	1	1	1	1	1	1
4	4	1	2	4	1	2	1	3

4	4	2	3	3	3	2	2	2
3	3	2	2	2	1	3	1	2
3	4	2	3	1	1	1	1	3
4	4	4	4	4	4	4	4	4
3	3	2	3	4	3	2	2	3
2	3	2	2	4	3	2	1	4
2	3	3	3	3	2	2	1	3
1	4	1	3	4	1	1	1	4
1	3	3	4	4	1	1	1	4
4	4	2	4	1	4	4	1	1
3	3	3	3	3	2	3	3	2
2	3	1	3	3	3	1	1	3
3	4	3	2	3	2	3	1	3
3	4	3	3	4	3	2	2	4
2	3	3	3	3	3	2	1	4
3	3	4	3	3	1	2	1	3
3	3	1	3	3	3	2	1	3
1	4	1	4	4	4	1	1	4
3	4	2	4	4	3	1	1	3
4	4	4	4	3	3	1	3	3
2	3	2	3	3	3	2	2	3
4	4	1	4	4	1	1	4	4
4	4	4	4	4	4	4	4	4
3	2	3	1	2	2	3	1	3
2	2	4	2	2	2	3	1	2
3	3	1	2	3	3	3	1	3
2	2	1	2	2	2	2	2	2
1	2	3	2	3	1	1	1	3
2	2	1	2	3	4	4	1	2
1	4	1	4	4	1	1	1	4
4	4	4	4	4	4	4	1	4
1	4	2	4	1	4	1	1	1
3	3	1	3	3	3	3	3	3
2	2	3	3	3	3	3	1	3
1	4	1	4	4	3	1	1	4
1	4	1	3	3	1	2	2	3
1	1	1	1	1	1	1	1	1
1	2	1	4	4	1	1	1	1
1	2	1	4	4	1	1	1	4
2	2	2	2	2	2	2	2	2
2	4	1	4	4	4	4	3	4
3	2	3	2	2	3	1	1	1
4	4	4	1	1	2	3	1	4
4	3	4	3	3	4	4	2	2
1	1	2	1	1	1	1	1	1
2	4	4	3	4	4	1	3	4
4	3	4	1	1	3	4	1	1
2	4	3	4	4	4	2	2	3
1	4	3	4	2	1	2	1	4
3	3	4	2	3	4	3	1	3
2	2	1	4	3	2	1	1	2
4	3	4	4	4	4	4	4	4
4	4	3	3	3	1	2	1	3
1	4	1	3	4	3	1	1	4

1	4	3	3	3	1	2	1	3
2	3	2	2	3	2	1	1	3
3	4	3	4	4	4	2	4	4
2	3	3	3	3	3	2	2	3
3	3	1	4	1	3	1	2	1
3	4	2	4	4	3	2	2	3
1	3	2	4	4	2	1	1	4
1	3	1	3	4	3	1	1	4
4	4	4	2	2	3	4	4	2
4	4	4	1	1	4	1	4	1
2	4	3	4	4	4	2	1	4
2	3	3	3	2	2	2	2	2
2	3	2	3	3	2	2	2	3
1	4	1	4	4	1	1	1	4
3	3	2	2	2	2	2	2	3
3	4	3	3	3	1	1	1	4
2	2	2	2	3	3	1	2	4
2	3	2	2	3	2	1	3	3
4	4	4	4	4	4	4	4	4
1	1	1	3	1	2	2	1	1
1	4	1	4	4	4	1	1	4
1	3	4	3	4	3	2	2	4
2	4	2	2	2	3	1	3	4
1	1	2	4	3	1	1	1	3
1	1	4	4	2	2	2	2	2
2	2	1	1	3	2	2	1	2
2	3	2	2	3	2	2	1	3
4	4	4	4	4	4	4	4	4
2	2	4	2	2	3	2	1	1
1	3	1	4	4	4	1	4	4
2	3	2	2	2	2	2	2	2
1	4	1	3	4	1	1	1	4
1	3	1	4	4	1	1	1	3
2	4	4	3	4	1	3	1	4
2	3	3	4	4	4	1	2	4
2	3	3	3	3	2	2	2	3
1	3	1	4	1	1	1	1	1
2	3	2	3	3	3	3	1	3
2	3	2	3	3	2	2	2	3
3	4	2	4	4	4	2	3	3
4	4	4	4	4	4	4	4	4
3	3	3	3	3	3	3	3	3
4	4	2	4	4	3	4	2	4
1	3	2	2	3	2	1	1	3
3	2	2	2	3	3	2	2	3
1	3	3	3	3	2	2	1	3
4	4	4	4	4	4	4	4	4
1	4	1	4	1	1	1	1	1
2	4	1	4	4	3	4	3	4
1	4	1	4	4	2	2	1	4
1	4	3	4	4	3	2	4	3
1	3	2	1	3	1	1	1	3
1	4	4	4	4	4	1	4	4
2	4	3	3	3	2	3	1	3

3	3	3	3	3	3	3	3	3
4	4	1	4	4	4	1	1	4
3	4	1	4	4	1	1	1	4
2	3	2	3	3	2	2	2	2
4	4	2	4	4	2	2	1	4
4	4	4	4	4	4	4	4	4
4	4	4	4	4	4	4	4	4
3	4	3	4	4	1	1	2	4
2	4	1	4	4	1	3	1	3
3	3	4	3	3	1	3	1	3
4	4	3	4	4	4	2	1	4
4	4	4	4	4	4	4	4	4
3	4	3	4	4	1	1	2	4
4	2	1	3	3	1	1	1	3
3	3	4	4	4	4	1	1	4
3	4	4	4	3	1	3	4	4
3	3	3	3	3	3	3	3	3
3	3	3	3	3	3	3	3	3
2	4	3	2	3	2	3	3	3
4	2	3	3	2	2	3	3	2
3	3	3	3	3	3	3	3	3
4	2	1	4	2	4	4	4	4
2	4	3	4	4	1	1	1	4
3	2	1	1	1	4	2	1	1
3	4	2	4	4	4	2	2	3
2	2	4	4	3	4	2	3	3
2	2	2	2	2	2	2	2	2
4	4	2	4	4	4	3	4	4
4	4	4	4	4	4	4	4	4
1	4	2	3	4	3	1	1	3
2	2	2	2	2	2	2	2	2
4	4	4	4	1	1	4	1	1
3	3	1	3	3	3	3	3	1
4	4	4	4	4	4	4	4	4
2	2	3	3	4	2	1	1	4
2	4	3	3	4	3	1	2	4
2	4	1	4	4	1	1	2	3
2	3	2	4	4	4	1	2	4
4	4	4	4	4	4	4	4	4
4	1	1	4	4	1	1	1	4
3	4	1	4	4	4	3	4	4
3	4	3	4	4	3	3	3	4
4	3	2	4	2	2	3	2	3
1	4	1	4	4	4	1	1	4
4	1	3	2	3	4	4	3	3
2	3	1	2	3	1	1	1	4
2	3	1	3	3	2	2	2	3
3	3	2	4	4	2	2	2	3
4	4	4	3	4	3	4	4	4
3	3	1	3	4	2	3	2	3
3	3	1	3	4	2	3	2	3
4	4	2	2	4	4	1	1	4
1	3	1	3	3	3	1	1	3
2	2	3	3	3	2	2	2	2

1	4	2	4	4	1	1	1	4
2	3	2	3	2	1	1	1	3
2	4	1	2	4	1	1	1	4
3	3	2	3	3	3	2	3	3
4	4	4	4	4	4	4	4	4
2	3	3	3	3	3	2	2	2
3	3	3	3	3	3	3	3	3
3	3	1	1	4	2	3	2	3
4	2	2	2	4	1	2	1	2
3	3	1	2	3	2	3	2	2
1	4	2	2	3	1	1	1	3
4	4	4	3	4	3	4	4	4
4	4	1	4	4	3	4	1	4
1	1	1	2	2	1	1	1	1
1	2	2	4	4	4	1	2	4
3	3	3	3	3	3	3	3	3
4	4	4	4	4	4	4	1	4
1	1	1	4	4	2	1	1	3
2	3	1	4	1	1	1	1	1
3	2	2	2	3	2	3	2	3
3	3	1	3	3	2	4	3	3
2	2	1	2	1	1	2	1	2
1	4	3	4	4	1	3	1	4
2	3	3	2	2	2	3	2	3
3	3	3	3	3	3	3	3	3
1	4	3	3	4	1	1	1	4
2	2	3	2	2	2	3	3	2
2	2	3	3	3	4	3	3	3
1	4	4	1	4	4	2	3	3
1	4	1	4	4	1	2	1	4
1	3	3	3	1	1	1	1	1
2	4	1	4	3	1	1	1	2
1	3	4	1	1	1	1	1	1
2	4	3	2	3	2	3	3	3
2	3	2	3	3	2	2	1	3
3	3	3	3	3	3	3	3	3
2	4	1	4	4	4	1	1	4
2	3	2	4	4	3	1	2	4
3	3	1	2	3	3	3	3	3
3	3	2	3	3	2	3	3	3
3	3	2	3	3	3	2	3	3
2	3	3	4	4	3	2	2	4
1	3	1	3	3	2	1	1	2
3	3	3	3	3	3	3	3	3
3	3	2	3	3	3	3	3	3
2	3	4	2	2	3	2	2	2
2	3	3	3	3	3	3	3	3
1	3	1	3	1	3	1	1	1
2	2	2	2	3	2	1	1	3
3	2	3	2	3	4	3	3	3
2	2	3	3	3	2	1	2	3
1	4	2	2	4	1	1	1	3
4	3	3	1	3	2	1	1	2
3	2	4	3	2	3	3	2	2

2	4	3	3	4	2	1	2	4
2	3	1	4	2	2	2	2	2
4	4	3	2	4	2	4	3	4
4	3	3	4	4	3	4	3	4
4	4	2	4	4	4	4	4	4
3	3	3	2	3	3	3	2	3
1	3	1	3	3	2	1	1	4
4	2	1	3	2	2	3	4	2
2	2	3	3	3	2	1	1	3
4	4	4	4	4	4	4	4	4
3	4	3	2	4	2	2	1	3
2	2	2	3	3	3	3	2	3
2	3	2	3	3	3	3	3	3
2	3	3	3	3	3	2	2	3
3	4	2	3	3	3	2	2	2
4	4	4	4	4	4	4	4	4
4	4	4	4	4	4	4	4	4
1	2	2	3	3	2	1	1	3
3	3	3	3	3	3	3	3	3
3	2	3	3	3	3	2	3	2
1	3	3	2	4	2	1	1	4
4	3	3	3	3	3	3	3	3
1	4	1	4	4	3	1	1	4
1	3	2	2	3	1	1	1	3
2	3	2	3	3	3	2	2	3
2	4	3	4	4	4	2	3	4
3	3	3	3	3	3	3	3	3
1	3	4	2	4	4	3	1	4
4	3	2	1	2	3	3	2	3
2	2	2	2	3	2	2	2	3
3	3	1	2	3	1	1	1	3
2	2	2	2	2	2	2	2	2
2	3	3	3	3	2	2	3	3
3	2	2	3	1	3	4	3	2
1	3	1	2	2	1	2	1	1
1	1	1	1	3	4	1	1	3
4	2	4	2	3	4	4	2	3
2	3	2	2	3	2	1	1	3
2	3	2	3	3	3	2	2	3
4	4	4	4	4	4	4	4	4
1	3	3	2	4	4	2	2	4
4	4	2	4	4	4	4	2	4
3	2	4	3	3	3	3	2	3
2	3	1	2	3	3	1	1	3
4	4	3	2	2	4	4	1	1
3	3	2	4	3	3	2	2	3
1	3	4	3	4	1	1	3	4
3	3	3	3	3	3	3	3	3
1	3	1	2	2	2	2	2	2
3	3	3	3	3	3	3	3	3
1	1	1	4	4	1	1	4	4
3	3	3	3	3	3	3	3	3
3	3	3	2	2	3	2	3	2
3	3	3	3	3	3	2	2	2

2	3	1	2	3	2	1	1	3
1	3	1	4	4	4	1	4	4
3	3	2	3	3	2	3	3	3
1	1	3	3	3	2	1	1	3
2	2	2	2	3	2	2	2	3
1	4	1	4	4	4	1	4	4
1	3	2	3	2	1	1	1	3
2	3	2	3	4	3	2	1	4
1	3	1	3	4	4	1	1	4
2	3	3	3	4	1	1	1	4
2	3	2	3	3	3	3	3	3
4	3	4	3	3	2	4	2	3
2	3	3	3	3	2	2	3	3
1	3	1	2	3	2	2	1	4
1	3	1	2	2	4	1	1	3
2	4	3	3	4	4	1	1	4
1	3	2	3	3	2	1	2	4
1	2	3	2	3	4	3	2	3
4	4	1	4	4	4	4	4	4
4	4	3	4	4	4	4	4	4
2	2	2	3	3	3	2	2	2
1	2	1	1	4	1	1	1	4
3	3	2	3	3	3	2	2	2
2	4	2	4	4	2	2	2	4
4	2	4	4	4	4	3	1	1
1	1	2	2	1	2	1	1	2
2	4	3	3	3	2	1	1	4
4	4	2	4	4	4	4	1	4
1	2	4	4	4	4	1	2	2
4	3	4	4	4	3	3	2	4
3	3	2	3	3	3	1	1	3
3	3	2	2	2	2	3	3	3
2	3	4	4	3	3	2	3	2
1	2	1	2	2	1	1	1	3
3	3	1	3	3	3	2	1	3
4	4	1	1	4	1	1	1	4
4	4	3	3	4	4	4	4	2
3	3	2	3	4	2	3	3	3
2	2	2	2	4	2	1	1	1
3	3	4	3	4	3	3	3	4
1	4	1	3	4	4	1	2	4
2	2	2	3	4	2	2	2	3
2	2	2	1	3	1	3	1	4
2	3	1	3	3	3	1	2	3
3	3	3	3	3	3	3	3	3
2	3	3	3	4	3	2	2	4
1	3	1	3	3	1	2	1	3
4	4	3	4	4	4	4	3	3
4	4	4	4	4	4	4	4	4
1	3	3	3	4	1	1	1	3
3	3	3	3	3	3	3	3	3
2	4	2	3	2	1	1	1	4
1	3	1	1	4	3	1	1	4
1	3	1	3	4	3	2	2	4

4	3	2	4	4	2	1	1	4
4	3	1	4	4	1	3	1	4
2	4	2	3	3	3	3	3	3
2	2	2	3	3	2	2	1	3
4	1	4	2	1	1	3	1	1
1	3	1	3	4	1	1	1	4
1	3	2	3	4	3	1	1	3
2	4	2	3	2	1	1	1	4
2	4	1	4	4	4	2	4	3
4	4	4	4	4	4	4	4	4
1	3	2	4	4	4	1	1	4
1	3	1	4	2	1	1	4	4
4	3	1	4	2	1	1	1	2
1	1	1	1	3	2	2	1	1
4	3	2	4	4	1	2	1	4
1	4	1	4	4	3	1	1	4
3	3	3	3	3	3	3	3	3
3	4	1	3	3	3	3	2	3
2	3	4	2	4	1	1	2	4
3	3	2	4	4	4	1	2	4
4	4	3	3	3	3	4	4	4
3	3	1	3	3	1	2	1	3
2	3	3	3	3	3	2	1	4
1	3	1	3	3	1	1	1	3
2	3	2	3	4	4	2	2	4
4	4	1	4	4	4	4	1	4
1	3	1	4	3	1	1	3	3
2	3	2	2	3	2	2	1	3
4	4	4	4	4	4	4	4	4
3	3	2	2	3	3	3	2	3
3	3	2	3	4	3	2	1	4
1	4	1	4	4	4	4	1	4
2	2	4	2	3	3	2	2	4
1	3	2	3	3	2	1	1	3
1	4	1	4	1	2	1	4	1
3	3	4	3	3	3	3	2	3
1	4	1	4	3	3	1	1	3
3	2	3	3	3	3	3	2	2
3	3	3	3	3	3	3	3	3
2	4	3	3	3	2	2	2	4
2	2	4	2	3	3	2	2	2
4	4	2	2	4	4	4	3	4
2	3	1	3	4	2	2	2	4
1	3	1	3	3	2	1	3	3
3	4	1	4	4	1	1	1	4
2	3	1	3	3	2	2	2	3
4	2	1	3	2	2	3	4	2
2	3	2	4	4	4	1	1	4
1	4	1	4	4	1	1	1	4
3	3	3	3	3	3	3	2	3
1	3	3	1	3	3	3	3	3
2	4	1	4	2	2	3	1	3
4	4	2	3	3	4	4	4	4
4	4	2	1	4	4	4	4	4

3	3	3	3	3	3	3	3	3
2	2	3	2	2	3	2	3	3
1	3	1	2	3	2	1	1	3
3	2	3	2	3	2	3	2	3
2	3	3	3	3	3	2	2	3
2	3	2	3	3	2	1	1	3
1	3	1	1	3	3	3	1	3
2	3	4	2	4	4	2	1	3
2	2	4	3	2	3	2	2	2
2	2	3	2	2	3	2	2	2
2	2	4	3	3	3	2	1	3
1	3	2	3	1	1	1	1	1
3	3	3	3	2	2	2	2	2
1	4	3	3	3	3	1	1	3
2	2	2	2	4	2	2	2	2
3	3	4	2	3	3	3	2	2
3	3	4	2	2	3	3	3	2
2	3	1	3	3	1	1	1	2
1	3	3	3	4	1	1	1	3
3	3	2	3	4	4	4	2	4
4	3	2	4	4	1	2	1	4
3	3	3	2	3	3	3	1	3
2	2	2	2	2	2	2	2	2
3	2	4	3	3	4	2	2	3
3	3	1	4	3	3	2	1	3
4	4	4	1	4	4	1	1	4
2	3	3	2	3	1	2	1	1
3	3	2	3	3	3	2	3	3
4	2	4	2	3	4	4	4	3
1	2	2	3	4	3	3	1	4
4	4	4	4	4	4	4	4	4
2	2	2	2	2	2	2	2	2
4	4	2	4	4	4	4	4	4
3	3	3	3	3	3	3	3	3
2	3	2	3	2	2	2	2	2
4	4	4	4	4	4	4	4	4
4	4	4	4	4	4	4	4	4
1	3	1	3	3	4	1	1	4
3	3	3	3	4	4	3	3	3
1	3	3	3	1	1	1	1	1
2	4	4	4	4	4	4	3	3
2	2	3	3	3	3	3	3	3
1	4	3	4	3	1	3	3	3
2	3	2	3	3	2	2	2	3
3	3	3	3	3	3	3	3	3
2	3	4	3	3	3	3	1	3
3	3	2	3	3	3	3	3	3
2	3	2	2	2	3	2	2	3
2	3	2	3	2	2	2	2	2
4	4	4	4	4	4	4	4	4
2	2	2	2	2	2	2	2	2
1	2	2	3	2	1	1	2	2
2	3	3	3	3	3	2	1	3
2	2	2	2	2	2	2	2	2

1	1	1	1	1	1	1	1	1
2	4	4	4	4	4	4	4	4
1	2	1	2	3	2	1	1	4
2	3	2	3	3	4	4	2	3
1	4	2	3	4	3	2	1	2
1	1	1	1	1	1	1	1	1
4	4	1	4	4	2	1	1	4
3	3	2	4	3	1	1	1	3
1	3	2	2	3	2	2	1	3
4	4	4	3	2	1	3	3	2
2	3	2	3	3	3	2	2	2
2	2	2	2	2	2	2	2	2
1	3	2	3	3	2	2	2	3
3	3	2	3	3	3	3	3	3
3	3	3	3	3	3	3	3	3
3	3	4	2	3	2	3	3	3
2	2	3	2	3	3	3	2	2
2	2	2	2	2	2	2	2	3
3	3	3	3	3	3	2	2	3
1	3	1	2	3	2	1	1	4
3	3	1	3	3	3	2	2	3
2	4	3	2	2	2	2	2	2
3	3	3	3	3	3	3	3	3
4	3	4	3	3	3	3	3	3
2	3	3	3	2	4	4	1	1
2	3	2	2	4	2	2	2	4
3	3	3	3	3	3	3	2	3
3	3	3	2	3	2	1	1	2
4	4	3	3	4	4	4	4	4
4	4	4	3	4	4	4	2	4
1	3	1	2	3	2	1	1	3
3	4	2	3	2	2	1	2	2
4	4	4	4	4	4	4	4	4
3	3	1	2	3	3	2	2	4
1	3	1	3	4	3	4	2	4
3	3	2	3	3	3	3	3	3
2	1	3	2	2	3	2	3	2
2	2	2	2	2	2	2	2	2
1	3	1	3	2	2	2	1	3
2	3	2	3	3	2	2	2	3
4	4	4	4	4	4	4	4	4
1	1	1	1	1	1	1	1	1
2	3	2	3	1	2	1	1	2
2	2	1	1	3	2	2	1	2
1	4	1	4	4	4	1	2	4
3	3	4	1	1	1	4	1	1
4	4	2	4	2	3	4	4	2
3	3	3	3	3	3	3	3	3
4	4	2	4	1	4	4	1	1
4	2	4	3	3	2	4	2	3
3	3	4	3	3	3	3	3	3
2	3	3	2	3	1	1	1	3
4	4	4	4	4	4	4	4	4
2	4	1	4	4	2	2	2	4

1	1	1	4	4	1	2	4	1
3	3	3	3	3	3	3	3	3
2	3	2	1	2	2	1	1	2
2	3	2	3	3	3	2	3	3
4	4	4	4	4	4	4	4	4
1	4	1	4	4	1	1	4	3
1	2	2	1	1	1	1	1	1
3	3	3	3	3	3	3	3	3
4	4	1	3	4	4	4	4	4
1	4	1	4	4	1	1	1	4
3	2	3	2	3	3	2	3	2
3	3	3	3	3	3	3	3	3
2	3	3	2	3	2	3	2	3
4	4	4	4	4	4	4	4	4
4	3	4	3	4	4	4	4	3
2	3	2	4	3	2	2	2	3
1	4	1	4	2	1	1	1	2
4	4	1	4	4	4	4	4	4
1	2	2	2	2	2	3	1	2
1	2	1	4	2	1	1	1	2
4	1	1	2	3	2	3	3	1
2	3	1	3	4	3	1	2	4
2	4	2	3	4	3	2	3	3
3	3	3	3	3	3	3	3	3
2	4	1	4	4	3	1	3	4
1	4	1	4	4	1	1	2	4
1	4	3	4	4	1	1	1	4
1	3	3	3	2	1	3	1	2
3	4	2	2	3	2	2	2	3
1	2	1	4	2	1	1	1	2
1	4	3	2	3	3	2	1	4
4	4	3	4	4	3	1	1	4
1	4	1	1	4	4	1	4	4
1	4	2	4	4	3	2	3	4
4	3	3	3	3	2	3	1	4
2	2	1	4	1	1	1	1	1
3	3	2	4	4	4	4	1	4
1	4	1	4	4	4	1	1	4
4	4	1	4	4	1	1	4	4
3	3	3	3	3	1	1	1	3
2	3	1	3	3	3	2	2	3
1	4	1	4	4	4	1	1	4
1	1	1	4	4	4	1	1	4
2	4	2	4	3	2	1	1	3
1	1	1	1	1	1	1	1	1
4	4	4	4	4	4	4	4	4
1	1	1	4	4	1	2	4	1
2	4	2	4	4	1	1	2	4
2	2	3	3	3	3	3	3	3
1	3	1	3	4	4	1	1	4
2	3	2	3	2	3	2	3	2
4	4	4	4	4	4	4	4	4
1	3	2	3	3	2	1	3	4
4	3	2	4	4	2	2	1	4

1	3	2	2	3	1	1	1	4
1	4	1	3	3	2	3	2	3
1	3	1	4	4	1	1	1	4
4	4	3	3	3	3	3	2	2
4	4	2	4	4	4	4	4	4
1	4	1	4	4	4	1	4	4
4	3	4	4	4	3	4	3	3
1	4	1	1	4	4	4	4	4
2	4	2	4	4	2	3	1	4
4	4	4	4	4	4	4	4	4
1	4	1	4	2	4	1	1	1
2	2	4	4	4	2	3	1	4
1	4	1	4	2	4	1	1	1
4	4	4	4	4	4	4	4	4
1	3	4	3	3	3	2	3	3
4	4	3	4	4	1	1	3	3
1	3	4	3	3	3	2	3	3
1	3	1	4	4	1	2	1	4
1	3	2	3	3	1	1	1	3
2	3	3	3	2	2	2	2	3
1	3	1	3	1	1	1	1	1
3	3	3	3	3	3	3	3	3
4	4	4	4	4	4	4	4	4
1	4	1	4	4	4	4	4	4
2	2	2	2	2	2	2	2	2
3	3	3	3	3	3	3	3	3
2	1	3	2	3	3	2	2	4
2	3	2	3	3	2	2	2	3
4	4	2	4	4	4	4	1	4
2	3	2	3	2	2	3	1	1
2	2	2	3	3	2	2	2	2
2	3	3	3	3	3	2	2	2
1	4	3	4	4	3	1	1	4
3	3	2	3	3	3	2	3	3
2	3	3	3	3	3	2	1	4
3	3	3	3	3	3	3	3	3
2	2	1	2	2	2	2	2	3
1	3	2	2	1	2	2	2	2
2	3	2	3	3	3	2	1	3
3	3	3	3	3	3	3	3	3
2	4	2	4	4	4	3	3	4
2	3	1	3	4	1	1	1	4
1	3	3	4	4	1	1	1	3
2	2	2	2	2	2	2	2	2
4	4	4	4	4	4	4	4	4
1	4	1	4	4	1	1	4	1
3	4	2	3	3	2	3	2	3
2	3	2	3	3	2	2	2	3
2	3	1	3	3	1	1	1	3
2	4	2	2	3	2	3	2	2
1	3	1	3	3	2	2	1	3
1	3	1	3	1	3	1	1	1
3	3	3	3	3	3	3	3	3
2	3	2	3	3	3	2	3	2

3	4	3	4	4	3	1	2	4
3	2	3	3	2	2	4	2	2
3	4	3	3	3	3	4	2	3
2	3	1	3	3	3	1	1	3
1	4	1	4	1	4	4	1	1
4	4	1	4	4	1	2	1	4
1	2	3	3	4	1	2	2	4
2	1	1	3	3	3	3	3	2
2	3	3	3	3	3	2	2	3
2	2	1	2	4	1	1	1	3
4	4	4	4	4	4	4	4	4
4	4	4	4	4	4	4	4	4
2	3	2	2	3	2	2	2	3
3	3	3	2	2	2	1	3	3
1	4	1	4	4	1	1	1	4
2	4	4	4	4	4	4	1	4
1	3	2	3	3	1	1	1	3
4	3	3	3	3	3	4	2	2
2	3	3	3	3	3	3	2	2
1	3	1	3	3	3	1	1	3
4	4	4	4	4	4	4	4	4
3	3	4	3	3	3	3	3	3
2	3	2	3	3	2	2	2	3
1	4	1	4	4	2	1	1	4
4	2	4	2	4	2	3	2	4
2	3	3	2	4	3	3	3	3
1	4	1	4	4	4	2	1	4
4	4	4	4	4	4	4	4	4
4	2	2	4	4	4	3	4	4
2	3	1	2	2	2	2	3	1
1	3	3	2	3	1	1	1	3
3	3	2	3	3	2	2	2	3
3	3	3	3	3	3	3	3	3
2	2	2	2	2	2	2	2	2
3	1	2	1	3	4	4	1	3
1	4	1	4	4	2	1	1	4
3	3	2	3	3	3	3	2	3
3	4	1	4	2	3	2	1	1
2	3	3	3	3	2	2	1	3
2	2	4	2	2	2	2	2	2
3	3	3	3	3	3	3	3	3
2	3	3	3	4	1	2	1	4
4	4	4	4	4	4	4	4	4
1	3	1	3	3	2	1	1	2
2	3	2	3	3	3	3	2	3
2	3	2	3	3	2	2	1	3
2	3	2	3	3	2	3	2	3
3	3	2	3	3	3	2	3	3
1	3	2	1	3	1	1	3	3
4	4	2	4	4	4	4	4	4
1	4	1	4	4	2	1	1	4
1	4	1	3	4	4	1	1	4
3	3	1	2	1	1	1	1	1
1	3	1	3	3	2	2	1	3

2	3	2	3	2	2	2	1	2
2	3	3	3	3	3	3	3	2
1	2	2	2	2	2	2	2	2
1	3	1	3	3	1	1	1	3
3	3	4	3	3	3	3	3	3
2	3	3	2	2	3	2	2	3
1	2	1	4	4	1	1	1	1
4	4	4	4	4	4	4	4	4
2	3	3	3	3	3	2	2	3
3	3	2	3	2	3	2	3	3
3	3	2	3	2	3	2	3	3
3	3	3	4	4	3	3	4	3
2	3	2	2	3	1	2	2	3
1	3	2	3	3	3	1	1	3
3	4	2	4	4	3	3	3	4
1	4	1	4	4	1	1	1	4
1	3	1	3	3	4	1	1	3
2	4	1	3	1	1	1	1	1
3	3	3	3	4	3	3	2	4
2	3	4	3	3	2	1	1	2
1	4	2	2	4	4	1	3	4
2	4	1	3	3	3	4	2	3
1	4	1	4	4	1	1	1	1
1	4	1	4	4	2	1	1	4
2	4	1	3	4	2	3	2	3
2	3	3	1	2	1	1	1	2
2	3	2	3	2	2	2	2	2
1	4	3	1	1	1	1	1	1
3	3	3	3	3	3	3	3	3
1	4	1	4	4	1	1	4	4
2	3	1	3	4	4	1	1	4
1	3	1	3	4	4	1	2	4
2	1	3	2	2	2	2	2	4
2	4	1	4	3	4	1	1	3
2	4	1	3	4	4	1	3	4
1	2	1	2	3	3	1	3	3
3	3	3	2	2	2	2	2	3
2	4	1	4	4	3	2	1	3
1	4	1	4	4	4	4	4	4
1	3	2	3	3	2	2	2	3
1	2	1	1	3	1	1	1	2
2	3	2	3	2	3	2	3	2
1	4	1	4	4	1	1	1	4
3	3	2	3	2	2	1	1	3
1	2	1	2	3	2	1	1	3
1	3	2	4	4	1	2	1	3
1	3	1	1	4	1	1	1	2
3	3	1	3	4	2	2	4	4
2	3	1	3	3	2	1	1	3
2	3	1	3	3	3	2	1	2
1	4	1	4	4	1	1	1	4
1	4	1	4	4	1	1	1	3
2	3	1	2	3	1	2	1	3
2	3	2	4	3	2	2	3	3

3	4	2	3	2	2	1	1	1
1	4	1	4	4	4	3	1	4
1	4	2	3	4	1	1	1	4
2	3	2	3	3	1	1	1	3
2	3	2	4	4	1	1	1	4
2	2	2	2	2	3	3	3	2
1	2	2	2	2	2	1	1	2
1	4	3	4	4	4	1	4	4
2	3	1	3	2	3	1	1	3
2	4	2	3	3	3	2	1	4
2	3	1	3	4	1	1	1	3
1	3	2	3	3	3	1	4	4
1	3	2	3	4	2	1	1	3
1	4	1	4	4	1	1	2	4
1	4	1	4	4	1	1	2	4
1	1	3	1	2	2	1	3	2
3	3	2	2	3	2	2	2	3
2	2	1	2	2	2	1	1	1
1	3	1	2	2	3	1	3	1
1	4	1	4	4	1	1	2	4
3	3	3	3	4	4	4	3	4
3	3	4	3	3	3	2	1	2
1	1	1	1	1	1	1	1	1
3	3	3	3	3	3	3	3	3
3	3	3	3	3	2	2	2	3
2	3	3	2	2	2	2	2	3
2	3	3	2	3	3	3	2	2
1	3	4	4	4	1	1	1	4
2	2	2	2	2	2	2	2	2
2	3	2	3	2	1	2	2	2
2	2	2	1	1	1	1	2	2
1	3	3	1	3	1	1	1	3
1	4	1	3	2	3	1	2	2
1	3	1	4	4	3	1	1	4
2	3	2	3	3	3	3	2	3
2	3	2	3	3	2	2	2	3
3	3	2	3	3	3	3	3	3
1	2	1	3	2	1	1	1	4
3	2	3	2	2	2	2	1	2
2	4	3	4	3	2	1	1	3
3	3	1	3	4	4	3	1	4
1	4	3	3	4	2	3	3	4
3	3	3	2	3	3	2	2	3
1	3	3	1	4	4	1	4	4
2	2	3	3	4	4	2	2	2
1	4	1	1	4	1	1	1	1
1	4	3	3	4	2	3	3	4
2	3	3	3	3	1	3	1	3
2	2	2	3	3	2	2	2	3
2	3	1	3	3	2	1	1	2
2	3	2	3	4	2	2	2	4
3	2	4	2	3	3	3	3	3
3	3	1	4	3	4	1	1	4
2	2	2	2	2	2	2	2	2

1	4	1	4	4	2	1	1	4
2	3	2	3	4	2	1	1	4
2	3	2	2	3	3	2	3	3
3	3	4	3	4	4	2	4	4
1	3	1	3	3	3	1	1	4
2	2	2	2	2	2	2	2	2

A24	A25	A26	A27	A28	A29	A30	Metacognit	B1
3	2	2	3	3	2	3	84	5
4	1	3	4	4	4	4	101	5
2	2	2	3	3	2	2	77	5
3	2	2	2	3	3	3	73	5
1	4	1	4	4	1	1	96	5
2	1	1	2	1	2	3	64	5
2	1	3	3	4	3	2	77	3
3	1	3	4	4	3	4	80	2
4	1	1	1	4	2	4	78	5
1	3	1	3	4	1	4	81	5
2	3	2	3	3	2	3	88	5
4	2	1	3	4	3	4	81	4
3	4	1	4	4	1	4	92	5
4	4	4	4	4	4	4	77	5
3	2	2	4	3	2	4	79	5
3	1	3	1	4	3	4	74	5
1	3	1	3	3	2	3	119	3
4	4	4	4	4	4	4	74	5
2	2	2	2	3	1	3	94	5
3	3	2	3	3	4	4	71	5
3	2	2	2	3	3	2	78	3
3	2	2	3	3	3	3	80	5
1	1	1	1	4	1	4	69	5
1	1	1	2	3	2	3	84	5
3	3	2	3	3	3	4	89	5
3	2	1	2	3	2	3	83	5
3	2	2	4	2	2	3	75	5
3	2	2	3	3	2	3	71	2
4	2	3	2	1	4	3	85	5
2	2	2	2	3	3	3	94	5
3	3	3	3	4	3	3	79	5
1	1	1	2	3	1	3	62	5
2	1	1	1	2	1	3	90	5
3	3	3	3	3	3	3	80	4
4	2	2	3	2	3	2	97	5
3	3	3	2	2	3	3	91	5
1	3	1	4	3	3	4	120	5
4	4	4	4	4	4	4	120	5
3	3	2	3	3	3	3	82	4
4	4	4	4	4	4	4	90	5
1	1	1	1	4	1	4	90	5
3	3	3	3	3	3	3	60	5
3	3	3	3	3	3	3	103	2
3	3	3	3	3	3	3	80	4
1	1	1	1	1	1	1	120	2
3	3	3	3	3	3	3	46	4
3	3	3	3	3	3	3	94	5
4	4	4	4	4	4	4	117	5
2	2	2	1	1	1	1	74	5
3	3	3	3	3	3	3	75	3
4	4	1	4	4	4	4	81	5
1	2	1	1	4	3	4	100	5
3	2	2	3	3	2	3	33	5

4	4	3	4	3	3	4	102	5
2	2	2	2	4	2	3	73	5
1	2	2	2	2	4	4	56	5
1	1	1	1	1	1	1	30	5
4	4	2	2	4	4	4	106	5
1	1	1	1	4	1	2	81	5
1	1	1	3	4	1	1	65	5
1	1	1	1	1	1	1	83	5
4	4	4	4	4	4	4	74	5
3	2	2	1	3	2	2	37	5
2	2	2	3	1	2	3	74	5
2	2	2	3	3	2	3	79	5
2	1	2	1	4	2	4	30	5
2	1	1	1	1	1	1	66	5
3	3	1	3	3	3	3	86	5
1	4	1	3	3	1	4	76	5
1	1	1	1	1	1	1	80	5
2	2	1	1	3	1	1	87	5
1	4	1	4	4	1	4	75	5
2	1	2	3	3	2	3	112	5
2	2	2	4	4	2	3	46	5
3	2	2	3	2	3	4	76	5
3	1	1	4	2	2	2	65	5
4	2	4	4	4	4	4	88	5
1	1	1	2	1	1	3	61	5
1	1	1	1	3	1	4	39	5
3	1	2	1	3	2	3	76	5
3	3	3	3	3	3	3	76	5
2	2	1	3	2	2	2	75	5
1	1	1	1	1	1	1	69	5
2	2	2	3	3	2	3	85	5
1	1	1	3	1	1	4	62	5
2	3	2	2	3	2	4	73	5
2	2	2	3	4	2	3	77	5
2	3	3	3	3	3	3	102	2
1	1	1	1	1	1	4	78	5
1	2	1	2	4	1	4	82	5
3	3	1	3	4	2	3	78	5
4	3	4	3	3	4	3	90	5
1	4	1	1	3	1	4	64	5
2	3	3	3	2	3	3	80	5
2	2	3	2	2	3	2	78	5
4	4	4	4	1	4	4	58	5
2	1	1	1	2	1	4	72	5
2	4	1	1	4	1	3	74	5
2	2	2	2	2	2	3	62	5
1	1	1	2	1	2	1	64	5
1	2	1	1	4	1	4	109	5
4	1	1	1	4	1	4	39	5
2	2	1	2	2	2	1	72	5
1	2	2	2	3	3	3	89	5
4	4	4	4	4	4	4	77	5
1	1	1	1	1	1	1	75	5
1	1	1	2	3	1	3	111	5

3	3	3	3	3	3	3	83	3
3	1	3	3	2	3	3	82	4
4	1	3	1	1	3	3	75	5
4	1	1	1	4	4	4	72	5
3	2	2	2	3	2	3	75	1
3	1	2	2	3	2	4	92	5
2	1	1	2	3	3	3	84	5
1	1	1	2	4	1	4	68	5
3	1	1	3	4	1	4	91	5
1	4	4	4	4	2	4	92	4
2	3	1	2	3	2	4	79	5
3	3	1	1	4	2	2	79	5
4	3	2	3	3	3	3	72	5
3	1	2	1	4	3	2	69	5
1	1	1	1	4	1	3	77	5
3	1	1	3	2	3	3	106	5
3	1	1	1	1	1	3	75	5
1	4	1	4	1	1	1	105	5
4	3	1	1	1	1	3	119	5
3	3	3	3	3	3	3	69	5
2	2	2	2	3	2	3	68	5
4	1	1	4	4	4	4	61	5
4	4	4	4	4	4	4	65	3
2	1	1	1	2	3	3	66	5
1	4	3	1	2	2	2	70	5
1	1	1	1	4	1	2	78	5
2	2	2	2	2	2	2	97	5
2	1	1	3	2	2	3	56	5
2	2	1	2	2	2	2	86	5
1	1	1	4	4	1	4	74	5
4	4	1	1	1	1	4	72	5
1	2	1	1	1	1	1	70	5
3	3	1	3	3	3	3	30	5
3	3	2	3	4	2	2	41	5
2	1	1	1	4	1	4	66	5
1	1	1	3	3	1	4	60	5
1	1	1	1	1	1	1	103	5
1	1	1	1	1	1	1	71	1
1	1	2	1	1	1	4	82	5
2	2	2	2	2	2	3	81	3
3	3	3	4	4	3	4	44	5
3	4	1	2	1	1	2	89	5
3	4	1	3	3	2	4	85	5
2	2	2	2	3	2	3	93	4
1	1	1	1	1	1	1	88	5
3	2	2	3	4	2	4	91	5
4	3	3	1	3	4	4	65	5
4	3	4	3	2	4	3	105	1
4	3	2	4	4	4	2	85	5
4	3	1	4	4	3	2	73	5
3	2	1	1	2	2	4	69	5
2	3	1	4	4	4	3	70	3
4	4	4	3	2	4	3	109	5
2	1	1	3	3	1	4	85	4

1	2	1	1	3	1	3	68	5
3	1	2	1	3	1	3	76	5
4	4	2	3	4	3	4	69	5
3	2	3	3	3	3	3	71	5
2	2	1	2	3	2	3	93	5
3	2	2	2	4	2	1	75	1
2	1	2	1	3	1	3	86	5
3	1	1	1	4	1	3	63	5
4	2	4	3	2	4	3	74	4
2	1	1	1	2	3	2	72	4
1	2	1	1	4	1	3	66	5
2	1	1	1	1	2	2	76	3
2	2	2	2	3	2	3	59	5
2	1	1	1	4	1	4	70	5
2	2	1	2	2	1	2	115	4
3	1	1	1	3	3	4	60	4
2	1	1	1	1	1	3	77	5
3	2	2	2	3	3	2	85	4
4	4	4	4	3	1	4	84	5
1	1	1	4	1	1	2	57	5
1	4	1	1	4	1	4	61	5
3	2	1	3	4	1	4	67	5
3	4	3	4	3	2	3	79	5
2	1	1	1	3	1	2	115	1
1	2	1	1	1	4	4	62	2
2	3	2	2	2	3	2	91	5
3	2	2	2	2	2	3	65	3
4	4	3	3	4	4	4	72	5
2	1	1	1	3	2	1	66	5
2	3	4	1	1	4	4	84	5
2	2	2	3	3	3	2	81	5
1	1	1	1	4	1	4	76	5
2	1	1	1	3	1	3	53	5
1	3	1	2	4	1	3	85	5
1	1	2	2	4	2	1	72	5
3	2	2	2	3	2	2	101	5
2	1	1	1	1	3	2	120	5
3	4	2	4	4	2	4	90	5
2	2	2	1	3	2	3	98	5
3	3	3	3	3	3	4	66	5
4	4	4	4	4	4	4	73	5
3	3	3	3	3	3	3	73	5
4	4	2	2	3	2	4	109	2
2	1	1	2	4	2	3	60	5
3	2	2	2	2	3	3	98	5
3	1	1	2	2	2	2	80	5
2	4	1	4	4	4	4	88	5
1	4	1	1	4	1	1	63	5
3	3	3	3	3	3	4	97	5
3	1	2	4	4	2	3	82	5
2	3	1	2	3	3	4	96	1
3	1	2	1	3	2	1	104	5
4	4	1	4	4	1	4	83	5
2	1	2	4	3	2	4	71	4

3	3	3	3	3	3	3	85	5
2	4	4	3	3	4	4	120	5
1	1	1	1	1	1	1	120	5
2	2	2	3	2	3	3	83	5
3	1	3	3	3	2	2	76	5
4	4	4	4	4	4	4	82	5
4	4	4	4	4	4	4	96	5
2	2	2	4	4	3	1	114	5
3	1	2	3	3	2	3	83	5
3	3	3	3	3	3	3	70	5
4	3	2	1	4	4	4	79	5
4	4	1	4	4	4	4	96	5
2	2	2	4	4	3	1	92	5
3	4	3	1	3	1	4	86	5
1	1	1	1	2	1	4	85	5
2	2	1	2	3	3	4	84	5
3	3	3	3	3	3	3	91	5
3	3	3	3	3	3	3	94	3
3	1	4	3	3	3	2	74	5
2	2	3	3	3	3	3	50	5
2	3	2	4	3	2	3	86	5
2	4	3	3	2	3	3	94	5
2	1	2	2	3	2	4	64	5
1	4	1	1	1	1	1	94	5
3	1	1	2	2	1	4	120	3
4	3	4	2	3	4	3	72	5
2	2	2	2	2	4	4	61	1
1	1	1	4	4	1	4	87	5
4	4	4	4	4	4	4	83	5
2	2	1	3	2	3	4	118	5
2	2	2	2	2	2	2	70	4
4	4	4	4	1	4	4	91	3
3	3	1	3	3	3	2	69	5
4	4	4	4	4	4	4	88	5
1	1	1	1	3	3	3	120	5
3	4	3	4	4	2	2	79	5
1	1	1	1	4	1	4	105	5
4	1	1	4	4	1	4	90	1
4	4	4	4	4	4	4	86	4
2	4	2	2	4	2	4	65	5
1	4	2	4	4	4	4	96	5
3	2	3	3	4	3	3	93	5
3	3	2	3	3	3	3	69	5
4	1	1	4	4	4	4	73	5
4	1	4	4	2	4	3	79	5
1	2	1	1	2	2	4	107	5
2	1	1	1	3	1	3	79	5
2	2	2	2	3	2	3	79	5
4	2	2	3	4	2	3	76	5
3	2	3	2	3	2	3	69	3
3	2	3	2	3	2	3	69	3
1	1	1	4	4	1	4	80	5
2	1	1	4	3	1	3	72	5
2	2	2	3	3	2	2	68	5

2	1	2	3	4	2	4	85	5
2	2	2	2	3	3	4	110	5
1	1	1	4	1	1	3	74	5
2	2	2	3	4	2	3	90	5
4	4	4	4	4	4	4	70	5
2	3	2	2	2	2	3	69	5
3	3	3	3	3	3	3	70	4
2	2	1	1	1	1	2	67	5
4	1	1	1	2	3	4	107	5
2	1	2	2	3	2	3	108	5
2	1	1	1	3	1	3	49	5
4	2	2	3	4	2	3	84	5
4	4	4	4	4	4	4	91	5
2	1	1	1	2	3	4	106	5
1	4	2	1	4	1	4	44	5
3	3	3	3	3	3	3	44	5
3	3	2	4	4	4	4	84	5
1	2	1	1	1	1	1	96	5
1	1	1	1	1	1	1	72	5
3	2	3	4	3	3	3	88	4
2	2	3	2	4	3	4	81	3
3	2	2	3	3	3	4	90	5
4	3	2	4	3	3	4	78	5
3	2	2	3	3	2	2	69	5
3	3	3	3	3	3	3	79	5
2	2	1	1	4	1	4	88	1
3	2	3	2	2	1	2	73	5
3	3	2	3	3	3	3	62	5
4	2	4	4	3	1	4	63	5
1	1	1	1	4	1	2	44	5
3	1	3	1	1	2	3	85	5
3	2	2	1	2	2	3	74	5
1	1	1	1	1	1	1	80	5
3	1	4	3	3	3	2	85	5
2	2	2	3	3	2	3	74	5
3	3	3	3	3	3	3	80	5
2	1	1	1	1	2	4	88	5
2	1	1	1	4	1	4	83	3
3	3	1	3	2	1	3	92	5
3	3	2	3	3	2	3	63	3
3	3	2	3	3	3	3	90	5
3	3	2	2	4	2	4	89	4
1	3	1	1	3	1	3	75	5
3	3	3	3	3	3	3	81	5
3	3	3	3	3	3	3	51	4
3	3	2	2	2	3	3	54	5
3	3	3	3	3	3	3	84	5
1	3	1	1	1	1	3	72	5
2	2	1	1	3	1	1	61	2
4	4	3	2	3	3	3	69	5
2	2	2	2	4	2	3	84	4
1	1	1	2	1	1	2	88	5
2	1	1	2	3	1	1	66	5
4	4	3	3	2	4	2	94	3

3	2	2	2	4	3	3	108	5
2	2	2	2	2	2	3	118	5
3	3	2	3	3	4	4	87	5
3	1	3	3	4	4	4	72	5
4	4	4	4	4	4	4	81	5
3	3	3	3	2	3	3	70	5
2	1	1	3	4	1	4	120	5
1	2	2	4	3	3	2	80	5
3	3	1	2	3	1	2	74	5
4	4	4	4	4	4	4	88	5
3	1	1	3	4	2	3	82	3
2	2	1	3	3	2	3	78	2
3	3	3	3	3	3	3	120	5
3	2	3	3	3	3	3	120	5
3	2	1	3	2	3	3	61	5
4	4	4	4	4	4	4	90	5
4	4	4	4	4	4	4	80	5
2	1	1	1	1	1	2	71	2
3	3	3	3	3	3	3	93	5
3	3	2	3	3	2	3	80	4
3	1	1	2	2	2	3	66	5
3	3	3	3	3	3	3	67	3
3	3	1	1	4	2	3	92	5
3	1	1	1	3	2	3	86	3
3	2	2	2	3	2	2	71	5
3	1	1	3	4	1	4	71	5
3	3	4	4	3	3	3	65	3
3	1	1	4	3	3	3	65	4
2	2	1	3	2	1	3	91	5
3	2	2	3	2	2	2	81	5
1	1	1	1	3	2	3	63	5
2	2	2	2	2	2	2	60	4
2	3	2	4	4	3	4	96	3
3	2	1	3	2	1	4	81	5
2	2	3	2	2	2	2	74	5
1	3	1	3	3	1	1	102	1
4	4	2	3	2	3	2	77	5
3	3	3	3	3	3	3	100	4
2	2	2	4	3	2	3	86	5
4	4	1	4	4	4	1	68	5
1	1	1	4	4	2	2	89	5
1	4	1	4	4	1	4	88	5
3	3	4	3	3	3	2	83	1
2	2	2	2	4	2	3	90	5
3	4	2	2	4	3	4	60	5
3	2	2	3	2	3	3	88	4
3	4	2	1	4	3	2	60	5
3	3	3	3	3	3	3	84	3
2	2	2	2	2	2	2	78	5
3	2	2	3	3	2	2	71	4
1	1	1	1	1	1	1	65	2
3	3	3	3	3	2	3	95	4
2	3	2	3	2	3	2	88	2
1	1	1	1	4	3	4	69	5

1	3	1	1	3	1	3	70	4
2	2	1	1	4	4	4	72	5
3	3	2	3	3	2	3	56	3
2	2	2	2	3	2	4	80	1
2	2	2	3	3	2	2	73	5
1	1	1	1	4	1	4	74	5
2	1	1	1	1	1	3	80	5
3	2	1	3	4	3	4	92	5
1	1	1	1	4	1	4	75	5
1	2	1	1	3	3	3	79	5
3	2	2	3	3	1	3	57	5
3	2	2	2	3	3	2	105	5
3	2	2	2	3	3	3	69	5
2	3	1	4	4	2	4	76	5
2	1	1	2	2	1	1	99	5
4	4	3	2	4	4	4	118	4
2	2	1	4	3	1	3	76	5
4	2	1	1	2	1	2	60	5
1	4	1	1	4	1	4	80	5
4	4	4	4	4	4	4	81	5
2	2	2	3	3	3	3	76	1
2	1	1	1	3	1	3	46	5
2	3	2	3	3	2	3	72	2
2	2	2	2	3	2	4	97	3
2	1	1	4	4	4	4	65	5
2	2	2	1	1	1	1	106	5
2	1	1	2	4	1	4	72	5
2	1	1	4	4	2	2	81	5
1	1	4	2	2	3	3	81	5
4	4	2	4	2	3	3	53	5
3	2	1	1	3	1	2	74	5
3	3	2	2	3	3	3	66	5
2	2	2	3	2	2	3	113	4
4	1	1	1	2	3	1	83	5
1	2	2	3	3	1	3	63	5
1	1	1	3	3	1	3	98	5
3	4	4	4	4	4	4	81	5
3	3	2	3	2	3	3	76	5
1	1	1	1	2	2	2	68	5
4	2	3	2	4	3	3	75	5
3	1	1	1	4	3	3	90	5
2	2	2	3	3	2	3	88	2
1	1	1	1	4	1	4	63	5
3	2	2	3	3	2	3	97	5
3	3	3	3	3	3	3	120	4
2	2	1	4	3	3	3	73	5
3	2	2	2	3	2	3	90	5
3	3	1	3	3	3	4	73	5
4	4	4	4	4	4	4	64	5
3	2	2	1	3	3	3	76	5
3	4	3	3	3	3	3	84	5
4	2	1	1	3	1	1	87	5
2	1	1	1	4	2	3	103	5
2	2	1	2	3	1	3	67	5

1	3	1	4	4	2	3	68	5
1	4	2	4	4	4	4	60	5
3	3	3	3	3	3	3	75	5
1	2	1	1	2	3	3	73	5
4	1	2	1	1	4	4	91	5
1	1	1	1	3	1	3	120	1
3	2	2	3	4	2	3	78	5
4	2	1	1	3	1	1	63	5
2	3	3	3	3	2	4	67	5
4	4	4	4	4	4	4	52	5
2	1	1	1	4	1	4	83	5
1	1	1	1	1	1	4	70	1
1	1	1	2	3	1	4	101	5
2	2	2	2	1	1	2	80	5
3	2	2	3	4	3	3	77	5
1	1	1	1	4	1	4	95	5
3	3	3	3	3	3	3	102	5
2	2	2	3	4	2	3	75	5
2	2	1	2	3	2	4	79	1
1	1	2	4	4	2	4	65	5
3	3	3	3	3	3	3	88	5
2	2	1	2	3	1	3	91	5
1	1	1	1	4	1	3	65	5
1	1	2	1	3	1	3	71	5
3	1	1	2	3	2	4	119	5
1	1	1	3	4	2	2	79	5
1	1	1	1	3	1	4	82	5
2	2	2	3	2	2	3	90	5
4	4	4	4	4	4	4	82	5
2	2	2	3	3	2	2	69	4
3	2	1	2	4	3	3	63	4
1	1	1	4	4	1	4	88	5
3	2	2	3	3	2	2	67	5
2	1	2	2	3	2	3	73	4
1	3	1	1	1	1	4	89	5
2	3	3	3	2	3	3	83	5
2	1	1	2	3	2	3	77	5
3	3	3	2	2	2	2	104	3
3	3	3	3	3	3	3	83	3
2	3	2	2	3	2	4	66	1
4	3	3	2	1	4	2	67	4
3	2	2	4	4	3	4	73	5
2	3	2	2	3	2	3	81	5
3	1	1	3	3	3	3	76	5
1	1	1	1	1	1	3	71	5
3	2	2	2	3	2	3	83	5
1	2	2	4	3	3	2	75	5
1	2	2	2	3	2	3	86	5
1	2	1	1	3	1	4	93	5
3	3	3	2	3	3	3	92	5
3	3	3	3	3	3	3	90	3
4	4	4	4	4	4	4	71	5
3	3	2	2	3	2	4	58	5
2	1	1	1	2	2	4	67	5

3	3	3	3	3	3	3	85	5
2	2	3	2	2	2	2	70	4
2	1	1	1	2	1	3	55	5
2	3	2	3	2	3	2	86	3
3	2	3	3	3	3	3	67	5
2	2	1	1	3	3	3	69	3
1	1	1	1	2	1	2	77	2
4	2	3	1	3	4	3	51	5
2	2	1	1	1	1	2	74	5
2	2	2	2	2	2	2	73	5
3	2	2	2	2	2	2	62	4
1	1	1	2	1	1	3	96	5
2	3	3	3	2	2	2	82	5
2	1	2	1	3	3	3	64	5
2	2	2	2	2	2	2	73	3
4	4	3	4	3	3	4	89	5
3	3	2	3	2	3	3	83	3
2	2	2	3	2	2	3	81	5
3	2	2	1	3	3	3	60	5
2	2	2	2	2	2	4	85	5
3	2	2	3	4	3	3	82	5
3	1	1	3	3	3	3	83	5
2	2	2	2	2	2	2	64	5
3	2	3	3	3	3	3	83	4
2	3	1	2	2	3	4	87	5
1	1	2	1	4	1	4	82	5
4	1	2	1	1	2	3	120	5
3	3	2	3	3	3	3	65	5
1	2	2	3	3	2	3	111	5
3	2	3	4	4	3	4	85	3
4	4	4	4	4	4	4	77	1
2	2	2	2	2	2	2	120	4
3	3	3	3	3	3	3	120	5
3	3	3	3	3	3	3	76	4
3	3	2	3	3	3	3	101	5
4	4	4	4	4	4	4	50	5
4	4	4	4	4	4	4	100	5
2	2	3	3	2	2	2	74	5
3	3	3	4	4	4	3	107	5
1	1	1	1	1	1	4	73	5
3	3	3	3	3	3	3	87	5
3	3	3	3	3	3	3	81	5
3	3	4	4	4	4	4	88	5
2	2	2	2	2	2	3	76	5
3	3	3	3	3	3	3	73	5
1	2	2	3	4	2	3	120	5
3	2	2	3	3	3	3	61	5
3	2	2	3	3	3	3	68	5
2	2	2	3	2	2	3	71	5
4	4	4	4	4	4	4	61	5
2	2	2	2	2	2	2	37	4
3	3	2	3	3	3	3	104	5
2	2	2	2	2	2	3	58	5
2	2	3	2	2	2	2	86	5

1	1	1	2	1	1	1	83	5
4	4	4	4	4	4	4	37	1
1	1	1	2	2	1	3	94	5
2	3	2	3	3	2	3	66	5
3	2	3	1	3	2	2	69	3
1	1	1	1	1	1	1	91	5
4	1	2	4	4	3	4	76	5
1	1	1	3	3	1	4	60	5
3	1	3	1	2	3	3	70	5
3	4	1	2	2	3	3	83	5
3	2	2	3	2	3	3	85	1
2	2	2	2	2	2	2	91	4
2	3	2	3	2	2	3	80	5
2	3	2	3	3	2	3	64	5
3	3	3	2	3	1	3	82	5
3	3	3	3	3	2	3	55	1
2	3	2	3	3	3	3	83	5
2	2	1	2	3	3	3	68	5
3	2	2	3	3	3	3	92	5
2	3	1	1	1	1	2	90	5
3	3	2	3	3	2	4	83	5
2	2	2	2	3	3	3	80	5
3	3	3	3	3	3	3	83	5
3	3	2	3	3	3	3	76	5
2	3	2	4	2	2	3	111	5
2	2	2	2	4	2	3	107	5
3	2	2	3	3	3	3	61	5
1	3	1	2	2	3	3	67	5
4	4	2	4	4	4	4	120	5
4	4	2	4	4	4	4	78	5
2	2	1	2	2	2	2	74	1
3	2	1	1	3	1	2	83	4
4	4	4	4	4	4	4	58	5
3	3	1	1	3	2	3	61	5
1	2	2	1	4	1	3	63	5
2	3	2	3	3	2	3	76	5
1	1	1	1	3	1	3	120	5
2	2	2	2	2	2	2	41	5
1	3	1	1	3	1	2	63	5
2	2	2	3	3	2	3	67	5
4	4	4	4	4	4	4	81	5
1	1	1	1	1	1	1	79	5
2	2	1	3	2	2	4	100	4
2	3	2	2	2	3	2	88	5
1	3	1	3	4	1	4	92	5
4	2	1	4	3	1	4	88	5
4	4	4	4	1	4	4	93	5
3	2	3	3	2	3	3	71	5
1	4	4	4	4	2	4	120	4
3	3	3	2	3	2	2	79	5
3	1	4	3	3	4	4	64	5
3	3	3	3	2	3	1	91	4
4	4	4	4	4	4	4	62	5
2	1	1	1	4	1	4	78	4

1	1	1	1	1	1	4	111	5
3	3	3	3	3	3	3	78	5
3	2	3	1	3	2	2	56	5
2	3	1	3	3	2	3	88	4
4	1	4	4	4	4	3	110	5
3	4	1	1	4	1	4	66	5
3	2	1	3	4	3	2	74	5
3	3	3	3	3	3	3	88	5
4	4	4	4	4	4	4	74	5
1	1	1	1	4	1	4	120	5
2	2	2	2	2	2	3	105	5
3	3	3	3	3	3	3	81	5
2	2	3	3	3	3	3	62	5
4	4	4	4	4	4	4	116	5
3	4	4	4	3	4	4	86	5
2	2	1	2	3	3	3	51	5
1	1	1	1	1	2	4	63	5
4	4	4	3	4	4	4	76	1
2	3	2	3	2	3	2	83	5
1	1	1	1	2	1	1	90	5
2	1	1	1	1	1	1	84	3
2	3	1	4	4	2	2	78	5
3	3	1	2	3	1	4	86	5
3	4	4	2	4	2	3	70	5
1	2	1	1	4	1	3	79	5
3	1	1	2	4	2	2	51	5
4	2	1	3	4	3	4	69	5
3	2	3	1	2	1	3	104	5
2	3	2	2	3	2	3	75	4
1	1	1	1	2	1	1	92	5
1	1	1	1	3	1	1	83	2
3	1	2	3	4	3	4	49	5
4	1	1	1	4	1	4	96	5
3	3	3	4	4	3	3	75	5
2	2	3	2	3	3	4	79	3
1	1	1	1	1	1	1	84	5
4	4	1	2	3	4	4	77	5
1	1	1	1	4	1	4	71	5
4	4	1	2	3	3	3	75	5
3	3	2	3	2	2	2	72	5
2	2	2	2	3	2	3	33	5
1	1	1	4	4	1	4	120	1
1	4	1	1	4	1	4	64	5
2	2	2	1	3	2	2	75	5
1	1	1	1	1	1	1	77	5
4	4	4	4	4	4	4	83	5
1	1	1	1	1	1	4	73	5
2	1	1	1	2	1	1	72	4
3	3	1	1	3	3	3	120	5
1	1	1	1	4	1	4	71	5
3	2	3	2	3	2	3	86	5
4	4	4	4	4	4	4	63	5
2	1	2	2	2	1	2	71	5
4	2	1	4	4	2	3	67	5

2	1	1	1	3	1	3	89	1
3	1	1	2	2	3	3	118	5
1	1	1	1	4	1	3	72	5
2	2	2	3	3	2	4	95	5
4	4	4	4	4	4	4	90	5
1	1	1	1	1	1	1	87	5
3	3	3	3	3	2	3	117	5
1	1	4	1	4	4	4	60	5
4	2	1	4	4	1	4	92	5
4	4	4	4	4	4	4	60	1
1	4	1	4	1	1	1	111	5
4	3	2	2	3	4	4	77	5
1	4	1	4	1	1	1	84	5
3	3	3	3	3	3	4	77	5
2	2	1	3	3	1	3	74	5
3	1	1	3	3	2	4	61	5
2	2	1	3	3	1	3	72	5
4	1	1	1	4	2	4	43	5
1	1	1	1	3	1	4	90	5
2	2	2	2	2	1	4	120	5
1	1	1	1	1	1	1	96	5
3	3	3	3	3	3	3	60	5
4	4	4	4	4	4	4	90	5
4	4	1	4	4	4	4	69	5
2	2	2	2	2	2	2	76	2
3	3	3	3	3	3	3	97	4
3	1	1	2	3	1	2	67	2
2	2	2	2	3	2	3	74	5
2	1	1	4	4	2	2	69	5
3	1	1	3	1	3	3	74	5
2	2	2	2	2	2	2	80	5
2	3	2	2	2	2	3	80	5
3	1	1	1	4	3	3	79	5
2	3	2	3	3	2	3	86	5
1	1	1	1	4	1	3	84	5
3	3	3	3	3	3	3	65	5
3	1	1	2	2	3	3	54	5
2	1	1	3	2	1	1	84	5
2	2	2	2	3	2	3	75	5
3	3	3	3	3	3	3	89	4
1	4	2	2	4	2	4	101	5
1	2	1	1	3	1	3	71	5
3	1	1	2	3	2	4	76	5
2	2	2	2	2	2	2	65	5
4	4	4	4	4	4	4	115	5
1	4	1	4	4	1	4	75	5
1	2	3	3	3	2	4	89	5
2	2	2	2	3	2	3	65	5
2	2	2	2	3	2	3	64	1
2	2	2	1	3	2	2	70	4
3	2	2	2	3	2	3	71	5
1	1	1	3	3	1	1	50	5
3	3	3	3	3	3	3	90	3
3	4	2	3	2	3	3	81	4

2	3	2	3	4	2	4	92	5
3	2	2	2	2	3	3	78	5
3	2	3	3	3	3	4	92	5
2	2	1	3	3	2	3	74	5
1	1	4	4	1	1	4	69	3
3	1	1	4	4	4	4	82	5
3	2	3	2	4	3	3	86	4
3	2	2	3	3	3	2	76	5
3	2	3	3	3	3	3	85	5
1	3	1	1	1	1	2	61	5
4	4	4	4	4	4	4	117	1
4	4	4	4	4	4	4	120	1
1	2	1	2	3	2	2	68	5
2	2	2	3	2	2	2	77	5
1	1	1	1	4	1	1	72	5
4	3	3	4	4	4	3	102	5
2	3	2	2	3	2	3	68	5
3	3	3	2	3	3	3	89	4
3	2	3	2	2	3	3	77	5
2	1	1	1	2	1	3	62	5
4	4	4	4	4	4	4	120	5
3	3	3	3	3	3	3	91	5
2	2	2	2	2	3	2	70	5
1	1	1	1	4	1	4	70	5
2	4	4	2	3	2	3	89	4
3	3	3	3	4	3	3	92	5
1	1	1	1	3	1	4	76	3
4	2	3	4	4	4	4	109	5
4	4	3	4	4	4	4	108	5
4	3	2	2	2	2	2	68	5
3	1	2	1	3	2	3	65	5
3	2	2	3	3	3	3	80	3
3	3	3	3	3	3	3	90	5
2	2	2	2	2	2	2	64	1
1	4	4	4	4	4	4	85	4
1	1	1	1	4	1	4	70	5
3	2	2	3	3	2	3	79	5
1	1	1	3	3	1	4	74	5
2	2	1	2	3	2	3	76	5
4	3	2	2	2	2	2	70	5
3	3	3	3	3	3	3	88	5
3	1	2	2	4	3	4	78	5
4	4	4	4	4	4	4	120	5
1	3	1	1	3	1	3	63	5
1	2	1	1	3	1	3	73	5
1	1	1	2	3	2	3	67	5
2	3	2	3	4	2	3	84	5
1	4	1	4	3	3	4	87	4
2	4	1	4	3	3	3	73	5
2	3	3	3	4	3	4	107	5
1	1	1	1	4	1	4	74	5
4	2	1	1	3	1	4	71	5
1	1	1	1	1	1	1	48	5
2	2	1	2	2	1	3	65	5

2	2	1	3	3	3	3	70	4
2	2	3	3	2	2	2	80	5
2	2	2	2	2	2	2	60	5
1	4	1	1	3	1	3	63	5
3	3	3	3	3	3	3	91	5
2	2	2	2	2	2	3	74	5
1	1	1	1	1	1	1	41	1
4	4	4	4	4	4	4	120	5
3	3	2	2	2	2	4	78	4
2	3	2	2	3	2	3	79	5
2	3	2	2	3	2	3	79	5
4	3	2	3	4	4	2	104	4
3	2	2	4	1	3	3	72	5
2	2	2	2	3	2	3	66	5
4	2	3	1	4	3	4	90	5
1	1	1	1	4	1	1	54	5
1	1	1	1	2	1	3	59	5
2	2	1	1	1	1	4	62	5
4	2	3	3	2	4	4	97	5
1	1	1	1	1	1	3	66	5
3	2	2	4	3	3	3	83	5
3	2	1	2	3	2	3	90	5
1	1	1	1	4	1	1	51	5
1	2	1	2	4	1	4	78	5
4	1	1	4	3	3	4	82	5
2	2	2	2	2	2	3	66	5
1	2	2	2	4	3	3	67	5
3	1	1	1	1	1	4	57	5
3	3	3	3	3	3	3	91	5
1	4	1	1	4	1	4	78	1
3	3	2	4	3	1	2	73	5
3	1	1	1	4	1	3	73	5
2	2	2	2	2	3	4	73	4
1	2	1	2	3	1	3	70	4
1	1	1	3	3	1	4	88	5
3	2	1	1	4	3	3	66	5
3	2	2	2	2	2	3	73	3
1	2	1	2	3	1	4	78	5
4	4	4	4	4	4	4	94	5
2	1	2	2	3	2	3	69	5
1	1	1	1	2	1	2	51	5
3	1	2	3	3	2	2	73	5
1	1	1	1	4	1	4	78	5
2	3	2	2	2	2	2	70	5
1	1	1	1	3	1	2	59	5
2	1	2	3	3	2	4	76	4
1	1	1	1	3	1	4	57	5
2	3	2	3	4	3	2	90	5
1	1	1	3	3	1	2	64	5
2	1	1	2	3	1	3	63	5
1	1	1	1	1	1	4	58	1
1	1	1	1	1	1	3	59	5
3	2	2	3	2	3	2	70	5
2	1	1	2	3	1	4	69	5

2	3	2	3	2	3	4	76	5
3	2	1	2	4	2	4	89	5
1	3	1	1	3	1	4	69	5
1	2	1	2	3	2	3	67	5
2	2	2	3	4	2	4	81	5
2	2	2	2	2	2	3	72	4
2	1	1	1	1	2	1	52	5
4	2	1	4	4	3	1	86	5
2	2	2	1	3	3	3	67	5
3	1	2	1	3	3	3	83	5
2	1	1	1	3	2	4	65	4
2	1	2	2	4	2	4	82	3
3	2	2	3	4	3	3	75	5
2	1	1	1	4	1	4	75	5
2	1	1	1	4	1	4	75	5
1	1	3	2	1	3	2	58	5
2	3	2	2	2	2	3	82	5
1	3	1	1	1	1	2	50	4
2	3	1	2	2	3	4	67	4
2	1	1	3	4	3	4	79	5
4	2	2	4	4	3	3	104	5
3	3	3	2	3	3	2	83	2
1	1	1	1	1	1	1	36	5
3	3	3	3	3	3	3	88	5
3	2	2	3	3	2	3	81	5
3	2	2	2	3	3	3	74	3
2	3	3	3	2	3	2	75	3
1	1	1	1	3	1	3	66	5
2	2	2	2	2	2	2	61	2
3	2	1	2	2	2	2	62	5
2	2	2	2	2	2	2	91	5
2	1	1	1	3	2	3	56	5
3	4	1	1	1	2	3	62	5
1	1	1	1	4	1	3	63	3
3	3	2	2	2	3	3	65	3
3	3	2	2	3	3	3	78	4
3	2	3	3	4	3	3	79	5
1	1	1	1	4	1	2	89	1
1	1	2	1	1	1	3	65	5
2	2	1	4	2	2	2	66	5
1	1	1	1	3	1	3	72	5
2	4	1	3	4	3	3	82	5
3	3	2	3	3	3	2	89	5
1	2	1	3	3	2	4	82	5
2	2	1	1	4	1	2	75	5
1	1	1	1	4	1	1	72	5
2	4	1	3	4	3	3	68	5
3	2	2	3	3	3	3	89	5
2	2	2	2	3	2	3	81	5
1	3	2	2	2	2	3	74	5
3	2	1	2	3	2	3	63	5
2	3	2	3	3	2	3	76	5
1	3	1	4	3	1	4	79	5
2	2	2	2	2	2	2	78	5

1	2	1	1	3	1	4	66	5
3	3	3	2	3	2	3	64	5
2	3	2	3	3	2	2	80	5
4	4	4	4	4	4	4	76	1
2	2	1	1	3	1	3	111	5
2	2	2	2	2	2	2	67	5

B2	B3	B4	B5	B6	B7	B8	B9	B10
4	4	1	5	5	5	5	5	5
1	3	1	2	4	3	2	5	5
5	5	5	5	5	5	5	5	5
5	5	5	5	4	5	5	5	4
5	5	1	5	5	1	1	3	1
5	5	5	5	5	5	5	5	5
2	2	5	2	3	2	2	2	4
2	1	1	3	2	3	4	4	5
1	3	1	2	4	2	3	2	1
5	5	1	5	5	5	5	5	5
3	1	1	3	4	4	4	4	3
4	3	4	3	4	4	4	4	4
5	5	1	3	5	5	5	5	5
2	1	5	1	3	5	5	5	5
5	5	5	5	5	5	4	5	3
1	1	1	5	1	5	5	3	5
3	3	3	3	3	3	3	3	3
5	5	1	5	5	5	5	5	5
3	2	2	2	2	2	2	3	5
3	2	1	2	2	3	2	4	3
3	2	2	3	3	3	3	2	4
3	1	2	2	2	2	2	4	4
5	5	1	5	5	5	5	5	5
4	2	2	3	4	4	4	2	2
2	3	1	3	3	3	3	4	4
5	5	3	5	5	5	5	4	1
4	4	1	5	5	5	5	3	3
4	3	4	4	4	4	4	2	2
4	3	1	4	4	5	3	1	4
5	3	2	3	3	3	3	4	4
5	5	1	5	5	5	5	5	5
5	5	1	5	5	5	5	5	5
3	3	1	3	4	4	4	5	5
4	4	4	4	4	4	4	4	4
5	3	2	3	4	5	5	4	5
5	5	4	5	5	5	5	5	5
5	5	1	5	5	5	5	5	5
5	5	5	5	5	5	5	5	5
3	4	2	3	3	3	3	3	5
5	5	5	5	5	5	5	5	5
5	5	1	5	5	5	5	5	5
4	4	1	4	4	4	4	4	4
5	5	2	5	5	5	5	2	5
4	4	4	4	4	4	4	4	4
5	5	2	2	5	5	5	2	5
4	4	4	4	4	4	4	4	4
5	5	5	5	5	5	4	4	4
5	5	5	5	5	5	5	5	5
4	3	3	3	3	3	3	3	3
2	3	2	3	3	5	4	4	1
5	5	1	5	5	5	5	5	5
5	5	1	5	5	5	5	5	5
5	5	2	5	5	4	5	3	5

3	3	1	3	2	2	2	5	5
3	3	1	3	4	3	3	3	3
5	4	4	4	4	3	4	4	4
5	5	5	5	5	5	5	5	5
5	5	1	5	5	5	5	3	5
4	5	1	5	5	5	5	5	5
5	5	1	5	5	5	5	5	5
5	5	5	5	5	5	5	5	5
4	4	5	4	5	4	4	5	5
4	4	1	5	5	4	4	5	4
3	3	1	3	5	3	3	4	3
4	4	1	4	5	3	3	4	5
5	5	1	5	5	5	5	3	5
5	5	1	5	1	3	3	3	3
5	5	1	5	5	5	5	4	5
3	4	1	3	5	3	3	5	5
5	5	5	5	5	5	5	5	5
5	5	1	5	5	5	5	1	5
5	5	1	5	5	5	5	5	5
5	5	1	4	4	4	4	5	4
3	4	2	3	4	4	5	4	5
5	5	1	5	5	5	5	5	5
2	2	1	5	3	3	3	4	5
4	3	1	2	2	2	2	4	3
5	5	5	5	5	5	5	5	5
3	3	1	2	4	2	2	1	5
4	4	1	5	5	4	4	5	5
3	3	1	3	3	4	3	3	3
5	4	1	3	3	3	3	5	5
5	5	1	5	5	5	5	5	5
3	2	1	3	3	2	2	5	3
5	3	1	3	3	3	3	5	3
5	3	1	3	5	5	5	3	5
3	3	3	2	5	3	3	3	5
5	4	3	2	5	5	5	1	4
5	5	1	5	5	5	5	1	5
5	5	1	5	5	5	5	5	5
4	4	2	3	3	3	3	3	4
5	5	1	5	5	5	5	4	5
5	5	1	5	5	5	5	5	5
2	2	1	4	3	2	2	3	5
3	3	3	4	3	4	3	3	3
1	1	1	1	2	1	1	3	5
5	5	1	5	5	5	5	5	5
3	5	1	2	3	3	2	5	2
4	5	1	4	5	4	4	4	2
5	5	3	5	5	5	5	5	4
3	4	1	3	4	3	3	5	5
5	5	1	5	5	5	5	5	5
4	3	1	3	5	3	3	2	5
2	2	4	2	2	2	2	5	5
5	5	1	5	5	5	5	5	5
5	5	5	5	5	5	5	5	5
4	4	1	3	5	2	3	5	5

3	3	3	3	3	3	3	3	3
2	2	1	3	3	2	2	2	2
4	5	1	5	5	5	5	1	5
5	5	1	5	5	5	5	5	5
5	5	1	5	5	4	4	3	5
3	3	1	3	5	3	3	4	5
4	4	1	3	5	3	3	5	4
4	3	1	4	5	3	3	5	1
4	4	1	5	5	4	4	5	1
3	3	4	3	3	3	3	3	3
4	3	1	4	3	3	2	3	4
5	5	1	5	5	5	5	1	5
5	5	1	4	5	5	3	1	5
4	4	1	5	5	5	5	4	4
5	5	1	5	5	5	5	1	5
4	4	1	5	4	4	5	5	5
4	4	1	4	5	5	5	3	4
5	5	1	5	5	5	5	3	2
5	4	1	5	5	4	5	2	5
5	5	2	5	5	5	4	4	3
4	4	1	4	2	4	4	3	3
5	5	1	5	5	5	5	1	5
5	5	3	5	5	5	5	5	5
3	3	1	3	4	3	3	3	3
3	3	1	2	3	2	3	4	5
2	2	1	2	5	2	2	4	5
3	4	1	3	3	3	2	5	5
1	1	1	3	1	1	1	5	5
3	3	2	2	3	2	2	3	5
5	5	1	5	5	5	5	5	5
5	5	5	5	5	5	5	5	5
3	4	1	3	5	3	3	5	4
4	4	1	4	4	4	4	2	4
1	1	1	1	3	1	3	3	5
5	5	1	5	5	5	5	5	5
4	3	1	5	5	5	5	5	5
5	5	1	5	5	5	5	2	5
5	5	1	5	5	5	5	5	5
5	5	3	5	5	5	5	3	5
5	5	3	2	4	3	3	2	5
5	4	1	5	5	4	4	4	3
3	5	1	2	5	4	2	5	4
3	3	1	3	4	3	3	5	3
2	2	2	2	4	3	3	4	5
5	5	1	1	5	1	5	1	5
3	3	1	3	5	5	5	2	5
3	2	1	2	5	2	2	5	5
5	4	1	5	5	5	5	5	5
4	5	1	4	4	4	4	5	5
3	3	1	3	4	3	3	4	5
4	3	1	5	5	5	5	4	5
3	3	1	5	5	5	5	5	5
3	3	1	3	4	3	3	3	5
4	4	2	4	4	4	4	3	5

4	4	1	4	5	4	5	4	5
3	4	1	3	5	3	3	3	5
4	5	1	5	5	5	5	5	5
3	3	1	5	5	5	3	5	4
2	2	1	1	5	4	3	5	5
5	4	1	5	5	5	5	3	5
4	5	1	4	5	5	5	4	5
4	5	2	4	5	4	4	5	5
1	2	2	1	2	1	1	4	3
3	2	2	2	4	3	3	1	2
5	5	1	5	5	5	5	3	5
3	3	3	5	5	3	3	3	3
5	5	1	5	5	5	5	2	5
5	5	1	5	5	5	5	4	5
3	3	1	3	4	3	3	3	5
3	3	2	2	5	5	5	1	5
5	5	2	3	4	5	5	3	4
3	3	2	3	5	4	4	3	4
5	5	1	5	5	3	5	3	4
5	5	1	5	5	5	5	5	5
2	3	1	3	5	3	3	5	5
5	5	1	5	5	5	5	5	5
3	4	1	4	5	4	5	3	5
5	5	1	5	5	5	5	2	5
3	1	1	5	5	5	5	5	5
4	4	1	4	5	5	4	1	5
4	4	3	3	3	4	3	2	3
5	5	1	5	5	5	5	5	2
2	2	1	5	5	1	1	5	5
5	5	5	5	5	5	5	2	5
3	2	1	5	5	4	5	5	5
5	5	1	5	5	5	5	5	5
5	5	1	5	5	5	5	1	5
3	3	1	1	5	3	2	5	5
3	3	1	5	4	5	5	1	1
4	3	4	3	5	5	5	4	3
5	4	1	5	5	3	4	3	4
5	3	4	3	5	5	5	5	5
3	3	5	3	3	3	3	3	5
5	5	1	5	5	5	5	5	5
5	5	5	5	5	5	5	5	5
5	5	1	5	5	5	5	1	5
5	3	1	5	5	5	5	1	1
4	5	1	4	5	5	5	4	5
3	3	2	3	4	3	3	3	4
3	3	1	3	4	4	4	5	5
5	5	5	5	5	5	5	5	5
5	5	1	5	5	5	5	5	5
5	5	1	5	5	5	5	5	1
5	5	1	5	5	5	5	5	5
5	5	1	4	5	5	5	5	3
5	5	1	5	5	5	5	5	5
5	5	1	5	5	5	5	5	5
3	2	2	3	2	2	4	2	2

3	5	1	5	5	5	5	4	5
5	3	1	5	5	5	5	1	5
5	3	1	4	5	5	5	5	5
5	5	1	5	5	3	3	3	5
5	4	1	5	5	4	5	5	5
5	5	1	5	5	5	5	5	5
5	5	5	5	5	5	5	5	5
5	5	1	5	5	5	5	5	5
4	4	2	3	5	4	3	4	5
3	2	1	3	5	3	3	5	2
5	5	1	5	5	5	5	5	5
5	5	1	5	5	5	5	5	5
5	5	1	5	5	5	5	5	5
4	3	1	2	2	3	3	5	2
5	5	1	5	5	5	5	5	5
5	5	1	5	5	5	5	5	5
5	5	1	5	5	5	5	3	5
3	3	3	3	3	3	3	3	3
2	4	3	2	3	2	3	3	5
4	5	1	3	4	3	3	5	5
5	5	1	5	5	5	5	5	5
5	5	1	5	5	5	5	5	5
4	4	1	4	5	4	5	5	5
5	5	1	5	5	5	5	5	5
3	3	3	3	3	3	3	3	3
2	2	1	2	2	2	2	5	2
3	2	1	2	5	5	5	5	5
5	5	1	5	5	5	5	5	5
5	5	1	5	5	5	5	5	5
4	4	1	3	5	3	3	5	5
4	4	3	3	3	3	3	3	3
1	1	3	1	1	1	1	5	5
3	3	4	3	3	3	3	5	5
5	5	5	5	5	5	5	5	5
4	3	1	3	5	3	5	3	5
5	4	1	4	4	4	4	4	5
3	4	1	3	5	3	5	5	5
5	5	1	5	5	5	5	2	5
4	4	4	4	4	4	4	4	4
5	5	2	5	5	5	5	5	4
5	5	1	5	5	5	5	5	5
3	4	1	5	3	3	3	5	4
3	5	2	4	5	4	4	4	5
4	4	2	5	5	5	5	5	5
2	3	1	3	3	3	3	5	4
5	5	1	5	5	5	5	5	5
3	3	1	3	4	3	4	5	5
4	4	1	5	5	5	3	3	5
5	5	1	5	5	5	5	5	5
4	2	3	3	3	3	3	3	3
4	2	3	3	3	3	3	3	3
5	5	1	5	5	5	5	5	3
3	3	1	3	3	3	3	3	4
4	4	1	5	5	5	5	3	5

4	4	1	4	5	4	3	5	5
5	5	1	5	5	5	5	4	5
5	5	1	5	5	5	3	4	4
3	3	2	4	4	3	3	4	5
5	5	5	5	5	5	5	5	5
3	5	1	5	5	5	5	5	1
4	1	4	4	4	2	4	2	4
5	5	1	5	5	5	5	5	5
1	1	1	2	3	2	1	2	3
2	1	3	2	2	1	2	5	5
3	2	1	3	3	4	3	4	5
5	5	1	5	5	5	5	5	5
5	5	1	5	5	5	5	5	5
3	4	1	3	5	5	5	5	5
5	5	1	3	5	5	5	5	5
5	5	1	5	5	5	5	5	5
5	5	1	5	5	5	5	5	5
3	3	5	5	5	5	5	5	5
5	5	1	5	5	5	5	5	5
2	2	2	2	3	3	2	3	3
5	5	2	5	5	5	5	3	5
4	4	1	5	4	4	5	5	5
4	5	1	3	5	4	5	5	5
3	3	1	3	3	3	3	3	5
5	5	5	5	5	5	5	5	5
1	1	5	1	1	1	1	3	5
5	3	1	5	5	5	5	4	5
2	2	1	4	4	3	3	4	4
5	5	1	5	5	2	5	4	1
4	5	1	5	5	5	5	5	5
5	5	1	5	5	5	5	5	3
4	5	1	3	5	4	4	3	5
4	4	1	3	4	3	3	3	3
2	4	3	2	3	2	3	3	5
4	3	1	3	3	3	3	3	5
2	2	4	5	5	3	3	3	5
5	5	1	5	5	5	5	5	5
4	4	1	3	4	5	4	3	5
4	4	1	3	4	3	4	3	5
2	3	3	2	3	4	3	4	3
3	4	2	3	3	3	3	3	3
3	3	3	3	3	3	3	4	3
3	4	1	3	3	3	4	4	5
4	4	1	5	5	5	5	3	4
3	3	3	3	4	5	3	2	4
3	3	2	3	3	3	3	3	4
5	5	1	5	5	5	5	1	5
5	5	3	5	5	5	5	2	5
4	3	1	3	5	5	4	3	5
3	3	2	3	5	3	4	3	4
3	3	2	2	1	1	3	2	4
3	2	2	4	5	3	3	4	3
5	3	2	3	3	4	5	2	5
2	2	2	2	2	2	2	3	4

4	3	1	3	5	4	4	4	4
4	4	2	4	5	5	4	3	4
3	2	1	2	2	3	2	4	3
5	5	1	5	5	5	5	5	5
5	5	5	5	5	5	5	5	5
3	4	1	4	3	3	3	3	3
4	3	1	4	4	5	5	3	5
4	5	1	4	3	3	4	4	3
2	2	1	3	3	2	2	3	4
4	5	4	5	5	5	5	5	5
4	3	1	3	4	4	4	5	5
4	4	4	3	3	4	3	3	3
5	5	1	5	5	5	5	5	5
4	4	1	5	5	5	5	5	5
5	4	1	5	5	5	5	5	5
5	5	5	5	5	5	5	5	5
5	5	5	5	5	5	5	5	5
3	3	2	4	4	4	4	2	3
4	3	3	4	4	3	4	4	4
2	3	4	2	3	3	3	3	3
5	5	1	5	5	5	5	3	5
3	3	3	3	3	3	3	3	3
4	3	1	4	3	4	3	3	3
2	2	2	2	5	3	3	5	5
4	4	1	4	4	3	3	4	4
5	5	1	3	2	5	3	3	5
3	2	2	3	3	3	3	3	3
2	3	2	3	5	3	3	3	5
2	3	1	3	3	4	4	4	5
3	3	2	3	3	3	3	4	3
4	4	1	4	4	3	3	5	4
3	3	1	3	3	3	3	3	5
2	2	2	3	3	3	3	2	3
3	2	1	2	3	2	3	3	1
4	5	1	5	5	5	5	4	3
3	3	1	3	2	2	2	2	5
2	1	1	2	1	1	1	5	3
3	3	3	3	3	3	3	3	3
4	4	1	5	5	4	4	4	4
5	5	5	5	5	5	5	5	5
3	2	1	5	3	3	3	5	5
5	5	2	2	2	3	3	3	3
3	3	1	3	3	3	3	3	4
2	2	2	2	2	2	2	4	2
2	2	1	3	5	2	1	5	5
4	4	2	4	5	5	5	5	5
2	2	1	3	5	4	3	5	5
3	3	3	3	3	3	3	4	3
5	5	3	5	5	5	5	5	5
4	4	4	4	4	4	4	4	4
5	5	2	2	5	5	5	2	5
4	3	3	3	3	3	3	3	3
2	3	2	3	3	2	3	2	3
5	4	4	4	4	4	4	4	4

3	4	1	3	2	2	2	3	5
5	5	2	5	5	5	5	5	5
2	3	3	2	3	4	3	4	3
3	3	1	3	3	3	3	3	5
3	3	1	3	4	3	3	3	4
5	5	1	5	5	5	5	5	5
4	3	1	3	3	3	3	4	3
4	5	1	3	3	5	2	3	5
4	3	1	5	5	5	5	5	5
2	2	1	1	1	1	5	5	5
4	3	1	3	3	3	3	3	3
3	3	1	3	3	3	3	4	5
4	4	2	4	4	4	4	4	5
3	3	2	2	2	3	4	5	4
3	3	3	3	3	3	3	3	3
3	3	3	3	3	3	3	2	3
2	2	2	3	5	4	3	5	5
4	3	1	3	3	3	4	2	4
5	5	1	5	5	5	5	5	5
5	5	4	5	5	5	5	1	5
5	4	1	5	5	5	3	3	4
5	5	1	5	5	5	5	5	5
4	4	1	4	4	4	4	3	3
4	5	2	5	5	5	5	3	5
2	1	1	5	5	5	5	1	5
4	3	3	3	3	3	3	3	3
5	5	4	3	3	5	5	3	2
2	2	1	3	3	5	3	2	5
4	3	1	3	5	3	3	5	5
3	3	1	3	5	5	3	5	5
3	3	1	4	5	5	5	4	5
3	3	3	3	3	3	3	3	3
4	5	1	4	4	5	4	2	4
2	5	1	1	4	5	1	1	5
3	3	1	2	3	1	2	3	5
2	2	1	1	5	2	1	5	5
3	3	1	5	5	5	5	2	5
5	5	1	2	4	3	3	3	3
5	5	1	3	3	3	3	3	5
3	2	2	5	5	2	2	5	5
5	5	1	5	5	5	5	5	5
2	2	2	2	2	2	2	2	2
3	2	1	2	2	2	2	3	3
5	5	2	5	5	4	5	3	5
4	4	4	4	4	4	4	4	4
4	4	1	4	5	4	4	5	5
4	4	1	3	3	3	3	3	3
5	3	1	1	1	1	3	5	3
5	5	5	5	5	5	5	5	5
5	5	1	5	5	5	5	5	5
1	3	1	5	5	5	5	3	5
3	3	1	3	3	3	3	3	3
5	3	1	3	3	3	3	3	4
4	3	2	4	3	4	3	2	5

3	2	2	1	1	1	1	5	3
5	3	1	5	5	3	3	5	5
4	4	1	4	4	4	4	4	4
4	3	1	3	3	3	3	3	4
1	1	2	1	1	1	1	2	5
3	3	1	1	5	3	3	5	5
4	4	1	3	4	4	4	4	4
3	3	1	3	3	3	3	3	3
5	5	2	5	5	5	5	3	3
5	5	2	5	5	5	5	5	5
5	5	3	5	5	5	5	5	5
5	5	5	5	5	5	5	5	5
2	4	4	3	4	4	5	4	3
3	2	1	3	3	3	3	2	3
4	5	1	3	3	3	3	3	3
5	5	1	5	5	5	5	5	5
5	5	1	5	5	5	5	5	5
3	3	2	3	3	3	3	3	3
3	3	1	3	4	3	3	3	5
5	4	2	5	5	5	5	5	5
3	3	1	3	3	3	3	4	4
5	5	3	3	3	4	4	3	4
5	5	1	5	5	5	5	1	5
2	2	4	3	3	3	2	3	3
2	1	2	1	2	1	2	4	1
1	1	1	1	3	1	1	1	4
5	5	2	5	5	5	5	5	5
4	4	2	3	3	3	3	3	4
5	5	5	5	5	5	5	5	5
2	2	2	2	2	2	2	3	4
4	4	2	4	4	4	4	4	4
5	5	1	5	5	5	5	1	5
3	3	2	3	3	3	3	3	4
4	4	3	3	3	3	3	3	4
5	5	1	5	5	5	5	1	5
3	3	2	3	3	3	3	5	4
5	5	1	5	5	5	5	5	5
3	3	3	3	3	3	3	3	3
3	3	3	3	3	3	3	3	3
5	5	1	5	5	5	5	5	5
5	4	1	3	3	3	3	3	5
2	4	1	2	4	2	3	4	5
3	3	1	5	5	3	3	4	5
3	5	1	3	4	3	3	5	5
5	5	1	5	5	5	5	5	5
5	5	2	5	5	5	5	2	5
4	5	1	4	3	3	4	4	3
3	5	2	3	4	4	4	5	3
1	1	1	1	1	1	1	2	5
3	4	2	3	3	3	3	3	3
3	3	3	3	3	3	3	3	3
1	3	1	2	5	2	2	5	5
4	3	2	3	4	3	3	5	4
3	3	1	3	3	3	3	3	5

5	4	4	4	4	4	4	4	4
5	5	5	5	5	5	5	5	5
4	4	1	4	4	4	4	1	4
3	2	4	2	4	2	4	2	4
4	4	1	5	5	5	5	5	5
5	5	3	5	5	5	5	5	4
1	2	1	3	2	1	1	1	3
3	3	2	3	2	2	2	4	4
3	3	1	1	3	2	1	3	5
3	5	1	3	2	2	2	3	5
2	3	2	3	4	3	3	5	3
3	5	1	3	3	3	3	4	5
2	3	1	5	5	5	3	5	5
3	4	1	2	5	3	3	4	5
3	3	3	3	3	3	3	3	3
3	3	1	3	2	2	2	5	5
2	3	2	2	2	2	2	3	3
3	3	1	3	3	3	3	4	2
5	5	1	5	5	5	5	5	5
3	2	1	2	5	3	3	4	5
4	5	1	3	3	3	3	3	3
2	2	1	2	3	3	3	3	3
3	3	3	3	3	3	3	3	3
4	4	4	4	4	4	4	4	4
5	5	1	5	5	5	5	3	5
3	5	1	1	5	1	1	5	5
3	5	1	5	5	5	5	5	5
3	4	2	3	3	3	3	3	3
4	4	1	2	3	3	4	3	2
2	3	3	4	5	4	5	3	5
1	1	1	1	1	1	1	1	1
3	3	1	3	3	3	3	3	5
5	5	5	5	5	5	5	5	5
4	4	4	4	4	4	4	4	4
3	3	2	4	4	4	4	3	4
5	5	5	5	5	5	5	5	5
5	5	5	5	5	5	5	5	5
5	5	2	5	5	5	5	5	5
4	5	5	5	5	5	5	5	5
5	5	3	5	5	5	5	5	5
4	4	1	4	4	4	4	4	4
3	4	1	5	4	5	5	5	4
5	5	1	5	5	5	5	5	5
3	4	3	3	3	4	4	3	4
4	4	4	4	4	4	4	4	4
3	2	2	3	3	3	3	3	4
4	3	2	3	3	4	4	5	3
3	3	2	3	4	4	3	5	3
4	3	2	4	4	4	4	3	4
5	5	5	5	5	5	5	5	5
4	4	1	4	4	4	4	4	4
5	5	1	5	5	5	5	5	5
5	5	1	5	5	5	5	5	5
1	1	1	5	5	5	5	3	3

5	5	5	5	5	5	5	5	5
5	5	1	5	5	5	5	5	5
4	3	1	4	5	5	5	2	5
3	3	1	2	3	2	2	5	5
4	3	3	3	4	3	3	4	5
5	5	2	5	5	5	5	5	5
5	5	1	5	5	5	5	5	5
4	5	1	4	5	5	5	4	5
4	5	1	3	5	3	3	3	5
5	5	1	5	5	5	5	5	5
5	4	1	5	5	5	5	5	5
3	3	3	3	4	4	3	3	3
3	4	1	5	5	5	4	5	5
4	3	3	4	4	4	4	3	4
4	4	2	3	4	4	4	4	4
1	2	1	2	2	2	2	2	2
3	3	2	3	3	3	3	4	4
3	3	4	3	3	3	3	3	3
3	4	1	3	3	3	3	1	4
5	4	1	5	5	5	5	5	5
5	5	1	5	5	5	5	5	5
3	3	3	3	3	3	3	5	3
4	4	3	3	3	3	3	3	3
3	3	1	3	3	4	3	3	4
5	5	1	5	5	5	5	5	5
3	1	1	2	3	1	1	5	5
4	4	2	3	3	3	3	4	4
3	3	1	3	2	3	3	4	5
3	3	1	3	3	3	3	3	3
3	3	2	2	2	2	2	3	3
2	4	1	3	4	4	4	4	4
2	3	4	2	5	3	3	4	5
5	5	5	5	5	5	5	5	5
5	5	4	5	5	5	5	3	5
3	3	1	5	5	5	5	5	5
4	3	3	4	4	4	4	3	4
5	5	1	5	5	5	5	5	5
2	2	2	2	2	2	2	2	2
5	5	1	5	5	5	5	3	3
4	4	2	3	5	5	5	5	5
5	5	5	5	5	5	5	5	5
5	5	1	1	1	1	1	1	1
5	5	2	5	5	5	5	4	4
4	4	1	4	5	5	4	1	5
5	5	1	5	5	5	5	5	5
3	2	1	5	2	3	4	1	2
1	1	1	1	1	1	1	5	1
5	5	1	3	3	3	3	3	3
3	3	4	3	3	3	3	3	3
5	5	1	5	5	5	5	3	5
4	3	3	4	3	3	3	1	3
3	3	1	3	4	3	3	5	5
5	5	5	5	5	5	5	5	5
5	5	1	5	5	3	3	5	5

5	5	5	5	5	5	5	5	1
5	5	5	5	5	5	5	5	5
3	3	1	3	3	3	3	2	3
4	4	1	3	4	3	4	4	3
3	3	1	5	5	5	5	5	5
5	5	1	5	5	5	5	5	5
3	3	1	2	2	3	3	3	5
5	5	3	3	3	3	3	5	5
5	5	5	5	5	5	5	5	5
5	5	1	5	5	5	5	5	5
5	5	1	5	5	5	5	5	5
5	5	3	3	3	3	3	5	5
5	5	1	5	5	5	5	5	5
5	5	1	5	5	5	5	5	5
5	5	5	5	5	5	5	5	5
4	3	1	1	3	5	5	4	4
4	1	1	2	5	3	3	5	5
1	5	1	5	5	5	5	1	1
5	5	1	5	5	5	5	5	5
5	5	1	5	5	5	5	5	5
1	3	3	2	3	1	1	1	3
5	5	1	5	5	5	5	5	5
5	5	1	5	5	5	5	5	5
4	3	1	4	5	5	4	5	1
5	5	1	5	5	5	4	3	3
5	5	5	5	5	5	5	5	5
4	5	1	5	5	5	5	4	5
3	5	1	4	4	3	3	5	5
3	5	2	3	3	3	3	3	5
5	5	1	5	5	5	5	5	5
3	2	4	3	2	3	4	5	3
5	5	1	5	5	5	5	5	5
5	5	1	5	5	5	5	5	5
4	4	2	5	5	5	5	5	5
1	1	2	1	3	1	1	3	4
3	2	1	2	3	3	3	5	1
3	3	1	3	4	4	3	5	5
5	5	1	5	5	5	5	5	5
1	5	1	1	5	5	5	1	5
4	5	2	4	5	5	5	5	4
4	4	1	4	4	4	4	4	4
5	5	1	5	5	5	5	5	5
5	5	1	5	5	5	5	5	5
5	5	1	5	5	5	5	4	5
4	4	1	3	4	3	3	3	5
4	5	1	5	4	5	4	4	5
5	5	5	5	5	5	5	5	1
3	4	1	5	5	2	2	5	5
3	3	1	2	3	2	2	5	2
4	3	1	5	5	5	5	5	5
1	5	1	5	5	5	5	3	5
4	5	1	5	4	5	4	4	5
5	5	5	5	5	5	5	5	5
3	2	1	5	5	2	5	5	5

5	5	1	4	4	4	4	4	4
5	5	1	5	5	5	5	5	5
5	5	1	5	5	5	5	5	5
3	3	1	3	3	3	5	5	5
5	5	1	5	5	5	5	5	5
5	5	1	5	5	5	5	5	5
5	5	1	5	5	5	3	5	5
4	1	1	2	1	1	1	1	1
5	5	1	5	5	5	5	3	5
5	5	1	5	5	5	5	5	5
5	5	1	5	5	5	5	5	5
4	5	1	5	5	3	5	5	4
5	5	1	5	5	5	5	5	5
4	4	4	4	4	4	4	4	5
4	4	2	2	5	2	2	3	3
5	5	1	5	5	5	5	5	5
4	4	2	2	5	2	2	3	3
5	5	5	5	5	5	5	5	5
5	5	1	5	5	5	5	5	3
4	4	1	3	4	3	3	3	3
4	4	1	4	3	3	3	5	5
4	4	4	4	4	4	4	4	4
5	5	1	5	5	5	5	5	5
5	5	1	5	5	5	5	1	5
2	2	2	2	2	2	2	2	2
4	4	2	3	3	3	2	4	4
3	2	4	2	2	4	4	2	3
3	3	1	2	2	2	2	4	3
2	2	1	3	3	5	3	2	5
3	3	1	2	5	2	2	3	2
3	3	1	3	3	3	3	5	5
3	5	1	5	5	5	5	5	1
5	5	1	5	5	5	5	5	5
2	4	2	2	3	2	2	4	5
5	5	1	5	5	5	5	1	5
3	3	2	3	3	3	3	4	4
3	3	1	3	3	3	3	4	4
2	2	1	5	5	3	2	3	2
3	4	1	4	4	3	3	3	3
4	4	4	4	4	4	4	4	4
4	4	1	1	4	3	3	5	5
5	5	1	5	5	5	5	5	5
5	5	2	5	5	5	5	2	5
2	2	4	2	3	1	1	3	3
2	2	2	2	2	3	2	5	4
5	5	1	5	5	5	5	5	5
3	4	2	3	4	3	3	4	3
5	5	2	5	5	5	5	3	5
3	5	4	3	3	3	3	3	5
2	3	1	1	3	2	2	4	5
3	4	1	3	3	3	3	2	5
5	5	1	5	5	5	5	3	5
3	3	3	3	3	3	3	3	3
2	3	2	4	3	3	2	4	3

3	4	1	3	3	3	3	1	3
4	4	1	3	3	4	3	5	4
4	3	1	2	3	3	4	4	5
5	5	1	5	5	4	4	3	4
2	1	1	1	1	1	1	2	2
2	5	1	1	5	5	1	2	5
3	3	3	2	5	3	4	2	4
3	3	1	4	4	4	3	1	4
3	3	1	5	5	5	3	5	4
5	5	1	5	5	1	1	2	3
5	5	1	5	5	5	5	5	5
1	1	1	1	1	1	1	1	1
5	5	1	5	4	5	3	4	3
3	3	1	5	5	3	3	4	5
5	5	1	5	5	5	5	5	5
4	3	1	5	5	4	4	5	5
3	4	1	3	3	3	3	3	5
1	3	3	2	3	2	3	3	2
3	3	1	3	3	3	3	5	4
2	4	3	3	2	2	2	5	4
5	5	5	5	5	5	5	5	5
3	3	1	3	3	3	3	4	3
3	3	3	3	3	3	3	3	3
4	4	1	4	4	5	3	3	3
3	4	3	3	4	4	4	5	4
3	3	1	3	5	3	2	4	5
1	1	1	1	5	1	1	3	3
1	1	1	1	1	1	1	3	4
5	2	4	2	2	2	2	2	4
3	3	3	2	3	3	2	3	4
4	4	1	5	5	4	4	5	5
3	3	3	4	4	4	4	4	4
4	4	1	5	5	5	4	5	5
5	5	1	5	5	5	5	5	5
1	1	3	1	4	4	1	4	1
4	4	1	4	4	5	3	3	3
4	4	1	3	4	4	3	3	4
3	3	4	3	5	4	3	4	5
3	3	3	2	3	3	3	3	3
2	2	5	3	2	2	2	2	2
4	4	4	4	4	4	4	4	4
5	5	1	4	5	4	4	4	5
5	5	1	5	5	5	5	5	5
3	4	1	3	3	3	4	4	5
4	5	1	4	5	4	4	3	3
4	5	1	4	4	3	3	5	5
3	3	2	3	4	4	4	4	4
2	3	2	2	3	3	3	2	4
3	3	1	3	3	2	2	1	2
1	2	1	3	3	2	2	3	2
5	5	1	5	5	5	5	4	5
5	5	1	5	5	5	5	5	4
5	5	1	5	5	3	3	4	4
4	4	2	4	5	5	4	2	5

4	3	1	3	4	4	3	4	4
3	3	3	3	3	4	3	3	3
5	5	1	5	5	5	5	5	5
4	4	1	4	4	4	4	4	5
5	5	1	3	5	3	3	4	4
4	4	1	3	2	3	3	3	3
5	5	1	5	5	5	5	5	5
4	3	1	4	5	5	5	3	5
2	2	1	4	4	4	5	2	5
2	2	2	5	5	2	2	5	4
2	2	2	5	5	2	2	5	4
4	3	4	4	5	5	3	4	5
3	3	3	2	4	3	4	5	5
4	4	2	3	5	3	3	3	3
5	3	1	5	5	5	5	4	5
5	5	1	5	5	5	5	5	5
5	5	5	5	5	5	5	5	5
4	4	1	5	5	5	5	5	5
3	4	2	2	3	3	5	5	5
4	3	1	5	4	3	4	3	5
3	3	1	5	5	5	5	4	5
5	5	1	5	5	4	3	5	5
5	5	1	5	5	5	5	5	5
5	5	2	5	5	5	5	3	5
5	5	1	4	5	5	5	5	5
2	1	1	1	2	2	1	4	2
4	4	1	5	5	5	5	3	5
5	5	1	5	5	5	5	3	5
3	3	1	3	2	2	2	4	4
5	5	1	5	5	5	5	5	5
3	2	1	3	5	4	2	5	5
5	4	1	5	5	4	4	5	5
5	5	2	5	5	5	5	2	5
3	2	2	2	4	4	5	5	5
3	3	1	3	5	5	4	5	5
3	4	1	5	5	5	5	4	5
3	3	3	2	3	2	3	4	4
4	3	2	3	3	2	2	4	1
5	5	5	5	5	1	5	5	5
5	5	1	5	5	5	5	5	5
3	5	2	5	5	5	5	5	5
5	4	2	3	3	3	3	5	5
5	1	1	1	1	1	1	1	1
5	5	5	5	5	5	5	5	5
3	3	3	3	5	3	3	3	5
3	3	3	2	3	3	2	4	4
3	2	2	2	4	4	5	5	5
3	3	1	3	3	3	3	3	5
4	4	1	4	4	4	4	4	4
3	3	1	4	4	3	4	2	4
4	3	1	3	5	3	4	5	5
5	5	1	5	5	5	5	3	5
4	3	1	4	4	5	3	5	5
5	4	1	5	5	5	5	3	5

4	4	2	3	5	4	2	5	5
3	3	2	2	3	2	4	3	5
3	2	1	3	3	3	3	5	5
3	3	1	3	3	1	1	5	5
3	3	1	3	5	3	3	3	5
1	2	3	2	2	1	1	5	3
4	3	2	3	1	1	1	2	3
4	3	1	5	4	3	3	5	5
4	4	2	3	4	5	4	4	5
3	2	1	5	4	3	3	3	5
5	5	3	4	5	5	5	2	3
3	3	3	3	3	3	3	3	5
3	3	2	3	3	3	3	5	3
4	4	1	5	5	5	5	5	5
4	4	1	5	5	5	5	5	5
4	3	1	5	5	5	5	2	1
4	3	2	3	3	4	3	3	5
3	3	2	3	3	5	5	3	5
5	3	3	3	3	3	3	3	3
3	4	2	5	5	5	5	5	5
3	1	3	3	4	2	2	5	4
1	1	3	1	2	1	1	1	5
5	5	5	5	5	5	5	5	5
5	5	1	5	5	5	5	5	3
4	4	1	4	4	4	4	2	5
4	4	3	3	4	3	3	3	4
2	3	2	3	3	3	3	3	3
4	4	1	5	5	5	5	3	4
2	2	2	2	2	2	2	2	2
4	4	1	4	4	4	4	3	3
3	3	3	3	3	3	3	3	3
3	3	1	3	3	3	2	3	4
5	5	1	5	5	5	5	3	5
3	3	3	4	4	4	4	3	5
3	5	2	4	4	4	3	3	5
4	4	3	4	4	4	4	3	4
5	5	1	5	5	5	5	3	5
4	4	1	5	5	5	5	5	5
3	3	1	4	4	4	4	3	4
5	4	1	5	5	5	5	5	5
4	4	1	4	4	4	4	4	4
5	5	1	5	5	5	5	5	5
3	2	2	3	3	2	2	4	4
5	3	4	4	3	5	3	2	5
2	2	1	2	2	1	1	1	5
5	5	1	5	5	5	5	5	5
5	5	1	5	5	5	5	5	5
4	3	2	3	4	4	4	3	4
4	4	1	4	4	4	4	4	5
4	4	1	4	5	5	5	4	4
4	4	1	4	5	5	5	4	5
2	2	1	3	3	3	3	3	3
4	3	1	3	5	5	5	2	4
5	5	5	5	5	5	4	4	3

3	3	2	3	5	5	5	3	5
4	4	1	4	4	4	4	3	5
3	3	2	1	4	4	4	3	5
1	1	1	1	1	1	1	1	1
5	5	1	5	5	5	5	4	5
5	5	5	5	5	5	4	4	3

B11	B12	B13	Health Behavic	C1	C2	C3	C4	C5
5	5	5	59	5	5	5	5	5
3	5	1	40	2	3	4	1	3
5	5	5	65	5	4	5	3	5
5	4	4	61	1	5	5	5	3
5	5	5	47	2	5	4	3	3
5	5	5	65	2	4	5	5	5
3	2	5	37	2	4	5	2	2
5	5	2	39	1	4	4	2	2
4	5	3	36	5	3	4	2	2
5	5	5	61	1	5	5	5	5
5	4	4	45	1	5	5	5	5
4	4	4	50	2	4	5	4	2
5	5	5	59	1	5	5	5	4
5	5	1	48	5	1	3	2	1
5	4	5	61	1	4	4	3	1
1	1	5	39	5	5	5	4	5
3	3	3	35	2	4	4	3	4
5	5	5	37	1	5	5	5	5
3	3	3	36	2	4	4	2	3
3	4	2	40	1	4	4	2	3
4	4	4	35	2	4	4	3	4
2	4	2	61	2	3	3	3	5
5	5	5	56	1	5	5	5	5
3	4	4	44	1	5	5	5	5
5	4	4	58	2	5	5	1	2
5	5	5	51	1	4	4	4	4
4	4	3	42	3	3	3	3	2
3	3	3	45	2	4	4	4	4
4	4	3	47	1	5	5	5	5
4	4	4	41	2	3	5	3	4
5	5	5	60	1	5	5	5	3
4	5	5	52	1	5	5	5	5
5	5	5	52	1	5	5	5	3
4	4	4	51	1	5	5	5	5
2	3	5	64	2	4	5	4	3
5	5	5	61	3	5	5	4	4
5	5	5	34	1	5	5	5	5
5	5	5	33	5	5	5	5	5
5	5	5	50	2	5	5	4	4
5	5	5	56	5	5	5	5	5
5	5	5	52	1	4	4	5	4
4	4	4	53	2	4	4	4	4
5	5	5	34	4	4	4	4	4
4	4	4	59	2	4	5	5	3
5	5	5	31	2	4	4	4	2
4	4	4	65	3	4	4	4	4
4	4	4	40	1	4	1	4	3
5	5	5	45	5	5	5	5	5
3	3	3	61	3	3	3	3	3
1	4	5	59	1	5	3	3	2
5	5	5	42	2	1	5	4	3
5	5	5	43	1	5	5	5	5
5	5	5	65	1	5	5	5	4

3	3	3	43	2	4	3	3	2
4	4	3	60	1	4	4	4	4
4	4	4	61	3	5	5	5	5
5	5	5	65	1	5	5	5	5
5	5	5	45	2	5	5	5	1
5	5	5	55	1	4	4	4	3
5	5	5	51	1	5	5	5	3
5	5	5	43	1	5	5	5	5
5	5	5	59	2	2	4	3	4
5	5	4	62	1	5	4	3	3
4	3	3	60	1	4	4	4	4
5	5	3	50	2	4	4	4	3
5	5	5	65	1	5	5	5	4
3	3	3	57	4	5	5	4	5
5	5	5	43	1	5	5	4	2
5	5	3	53	1	5	5	3	3
5	5	5	50	1	5	5	3	5
5	5	5	61	2	5	5	5	2
5	5	4	43	4	4	5	4	2
4	5	3	37	1	4	5	3	3
4	3	4	65	2	4	4	4	4
5	5	5	41	1	5	5	4	3
4	3	3	54	3	4	3	1	1
3	3	3	45	3	3	4	3	3
5	5	5	49	3	3	3	3	1
5	3	5	61	3	4	4	2	2
4	5	3	37	1	5	5	4	4
4	5	5	44	3	4	4	3	3
4	4	4	54	3	3	3	3	3
5	5	5	45	3	3	3	4	3
3	2	3	51	3	3	3	2	1
3	5	2	55	1	5	3	5	3
5	5	4	61	1	5	5	5	3
3	4	3	46	3	3	2	3	3
5	5	5	43	4	4	3	3	4
5	5	3	61	1	5	5	5	5
5	5	5	39	1	5	5	4	3
4	4	4	44	2	4	3	3	3
5	5	5	27	2	4	4	4	3
5	5	5	61	1	5	5	5	3
4	4	2	41	3	2	4	3	2
4	3	3	49	1	5	4	4	4
3	1	2	60	3	3	3	3	3
5	5	5	47	1	5	5	5	2
3	2	5	61	4	3	4	3	1
4	4	3	47	2	4	2	3	2
4	5	4	42	4	4	3	3	3
5	3	3	41	2	4	5	3	3
5	5	5	65	1	5	5	5	5
5	5	3	51	3	4	5	3	3
5	4	2	39	4	2	4	3	1
5	5	5	31	2	4	4	4	3
5	5	5	56	2	2	2	2	2
5	4	5	31	2	3	4	2	2

3	3	3	53	1	3	3	3	3
3	2	3	46	3	2	3	1	3
5	5	5	48	1	5	5	4	4
5	5	5	48	2	5	5	4	2
5	5	5	53	1	5	5	5	5
5	3	3	41	2	5	5	2	4
4	4	3	43	3	3	4	3	3
4	5	5	57	2	5	5	3	2
5	5	5	52	1	5	5	4	4
3	3	3	57	3	4	3	3	3
3	4	4	57	2	2	3	3	3
5	5	5	56	1	5	5	5	4
5	3	5	52	1	5	5	5	3
5	5	5	56	2	4	4	4	3
5	5	5	55	1	5	5	5	4
5	5	4	45	2	4	5	4	3
5	5	2	44	2	5	4	4	3
5	5	5	47	1	5	5	5	2
5	5	4	46	2	4	4	4	3
4	4	4	41	2	5	4	3	4
3	3	4	40	1	5	5	4	2
5	5	5	39	1	5	5	5	5
5	5	5	44	4	3	3	5	3
4	3	3	31	3	4	3	3	3
3	3	3	41	3	2	4	2	2
3	3	3	61	3	2	4	1	2
4	3	3	65	3	4	2	2	1
4	2	1	47	3	2	2	1	3
4	4	3	48	3	4	4	2	3
5	5	5	35	1	5	5	4	3
5	5	5	61	1	4	4	4	4
3	5	3	57	2	5	5	3	2
4	4	4	65	3	3	3	3	1
3	3	5	63	5	1	1	1	1
5	5	5	61	1	4	4	3	2
5	5	4	50	1	5	5	4	3
5	5	5	44	3	3	3	3	3
5	5	5	48	1	5	5	5	5
5	5	5	42	1	1	3	3	4
5	5	5	41	3	5	5	4	2
5	5	4	49	1	5	5	4	2
5	3	4	48	3	3	2	2	1
3	3	3	36	3	3	3	4	2
4	3	3	54	3	3	4	2	1
5	5	5	54	3	5	5	2	1
5	4	2	44	1	5	5	4	3
2	1	1	56	2	4	3	1	1
5	3	5	45	2	5	5	4	3
5	4	4	44	2	4	4	4	3
4	3	3	50	2	4	4	2	4
5	5	4	55	1	5	5	4	4
5	5	3	47	2	5	5	3	3
4	4	3	45	3	4	4	3	3
5	4	3	51	2	4	4	4	4

5	5	4	43	1	5	4	4	3
5	4	3	54	2	4	4	3	4
5	5	5	57	1	5	5	4	4
4	5	3	58	2	4	4	3	2
4	4	2	31	2	5	5	4	5
5	5	5	36	4	4	4	4	2
5	5	4	57	1	4	4	4	3
5	5	5	45	2	4	4	4	4
5	3	2	58	3	3	3	2	2
3	3	4	60	2	4	4	4	1
5	5	3	44	2	4	4	3	2
5	3	3	50	1	5	5	5	4
5	5	5	55	3	3	3	3	3
5	5	5	46	1	5	5	5	5
5	4	3	35	2	4	4	3	3
5	5	5	61	1	5	5	3	3
4	5	5	45	2	4	5	5	5
4	3	4	61	2	5	4	4	4
2	4	3	52	2	2	2	2	2
5	5	5	54	2	5	5	5	3
3	4	3	45	2	4	5	4	4
5	5	5	51	1	5	5	4	5
5	5	3	42	1	4	4	3	2
5	5	5	34	1	5	5	5	4
4	3	1	43	3	2	1	2	1
5	5	3	62	2	4	4	3	1
3	3	4	55	2	4	5	4	2
5	5	1	61	2	4	5	4	2
5	3	3	55	4	2	2	1	1
5	5	5	41	1	5	5	5	4
5	5	5	42	4	4	4	3	3
5	5	5	52	1	5	5	5	5
5	5	3	52	1	5	5	5	3
2	2	4	60	1	4	5	3	2
1	5	3	51	4	5	5	4	4
3	3	5	34	2	5	5	5	4
5	5	3	33	3	4	4	4	2
5	5	5	57	1	5	5	5	5
5	5	5	44	3	4	4	4	3
5	5	5	57	1	5	5	5	5
5	5	5	47	1	5	5	5	4
5	5	5	50	1	5	5	5	5
3	3	5	45	1	5	5	5	1
5	5	4	57	2	4	5	4	4
5	5	4	57	2	4	4	4	3
5	5	3	61	2	4	4	3	3
5	5	5	54	3	3	3	3	3
5	5	1	61	1	5	5	5	4
5	5	5	61	1	5	5	5	2
5	5	5	34	1	5	5	5	5
5	5	5	58	2	5	1	5	4
5	5	5	43	1	4	5	4	3
5	5	5	58	1	5	5	4	3
2	2	4	53	1	5	5	4	4

5	5	5	59	3	5	5	5	3
3	5	5	31	1	5	5	3	1
5	5	5	31	1	5	5	4	4
5	5	3	61	2	4	4	3	3
5	5	5	51	3	5	4	2	3
5	5	5	45	5	5	5	5	5
5	5	5	61	4	4	4	4	4
5	5	5	32	1	5	5	5	2
4	4	4	61	2	4	4	4	3
4	4	5	41	2	4	4	4	2
5	5	5	61	2	4	3	3	3
5	5	5	59	2	5	5	3	3
5	5	5	59	1	1	1	1	1
5	3	3	39	1	1	5	3	4
5	5	5	46	3	3	4	4	1
5	5	3	53	1	5	5	5	5
5	5	5	59	2	2	5	5	5
3	3	3	61	1	4	4	4	3
5	5	4	57	2	4	4	3	2
5	5	5	61	1	5	5	3	4
5	5	3	39	3	4	4	3	3
5	5	5	34	3	4	4	3	2
5	5	5	49	1	5	5	4	4
5	5	5	61	1	5	5	5	3
3	3	3	34	3	4	5	3	2
3	3	3	49	3	4	4	2	2
5	5	5	42	3	4	4	3	2
5	5	5	31	1	5	5	5	5
5	3	4	43	1	5	4	5	3
5	3	3	34	1	5	5	4	4
3	3	3	49	2	2	4	3	4
3	5	1	55	5	1	3	1	1
3	1	2	54	1	4	3	2	2
5	5	5	54	1	5	5	5	5
5	4	3	44	1	5	4	3	4
5	5	5	61	2	4	3	4	3
5	5	5	34	1	3	4	3	4
5	5	5	50	1	5	5	3	2
4	4	4	54	2	4	4	4	2
5	5	5	57	2	4	5	5	5
5	5	1	60	1	4	4	4	4
5	5	4	47	1	5	3	3	2
5	3	5	61	3	5	5	4	3
5	5	5	48	1	5	5	5	1
5	5	5	54	3	5	5	3	5
5	5	5	32	2	5	5	3	3
4	4	4	40	3	4	4	3	2
5	5	4	40	2	4	4	4	2
5	5	5	59	1	5	5	4	5
4	2	4	40	3	3	4	4	3
4	2	4	54	1	1	5	4	3
5	5	5	53	5	5	5	5	3
3	3	3	60	3	3	3	3	3
4	4	4	50	2	5	5	4	4

4	5	4	45	1	5	5	5	4
5	5	5	35	1	4	5	5	3
3	1	4	54	3	5	4	2	2
3	3	3	41	3	4	4	3	3
5	5	5	61	3	3	3	3	3
4	5	5	30	5	5	5	4	5
2	4	2	38	1	5	5	5	4
5	5	5	45	1	5	5	4	3
2	4	3	43	3	2	2	3	1
5	2	3	43	3	3	3	1	1
5	4	3	55	1	5	5	4	3
5	5	5	58	1	5	5	4	3
5	5	5	61	2	4	4	4	1
5	5	4	43	2	5	5	4	3
5	5	4	59	1	5	5	4	4
5	5	5	61	1	5	5	5	5
5	5	4	36	2	4	3	3	3
5	5	3	56	3	5	5	4	4
5	5	5	56	1	4	4	4	4
3	4	3	55	2	4	4	3	2
5	5	3	41	3	4	4	4	4
5	5	4	65	1	5	5	4	2
5	3	5	31	3	5	5	5	3
3	3	3	57	3	5	4	3	3
5	5	5	43	1	5	5	5	5
5	5	1	51	5	1	5	1	1
4	5	5	60	1	4	4	4	4
4	3	4	59	3	4	4	2	4
5	5	3	54	5	5	5	5	1
5	5	5	42	1	5	5	5	3
5	5	5	46	1	5	5	5	5
5	5	5	42	2	4	4	4	3
3	3	3	46	1	5	5	4	2
5	5	4	61	2	5	5	5	5
3	3	3	48	2	4	4	4	3
3	3	3	49	3	4	4	4	2
5	5	5	41	1	5	5	5	5
4	4	4	41	2	5	5	4	3
5	5	3	41	1	4	4	3	3
3	4	4	49	2	4	4	4	4
3	3	3	56	2	4	4	4	4
3	3	3	46	3	4	4	3	3
5	4	5	41	2	4	5	4	3
5	5	5	57	2	5	5	4	3
4	4	4	59	2	4	4	3	3
3	3	3	49	3	4	3	2	2
5	5	5	45	2	4	4	4	4
5	5	4	37	1	4	4	4	4
5	5	4	44	1	5	5	4	3
4	4	2	51	2	5	4	3	3
4	5	3	34	2	4	4	1	3
4	3	3	50	1	5	5	4	4
4	5	5	53	2	5	5	4	4
4	3	3	36	3	3	3	1	1

5	5	3	44	1	5	5	4	4
5	4	4	34	2	4	4	5	4
3	4	2	41	2	5	5	5	4
5	5	5	54	2	4	5	4	3
5	5	5	46	1	5	4	4	3
3	3	3	38	2	4	4	2	4
5	5	5	34	2	5	5	5	4
3	5	2	49	2	4	4	4	4
4	4	3	44	2	4	4	2	4
5	5	5	61	4	4	4	3	3
5	5	3	59	2	4	4	3	2
3	4	4	57	2	4	4	4	4
5	5	5	34	4	4	4	4	4
5	5	5	34	2	4	4	3	3
5	4	3	41	2	4	4	4	3
5	5	5	50	4	4	4	4	4
5	5	5	39	1	4	4	4	1
3	4	3	58	1	5	5	4	3
4	4	4	39	2	4	4	4	3
3	3	3	44	2	4	4	4	3
5	5	4	47	2	4	4	4	4
3	3	3	47	1	4	4	3	4
3	5	3	52	1	5	5	4	4
5	5	5	44	1	5	5	3	3
3	5	3	49	2	4	4	2	4
5	5	5	41	1	4	5	5	2
3	3	3	48	1	4	4	3	4
4	4	3	44	2	4	4	2	2
5	5	5	35	1	5	5	4	4
3	3	3	37	3	4	4	2	3
4	4	3	55	2	5	5	3	3
3	5	5	33	2	4	4	4	3
3	3	3	28	2	4	5	4	4
4	4	4	40	3	3	4	2	3
5	4	4	53	2	4	4	4	4
4	3	2	44	2	4	4	2	3
2	3	1	44	3	5	4	2	3
3	3	3	44	2	4	5	3	3
5	5	3	44	4	4	5	4	4
5	5	5	31	2	5	5	5	2
3	3	3	42	2	5	4	3	3
3	3	5	58	3	3	5	4	4
3	3	3	44	3	4	3	2	2
2	2	2	41	3	4	4	2	3
5	3	3	63	3	4	4	3	3
5	5	5	52	3	4	4	4	3
3	3	3	53	1	4	4	2	4
4	3	3	41	1	4	4	4	4
5	5	5	32	1	5	5	5	4
4	4	4	55	2	4	4	4	4
5	5	5	40	1	5	5	5	4
3	3	3	62	3	4	4	4	4
2	3	2	41	3	3	4	5	4
5	4	4	37	1	5	5	4	4

4	4	3	42	2	4	4	4	3
5	5	5	61	1	5	5	5	5
3	4	4	41	2	4	4	2	2
3	3	3	47	3	3	3	3	3
4	4	2	58	2	4	4	2	2
5	5	5	43	1	5	5	5	5
3	3	3	42	2	4	4	3	4
4	5	2	45	1	4	4	4	4
5	5	5	50	2	4	4	4	2
5	5	5	47	3	2	4	2	1
4	4	3	41	2	4	4	4	3
4	4	4	39	1	5	4	3	2
3	4	3	49	2	4	4	4	3
4	5	5	46	2	4	4	3	3
3	3	3	61	1	5	5	3	3
3	3	3	34	1	4	4	3	3
5	5	3	48	1	4	4	3	3
5	4	5	61	3	4	3	2	4
5	5	5	45	1	5	5	5	5
5	5	5	57	1	5	5	5	5
5	4	3	41	5	4	5	4	2
5	5	5	42	2	5	5	5	5
4	4	4	55	4	4	5	4	3
5	5	5	39	2	5	5	5	3
2	2	2	52	3	3	3	1	1
3	3	3	32	3	4	4	2	3
5	5	5	53	1	5	5	5	5
3	3	2	41	1	5	5	3	3
5	5	5	51	1	4	4	4	3
5	5	5	40	1	4	4	2	3
4	5	4	40	1	5	5	4	3
3	3	3	44	3	5	5	3	3
5	5	4	35	1	5	5	5	5
4	5	1	43	3	1	1	1	1
4	4	4	45	2	4	3	2	3
5	5	5	48	3	2	4	3	2
5	5	5	59	2	2	5	2	2
3	3	3	26	2	4	4	3	4
3	3	3	36	1	5	5	5	5
5	5	2	59	3	5	5	2	2
5	5	3	52	1	5	5	4	3
2	2	2	55	2	4	4	4	3
4	4	3	41	3	4	3	2	2
5	5	5	39	2	5	5	2	2
4	4	4	35	4	4	4	4	4
5	5	4	61	1	5	5	4	4
3	3	3	47	1	4	4	4	2
3	3	5	39	2	3	3	2	1
5	5	5	41	1	5	5	4	4
5	5	5	48	2	4	4	4	4
3	3	3	34	1	5	5	3	3
3	3	3	53	3	4	4	3	4
1	3	4	43	3	4	4	2	4
5	5	3	42	2	4	4	4	4

5	3	2	27	1	5	5	2	4
5	5	3	39	3	4	4	3	3
4	4	4	49	2	5	4	3	4
4	3	3	39	2	4	4	3	4
3	3	1	54	3	2	1	1	3
3	3	3	33	1	5	5	3	4
4	4	4	63	1	5	5	4	4
3	3	3	61	3	4	4	3	3
4	4	3	50	1	5	5	4	2
5	5	5	39	2	5	5	5	5
5	5	5	42	2	5	5	5	5
5	5	5	61	1	5	5	5	5
4	4	4	45	1	5	5	4	4
4	4	3	40	3	4	4	2	3
3	3	3	38	2	4	4	4	2
5	5	5	61	2	5	5	5	3
5	5	5	43	1	4	4	4	4
3	3	3	51	4	3	3	4	2
4	3	2	57	2	4	4	3	4
5	5	5	38	1	5	3	4	4
4	4	3	32	2	4	4	3	4
4	4	4	29	2	4	4	4	2
5	5	5	62	2	3	4	3	2
3	3	2	48	1	4	4	3	3
5	4	2	34	2	4	4	2	4
5	3	2	33	2	5	5	1	1
5	5	5	50	1	5	5	5	4
5	5	4	57	2	4	4	3	2
5	5	5	41	5	5	5	5	5
3	3	2	47	4	2	4	2	2
4	4	4	57	3	4	4	3	3
5	5	5	45	1	5	5	5	5
3	3	3	61	2	4	4	3	3
5	4	4	39	2	4	4	4	3
5	5	5	39	1	5	5	5	5
5	4	2	57	3	3	5	3	3
5	5	5	46	1	5	5	5	4
3	3	3	43	2	4	4	3	3
3	3	3	52	4	4	4	2	2
5	5	5	48	1	4	4	4	4
4	4	4	61	3	4	3	2	2
5	5	3	57	2	5	5	2	2
5	5	5	46	2	3	3	2	3
5	3	3	50	2	4	4	4	3
5	5	5	24	1	5	5	5	3
5	5	3	43	1	5	5	4	3
3	5	2	39	1	5	5	5	1
4	4	4	46	2	4	4	4	4
1	3	1	47	3	3	3	2	2
4	4	3	42	3	4	4	3	3
3	3	3	54	2	4	4	4	4
5	5	5	64	3	3	3	3	3
3	4	4	47	3	4	4	3	2
5	3	2	38	3	4	4	2	4

4	4	4	58	2	4	3	4	4
5	5	5	59	4	5	4	4	4
4	4	4	23	2	4	4	2	2
2	4	2	43	3	2	4	2	4
5	5	4	36	2	4	4	4	3
5	5	4	40	2	5	5	5	3
4	1	1	41	3	1	4	1	1
5	5	3	49	2	4	4	1	2
3	3	3	54	3	4	4	2	1
3	3	3	49	3	3	3	3	3
2	4	3	54	4	4	5	3	3
5	5	4	40	2	5	5	4	4
5	5	5	30	3	4	3	2	2
4	5	5	36	2	5	5	3	4
3	3	3	61	2	5	5	3	2
3	3	3	44	1	5	5	4	2
2	2	2	42	2	4	4	2	3
2	2	2	36	2	4	4	3	3
5	5	5	41	1	5	5	4	4
5	3	3	52	1	5	5	3	3
3	3	3	59	3	3	4	2	3
3	3	3	45	3	4	4	4	3
3	3	3	57	4	4	3	3	2
4	4	4	41	2	4	4	4	4
5	5	5	42	1	5	5	5	4
5	5	3	48	1	5	3	3	1
5	5	3	13	2	5	5	4	4
3	3	3	44	2	4	4	4	4
5	3	3	65	1	4	3	3	3
4	4	3	43	2	4	3	3	4
1	1	1	46	1	1	1	1	5
3	5	5	34	1	5	5	5	5
5	5	5	32	2	4	3	3	3
4	4	4	62	4	4	4	4	4
3	3	4	43	2	4	4	4	4
5	5	5	63	2	1	1	1	1
5	5	5	45	1	4	5	5	5
5	5	5	53	1	5	5	5	5
5	5	5	42	1	5	4	4	3
5	5	5	48	1	5	5	5	5
4	4	4	53	5	5	5	5	5
4	4	4	40	2	4	4	3	3
5	5	5	47	1	4	4	4	4
4	4	4	46	2	4	4	4	4
4	4	4	46	3	4	4	4	4
3	4	2	32	3	4	4	3	2
4	4	3	49	3	4	4	4	4
3	4	4	57	1	5	4	3	2
4	2	3	61	2	5	4	4	4
5	5	5	43	1	5	5	5	3
4	4	4	65	2	4	4	4	4
3	5	3	43	3	4	4	4	3
5	5	5	48	1	5	5	5	5
3	3	3	41	1	4	4	4	3

5	5	5	46	5	5	5	5	5
5	5	5	61	1	5	5	5	5
3	3	3	61	1	5	5	5	5
3	4	3	58	2	4	5	4	3
4	3	4	47	1	3	3	5	4
5	5	4	61	1	5	5	5	4
5	5	5	54	3	4	5	3	5
5	5	5	42	1	5	5	4	3
4	3	3	57	1	4	5	3	3
5	5	5	50	1	5	5	5	4
5	5	3	50	1	4	5	5	4
3	3	3	24	2	2	2	1	4
5	5	5	42	2	4	4	4	4
4	4	4	48	2	4	4	4	4
4	4	4	44	2	4	4	4	4
2	2	3	60	2	3	4	2	2
3	3	3	61	2	4	4	2	4
5	5	5	43	1	4	5	4	4
5	5	4	43	2	4	4	3	4
5	5	5	43	2	5	5	3	5
5	5	5	61	1	5	5	5	5
3	3	3	40	3	4	4	2	1
3	3	3	47	4	4	4	4	3
4	4	3	45	1	4	5	4	3
5	5	5	39	1	5	4	4	4
5	5	3	36	3	3	3	2	1
4	4	4	42	2	4	4	3	2
5	5	3	49	1	5	5	3	4
3	3	3	65	1	5	5	4	4
3	3	3	62	4	3	1	1	1
4	4	3	57	2	4	4	4	4
4	5	5	50	2	4	4	3	3
5	5	5	61	1	5	5	5	5
5	5	5	29	1	5	5	5	4
5	5	5	57	4	4	5	1	4
4	4	4	56	1	5	5	5	5
5	5	5	65	1	5	5	5	5
2	2	2	25	1	4	3	4	4
5	5	5	57	1	3	5	5	3
5	5	3	51	4	4	4	4	4
5	5	5	61	5	5	3	5	5
1	1	1	37	1	5	5	5	5
5	4	4	27	3	5	5	5	3
5	5	3	43	2	4	4	4	4
5	5	5	41	1	5	4	4	3
1	4	4	58	1	4	3	3	1
5	3	1	41	1	5	5	1	3
3	3	3	43	1	5	5	5	3
3	3	3	65	2	4	4	4	3
5	5	4	56	1	3	4	3	2
3	3	3	61	3	3	3	3	3
3	3	3	65	2	4	4	3	4
5	5	5	38	2	5	4	4	4
5	5	5	47	1	4	5	4	3

5	5	5	56	1	5	5	5	5
5	5	5	60	1	5	5	5	3
3	3	3	36	3	4	4	4	1
4	5	4	54	1	4	4	4	4
5	5	4	65	3	4	4	3	3
4	5	5	59	1	4	4	4	5
3	1	2	61	2	1	1	3	2
4	5	5	54	2	5	5	4	3
5	5	5	61	5	5	5	4	5
5	5	3	61	1	1	5	4	3
5	5	5	65	1	4	4	4	3
4	5	5	50	2	5	3	2	3
5	5	5	47	1	5	5	5	3
5	5	5	41	1	5	3	3	3
5	5	5	61	2	3	4	4	2
5	5	5	61	1	4	4	3	1
5	5	3	31	1	3	5	3	3
5	5	1	61	1	1	1	1	1
5	5	5	61	2	2	2	2	2
5	5	5	48	2	4	3	4	3
5	3	2	50	2	4	4	4	3
5	5	5	65	1	5	4	4	4
5	5	5	58	1	5	5	5	3
4	3	4	53	1	5	5	4	4
3	3	3	47	3	3	4	2	3
5	5	5	61	5	5	5	5	3
4	5	5	41	5	5	5	3	4
5	5	5	61	3	3	4	4	3
5	5	3	61	1	5	5	3	3
5	5	5	60	2	4	4	4	3
3	2	5	31	3	4	3	3	3
5	5	5	40	1	5	5	5	4
5	5	5	46	2	4	5	4	4
5	5	5	61	1	5	5	4	3
4	5	2	48	2	3	4	1	1
5	4	3	58	3	4	4	3	2
3	4	3	49	1	5	5	3	1
5	5	5	57	5	5	5	5	5
5	5	4	61	4	5	4	4	2
5	5	4	59	1	5	5	5	3
4	3	4	47	2	4	4	4	4
5	5	5	55	5	5	5	5	1
5	5	5	61	2	5	5	3	5
5	5	4	50	1	5	5	5	3
4	4	4	53	3	4	4	4	3
5	5	3	35	3	4	5	4	2
5	5	5	58	1	5	5	4	3
5	5	4	55	1	5	5	4	3
2	3	2	55	3	3	4	2	4
5	5	5	65	1	5	5	5	5
5	5	5	53	3	3	3	3	3
5	5	3	48	1	5	5	4	3
5	5	5	61	1	5	5	5	5
5	5	5	61	2	5	3	3	2

4	4	4	49	3	3	3	3	3
5	5	5	61	1	4	5	4	3
5	5	5	61	2	4	4	5	4
3	5	5	53	3	3	3	3	3
5	5	5	26	5	5	3	5	3
5	5	5	59	1	5	5	5	2
3	3	3	57	3	4	4	2	1
1	2	5	61	2	4	4	2	1
5	5	5	51	2	5	5	5	5
5	5	5	61	5	5	5	5	4
5	5	5	57	1	5	5	5	5
1	4	4	43	1	4	3	4	3
5	5	5	59	1	5	5	5	5
5	5	5	43	5	1	1	1	1
4	4	3	65	1	5	5	3	4
5	5	3	59	3	4	5	5	2
4	4	3	45	1	4	4	3	5
5	5	5	51	1	5	5	4	5
5	5	5	53	1	5	5	5	4
4	4	4	61	3	4	4	4	4
5	4	5	57	1	5	5	5	3
4	4	4	26	3	3	3	3	3
5	5	5	42	1	5	5	5	2
5	5	5	39	1	5	5	5	5
2	2	2	35	2	2	2	2	2
3	3	3	39	2	4	4	4	4
3	4	4	55	2	1	4	5	4
3	3	2	37	3	4	3	3	2
3	3	2	47	2	4	4	4	4
3	4	2	54	2	4	4	3	4
5	5	3	61	2	4	4	3	2
4	5	5	42	2	3	3	4	3
5	5	5	57	1	4	5	4	4
5	3	3	44	3	3	4	3	2
5	5	5	38	3	4	3	3	3
4	4	3	45	1	4	4	4	4
5	5	3	39	3	4	4	3	3
3	4	2	60	2	5	4	4	1
4	3	4	44	2	4	4	4	4
4	4	4	52	2	5	4	4	4
5	5	4	49	2	4	4	2	3
5	5	5	61	2	5	5	5	5
5	5	5	59	2	4	5	4	4
4	2	2	34	3	3	4	2	2
3	3	3	38	3	3	3	2	2
5	5	5	61	1	5	5	5	5
5	4	3	46	3	4	4	3	3
5	5	5	60	2	4	4	4	4
3	4	3	43	2	4	4	4	4
3	3	3	36	3	4	4	2	4
4	4	4	44	1	4	4	4	3
5	5	5	59	1	4	4	4	3
3	3	3	39	3	3	3	3	3
3	3	1	37	3	4	4	3	3

5	3	3	40	1	4	4	3	4
5	5	3	49	3	3	4	3	3
4	3	3	44	2	4	3	3	1
5	5	3	54	1	4	4	4	3
2	2	2	21	3	4	4	2	2
5	5	5	47	1	3	5	2	1
5	5	4	47	2	4	4	3	2
3	3	3	41	2	4	4	3	3
4	5	3	51	1	5	5	3	2
5	5	2	45	5	5	5	5	5
5	5	5	57	2	4	4	4	2
1	1	1	13	1	1	1	1	1
5	5	4	54	2	5	4	4	5
4	4	4	49	2	4	3	2	2
5	5	5	61	1	5	5	5	3
5	5	5	56	3	4	2	1	2
5	5	3	46	2	4	4	4	3
2	4	3	35	3	4	4	2	3
4	3	3	43	2	3	4	2	4
3	4	3	42	4	2	3	2	4
5	5	5	65	3	3	5	5	3
3	3	3	40	3	4	4	3	3
4	3	3	42	3	4	4	4	3
4	5	4	49	2	4	4	4	1
4	4	4	50	2	4	4	4	4
5	5	2	46	2	4	4	3	2
5	5	5	35	2	4	4	2	3
5	1	5	30	2	4	4	2	1
4	4	2	40	1	4	5	2	4
4	4	2	41	2	5	5	2	4
4	5	3	54	3	3	3	3	3
4	4	4	48	2	4	4	4	3
5	5	5	58	1	4	4	4	3
5	5	5	57	1	5	5	5	5
1	1	1	27	2	4	4	2	3
4	5	4	49	2	4	5	4	4
4	4	3	46	2	4	4	3	4
5	5	3	52	2	4	4	2	2
3	3	3	40	3	4	4	2	2
2	2	2	33	2	4	4	3	2
4	4	4	53	1	5	5	5	5
5	4	4	55	1	4	4	4	3
5	5	5	61	5	5	5	5	5
5	4	5	49	2	4	3	3	4
5	5	4	52	1	5	5	4	3
4	4	3	50	3	4	4	3	2
3	4	3	46	2	4	4	3	4
4	5	3	40	3	4	4	2	2
3	2	4	34	3	4	3	2	2
3	4	2	33	1	5	5	2	3
5	5	5	60	2	5	5	4	3
5	5	4	59	1	5	5	5	4
5	5	4	54	2	4	5	4	3
5	5	5	55	1	5	5	5	4

3	4	3	44	2	3	3	3	2
4	3	4	44	2	4	4	4	4
5	5	5	61	3	5	4	4	3
5	5	3	52	1	3	5	4	4
2	3	3	46	3	4	4	4	3
5	3	4	43	1	4	4	4	4
5	5	5	57	1	2	3	2	2
4	5	5	54	2	5	5	4	3
5	5	4	47	1	1	1	4	5
4	4	2	44	2	4	5	3	4
4	4	2	44	1	5	5	5	5
4	4	3	52	2	4	5	4	4
3	3	3	46	1	5	3	3	4
3	3	3	44	2	4	4	4	3
4	5	3	55	1	4	5	5	5
5	5	5	61	1	1	5	4	3
5	5	1	61	1	5	5	5	5
5	5	5	59	3	4	4	4	2
5	5	5	52	1	3	4	2	1
3	4	4	48	3	3	3	2	2
5	5	3	54	1	5	5	3	3
3	4	3	53	3	3	4	3	3
5	5	5	61	1	5	5	5	5
5	5	5	60	1	5	5	5	4
5	5	5	60	1	5	5	5	5
1	2	1	25	3	2	4	1	2
5	5	4	56	1	5	5	5	5
5	5	5	59	1	5	5	5	5
4	4	4	41	1	5	5	3	4
5	5	5	57	1	5	5	5	5
5	4	1	45	1	5	3	5	3
4	5	3	55	1	5	5	5	5
5	5	3	56	3	4	3	3	2
3	5	5	49	1	5	5	5	5
5	5	5	54	1	5	5	3	5
5	5	5	57	2	5	5	4	4
4	3	2	39	2	4	4	4	4
5	4	3	41	3	2	2	2	1
5	5	1	57	3	3	3	3	3
5	5	4	60	1	5	5	4	4
5	5	5	60	1	5	5	5	5
5	5	3	51	3	4	4	3	3
1	1	1	21	3	3	3	3	3
5	5	5	65	3	5	5	4	3
5	5	4	50	2	3	1	3	2
3	4	4	42	2	4	3	3	2
4	3	4	48	1	5	5	4	4
3	4	3	42	2	5	5	3	3
4	4	4	50	1	4	5	4	4
5	4	4	46	2	4	5	3	4
4	5	5	48	2	4	4	2	4
5	5	5	59	1	5	5	5	3
5	5	5	54	2	4	4	4	2
5	5	4	57	1	5	5	5	5

5	4	5	53	2	3	3	4	5
5	3	5	45	3	5	5	2	3
5	4	3	45	3	3	4	2	1
5	5	5	45	5	1	1	1	1
5	4	4	47	1	4	4	3	3
3	3	1	31	3	2	2	2	1
3	3	3	34	2	4	4	3	3
5	5	5	53	1	4	4	4	2
4	5	5	54	1	4	4	4	4
2	2	3	41	3	4	3	2	1
4	3	3	51	2	5	5	4	3
3	3	5	43	2	4	5	3	1
4	5	4	46	2	4	3	4	2
5	5	4	58	1	5	5	5	4
5	5	4	58	1	5	5	5	5
3	3	3	45	3	3	3	4	3
4	4	4	47	2	4	4	4	2
5	5	3	49	2	4	5	4	4
5	5	4	47	3	4	4	3	2
5	5	5	59	2	4	4	3	4
4	3	4	43	2	4	4	3	3
4	3	3	28	4	3	3	2	2
5	5	5	65	5	5	5	5	5
5	5	5	59	1	5	5	5	4
4	5	3	49	3	5	5	4	4
4	4	3	45	3	3	2	3	2
3	3	3	37	3	3	3	2	3
5	5	4	55	1	5	5	4	4
2	2	2	26	3	3	3	3	3
4	4	4	48	2	4	4	4	3
3	3	3	58	2	4	4	3	3
4	3	3	41	4	2	3	2	1
5	5	3	40	3	3	5	3	1
5	5	5	57	2	5	4	3	3
5	3	4	51	3	4	4	3	3
4	4	4	48	2	4	4	4	3
5	5	3	50	1	5	4	4	3
5	5	4	57	3	4	4	4	3
4	4	3	54	2	4	4	3	2
5	5	5	46	3	5	5	4	2
4	4	4	60	1	4	4	4	4
5	5	5	50	4	5	4	4	3
4	3	4	61	2	4	4	3	3
5	5	4	41	2	4	4	4	4
5	3	5	53	3	2	3	2	2
5	5	5	35	2	5	5	2	4
5	5	5	61	2	5	5	5	4
4	4	4	61	2	4	5	4	4
4	4	4	48	2	4	4	5	4
4	5	5	51	1	3	5	5	4
5	5	5	55	1	4	4	5	4
2	3	3	57	1	4	4	3	4
5	4	1	36	1	5	5	5	5
3	3	2	47	2	4	4	3	3

5	5	5	54	3	5	5	4	3
4	4	4	54	1	4	4	4	4
3	3	4	50	2	4	4	4	4
1	1	1	44	1	5	5	5	5
5	5	5	13	1	5	5	5	5
3	3	2	60	2	4	4	3	3

C6	C7	C8	C9	C10	C11	C12	C13	C14
5	5	5	5	5	5	5	5	5
3	4	4	4	3	4	1	3	3
5	3	5	5	5	5	5	5	5
4	3	2	3	2	4	4	4	4
4	4	3	3	3	3	3	2	3
5	5	5	5	5	5	5	5	5
2	4	1	2	5	4	2	2	2
4	4	1	4	2	4	2	3	4
5	1	5	2	4	3	3	3	3
5	1	5	5	5	5	5	5	5
5	2	1	1	1	5	3	3	5
4	3	2	2	2	4	3	2	4
5	1	2	2	1	4	4	5	5
1	5	5	5	5	5	5	5	5
4	3	2	5	5	5	4	3	4
5	5	5	5	5	5	5	5	5
4	3	2	2	2	4	3	2	4
5	5	5	5	5	5	1	5	5
3	4	2	2	2	4	2	2	4
3	4	2	3	2	5	3	2	3
4	4	5	5	5	4	5	3	3
5	5	5	5	5	5	5	5	5
5	1	1	1	1	5	5	5	5
5	1	1	1	1	5	5	5	5
2	4	2	2	2	4	2	2	2
3	4	2	2	1	4	3	2	3
3	3	1	3	2	4	3	3	3
4	2	3	2	2	4	4	4	4
4	1	1	1	1	5	4	2	4
4	3	2	3	2	4	2	2	4
5	2	2	2	1	5	5	5	5
5	1	1	1	1	5	5	5	5
5	1	2	2	1	4	4	4	4
5	2	3	4	1	5	4	3	5
3	2	1	1	1	4	3	2	4
4	3	1	3	1	4	4	4	5
5	1	1	1	5	5	5	5	5
5	5	5	5	5	5	5	5	5
4	3	2	2	1	5	5	5	5
5	1	1	2	2	2	5	5	5
4	1	1	1	1	4	4	4	4
4	1	2	2	2	4	4	4	4
4	1	1	1	1	4	4	4	4
4	2	2	2	1	3	4	3	4
4	2	2	2	2	4	4	2	4
4	4	4	4	4	4	4	4	4
4	2	2	2	2	4	4	4	4
5	5	5	5	5	5	5	5	5
3	3	3	3	3	3	3	3	3
1	4	5	5	5	5	1	5	5
4	3	1	4	2	5	5	5	5
5	1	3	1	1	5	5	5	5
5	3	3	3	4	4	4	3	4

4	4	2	4	2	4	3	3	4
4	2	2	2	1	3	3	4	4
5	5	5	5	5	4	5	4	4
5	5	5	4	5	5	5	4	5
5	2	5	3	3	5	5	5	5
4	2	1	1	1	4	3	3	4
5	1	1	1	1	5	5	5	5
5	1	5	1	1	5	5	5	5
5	2	2	2	1	5	3	2	4
4	4	2	1	2	3	3	3	3
4	2	2	2	2	5	3	3	4
5	2	4	2	4	4	4	4	4
5	1	5	1	1	5	5	5	5
4	1	5	5	1	1	3	3	2
5	2	2	1	1	4	4	4	4
5	3	1	2	1	5	3	3	5
5	1	1	1	1	5	5	5	5
5	2	2	2	2	5	5	5	5
5	2	2	2	2	4	4	2	4
4	2	3	2	1	5	3	3	4
5	2	4	2	1	5	4	3	4
5	3	1	1	1	5	3	4	4
1	5	3	1	5	5	5	5	5
4	4	4	4	3	4	3	3	4
1	1	5	3	3	3	3	3	2
4	2	5	1	1	4	1	4	2
4	1	1	1	1	4	4	4	4
3	3	3	2	1	3	3	3	5
3	3	3	3	3	3	3	3	3
3	3	3	3	3	3	3	3	3
3	3	3	3	3	3	2	3	3
3	1	1	2	1	4	3	1	3
5	1	2	2	1	5	5	5	5
3	3	3	3	2	2	2	2	2
3	2	2	2	1	5	3	3	5
5	1	4	1	1	5	5	5	5
5	1	1	1	1	5	4	4	5
3	2	1	1	1	3	2	2	3
4	2	4	3	2	4	1	2	3
5	1	1	1	1	5	5	3	5
2	3	4	2	2	4	3	2	3
4	2	2	2	2	4	4	4	4
3	4	3	2	1	3	3	1	1
5	1	1	2	1	5	5	5	5
2	4	4	2	1	5	3	3	4
4	3	3	2	2	1	5	1	3
4	4	4	4	4	4	4	4	4
3	3	1	1	1	5	3	2	3
5	1	5	1	1	5	5	5	5
4	1	2	4	2	5	2	1	5
4	3	3	4	2	4	2	2	4
4	2	2	3	2	4	4	4	4
2	2	2	2	2	2	2	2	2
2	4	4	4	2	3	2	2	3

3	3	3	3	3	3	3	3	3
2	4	3	2	4	4	2	1	1
4	4	2	4	1	4	4	1	5
5	3	1	1	1	5	5	5	5
5	2	1	1	1	5	5	5	5
5	3	2	2	1	5	2	1	5
4	4	3	3	2	4	2	2	3
3	2	4	1	1	5	3	5	4
5	1	1	1	1	5	4	4	5
3	3	3	3	3	3	3	3	3
3	3	3	3	3	3	3	3	3
5	2	1	1	1	5	4	1	4
5	2	1	1	1	5	5	3	5
4	2	2	5	2	4	3	3	5
4	1	1	1	1	5	4	4	4
4	2	2	2	2	4	4	3	4
4	3	2	1	1	4	4	5	5
5	2	2	2	2	5	2	2	5
3	2	1	1	2	4	3	2	3
4	2	4	4	1	4	4	4	4
4	3	2	1	1	4	4	4	4
5	1	1	1	1	5	5	5	5
3	3	3	3	3	3	3	3	3
4	3	4	3	3	3	3	3	3
2	2	2	2	2	4	2	4	4
3	4	3	3	2	3	1	2	3
2	3	3	2	3	2	2	3	2
3	3	3	3	2	4	2	2	2
4	5	4	2	1	4	1	1	4
5	2	2	1	1	5	4	4	5
4	2	2	2	2	4	4	4	4
5	3	2	2	2	5	2	2	5
4	4	1	3	3	3	3	3	3
1	3	1	5	5	1	1	1	1
3	3	4	2	3	5	4	5	5
5	2	2	1	1	5	4	4	4
3	3	3	3	3	3	5	3	4
5	1	1	1	1	5	5	2	5
4	1	1	1	1	4	2	1	4
3	3	1	3	2	3	1	1	5
5	1	1	1	1	4	4	4	5
1	5	3	4	5	2	1	1	1
3	3	3	3	3	1	2	3	3
4	4	4	3	2	4	2	2	3
5	3	1	1	1	5	5	1	5
5	2	4	2	2	5	2	2	5
2	4	1	2	2	2	1	1	1
5	3	2	2	2	5	3	3	5
4	2	2	2	2	4	4	4	4
3	4	3	2	1	4	2	2	4
5	2	2	1	1	5	5	3	4
5	5	5	1	1	5	3	3	5
4	5	3	1	2	4	2	3	4
4	2	2	3	2	4	4	3	4

5	1	1	1	1	5	3	3	4
3	2	2	3	1	4	3	2	3
5	1	1	2	1	5	4	4	5
4	3	3	3	2	4	3	4	3
5	2	2	1	1	5	4	4	5
4	4	4	4	2	4	4	4	4
4	3	2	2	1	4	3	3	4
4	2	2	2	2	4	4	4	4
2	4	4	4	3	3	3	3	3
4	3	2	1	1	4	3	4	4
4	2	2	2	2	4	3	4	4
5	3	2	1	1	5	4	4	5
3	3	3	3	2	3	3	3	3
5	1	1	1	1	5	5	5	5
3	3	2	2	2	3	3	3	3
4	4	3	1	1	5	2	3	4
5	2	2	3	1	5	4	4	4
4	2	1	1	1	4	3	2	4
4	4	1	3	3	3	3	3	3
5	1	1	1	1	5	4	5	5
4	1	2	1	1	5	2	3	4
5	2	2	2	1	5	5	4	5
4	2	1	1	1	4	4	1	3
5	2	1	1	1	5	5	5	5
1	5	3	1	1	1	1	1	1
4	2	3	1	1	5	3	4	3
4	2	2	3	2	4	2	4	4
4	3	1	1	2	2	4	3	3
1	5	1	5	4	4	1	1	1
5	1	1	1	1	5	5	2	5
3	4	3	2	2	4	3	4	3
5	1	1	1	1	5	5	5	5
5	1	1	1	1	5	5	3	5
5	3	1	1	1	5	3	4	5
4	3	3	3	3	3	3	1	1
4	2	2	2	1	2	3	5	2
4	3	3	3	2	2	3	4	4
5	2	2	1	2	5	5	4	5
4	3	4	3	2	4	3	3	4
5	4	4	2	1	5	5	5	5
5	4	3	3	1	5	4	3	5
5	2	4	2	2	4	4	4	4
4	2	3	2	1	5	2	4	4
4	2	3	2	1	5	3	3	4
4	4	3	3	2	4	3	3	4
3	3	2	1	2	4	3	3	3
3	3	3	3	3	3	3	3	3
5	1	1	1	2	5	5	5	5
5	5	1	1	1	5	4	5	5
5	1	1	1	1	5	5	5	5
5	2	2	1	1	3	5	4	5
4	1	1	1	1	4	4	1	4
3	3	3	3	3	3	3	3	3
5	4	4	1	1	4	2	3	5

5	3	3	3	3	5	5	3	5
5	3	4	2	1	5	5	5	5
5	1	4	1	1	4	3	5	5
4	3	2	1	1	5	3	2	4
3	4	3	2	1	5	3	4	2
5	5	5	5	5	5	5	5	5
4	4	4	4	4	4	4	4	4
5	1	1	1	1	5	4	4	4
4	4	2	2	2	4	2	2	3
5	2	2	2	2	5	2	5	4
3	3	3	3	3	3	3	3	3
5	1	1	2	1	5	3	5	5
1	1	1	1	1	1	1	1	1
4	3	3	1	1	4	4	2	5
4	3	4	3	1	2	3	2	4
5	3	5	3	2	5	5	5	5
5	4	4	2	3	3	3	3	3
4	1	1	3	2	4	4	3	4
4	3	4	2	2	5	3	3	3
4	1	1	1	1	4	4	4	4
4	4	4	4	3	2	4	4	5
4	4	2	2	2	4	4	4	4
5	2	2	2	2	5	5	5	5
5	5	1	1	1	5	5	5	5
3	3	3	3	2	4	3	3	4
4	4	4	2	2	4	2	2	3
3	3	2	3	2	4	3	3	3
5	3	3	1	1	1	5	3	5
4	5	5	5	5	5	4	4	5
5	1	1	1	1	5	2	4	5
3	3	3	3	3	3	3	3	3
1	5	4	4	4	3	3	1	1
2	4	1	3	3	2	2	2	2
5	5	5	5	5	5	5	5	5
4	2	3	1	1	4	3	3	4
4	3	2	2	2	5	3	3	4
3	2	1	1	1	5	3	3	3
5	2	3	1	1	5	4	1	4
4	4	4	4	4	4	4	4	4
5	5	5	3	3	5	4	5	5
4	2	2	2	1	4	4	4	4
4	3	3	3	1	3	3	3	3
3	4	1	4	1	4	3	4	5
5	3	1	1	1	5	4	2	5
5	3	3	3	3	5	5	3	5
5	2	3	2	1	5	3	3	4
3	4	3	3	2	4	3	3	3
4	3	2	2	2	4	3	3	4
4	3	2	1	1	5	4	3	5
4	2	2	1	1	4	4	3	4
4	4	3	3	3	4	3	4	4
5	1	4	1	1	5	5	4	5
3	3	3	3	3	3	3	3	3
4	3	2	3	2	4	2	2	4

5	3	1	2	1	5	5	4	5
4	1	1	1	1	4	4	3	4
3	4	3	4	1	5	2	2	4
3	3	3	1	3	4	3	3	3
3	3	3	3	3	3	3	3	3
5	1	5	2	1	5	5	5	5
4	1	1	1	1	5	5	5	5
4	4	3	3	1	1	4	4	4
4	3	1	4	1	5	3	2	4
2	5	3	1	3	3	1	1	1
3	1	1	3	1	4	3	1	5
3	2	4	1	1	5	4	4	2
2	2	2	2	2	4	2	2	2
5	3	2	2	1	4	3	4	5
5	1	1	1	1	5	3	2	5
5	2	2	2	2	5	5	5	5
3	3	3	3	3	3	3	3	3
5	4	2	1	1	5	4	4	5
5	1	1	1	1	4	5	4	5
3	4	3	3	1	4	2	2	3
4	3	3	4	4	5	4	4	4
4	3	2	2	1	4	3	4	4
5	5	2	3	1	1	4	4	4
3	2	2	2	2	4	3	3	2
5	1	2	1	3	5	5	5	5
5	5	1	5	1	5	1	1	4
4	2	3	3	2	5	4	3	4
4	4	3	2	2	4	3	3	4
5	1	5	1	5	5	5	3	5
5	1	1	1	1	5	4	2	5
5	1	1	1	1	5	5	4	5
4	3	2	2	1	4	3	2	4
1	3	3	2	2	4	2	2	1
5	5	5	5	5	5	5	5	4
3	3	3	2	2	4	3	3	3
4	3	2	1	1	4	3	3	4
5	3	3	1	1	5	5	5	5
5	2	2	2	1	5	4	3	4
4	2	2	4	4	5	3	4	4
4	4	4	3	4	3	3	4	4
4	2	2	2	2	4	4	4	4
3	4	3	4	3	4	3	3	3
4	4	2	2	2	4	3	3	4
4	4	2	2	1	5	3	3	5
4	4	2	2	2	4	3	3	4
3	3	2	4	3	4	2	2	2
4	2	3	4	4	4	3	4	4
4	2	1	2	1	4	4	4	4
5	1	1	1	1	5	2	2	5
3	5	2	2	1	4	2	2	4
4	4	2	3	2	4	2	1	4
4	2	2	2	1	5	4	4	4
4	3	2	4	2	4	4	2	4
2	4	2	4	3	3	2	2	2

4	3	2	1	1	4	4	5	4
4	4	2	2	2	4	4	4	4
4	3	2	3	2	4	4	4	4
5	2	4	1	1	5	4	4	5
4	5	5	5	2	4	4	4	4
4	4	2	4	2	4	2	2	2
4	3	2	2	1	4	4	3	4
3	4	2	2	2	4	2	2	4
4	4	2	2	2	4	2	2	4
4	3	4	3	3	4	3	4	4
4	4	1	1	1	4	3	2	4
4	2	2	2	2	4	4	4	4
4	4	4	4	4	4	4	4	4
4	4	2	2	2	4	4	4	4
4	2	4	2	1	4	4	4	4
4	4	4	4	4	4	4	4	4
4	4	4	4	4	4	4	4	4
4	2	2	3	1	4	4	3	5
4	3	2	2	1	4	3	3	4
4	3	3	3	3	3	3	3	3
4	4	2	2	1	4	4	4	4
4	4	4	2	2	4	2	3	3
5	3	1	3	1	5	4	3	5
4	3	2	2	2	4	3	3	3
4	4	3	2	2	4	3	4	4
5	1	1	2	1	5	5	5	4
4	2	1	2	2	4	3	4	4
3	4	2	2	2	4	2	2	4
5	3	1	1	1	5	2	2	5
4	3	3	2	2	4	3	4	4
5	4	3	2	1	5	2	4	4
4	4	4	4	2	4	3	4	4
4	4	2	2	2	4	3	3	4
4	3	1	3	2	4	2	2	4
4	2	4	2	2	4	4	4	4
3	3	1	1	1	4	2	2	2
3	4	2	2	1	3	2	2	3
4	5	3	3	3	3	3	4	4
4	2	2	2	2	5	2	2	4
5	2	1	1	1	5	3	3	5
3	4	3	3	1	4	3	3	3
3	4	4	4	2	4	4	4	5
2	3	2	3	2	3	3	2	3
3	3	3	3	1	4	3	2	4
4	4	4	3	1	5	2	3	4
4	3	3	2	2	4	4	4	4
4	4	2	2	2	5	2	2	5
4	1	1	1	1	4	4	4	4
5	2	1	2	1	5	4	4	4
4	4	4	2	2	4	4	4	4
5	1	1	2	2	5	4	3	5
4	2	2	4	4	4	4	4	4
4	3	4	4	4	4	4	5	4
4	4	4	4	1	4	4	4	4

4	2	2	2	2	4	4	2	4
5	4	4	1	1	4	4	4	4
3	4	3	4	3	4	4	2	4
3	3	1	3	3	3	3	1	3
3	4	4	2	2	4	2	2	3
5	2	4	2	2	5	5	5	5
4	2	2	2	2	4	3	4	4
2	4	1	1	1	2	4	1	1
4	2	3	2	2	4	4	2	4
4	5	4	4	4	4	1	1	4
4	4	3	2	3	4	3	4	4
3	5	2	1	2	3	3	4	3
4	4	3	4	2	4	3	3	4
4	4	2	1	1	4	2	2	3
5	3	1	1	1	3	3	1	4
3	3	2	2	1	4	3	1	3
4	4	1	1	1	4	2	4	4
2	4	1	2	3	3	3	4	3
5	1	5	1	1	5	5	5	5
5	1	3	1	1	5	5	3	5
5	4	3	2	2	4	4	4	4
5	2	2	1	1	3	5	5	5
5	4	4	4	2	5	4	4	4
5	1	1	1	1	5	5	4	5
1	5	2	4	1	3	1	1	1
3	4	2	2	2	4	3	3	3
5	1	2	1	1	5	4	4	4
3	5	1	1	1	5	3	5	3
4	4	4	4	2	4	3	3	4
2	3	2	2	1	5	3	4	4
5	1	1	1	1	4	4	4	4
3	3	3	3	3	3	3	3	3
5	2	2	2	1	5	4	5	4
1	1	1	5	3	1	1	1	1
3	3	2	2	1	4	2	2	3
4	5	1	1	1	5	2	2	4
3	3	2	2	1	5	2	2	2
4	4	2	3	2	4	3	2	4
5	5	1	1	1	5	5	5	5
3	5	2	2	1	3	2	2	2
5	2	1	1	1	5	3	2	5
4	3	3	3	2	4	3	4	4
2	4	2	4	3	4	2	2	2
2	4	4	4	1	5	2	5	2
4	4	4	4	4	4	4	4	4
4	2	1	2	1	4	4	3	4
4	2	1	1	1	4	4	4	4
2	4	3	4	3	3	1	1	3
4	1	1	1	1	5	5	4	5
4	3	2	2	2	4	4	4	4
5	5	1	3	1	5	3	1	5
3	5	2	5	2	4	3	3	3
4	4	2	3	3	4	2	2	2
4	2	2	3	2	4	3	3	4

4	4	2	3	1	4	1	1	4
3	3	3	2	3	4	2	2	3
4	3	1	2	2	5	2	4	4
4	4	2	2	2	4	3	3	4
1	1	5	5	3	2	1	5	2
5	4	1	3	1	5	2	2	5
4	2	2	2	1	5	4	4	4
3	4	2	3	2	3	2	2	3
4	1	4	4	1	5	5	5	5
5	3	3	2	1	5	5	5	5
5	3	3	4	2	4	4	4	4
5	5	1	1	1	5	5	5	5
4	3	1	1	1	5	4	4	5
3	3	2	3	2	4	3	2	3
4	2	2	2	2	4	4	2	4
5	1	1	1	1	5	5	5	5
4	3	3	2	2	4	4	4	4
4	2	2	3	3	4	3	4	4
5	4	2	2	1	5	3	4	5
3	4	1	2	2	3	2	2	2
4	4	2	3	2	4	2	2	3
4	4	2	2	2	4	4	4	4
4	3	2	2	2	4	2	3	3
4	4	4	2	2	4	2	2	3
4	4	2	2	1	4	2	3	4
5	5	1	1	1	5	1	1	5
5	3	1	1	1	4	5	5	5
3	3	4	3	2	4	2	2	3
5	5	5	5	5	5	5	5	5
4	4	2	4	2	4	2	2	4
3	3	3	3	3	4	3	3	3
5	2	2	1	1	5	5	5	5
3	5	3	3	2	4	2	2	2
4	3	2	2	2	4	2	4	4
5	1	1	1	1	5	5	5	5
3	3	3	2	1	5	3	3	4
4	3	2	1	1	4	5	2	5
4	3	3	3	3	4	4	4	4
3	4	4	4	4	4	2	2	4
4	4	2	2	1	4	4	4	4
3	4	2	3	3	3	2	2	3
5	4	4	3	1	5	2	4	4
3	4	3	2	2	4	2	2	2
4	2	2	3	1	1	3	2	4
5	1	1	1	1	5	5	4	5
5	2	2	2	2	5	5	4	4
3	1	5	1	3	3	2	3	3
4	2	2	2	2	4	4	4	4
3	3	3	4	2	4	2	2	3
3	3	3	2	2	3	3	3	3
4	2	2	2	2	4	4	4	4
3	3	3	3	3	3	3	3	3
4	4	3	4	3	4	3	3	4
4	3	2	3	2	4	4	3	4

4	2	2	2	2	4	4	4	4
4	5	5	5	5	5	5	5	5
4	3	4	4	2	4	4	3	2
2	4	2	4	2	2	4	4	2
4	3	2	2	2	4	3	3	4
4	2	2	2	2	3	5	3	5
1	4	4	4	1	4	1	1	1
2	2	2	3	2	4	2	2	2
3	3	2	4	3	4	1	1	3
3	4	4	3	1	4	1	1	2
4	5	3	2	1	5	3	4	4
4	2	2	2	1	4	3	4	4
3	4	3	3	3	3	1	1	3
4	1	1	3	1	5	2	3	4
5	2	1	1	1	5	4	5	4
5	1	1	2	1	5	4	4	5
3	4	2	3	1	4	2	2	3
4	3	2	2	1	1	2	3	3
5	1	1	1	1	3	4	3	4
4	4	1	2	1	5	2	2	5
3	3	4	2	2	4	2	2	3
4	4	4	4	4	4	4	4	4
3	2	1	3	3	3	3	3	3
4	2	2	3	2	4	3	3	3
5	2	4	2	1	5	5	5	5
4	3	1	4	1	3	1	1	5
5	5	2	3	2	4	3	3	4
4	2	2	2	2	4	4	3	4
3	3	2	1	1	4	3	3	3
3	2	2	2	1	4	3	4	3
5	4	2	2	3	5	2	5	5
5	2	2	2	1	5	3	4	5
3	3	3	2	2	3	3	3	3
4	4	4	4	4	4	4	4	4
2	2	2	2	2	4	4	4	4
1	5	3	3	3	3	3	3	3
5	1	1	1	1	5	5	5	5
5	2	1	1	1	5	5	5	5
4	4	4	4	4	4	4	4	4
5	1	5	1	1	5	4	4	5
5	5	4	4	4	4	4	4	4
4	4	2	5	3	4	3	3	4
3	3	2	3	2	4	4	2	4
4	3	2	2	2	4	4	4	4
4	4	4	4	4	4	4	4	4
3	4	2	3	2	4	2	2	3
4	3	3	2	2	4	4	4	4
4	3	2	3	2	4	4	4	4
4	2	4	2	2	2	4	4	4
4	2	4	3	2	4	4	4	4
4	2	2	2	2	4	4	4	3
3	4	4	2	2	4	3	4	3
5	2	2	1	1	5	5	5	5
4	2	2	2	2	2	5	2	4

5	5	5	5	1	1	5	5	5
5	3	5	2	2	5	5	5	5
5	2	1	1	1	4	4	4	4
4	3	4	4	2	5	3	3	4
4	5	1	2	1	3	1	4	2
5	1	2	1	1	3	4	4	5
5	4	2	2	1	5	2	4	4
5	2	2	2	2	5	4	4	5
4	2	2	3	4	5	3	1	1
4	2	1	1	1	3	4	4	4
5	2	1	1	1	5	5	3	4
2	2	2	1	1	1	1	1	2
4	3	2	2	2	4	3	4	4
4	3	2	2	2	3	4	4	4
4	4	4	4	4	4	4	4	4
3	4	2	3	2	4	2	2	2
3	4	2	2	2	4	2	2	4
4	2	1	1	1	4	3	2	4
4	4	1	1	1	4	3	4	4
5	3	1	1	1	5	3	5	5
5	1	1	1	1	5	5	5	5
1	3	3	1	1	4	1	1	3
4	4	4	2	4	3	3	3	3
5	4	3	2	1	4	3	4	5
1	1	1	5	5	5	5	5	5
3	3	3	3	3	3	3	3	3
3	3	2	2	2	3	3	3	3
3	3	1	2	1	4	3	3	4
4	2	4	2	2	5	4	4	4
1	4	4	5	5	1	1	1	1
4	2	2	2	1	4	4	3	4
4	3	2	3	1	5	2	3	5
5	1	5	1	1	5	5	5	5
3	1	1	2	2	5	4	4	3
5	2	2	1	2	4	5	5	4
5	1	5	1	1	5	5	5	5
5	3	1	1	1	5	5	4	5
4	2	2	2	2	4	4	4	4
5	1	1	1	1	5	1	3	5
4	3	2	2	2	4	4	4	4
5	5	5	5	5	5	5	5	5
5	1	5	1	1	5	5	5	5
4	2	3	1	1	4	4	3	4
4	2	2	2	2	4	4	4	4
4	2	2	1	1	5	4	4	4
3	4	2	1	1	4	4	4	4
5	5	1	1	1	5	1	1	5
3	3	3	1	1	3	3	3	3
4	4	2	2	2	4	4	4	4
4	2	1	3	3	4	4	3	5
3	4	3	3	3	3	3	3	3
4	2	2	2	2	4	3	2	4
4	2	4	2	2	4	4	4	4
5	2	2	1	1	5	5	5	5

5	1	2	2	1	5	5	5	5
5	1	3	3	2	2	5	5	5
3	3	2	2	2	4	2	3	4
4	2	2	2	2	4	4	4	4
2	5	3	2	2	3	2	2	2
4	2	4	1	2	5	3	4	5
4	3	1	2	1	4	2	3	4
5	2	3	1	1	5	5	5	5
5	5	5	5	5	5	5	5	5
4	1	1	1	1	1	5	4	5
4	4	2	2	2	4	4	4	4
3	3	4	4	2	3	2	5	3
5	1	1	1	1	5	5	5	5
3	3	3	3	3	3	3	3	3
3	2	2	2	2	3	3	4	4
4	2	4	1	1	5	5	5	4
3	1	2	1	1	5	3	3	4
1	1	1	1	1	2	1	1	1
2	2	2	3	4	3	3	3	2
4	2	2	2	3	3	4	4	4
3	2	2	2	1	4	3	2	3
4	2	1	2	1	4	4	4	4
5	3	1	1	1	5	5	3	5
5	1	1	1	1	4	4	5	4
4	4	3	1	2	3	3	3	3
5	1	3	1	1	3	5	5	5
5	1	1	1	1	5	4	3	5
4	2	2	3	2	3	2	2	4
5	3	1	2	1	4	3	3	5
4	2	2	4	2	3	4	3	4
3	3	3	3	3	3	3	3	3
5	1	1	1	1	4	4	5	4
4	1	2	1	1	4	4	4	4
5	2	1	1	1	3	4	4	5
4	4	4	1	1	4	1	2	4
4	3	3	3	3	4	3	2	3
3	1	2	3	1	3	3	3	4
5	2	4	1	1	1	5	5	5
3	3	1	1	1	2	4	2	4
4	3	2	1	1	4	4	4	4
4	2	2	2	2	3	4	3	4
5	1	1	1	1	1	5	5	5
5	5	4	1	1	5	3	3	5
5	1	1	1	1	5	5	5	5
3	3	3	3	3	3	3	3	3
5	1	2	2	2	2	5	5	5
5	2	1	1	1	5	4	3	5
5	2	1	3	1	5	4	1	4
4	4	3	2	3	4	2	2	4
5	1	1	1	1	5	4	4	5
3	3	3	3	3	3	3	3	3
5	2	3	1	1	2	5	4	5
5	1	1	1	1	5	5	5	5
3	4	2	3	2	3	2	2	4

3	3	3	3	3	3	3	3	3
5	3	3	1	1	3	4	4	5
4	1	1	2	2	4	5	4	4
3	3	3	3	3	3	3	3	3
4	1	3	4	4	2	2	4	3
5	1	1	1	1	5	4	4	5
2	5	3	5	1	5	5	5	5
4	2	3	2	2	2	2	2	4
5	1	1	2	2	2	5	5	4
5	1	5	1	1	5	5	5	5
5	1	5	1	1	5	5	5	5
3	4	2	3	2	4	4	2	4
5	1	1	1	1	4	5	5	5
1	1	1	5	5	1	1	1	1
5	3	2	2	1	4	3	2	4
5	4	1	2	5	5	2	5	4
4	2	2	2	1	5	3	3	4
5	1	1	1	1	4	5	4	5
4	2	3	2	2	3	4	3	4
3	3	2	2	2	3	3	4	3
4	2	1	3	1	1	3	2	4
3	3	3	3	3	3	3	3	3
5	1	2	1	1	1	5	5	5
5	1	5	1	1	5	5	5	5
2	2	2	2	2	2	2	2	2
4	4	3	3	2	4	4	2	4
4	1	3	2	3	4	5	5	2
4	2	2	3	2	4	2	2	4
4	3	3	3	2	3	4	4	4
4	3	2	2	2	4	3	3	4
2	2	2	2	2	4	2	4	4
3	2	4	2	2	4	4	3	3
4	4	3	2	1	4	3	4	4
3	4	3	3	2	4	2	2	4
3	4	3	2	2	3	3	3	3
4	2	2	2	2	4	4	2	4
3	4	3	2	2	4	3	2	3
4	4	2	2	2	4	4	4	4
4	3	3	2	2	2	4	4	4
4	3	4	2	1	4	3	3	4
2	4	2	5	2	5	2	1	3
5	2	2	2	2	5	5	3	5
4	2	3	1	1	5	4	4	4
3	3	2	4	2	4	2	3	3
3	4	3	3	3	3	2	2	3
5	1	5	1	1	5	5	5	5
3	3	4	4	3	3	2	3	3
4	2	2	2	2	4	4	4	4
4	2	2	3	2	4	4	3	4
4	4	3	2	1	4	2	3	4
4	2	2	2	2	2	4	4	4
4	2	2	4	2	4	3	2	4
3	3	3	3	3	3	3	3	3
4	4	3	2	1	5	3	4	4

5	3	2	1	1	4	3	2	4
5	4	2	3	1	5	3	3	4
3	4	1	3	1	3	2	4	3
4	4	2	2	2	3	4	4	4
2	4	4	4	2	4	2	2	3
5	3	1	1	1	5	5	1	5
2	3	1	2	2	3	2	2	4
3	2	2	2	2	4	3	2	3
5	2	1	1	1	5	4	4	5
5	3	3	3	3	5	5	5	5
4	2	2	2	2	4	4	4	4
1	1	1	1	1	1	1	1	1
4	2	2	2	2	5	4	4	4
3	4	3	3	2	3	2	2	3
5	3	1	1	1	1	4	5	5
3	3	3	3	3	3	3	3	4
4	4	2	1	1	4	3	4	4
2	3	2	2	2	3	1	2	1
4	2	3	4	2	4	3	2	4
3	4	1	4	2	4	2	1	2
5	5	5	5	5	4	3	3	3
3	3	3	3	3	3	3	3	3
3	3	2	2	2	3	3	3	4
1	1	1	2	2	4	3	3	4
4	3	4	3	2	4	4	4	4
4	4	1	3	1	4	3	3	5
3	4	2	3	2	4	2	4	4
4	4	1	1	1	4	1	1	4
4	4	2	2	2	4	3	2	2
5	4	2	2	2	4	2	2	4
5	3	1	1	3	5	4	2	5
4	3	3	2	2	4	3	4	4
4	3	2	2	2	4	4	2	4
5	1	5	1	1	5	5	5	5
4	4	2	4	2	4	2	4	4
4	2	2	2	2	4	3	2	4
4	4	2	3	2	4	3	4	4
3	4	2	4	2	4	3	4	4
4	2	2	2	2	4	2	2	4
3	4	4	4	3	4	2	2	4
5	2	2	3	1	5	5	3	5
4	2	2	2	2	3	4	3	4
5	5	2	2	2	4	4	4	4
3	3	2	2	1	4	3	3	3
4	2	3	2	2	4	4	4	4
4	3	2	2	2	4	2	2	4
4	3	3	3	2	4	3	3	4
4	3	2	3	2	4	2	2	3
4	4	3	3	2	4	4	1	4
4	4	2	2	2	2	2	2	2
4	2	2	2	2	3	3	3	4
5	1	2	1	1	5	5	4	5
5	1	2	2	1	4	4	3	5
5	2	1	1	1	5	4	4	4

3	3	2	2	2	3	3	3	3
4	2	3	3	2	4	4	4	4
4	3	2	2	1	4	3	3	4
4	3	3	2	2	4	4	4	4
4	4	2	2	2	4	3	2	4
4	2	2	2	1	4	4	3	4
2	1	1	2	2	4	4	3	5
4	2	1	2	1	5	4	3	4
4	3	1	1	1	5	3	4	4
5	3	1	1	1	5	2	3	5
5	2	1	1	1	5	4	4	4
4	3	2	2	3	5	4	4	5
5	3	1	1	1	4	3	1	4
4	2	2	5	2	4	3	4	4
5	3	2	1	1	5	4	3	5
4	2	2	2	2	4	4	2	4
5	3	1	1	1	5	5	4	5
4	2	2	3	2	4	4	3	4
4	2	2	3	2	4	3	2	3
3	4	2	3	3	2	1	1	3
5	2	1	1	1	5	3	4	5
2	2	1	2	1	2	3	3	2
5	1	1	5	1	5	5	5	5
5	1	1	1	1	4	5	4	5
5	1	5	1	1	5	5	5	5
2	4	2	4	2	4	1	1	3
5	2	2	1	1	5	5	4	5
5	1	3	1	1	5	4	4	5
4	1	4	2	1	4	3	3	5
5	5	1	1	1	5	5	5	5
4	1	1	1	1	4	4	1	4
5	1	2	1	1	5	5	5	5
3	3	3	4	2	3	2	2	2
5	1	1	1	1	1	5	4	5
5	3	1	1	1	4	3	3	5
4	2	2	1	1	4	4	3	4
4	3	4	2	2	4	2	4	4
2	2	2	4	2	3	2	2	1
3	3	3	3	3	3	3	3	3
4	2	5	1	1	5	4	3	5
5	1	4	1	1	4	4	4	5
3	2	3	3	2	4	4	4	3
3	3	3	3	3	3	3	3	3
3	2	1	2	3	3	3	4	3
2	2	2	2	3	3	2	4	2
3	3	4	2	1	3	2	1	3
4	2	1	2	2	4	3	4	4
5	5	3	3	2	3	3	3	4
4	1	2	2	2	4	4	4	4
5	4	2	2	2	4	3	2	4
5	1	1	2	1	5	5	1	5
5	1	3	1	1	4	5	5	5
4	2	1	2	1	3	4	4	4
5	2	1	1	1	4	5	3	5

4	3	2	4	1	4	3	2	4
5	5	4	3	2	5	3	4	5
3	1	1	4	1	5	3	1	5
1	5	1	5	5	1	1	1	1
3	3	2	1	1	4	3	3	3
3	2	3	4	3	3	2	2	3
4	4	2	3	2	4	2	2	4
3	1	1	1	1	4	4	5	4
5	2	2	1	1	4	4	2	4
3	4	3	3	1	3	2	4	2
5	2	2	2	1	4	4	4	4
3	2	2	4	2	4	3	1	4
3	3	2	2	2	3	2	3	3
5	1	1	2	1	4	5	5	5
5	1	4	2	1	5	5	5	5
3	4	2	3	3	3	3	3	4
4	2	2	2	1	4	3	4	4
5	2	2	2	1	5	4	4	5
4	4	4	3	3	3	4	2	4
4	3	3	2	2	4	4	4	4
4	2	4	2	2	3	3	4	4
3	2	4	3	2	3	2	3	3
5	5	5	5	5	5	5	5	5
5	2	5	1	1	3	5	3	4
4	5	4	5	2	4	2	4	4
2	2	2	3	2	3	2	2	3
3	3	3	3	3	3	3	2	2
4	1	1	1	1	3	4	5	4
3	3	3	3	3	3	3	3	3
4	3	2	2	2	4	4	4	4
3	3	3	3	3	3	3	3	3
2	3	2	4	3	4	2	2	3
4	3	2	4	2	5	3	3	4
4	4	3	2	1	4	3	3	4
4	3	4	2	2	4	3	3	4
4	3	2	2	2	3	4	4	4
3	2	2	2	2	4	4	4	4
3	2	2	2	1	3	4	4	4
4	3	2	3	2	4	3	3	4
4	3	1	3	3	3	3	3	3
4	1	1	3	1	4	3	4	4
4	2	2	1	1	3	3	4	4
3	4	4	3	2	3	2	2	3
4	1	1	1	1	2	4	4	4
2	3	2	2	2	3	2	2	2
4	4	4	4	2	5	2	2	4
5	1	1	1	1	5	5	4	5
4	2	2	1	1	4	3	2	3
4	2	1	2	2	4	4	4	2
4	3	1	2	1	4	3	3	4
4	1	1	2	1	3	4	4	4
4	3	2	2	2	4	4	4	4
5	2	1	2	1	5	5	4	5
5	2	2	1	1	4	2	2	4

4	3	1	4	1	5	3	4	3
4	2	2	2	2	4	4	4	4
4	2	3	2	2	4	3	2	4
5	4	2	2	1	5	5	5	5
5	1	1	1	1	1	4	2	4
5	2	2	1	1	4	2	2	4

C15	C16	C17	C18	C19	C20	C21	C22	C23
5	5	5	5	5	5	5	5	5
5	4	1	1	5	3	2	4	3
5	5	5	5	5	5	5	5	5
4	3	4	2	5	5	5	5	5
3	3	3	3	3	3	3	3	3
5	5	5	5	5	5	5	5	5
3	5	5	5	5	3	5	5	5
4	2	3	2	4	2	4	3	4
3	3	3	3	3	3	3	3	3
3	5	5	1	5	5	5	5	5
3	1	4	1	5	5	4	5	5
4	2	3	2	4	3	3	3	4
1	4	4	1	4	5	5	5	5
5	5	5	5	5	5	5	5	5
5	5	2	5	5	5	3	5	4
5	5	5	5	5	5	5	5	5
3	2	2	2	4	3	3	3	4
3	4	5	1	5	5	5	5	5
3	5	4	5	4	3	3	4	5
4	2	3	1	5	3	3	4	4
1	5	2	5	4	5	2	5	4
5	2	3	5	4	2	5	5	5
5	4	5	1	5	5	4	5	4
1	1	5	1	5	5	5	5	3
4	2	2	3	3	3	4	2	2
3	2	3	2	4	4	3	3	4
3	2	3	2	3	3	3	3	3
4	3	2	2	4	4	4	4	4
4	2	4	1	4	5	4	5	5
4	2	2	2	4	4	3	4	3
2	5	5	1	5	5	5	5	5
1	1	5	1	5	5	5	5	5
3	2	4	1	5	5	4	4	4
3	4	5	1	5	4	4	5	4
3	1	3	1	5	3	2	4	4
4	1	4	1	4	4	4	4	4
1	1	5	1	5	5	5	5	5
5	5	5	5	5	5	5	5	5
2	2	4	1	5	5	5	5	5
1	5	5	1	5	5	5	5	5
4	4	4	1	4	4	4	4	4
2	4	4	2	5	5	5	4	4
2	3	5	2	5	5	5	5	5
1	2	3	2	5	5	3	3	5
4	4	4	4	4	4	4	4	3
4	4	4	4	4	4	4	4	4
2	2	4	2	5	5	4	5	5
5	5	5	5	5	5	5	5	5
3	3	2	2	3	1	2	1	1
4	5	1	1	1	2	1	5	2
5	5	5	1	5	5	5	5	5
4	4	5	1	5	5	5	5	5
3	3	5	2	4	4	5	5	3

5	3	2	2	4	4	4	4	4
3	3	3	1	4	4	4	4	4
2	3	2	2	3	2	4	5	5
5	5	5	5	3	4	5	5	5
5	3	3	1	3	5	5	3	2
3	3	3	1	5	4	4	4	4
1	3	5	1	5	5	5	5	5
5	5	5	1	5	5	5	5	5
4	3	2	2	4	4	5	4	2
2	3	2	1	5	3	3	4	4
3	3	4	2	4	3	4	4	4
2	4	3	2	5	4	4	4	4
3	5	5	1	5	5	5	5	4
3	5	1	2	2	5	4	3	3
2	4	4	2	4	4	4	4	4
2	1	3	1	5	3	5	5	3
1	1	5	1	5	5	5	5	5
2	2	5	2	5	5	5	5	4
4	4	4	1	5	4	4	3	4
3	4	2	1	5	3	3	3	3
2	4	3	2	5	4	4	4	4
4	4	3	1	5	5	4	4	3
5	5	5	5	5	5	5	5	5
3	3	3	3	3	3	3	3	3
4	3	2	3	3	3	3	3	3
5	5	1	1	3	1	1	1	1
2	3	4	1	5	5	4	4	4
3	3	2	1	5	4	4	4	2
4	1	3	1	4	4	3	3	2
4	3	3	3	3	3	3	3	2
4	3	1	3	3	3	1	1	1
2	1	3	1	5	3	3	3	2
3	2	4	1	5	5	3	5	4
2	2	2	2	3	2	2	3	1
2	1	3	1	5	4	4	3	5
2	2	5	1	5	5	5	5	5
4	1	4	1	5	4	5	4	4
3	1	3	2	3	3	3	3	3
5	3	5	2	3	2	1	3	2
3	3	5	1	5	5	3	4	5
4	2	3	2	4	3	3	3	3
2	2	4	2	4	4	4	4	4
3	3	3	3	3	3	3	3	3
1	1	5	1	5	4	4	5	5
2	5	2	1	3	2	2	1	2
4	2	2	3	4	5	2	1	4
4	4	4	4	4	3	3	3	4
3	2	3	2	5	4	3	3	3
1	4	4	1	5	5	5	5	4
3	1	2	2	5	5	2	3	1
4	3	2	2	4	4	2	2	2
2	3	4	2	4	3	5	5	4
2	2	2	2	2	2	4	2	2
4	5	2	2	4	3	2	3	2

3	3	3	3	3	3	3	3	3
5	1	1	3	3	2	2	2	2
1	3	4	1	5	5	4	2	3
1	5	5	1	5	5	5	5	5
1	1	5	1	5	5	5	5	5
4	2	2	1	5	5	2	5	2
4	5	3	1	2	3	3	3	3
2	5	2	1	5	2	3	4	4
1	1	4	1	5	5	5	2	5
3	3	3	3	3	3	3	3	3
3	3	3	3	3	3	3	3	3
2	1	5	1	5	5	4	4	4
3	3	4	1	5	5	4	4	5
3	3	3	2	5	4	4	4	4
2	4	4	2	4	5	4	4	4
3	2	3	2	4	4	4	4	3
3	3	4	1	5	3	4	3	3
5	2	3	2	5	5	5	5	5
3	2	4	2	4	4	4	4	4
3	4	4	1	4	4	4	4	4
3	4	4	2	5	4	4	4	3
1	1	5	1	5	5	5	5	5
3	3	4	3	3	3	3	3	3
3	3	3	3	3	3	3	3	3
4	3	2	3	4	2	2	2	2
4	2	1	3	5	2	2	2	2
3	5	3	3	3	3	3	3	2
5	2	1	4	3	3	2	1	1
5	4	2	1	4	5	4	4	3
2	5	4	1	5	5	4	5	4
2	5	4	1	4	4	4	4	4
2	1	3	2	5	5	4	4	4
3	3	4	5	4	3	3	3	3
1	1	1	5	1	1	1	1	1
3	4	2	2	2	3	5	5	2
1	2	4	1	5	5	5	4	5
3	3	3	1	5	4	5	4	3
5	2	4	1	5	4	4	4	2
4	1	4	4	4	4	4	4	4
3	5	5	5	5	5	5	1	5
1	4	4	2	5	5	4	5	5
5	2	1	2	3	2	4	2	1
3	3	3	2	1	3	3	3	3
5	3	2	3	5	3	3	3	2
1	1	5	1	5	3	3	3	5
1	4	4	1	5	5	4	3	5
5	1	1	1	3	1	1	1	1
3	3	4	1	5	5	5	4	5
3	2	4	2	4	4	3	4	3
5	4	2	1	4	5	2	3	2
2	3	4	1	5	5	4	4	4
3	5	3	5	5	5	3	5	3
5	3	3	1	5	2	3	4	5
2	2	4	2	4	4	4	4	4

1	2	3	1	5	4	3	5	5
3	2	3	2	4	4	3	3	3
1	5	5	1	5	5	5	5	5
4	2	2	2	4	3	2	3	2
4	2	4	1	5	5	4	5	2
2	4	4	2	4	4	4	4	4
3	3	4	2	5	3	3	4	4
2	2	4	2	4	4	4	4	4
3	3	2	4	3	2	2	2	3
2	2	2	1	4	2	2	4	4
3	4	4	2	5	4	4	3	5
1	3	5	1	5	5	4	5	5
3	3	3	2	4	3	3	4	2
1	1	5	1	5	5	5	5	5
3	3	3	2	4	4	3	3	3
5	4	2	1	5	4	2	4	2
2	4	4	1	5	5	5	5	4
4	3	4	2	4	4	3	4	4
3	3	3	3	3	1	1	3	3
1	5	5	5	5	5	5	5	5
4	4	3	1	5	3	2	4	3
2	4	5	1	5	5	5	5	4
3	4	3	1	5	4	3	3	2
2	2	5	1	5	5	5	5	5
5	1	1	3	5	1	1	1	1
4	3	3	2	4	2	2	3	2
4	2	2	2	4	3	3	3	2
3	3	3	2	4	3	3	3	3
5	1	1	2	4	1	1	1	1
3	3	3	2	5	5	5	5	5
4	3	3	2	4	3	4	4	2
1	4	5	1	5	5	5	5	5
3	1	5	1	5	5	5	5	4
1	1	3	1	5	5	1	3	5
3	2	1	2	4	4	4	3	4
3	2	4	1	5	5	5	5	4
3	4	3	2	4	4	2	3	3
3	3	4	1	5	5	4	4	3
2	4	3	1	4	3	3	3	4
1	4	5	2	5	5	5	5	5
3	5	3	1	5	5	5	5	5
4	4	4	2	4	4	4	4	4
5	2	2	1	5	4	5	5	5
3	4	3	1	4	4	3	4	3
4	3	3	2	4	3	4	3	4
3	4	3	2	4	3	3	4	3
3	3	3	3	3	3	3	3	3
1	2	5	1	5	5	5	5	5
2	2	5	1	5	5	5	5	4
3	1	5	1	5	5	5	5	5
1	2	4	1	5	5	4	5	4
3	1	4	1	4	3	4	3	3
3	3	3	3	3	3	3	3	3
4	4	4	1	5	5	4	5	4

3	3	3	3	3	3	3	3	1
1	5	4	5	3	2	1	5	5
2	5	3	1	4	4	4	5	5
3	2	2	1	4	3	3	3	3
5	4	4	1	5	5	5	5	5
2	2	5	1	5	5	5	5	5
1	4	4	4	3	4	4	4	4
1	1	4	4	5	4	4	2	5
4	2	2	2	4	4	4	4	4
2	4	2	2	4	4	3	4	3
3	3	3	1	5	3	3	3	3
1	1	3	1	5	5	5	5	5
1	1	1	1	1	1	1	1	1
3	3	5	1	4	4	5	5	3
5	3	2	2	4	5	4	4	4
4	5	5	1	5	5	5	5	5
3	3	3	3	3	3	3	3	3
3	3	4	2	4	4	3	3	3
3	2	3	3	4	4	3	3	3
4	4	3	1	4	5	5	4	4
3	4	4	3	4	3	4	4	4
2	4	4	2	4	4	4	3	4
2	3	5	1	5	5	5	5	5
1	5	5	5	5	5	5	5	5
3	3	3	3	3	3	3	3	4
4	4	2	2	5	3	3	3	3
3	3	2	2	4	4	3	3	3
3	3	5	1	5	5	5	5	5
4	3	4	3	4	5	4	5	3
2	1	2	1	5	5	4	4	4
3	3	2	2	3	3	3	3	3
5	1	1	3	3	4	1	1	1
5	2	1	3	4	2	2	2	2
5	5	5	1	5	5	5	5	4
2	3	3	1	4	3	4	4	4
3	2	3	2	5	4	3	4	3
4	1	2	1	5	4	3	4	3
4	4	4	1	5	5	4	1	1
4	4	4	4	4	4	4	4	4
2	5	5	2	5	5	5	5	1
2	4	4	2	4	4	4	4	4
1	3	3	1	4	3	3	3	2
5	3	4	1	3	3	4	5	2
2	1	5	1	5	5	5	5	5
1	1	3	1	5	3	5	5	5
3	3	3	2	4	4	3	4	3
2	4	2	1	4	3	3	5	3
4	2	1	1	4	4	3	3	2
1	4	5	1	5	5	5	3	4
3	4	3	4	4	3	4	4	2
3	3	3	3	4	4	4	4	4
1	3	5	1	5	5	5	5	5
3	3	3	3	3	3	3	3	3
4	3	4	2	4	3	3	4	3

5	1	4	1	5	5	5	5	5
3	1	4	1	4	3	3	4	4
4	3	2	1	5	3	3	2	1
3	3	3	2	4	3	3	3	3
3	3	3	3	3	3	3	3	3
1	5	4	1	5	5	4	5	5
1	1	5	1	5	5	5	4	5
4	4	4	4	4	4	4	4	4
5	3	2	1	5	3	5	2	4
5	1	1	1	5	1	1	1	1
3	2	3	1	4	4	3	4	3
5	4	2	1	5	5	4	4	2
4	2	2	2	4	4	2	2	2
1	4	3	1	5	5	3	3	3
5	1	3	1	5	5	4	4	2
2	3	5	2	5	5	5	5	5
3	3	3	3	3	3	3	3	3
4	2	2	1	4	4	2	2	2
4	4	4	1	5	5	5	5	5
4	3	3	3	4	3	2	2	2
3	4	4	3	4	4	4	4	4
4	2	3	2	5	4	3	4	4
5	2	4	1	5	5	4	5	3
2	2	4	2	4	2	3	2	2
1	1	5	1	5	5	5	5	5
5	1	1	1	5	1	5	3	1
4	3	2	2	4	4	4	4	4
4	4	3	2	4	3	3	4	4
1	3	5	1	5	5	5	5	5
4	2	4	1	5	5	5	5	5
1	1	4	1	5	5	5	5	5
3	2	4	2	5	3	3	4	4
5	1	1	4	3	2	2	1	1
5	5	5	2	5	5	5	5	5
4	3	3	2	4	3	3	3	4
4	5	5	2	4	3	3	4	3
3	2	5	1	5	5	5	4	3
3	3	4	1	5	5	4	4	4
4	4	4	4	4	4	4	2	2
4	2	4	4	4	4	3	4	4
2	2	4	2	4	4	4	4	4
4	3	2	3	4	3	3	3	3
4	3	3	2	4	3	3	3	2
4	4	4	2	5	4	4	4	4
4	3	2	2	4	4	3	4	3
4	2	2	2	4	2	2	2	2
4	4	4	2	3	4	4	3	4
2	2	4	2	4	4	4	4	4
1	1	4	1	4	5	4	3	5
4	2	4	1	5	5	2	3	3
5	3	1	3	4	2	4	3	3
2	1	4	1	5	5	5	5	5
4	4	2	2	4	4	3	4	4
4	3	2	4	4	2	2	2	2

3	4	4	1	4	4	2	4	4
5	3	4	4	5	4	4	4	4
5	3	3	1	5	5	4	4	3
4	5	5	2	5	5	5	5	5
4	4	4	4	4	4	5	4	4
4	2	2	2	4	2	2	2	2
4	2	4	1	4	4	4	4	4
4	2	3	2	4	2	2	2	4
4	2	2	3	4	4	3	3	3
4	4	3	3	4	4	3	4	4
3	2	4	1	5	5	3	2	3
2	3	4	2	4	4	4	4	4
4	4	4	4	4	4	4	4	4
3	3	4	2	4	4	4	4	4
4	3	3	2	4	4	3	4	4
4	4	4	4	4	4	4	4	4
4	4	4	4	4	4	4	4	4
4	3	4	1	5	4	4	4	3
3	3	3	2	4	4	3	3	3
3	3	3	3	3	3	3	3	3
2	1	4	2	4	4	4	4	4
4	4	3	2	4	3	3	4	3
4	3	3	2	5	5	4	5	4
3	3	3	2	4	4	3	4	4
4	2	4	2	4	4	4	4	4
5	4	5	1	5	5	4	5	2
3	2	3	2	3	3	4	5	4
4	2	2	2	4	4	2	4	2
4	2	2	1	5	5	2	4	4
3	4	3	2	4	4	4	3	4
4	4	4	1	5	4	4	4	4
5	2	2	2	4	2	3	4	4
4	3	4	2	5	4	4	4	3
3	4	3	1	5	4	4	3	4
2	4	4	2	4	4	4	4	4
4	3	2	1	4	3	3	4	3
5	1	2	1	4	2	2	4	2
5	5	4	2	4	3	3	3	3
2	2	4	2	4	4	4	4	2
5	5	1	1	5	5	5	5	1
4	3	2	2	5	3	3	4	2
5	4	3	3	4	4	4	2	3
3	3	2	3	3	3	3	3	3
4	3	2	2	4	3	3	3	3
3	4	2	1	5	3	1	3	2
2	3	4	2	4	4	4	4	4
4	2	2	1	4	3	2	4	2
3	1	4	2	5	5	5	5	5
2	2	4	2	4	4	4	4	4
2	2	5	3	3	3	4	4	4
4	2	5	1	5	5	5	5	4
4	2	4	4	2	4	4	4	4
4	4	4	2	4	5	5	4	3
4	4	3	3	3	3	3	3	2

3	2	3	2	2	4	4	4	3
2	4	2	1	5	5	5	5	5
4	2	2	2	4	4	4	4	4
5	3	3	3	3	3	3	3	5
4	3	2	2	4	2	2	4	2
2	5	5	1	5	5	5	5	5
3	3	3	2	4	4	4	4	3
4	1	4	1	4	2	2	5	1
4	3	3	2	4	4	3	4	4
5	5	3	3	4	2	2	1	1
4	4	3	2	4	4	4	4	4
3	4	3	2	4	5	3	4	4
3	3	3	3	4	4	3	3	3
4	2	4	2	4	4	4	4	4
3	3	4	1	1	5	3	3	3
2	3	4	2	4	3	3	3	3
4	1	2	1	4	4	3	4	2
4	3	4	4	3	5	5	4	5
1	5	5	1	5	5	5	5	5
1	2	5	1	5	5	5	5	5
5	4	4	4	5	5	5	4	4
2	2	5	1	5	5	5	5	5
3	4	4	1	5	5	4	4	4
3	1	5	1	5	5	5	5	5
5	1	1	5	3	3	3	3	1
4	3	2	2	4	3	3	4	3
5	1	4	1	4	4	4	4	4
5	3	3	1	5	5	3	5	3
5	3	3	2	4	3	4	3	4
5	4	3	1	5	3	3	3	3
2	3	5	1	5	5	5	5	4
3	3	3	3	3	3	3	3	3
3	3	4	2	5	5	4	5	5
5	1	1	4	1	1	1	1	1
4	4	2	2	4	2	3	2	2
4	1	1	3	4	4	3	3	3
2	2	2	2	2	2	2	2	2
4	2	2	2	4	4	3	4	4
5	2	2	2	4	4	2	4	4
2	3	2	1	3	2	2	2	2
5	2	3	1	5	5	5	5	5
4	3	3	2	4	4	3	4	3
5	2	2	3	4	2	2	2	2
5	5	2	3	4	4	4	2	3
4	4	4	4	4	4	4	4	4
2	2	2	2	4	2	4	4	4
2	2	4	2	4	4	4	3	4
5	1	1	5	1	4	2	3	1
1	1	5	1	5	5	4	5	4
2	3	3	2	4	4	3	4	4
5	2	3	3	3	3	3	5	5
3	3	3	3	3	3	3	5	4
5	4	2	2	4	4	2	2	2
3	3	3	2	4	4	4	4	4

4	2	2	1	5	4	3	3	3
3	3	2	3	4	2	3	3	3
4	2	5	1	5	5	2	5	2
4	2	4	1	4	5	3	4	4
5	5	1	3	1	1	1	3	1
3	2	2	1	5	2	3	3	2
3	3	4	1	5	4	4	4	4
3	2	2	2	3	2	3	3	2
3	4	5	1	5	5	5	5	4
5	2	5	1	5	5	5	5	3
4	2	4	2	4	4	4	4	4
5	4	4	1	5	5	5	5	5
4	2	4	1	5	5	5	5	5
5	3	2	3	4	4	3	4	2
4	2	3	2	4	3	3	4	3
1	5	5	1	5	5	5	5	5
4	2	4	2	5	4	4	4	3
5	3	3	3	4	4	3	3	2
3	2	3	1	5	4	4	4	4
4	2	4	1	5	4	4	4	4
3	3	3	2	4	3	3	3	3
4	4	4	2	4	4	4	4	4
4	3	2	2	4	3	3	3	4
4	4	2	3	4	4	3	3	3
4	3	2	2	4	2	3	4	2
1	1	1	1	5	1	3	1	1
2	2	5	1	4	1	5	5	4
5	2	2	2	4	3	3	3	2
5	5	5	5	5	5	5	5	5
4	2	2	2	4	2	2	2	2
4	4	3	2	5	5	4	4	3
2	5	5	1	5	5	5	5	5
3	3	2	2	4	2	2	2	2
4	2	3	2	4	4	4	4	4
1	1	5	1	5	5	5	5	5
4	3	3	2	4	3	3	3	4
4	2	4	1	5	5	5	5	5
4	2	3	4	4	4	4	4	4
5	5	3	3	4	2	2	2	2
3	4	4	2	4	4	4	4	4
4	2	4	2	4	3	3	4	4
4	5	2	1	5	4	5	5	4
4	4	2	2	4	4	3	2	2
4	2	4	2	4	4	3	4	4
2	3	5	1	5	5	5	5	4
4	2	5	1	5	4	4	4	4
4	3	4	1	3	3	2	5	3
4	2	4	2	4	4	4	4	4
4	3	1	4	3	2	2	2	2
3	3	3	3	3	3	3	3	3
2	2	4	2	4	4	4	4	4
3	3	3	3	3	3	3	3	3
4	4	3	1	4	3	3	3	4
4	3	3	2	4	4	3	4	4

2	2	3	2	2	3	3	3	3
5	4	4	4	4	4	3	4	5
4	2	2	2	4	4	3	4	2
2	4	2	4	3	3	2	4	2
3	3	3	3	4	4	3	4	4
2	2	5	2	4	5	4	4	4
3	3	1	4	4	3	1	1	2
4	2	2	2	2	2	3	2	2
5	3	1	2	3	2	1	2	2
5	1	1	1	1	3	3	3	1
3	3	3	1	5	4	3	5	3
4	3	3	2	5	4	3	4	4
5	2	3	3	4	4	3	3	3
4	2	4	1	5	4	4	4	4
2	4	2	2	5	5	3	4	4
5	3	4	2	5	4	3	4	4
3	3	2	2	4	3	2	2	2
3	2	2	2	4	3	3	3	3
2	2	3	1	5	5	4	4	4
4	1	1	1	5	5	4	4	4
4	3	2	3	3	2	3	2	2
4	4	4	4	4	4	4	4	4
3	3	2	3	3	3	2	3	4
3	3	3	3	4	4	2	3	3
3	4	4	1	5	5	5	5	5
5	3	5	1	5	3	1	5	5
3	3	3	2	4	4	4	4	4
4	4	3	2	4	4	4	4	4
4	2	3	2	5	3	4	4	3
2	1	2	1	5	2	1	3	3
5	4	5	5	5	5	5	5	5
1	4	2	2	3	2	4	3	4
3	3	3	2	2	2	2	2	2
4	4	4	4	4	4	4	4	4
2	3	4	2	5	5	4	4	4
3	3	3	3	3	3	3	3	3
1	5	5	1	5	5	5	5	5
1	2	5	1	5	5	5	5	5
4	4	4	4	4	3	4	3	3
1	3	5	1	5	5	5	5	5
4	4	4	4	4	4	4	4	4
4	3	2	2	2	4	4	2	2
2	2	4	2	3	3	2	5	5
3	4	4	2	4	4	4	4	4
4	4	4	4	4	4	4	4	4
4	3	2	3	4	3	3	3	2
4	4	4	3	4	4	4	4	2
4	4	4	1	4	4	4	4	4
2	4	4	2	5	4	4	4	4
2	3	4	2	4	4	4	4	4
2	2	4	2	4	4	4	4	3
3	4	4	3	3	4	3	3	3
1	1	5	1	5	5	5	5	5
2	2	5	1	4	4	4	5	5

4	1	5	1	5	5	5	5	5
1	5	5	1	5	5	5	5	5
4	2	4	2	4	4	4	4	4
5	3	2	3	3	2	2	4	4
2	2	4	2	5	5	4	4	5
1	2	5	1	5	5	5	5	5
5	2	2	1	5	5	3	3	2
3	3	5	1	5	5	5	5	3
3	2	3	1	5	3	2	3	3
3	3	4	1	5	5	4	4	4
1	2	4	1	5	5	4	3	4
2	2	3	2	2	2	2	1	2
3	2	3	2	4	3	4	4	3
2	2	4	2	4	4	4	4	4
3	4	4	2	4	4	4	4	4
4	2	2	2	4	2	2	2	2
4	2	2	2	4	2	4	4	2
1	1	4	1	4	4	4	4	4
2	2	4	2	4	4	4	4	4
5	3	5	1	5	5	5	5	5
3	2	5	1	5	5	5	5	5
5	5	1	1	4	1	1	1	1
3	3	3	3	3	3	3	3	3
3	3	2	3	4	3	4	4	4
5	5	5	1	5	5	4	5	4
3	3	3	3	5	5	3	3	3
4	3	3	3	4	3	3	3	3
3	2	3	1	5	5	3	4	4
2	4	4	2	4	4	4	4	4
5	2	1	4	4	1	1	1	1
3	3	3	2	4	3	4	4	3
4	4	2	2	5	3	3	4	3
1	5	5	1	5	5	5	5	5
1	1	2	1	5	5	5	5	5
2	2	4	2	4	5	5	5	3
1	5	5	1	5	5	5	5	5
2	2	5	5	5	5	5	4	5
2	4	4	2	4	4	4	4	4
3	3	5	2	5	5	5	5	5
2	2	4	2	4	4	4	4	4
5	5	5	5	5	5	5	5	5
1	2	3	3	3	3	5	5	5
3	3	4	1	4	4	3	4	5
4	3	3	2	4	4	4	4	4
2	4	4	1	4	4	4	5	4
5	2	3	1	5	5	3	4	5
5	1	1	1	5	3	3	3	5
3	3	3	2	3	4	4	4	3
4	2	4	2	4	4	2	2	3
3	3	4	2	4	5	5	4	4
4	3	3	3	3	3	3	3	2
2	2	3	2	4	4	3	3	4
2	4	4	2	4	4	4	4	4
2	4	4	1	5	5	4	5	5

1	5	5	1	5	5	5	5	5
3	2	3	1	5	5	5	5	5
2	3	3	2	3	4	3	5	4
2	2	3	1	4	4	4	4	4
4	2	2	1	4	5	3	2	2
1	5	5	1	4	4	4	4	5
4	3	3	1	4	3	2	4	2
1	5	5	1	5	1	5	5	4
5	5	5	5	5	5	5	5	5
2	1	3	1	5	4	4	4	4
2	2	4	1	4	4	4	3	4
5	3	4	3	3	3	3	2	2
1	1	5	1	5	5	5	5	5
3	3	3	3	3	3	3	3	3
2	2	2	1	4	4	4	3	3
3	4	5	2	4	4	4	5	3
1	3	3	2	3	5	4	5	4
1	1	1	1	1	2	4	4	3
2	2	2	3	2	2	3	1	1
3	3	4	2	4	4	4	3	3
2	2	3	2	3	4	3	3	4
2	4	4	2	4	5	4	4	4
1	3	4	1	5	4	5	3	5
1	1	4	1	4	5	4	4	3
3	3	3	3	3	3	3	3	3
1	3	5	1	5	5	5	5	3
2	4	3	1	5	5	4	5	5
4	4	2	2	4	2	2	4	4
3	5	3	1	5	4	4	4	3
2	2	4	2	4	4	4	4	4
3	3	3	3	3	3	3	3	3
1	2	5	2	5	5	4	5	5
4	2	4	2	4	4	4	4	4
2	3	4	1	5	5	5	4	5
5	2	2	1	4	1	2	2	2
3	3	3	2	3	3	2	2	2
4	3	2	3	4	5	3	3	2
1	1	5	1	5	5	5	5	5
2	2	4	2	2	4	4	4	4
2	3	4	1	4	5	3	4	4
3	2	2	2	4	4	4	3	4
1	1	5	1	5	5	1	5	5
5	3	5	1	5	5	5	5	5
2	4	5	1	5	5	5	5	5
3	3	3	3	3	3	3	3	3
2	4	3	2	4	4	3	4	4
1	3	4	1	4	5	4	3	4
3	2	3	1	5	5	4	3	2
4	3	2	2	4	3	2	2	2
2	1	5	1	5	5	5	5	4
3	3	4	2	4	4	4	4	4
2	3	5	1	5	5	4	4	4
1	5	5	1	5	5	5	5	5
2	4	2	3	4	5	3	4	2

3	3	3	3	3	3	3	3	3
2	2	4	2	4	5	4	3	3
1	2	5	2	4	4	4	4	4
3	3	3	3	3	3	3	3	3
3	4	3	5	3	2	2	5	5
1	1	5	1	5	5	4	5	4
5	5	5	5	5	5	5	5	5
2	2	3	2	2	2	2	2	2
2	4	4	2	5	5	5	4	4
1	5	5	1	5	5	5	5	5
1	5	5	1	5	5	5	5	5
4	3	4	2	4	4	4	3	3
1	2	5	1	5	5	5	5	5
5	1	1	5	1	5	1	1	5
3	2	4	1	5	3	3	4	3
4	5	4	2	5	5	2	4	4
3	2	4	1	5	3	4	4	3
2	2	5	1	5	5	5	5	5
3	3	4	2	4	4	4	4	4
3	4	4	2	3	3	3	4	3
3	2	4	2	5	4	4	3	4
3	3	4	3	3	3	3	3	4
1	1	5	1	5	4	4	5	5
1	5	5	1	5	5	4	5	5
2	2	2	2	3	3	3	3	3
4	3	3	2	4	4	4	3	2
4	5	3	5	4	2	2	3	3
4	3	2	2	4	2	2	3	2
2	3	4	1	4	4	3	3	3
4	3	2	3	4	4	3	3	4
2	2	4	2	4	4	4	4	4
3	4	4	3	3	3	4	3	3
4	4	4	2	4	4	4	4	4
4	3	2	2	4	3	3	3	2
3	3	3	3	3	3	3	3	3
4	4	2	2	4	4	5	4	4
4	3	2	2	4	2	2	2	2
3	4	3	2	5	3	4	4	3
4	3	3	2	5	4	4	4	4
3	2	2	2	4	4	3	3	3
5	3	3	2	5	3	2	2	2
3	3	5	2	5	5	4	4	4
3	4	4	1	5	4	4	4	3
5	4	3	3	2	2	3	3	1
4	3	2	3	3	3	2	3	3
1	5	5	1	5	5	5	5	5
5	4	2	3	4	3	2	3	2
2	4	4	2	4	4	4	4	4
3	2	4	2	4	4	4	4	4
3	4	3	1	5	4	2	2	4
3	3	4	2	4	4	3	3	3
3	2	4	2	4	4	4	4	4
3	3	3	3	3	3	3	3	3
3	4	3	2	4	4	3	4	3

4	2	2	1	5	4	4	3	4
4	2	2	2	4	3	3	3	3
2	1	2	2	3	3	3	3	3
3	3	4	2	2	4	4	4	4
4	4	2	3	4	2	2	2	2
5	1	1	1	5	1	1	1	5
3	3	1	1	2	3	4	2	2
3	2	1	4	4	2	2	2	2
3	2	4	1	5	5	5	4	5
2	2	5	2	5	5	5	5	5
4	2	4	2	4	4	2	4	4
1	1	1	1	1	1	1	1	1
2	3	5	2	3	5	4	2	5
4	3	2	3	4	4	3	2	2
2	4	4	1	5	4	4	2	4
3	2	3	3	3	3	2	2	4
3	3	3	2	4	4	4	4	4
4	1	1	3	2	1	2	3	3
4	2	2	2	4	4	4	4	2
5	1	2	3	4	1	2	2	4
2	2	4	4	5	5	5	5	5
3	2	3	3	3	3	3	3	3
3	3	2	3	4	3	4	4	4
3	4	3	2	4	4	4	4	4
2	4	4	3	4	4	4	4	4
5	1	1	3	4	4	3	4	3
4	2	2	2	3	3	3	3	2
5	1	1	1	2	1	1	1	1
4	4	2	2	4	2	2	4	2
4	2	2	2	4	4	4	4	2
3	3	2	4	4	4	3	4	4
3	4	3	2	4	4	3	4	3
3	2	2	2	4	4	4	4	3
1	1	5	1	5	5	5	5	5
4	4	2	2	4	4	4	3	3
2	2	4	1	4	4	3	2	4
3	2	2	2	4	4	4	4	4
5	4	2	2	2	2	4	4	2
4	2	2	2	4	2	2	2	2
5	4	2	2	4	2	2	2	2
1	4	5	1	5	5	5	5	5
3	3	4	2	4	3	4	4	3
4	4	4	4	4	4	4	4	4
4	3	3	2	3	3	3	4	3
3	3	3	1	4	4	4	4	3
4	2	2	2	4	4	2	2	2
4	4	3	2	5	3	3	4	3
3	2	2	2	4	3	2	4	2
5	2	3	4	4	2	2	4	5
4	2	2	2	4	2	4	4	2
2	3	3	2	5	5	5	5	4
3	5	4	1	5	4	5	5	5
4	3	3	1	4	4	4	4	3
4	1	4	1	5	4	4	5	2

3	3	2	2	3	2	2	2	3
2	4	4	2	4	4	4	4	3
3	3	4	2	4	4	3	3	4
4	3	3	3	4	4	4	4	3
2	2	2	3	4	4	2	4	3
4	3	3	2	3	4	4	4	3
2	3	2	3	2	2	3	2	2
2	2	4	1	5	5	4	5	5
2	2	5	1	5	5	4	5	5
1	1	4	1	5	4	4	4	4
1	1	4	1	5	5	5	5	5
3	2	4	2	5	4	4	4	5
3	1	4	1	4	3	4	3	4
3	2	4	2	4	4	3	3	4
2	1	5	1	5	5	5	5	5
4	3	4	2	4	4	4	2	3
1	1	5	1	5	5	5	5	5
2	1	3	2	4	2	2	3	4
4	3	2	2	4	5	4	3	3
5	1	1	2	5	2	2	2	1
2	2	3	1	4	5	5	5	2
4	2	3	2	4	3	3	3	3
1	1	5	1	5	5	5	5	5
1	1	5	1	5	5	5	5	5
2	3	5	1	5	5	5	5	5
4	2	1	2	4	4	2	1	1
2	2	4	1	5	5	5	5	5
1	5	4	1	5	5	5	5	4
4	4	4	2	4	4	5	4	3
1	1	5	1	5	5	5	5	5
1	1	4	2	5	4	2	5	5
3	1	4	1	5	3	4	2	3
4	2	2	3	3	3	3	4	2
1	1	5	1	5	5	5	5	5
2	4	3	1	5	5	4	3	3
2	3	2	2	5	4	4	4	4
3	4	2	2	4	4	4	3	3
5	1	1	4	2	2	2	1	1
3	3	3	3	3	3	3	3	3
4	5	4	1	5	5	4	4	4
1	1	4	1	5	5	5	5	5
4	3	4	3	4	3	4	4	3
3	3	3	3	3	3	3	3	3
3	3	3	2	4	4	4	4	3
4	3	2	2	3	2	3	3	3
4	4	4	1	4	2	3	3	3
2	2	4	1	4	4	3	4	3
3	3	3	2	4	4	4	3	3
4	2	4	2	4	4	4	4	5
3	2	3	1	5	4	4	4	4
3	1	3	1	5	5	4	3	2
2	1	2	1	5	5	5	5	4
4	1	3	1	3	3	4	4	4
1	3	5	1	5	5	5	5	4

5	2	2	2	4	4	3	4	4
2	4	4	2	5	5	5	5	5
3	1	4	1	4	5	4	4	3
5	1	1	5	1	1	1	1	1
4	2	3	1	5	5	3	3	2
4	3	2	1	4	3	2	2	2
3	2	2	3	4	3	3	3	3
4	1	4	1	5	4	4	4	3
2	4	4	2	4	5	4	4	4
4	4	1	1	3	2	3	2	2
3	3	4	2	5	5	4	4	4
3	1	1	2	4	3	3	1	3
3	3	3	2	4	3	3	4	4
2	4	5	1	5	5	5	5	5
1	4	5	1	5	5	5	5	4
4	3	3	3	4	3	3	3	2
4	3	2	1	4	4	4	4	4
1	3	4	2	5	5	4	5	5
2	4	4	2	4	4	4	4	4
3	3	3	2	4	2	3	4	3
4	3	3	1	3	3	3	3	3
4	4	1	3	2	2	2	2	2
5	5	5	5	5	5	5	5	5
5	3	4	1	1	1	1	1	5
5	5	3	1	5	4	4	3	1
4	3	3	2	3	3	3	3	3
3	2	3	3	3	3	3	2	3
1	3	4	1	5	4	4	4	4
3	3	3	3	3	3	3	3	3
3	2	4	2	4	4	4	4	4
4	3	3	3	4	3	3	3	3
4	2	2	3	3	2	3	3	2
5	3	3	2	4	3	2	2	1
3	3	3	1	5	3	3	3	3
4	4	3	2	4	4	3	3	3
2	3	4	2	3	4	4	4	4
3	3	4	2	5	5	5	4	4
1	3	4	1	5	4	4	3	4
3	3	2	2	4	4	3	3	3
3	3	3	3	3	3	3	3	3
4	1	2	1	4	4	4	4	3
4	1	4	1	5	5	4	3	4
4	4	2	2	4	3	2	3	3
2	2	3	2	5	5	5	4	4
3	3	2	3	4	3	3	3	3
5	4	2	2	4	5	2	4	2
2	2	4	1	5	5	5	5	5
1	2	3	1	5	5	4	3	4
2	2	4	1	4	5	4	4	4
1	1	4	1	4	4	4	4	5
2	2	3	1	4	4	4	4	4
4	4	3	1	5	4	4	4	4
3	2	5	1	5	5	5	5	5
3	2	3	1	4	5	4	5	4

3	2	3	2	5	3	3	4	4
2	4	4	2	4	4	4	4	4
2	4	3	2	4	4	3	4	4
5	5	5	1	5	5	5	5	5
1	2	4	2	5	5	5	5	5
3	2	3	1	4	5	4	5	4

C24	C25	C26	C27	C28	C29	C30	C31	C32
5	5	5	5	5	5	5	1	4
1	5	4	4	1	1	2	5	4
5	5	5	5	5	5	5	5	5
5	5	5	5	5	5	5	5	5
3	3	3	3	3	3	3	3	3
5	5	5	5	5	5	5	5	5
5	2	4	4	5	5	5	5	5
2	4	4	4	2	2	4	4	2
3	3	3	3	3	3	4	4	4
4	5	5	5	4	4	5	1	4
4	2	5	5	2	4	5	4	5
3	3	4	3	4	3	4	4	2
4	1	5	5	3	5	5	1	5
5	5	5	5	5	5	5	5	5
5	5	5	5	1	5	3	5	5
5	5	5	5	5	5	5	5	5
2	2	4	5	2	3	3	2	3
5	5	5	5	5	5	5	5	5
5	2	4	4	3	4	3	3	2
1	4	5	5	2	3	2	3	1
2	3	3	5	5	5	5	5	5
5	5	5	5	5	5	5	5	5
4	1	5	5	4	5	5	1	5
1	1	5	5	3	5	5	1	1
2	4	4	4	4	2	3	4	4
2	2	3	4	3	4	3	2	3
1	3	3	3	2	2	3	3	3
3	2	4	4	3	4	4	4	4
2	1	4	3	2	5	5	2	4
3	3	4	5	4	2	3	2	2
3	1	5	5	4	5	5	1	5
1	1	5	5	5	5	5	1	4
2	1	5	4	4	4	4	2	4
5	1	4	5	5	5	5	5	5
2	2	5	4	3	2	4	5	5
2	1	4	4	4	3	5	4	4
1	1	5	5	5	5	5	5	5
5	5	5	5	5	5	5	5	5
3	1	5	5	4	4	4	4	4
5	1	5	5	5	5	5	5	5
4	1	4	4	4	4	4	1	4
3	4	4	4	4	4	4	4	4
3	1	5	5	5	5	5	2	5
2	1	5	5	3	5	4	2	2
4	2	4	4	4	3	4	2	4
4	4	4	4	4	4	4	4	4
2	2	4	5	5	5	5	2	3
5	5	5	5	5	5	5	5	5
2	3	2	2	3	2	2	2	2
5	1	5	5	4	1	5	4	3
5	5	5	5	5	5	5	5	5
5	1	5	5	5	5	5	3	5
3	3	4	4	5	4	3	3	4

2	3	3	3	3	3	3	3	3
4	2	4	4	3	3	3	3	4
5	5	5	5	5	5	5	5	5
5	5	5	5	5	5	5	5	5
1	4	5	5	5	5	5	5	5
3	1	4	4	3	4	4	2	5
5	1	5	5	5	5	5	1	5
5	5	5	5	5	5	5	1	5
2	4	4	4	4	4	4	4	4
5	3	4	4	3	3	4	4	5
2	2	4	4	3	3	4	2	3
4	2	5	5	3	3	4	2	5
5	1	5	5	3	5	5	2	5
4	5	5	5	4	3	4	3	2
5	2	5	5	4	4	4	2	4
3	1	5	5	4	4	5	1	5
1	1	5	5	3	5	5	1	5
1	3	4	5	3	5	5	1	5
4	2	5	5	2	3	4	3	5
4	3	5	5	3	3	3	3	4
4	4	3	4	4	4	4	3	4
4	3	5	5	3	5	5	2	4
3	3	5	5	5	5	5	5	5
3	3	3	3	3	3	3	3	3
3	3	3	3	3	3	3	3	3
1	4	3	4	2	1	2	5	4
3	1	5	4	3	4	4	2	5
1	3	3	3	3	3	3	3	4
3	1	4	4	3	3	2	4	4
1	3	4	5	2	3	3	4	4
3	4	3	3	3	3	3	4	3
3	2	5	3	2	2	2	3	1
1	1	5	5	5	5	5	1	2
2	2	3	3	3	3	3	3	2
1	1	5	3	3	4	4	2	1
5	5	5	5	5	5	5	1	2
1	1	5	5	3	4	5	1	4
3	2	5	5	3	2	3	2	4
2	1	3	2	2	2	4	1	5
3	1	5	5	5	5	5	1	3
2	3	3	4	2	2	2	4	3
2	2	4	4	4	4	4	2	3
5	4	3	3	2	3	1	3	5
1	1	5	5	5	5	5	1	4
1	1	4	5	5	4	4	2	2
3	3	4	3	3	4	4	5	1
4	4	5	4	5	5	4	4	4
2	3	4	5	3	3	3	3	5
5	1	5	5	4	4	5	2	5
1	4	4	5	1	2	2	5	5
5	2	4	4	2	2	4	4	5
3	2	4	5	3	3	4	2	3
2	2	2	2	2	2	2	2	2
1	4	3	4	2	2	2	3	3

3	3	3	3	3	3	3	3	3
2	4	2	4	2	2	2	2	4
3	1	5	5	1	4	2	4	5
5	5	5	5	5	5	5	5	5
1	1	5	5	5	5	5	1	5
2	4	5	5	2	2	4	5	5
3	4	5	5	3	3	4	4	3
1	3	4	5	1	3	4	2	4
1	1	5	5	2	5	5	1	3
3	3	3	3	3	3	3	3	3
3	3	3	3	3	3	3	3	3
2	1	5	5	4	4	4	1	4
3	2	5	5	3	5	4	1	4
4	1	4	5	4	4	4	1	5
2	2	4	4	4	4	4	2	4
2	2	4	4	3	3	4	4	3
4	2	4	5	4	4	4	3	5
1	2	5	5	5	2	5	2	2
2	2	4	4	4	3	4	2	1
4	4	4	5	5	5	5	2	5
3	1	4	4	3	4	4	3	2
1	1	5	5	5	5	5	1	5
3	3	2	2	3	3	5	4	3
3	3	3	5	3	3	3	3	3
3	4	3	4	2	2	2	4	2
1	4	2	5	2	2	3	4	4
1	3	3	3	3	2	3	4	5
4	4	1	4	1	2	1	4	5
1	1	5	5	4	3	2	2	3
3	1	5	5	3	5	5	1	4
4	4	4	4	4	4	4	2	4
2	2	5	5	3	2	5	3	4
3	1	4	4	4	5	5	3	2
1	5	1	5	1	1	1	5	4
1	1	5	5	5	4	4	1	4
3	1	4	4	4	5	4	2	5
1	1	5	4	4	3	5	1	5
2	2	5	5	5	4	5	2	4
1	1	4	4	2	4	4	4	4
5	5	5	5	5	5	5	5	5
2	1	5	5	5	4	5	2	5
4	5	2	5	1	1	2	4	5
3	3	3	3	3	3	3	3	3
3	4	4	4	3	2	2	4	3
1	1	5	3	2	3	5	3	1
2	3	5	5	5	3	5	2	4
1	5	4	4	1	1	1	3	1
3	4	5	5	4	5	5	1	2
2	2	4	4	2	4	4	3	5
3	4	4	4	2	4	4	5	4
2	1	5	5	5	5	5	1	5
5	3	5	5	5	5	5	5	5
3	4	5	5	2	2	2	5	4
2	2	4	4	4	3	4	2	3

2	1	5	4	2	5	5	2	2
2	3	4	5	4	3	3	2	3
5	1	5	5	4	5	5	1	5
3	3	4	4	3	2	3	4	4
1	1	5	5	2	4	4	4	4
4	2	4	4	4	4	4	2	4
3	3	4	4	3	3	4	2	4
4	2	4	4	4	4	4	2	4
2	4	2	2	1	2	2	4	2
1	2	4	4	4	2	3	4	1
2	3	5	5	3	4	4	2	5
2	1	5	5	5	5	5	1	5
3	2	4	3	3	3	3	3	3
1	1	5	5	5	5	5	1	4
3	2	3	4	2	4	3	3	3
3	2	5	5	4	3	4	4	5
2	1	4	5	4	4	4	2	2
2	2	4	5	3	3	3	2	2
3	3	3	3	3	4	4	4	1
5	1	5	5	5	5	5	1	3
4	4	4	5	2	3	3	4	4
4	1	5	5	2	5	5	2	5
5	2	4	4	2	4	3	2	5
2	1	5	5	2	5	5	1	1
1	5	3	5	1	3	3	3	3
3	2	3	3	2	4	3	3	2
2	2	4	4	2	3	4	2	4
3	3	3	5	3	3	3	3	3
1	1	1	4	4	3	1	4	1
5	1	5	5	5	5	5	1	1
3	2	4	3	2	3	4	3	2
4	1	5	5	4	5	5	1	4
1	1	5	5	3	5	5	1	3
1	3	4	5	2	3	4	2	4
3	3	1	1	1	1	1	2	4
2	1	5	5	4	5	5	2	1
3	3	4	4	3	4	3	3	4
2	1	4	4	3	4	5	2	3
2	2	4	5	2	4	4	3	3
4	4	5	5	5	5	4	2	4
4	1	4	4	4	4	4	3	4
2	2	4	4	2	2	4	2	2
1	1	5	5	2	5	5	4	1
3	2	4	5	2	4	4	2	3
3	2	4	4	3	4	3	3	3
3	2	4	4	2	3	3	3	4
3	3	3	3	3	3	3	3	3
2	1	5	5	4	5	5	1	5
3	1	5	5	5	5	5	1	4
5	1	5	5	5	5	5	1	5
2	3	4	4	4	5	5	1	4
1	3	4	4	4	4	3	1	2
3	3	3	3	3	3	3	3	3
4	1	5	5	3	4	5	2	5

3	3	3	3	3	3	3	3	3
5	5	5	3	3	3	4	4	4
3	1	4	5	5	4	4	4	4
2	2	4	5	2	3	4	2	5
1	5	1	5	2	4	5	3	5
2	2	5	5	5	5	5	5	5
4	3	3	4	4	4	5	5	4
2	2	5	5	2	5	5	4	5
2	2	4	4	2	4	4	2	4
2	2	4	4	2	3	4	2	2
3	3	5	5	5	5	5	5	5
5	1	5	5	5	5	5	3	5
1	1	1	1	1	1	1	1	1
2	2	5	3	3	5	5	3	1
4	3	2	4	2	3	4	3	4
5	2	5	5	5	4	5	5	5
3	3	3	3	3	3	3	3	3
3	3	3	3	3	3	3	3	4
2	4	4	5	4	4	3	4	5
3	3	4	5	4	4	4	4	5
4	3	4	4	4	3	4	3	4
4	4	4	4	4	4	4	4	4
3	1	5	5	4	5	5	1	4
5	5	5	5	5	5	5	5	5
4	3	3	3	3	3	3	3	4
3	3	5	5	3	3	3	3	3
4	3	3	4	3	4	3	3	4
3	1	5	5	5	5	5	3	5
5	5	5	5	5	5	5	5	5
1	1	5	5	4	5	5	2	4
3	3	3	3	3	3	3	3	3
1	5	3	1	1	1	1	5	4
1	4	2	4	2	3	3	3	2
1	1	5	5	4	5	5	5	5
3	2	4	4	3	3	3	2	5
3	3	4	4	3	4	4	3	4
1	2	4	5	4	3	4	4	2
1	1	5	5	2	4	4	3	2
4	4	4	4	4	4	4	4	4
3	3	2	3	1	3	5	3	3
4	2	2	4	4	4	4	2	4
3	1	2	5	3	3	3	3	4
5	4	5	4	1	4	3	3	5
5	1	5	5	5	5	5	1	4
3	3	5	5	5	5	5	1	3
3	2	4	4	4	3	3	3	3
4	2	4	4	4	5	4	2	5
3	2	4	4	3	3	4	2	4
3	3	5	3	2	4	5	2	1
3	2	4	4	4	4	4	2	5
3	3	4	3	3	4	4	4	4
2	1	5	5	5	5	5	1	1
3	3	3	3	3	3	3	3	3
3	2	4	4	2	4	4	2	4

2	1	5	5	5	5	5	2	5
1	1	5	5	3	5	5	1	5
3	4	5	5	2	2	2	4	5
3	3	3	4	3	3	3	3	3
3	3	3	3	3	3	3	3	3
1	1	5	5	3	5	5	1	5
1	1	5	5	5	5	5	1	1
4	4	4	4	4	4	4	4	4
1	5	5	5	4	3	2	1	1
5	5	3	3	1	1	1	4	5
3	3	4	4	4	4	4	2	4
2	2	5	5	2	4	4	4	4
2	2	2	2	2	2	2	4	4
4	2	5	5	2	4	3	2	4
1	1	5	5	5	4	5	2	4
5	2	5	5	5	5	5	2	5
3	3	3	3	3	3	3	3	3
2	3	4	5	2	2	2	4	5
4	1	5	5	4	5	5	1	4
2	3	4	4	3	3	3	4	3
2	1	4	3	4	4	4	2	2
4	2	4	5	3	3	4	4	4
2	3	5	5	1	4	4	3	4
3	2	4	3	2	4	4	4	4
4	3	5	5	5	5	5	5	1
1	5	5	5	1	1	1	5	1
2	2	4	4	4	4	4	4	3
3	2	4	4	3	3	4	3	4
2	5	5	5	5	5	5	1	1
2	1	5	5	4	5	5	1	5
1	1	5	5	4	5	5	1	5
4	2	4	5	3	3	4	3	5
1	4	2	4	2	2	2	3	2
5	5	5	5	5	5	5	5	5
3	3	3	3	3	3	3	3	3
4	3	4	4	4	4	5	3	5
2	1	5	5	5	5	4	5	4
3	2	5	5	3	4	4	2	4
1	4	5	5	1	1	1	4	2
3	4	4	5	3	2	1	4	4
2	2	4	4	4	4	4	2	4
3	3	3	4	3	3	3	3	2
2	2	4	4	3	3	4	3	3
4	4	4	4	4	4	4	4	4
3	2	4	4	2	2	4	3	3
2	3	4	4	3	3	2	3	4
4	2	4	4	4	4	4	1	4
2	2	4	5	4	4	4	1	4
2	2	5	4	3	4	4	2	3
3	1	5	5	4	3	3	4	4
2	3	4	3	3	2	3	4	2
2	1	5	5	3	4	4	2	4
2	4	5	5	2	4	4	4	2
2	4	3	4	2	3	2	4	4

4	1	5	4	3	4	4	3	5
3	2	4	4	4	4	4	4	4
3	3	5	5	3	3	3	3	3
5	4	4	4	2	4	4	5	5
4	4	4	4	4	4	4	4	4
2	3	2	4	2	2	2	4	2
4	2	4	5	4	5	5	2	2
1	3	3	4	2	2	4	4	2
4	3	4	4	2	4	3	3	4
3	3	4	4	3	3	4	4	3
5	3	4	5	2	3	3	2	5
3	2	4	5	4	4	4	2	4
4	4	4	4	4	4	4	4	4
3	3	4	4	4	4	4	3	4
4	3	4	4	3	4	4	3	4
4	4	4	4	4	4	4	4	4
4	4	4	4	4	4	4	4	4
4	1	5	5	4	4	4	2	5
3	3	4	4	3	3	3	3	4
3	3	3	3	3	3	3	3	3
1	1	3	4	2	4	4	2	4
3	3	3	3	3	3	3	3	4
3	2	5	5	5	4	5	3	3
3	3	4	5	4	3	3	3	5
3	2	4	4	2	4	4	2	4
2	2	5	5	2	5	5	2	5
4	2	4	5	4	4	4	3	3
2	4	4	4	2	2	2	4	4
5	3	4	4	2	3	4	4	5
3	2	4	4	4	4	4	2	4
3	1	4	4	3	4	4	3	4
4	2	4	4	4	3	4	2	4
2	2	4	4	3	4	4	4	3
3	3	4	5	4	4	4	3	5
4	2	4	5	4	4	4	2	4
2	3	5	5	2	3	3	4	4
1	5	2	4	2	2	2	4	5
3	4	4	4	2	3	3	3	3
5	2	4	4	2	4	2	2	4
1	1	5	5	5	3	5	5	5
2	5	5	5	2	2	2	4	4
3	3	3	3	3	4	3	3	3
2	4	3	3	2	2	3	4	3
2	4	3	4	3	3	3	4	4
4	2	1	4	2	2	3	4	5
3	3	4	4	3	4	4	2	3
2	3	5	4	2	3	3	4	2
5	1	5	5	5	5	5	5	5
2	2	4	4	4	4	4	2	2
4	2	5	4	4	4	3	4	4
3	1	5	5	5	5	5	2	3
2	3	2	3	4	4	4	2	4
4	2	4	4	2	3	4	2	4
3	2	3	3	3	3	3	3	3

2	2	4	4	4	4	4	2	4
5	2	5	4	4	5	5	1	4
4	2	5	5	4	4	4	4	5
3	3	3	4	3	2	2	4	3
3	4	4	4	2	2	2	4	4
5	1	5	5	5	5	5	2	4
3	2	4	4	3	4	4	3	4
1	3	3	4	1	2	2	2	1
2	2	4	4	2	4	4	2	4
1	5	3	5	2	3	2	2	2
3	3	4	5	3	3	3	2	4
3	2	4	5	2	4	4	2	5
3	2	4	5	2	3	3	2	3
3	4	5	5	2	3	4	4	2
3	1	5	5	2	3	3	3	3
3	2	4	4	2	3	3	2	4
4	4	4	4	2	2	3	4	4
1	4	4	4	3	3	5	3	4
5	1	5	5	5	5	5	5	5
5	1	5	5	5	5	5	1	3
4	2	4	4	4	4	5	4	5
2	1	5	5	2	5	5	1	3
2	1	5	4	4	4	4	2	4
1	1	5	5	5	5	5	2	4
1	5	2	5	1	1	1	1	1
2	3	3	3	2	4	3	3	2
3	1	5	5	5	4	5	3	3
1	4	5	5	3	3	3	4	2
3	3	4	4	3	3	4	3	4
3	3	3	5	3	3	3	3	3
3	1	5	5	3	4	4	2	3
3	1	5	5	3	3	3	3	3
2	1	5	5	3	4	4	2	4
1	5	2	1	1	1	1	5	5
2	4	4	4	3	2	3	4	5
3	4	4	4	2	2	2	4	2
2	2	2	2	2	2	2	2	2
2	3	4	4	3	3	4	4	4
3	1	5	4	4	4	4	3	4
3	4	3	4	1	3	2	5	5
5	2	5	5	1	2	5	1	5
3	2	4	4	4	3	3	3	3
2	4	3	5	2	2	2	4	5
1	2	4	5	1	1	4	5	1
4	4	4	4	4	4	4	4	4
2	2	4	4	4	4	4	4	2
2	2	4	5	2	4	4	2	4
2	5	5	3	3	3	1	1	4
1	1	5	5	5	5	5	2	1
3	2	5	5	3	4	4	3	4
5	2	5	5	5	5	5	5	5
3	3	3	5	3	4	3	3	3
4	4	4	4	2	3	3	4	4
2	3	4	4	3	3	4	2	2

2	3	4	4	2	2	4	4	2
3	3	4	4	3	4	3	2	4
3	2	5	5	5	3	5	5	5
3	1	4	4	3	4	4	2	4
5	4	3	5	1	1	2	5	1
2	3	4	4	2	3	3	2	4
3	2	5	5	4	5	4	2	5
2	3	2	5	2	3	3	3	3
5	1	5	5	4	5	5	1	5
1	1	5	5	5	5	5	1	1
4	2	4	5	3	4	4	4	4
5	5	5	5	4	2	3	2	2
2	1	4	5	3	5	5	3	1
2	3	4	4	4	4	4	4	3
2	2	4	4	3	4	4	3	2
5	1	5	5	5	5	5	1	5
3	2	4	4	3	4	4	4	4
3	3	3	3	2	2	4	2	5
3	4	5	5	2	4	4	4	3
2	2	4	5	3	4	4	4	3
3	3	4	5	3	2	3	4	4
4	2	4	4	4	4	4	2	4
3	3	4	3	3	4	3	3	4
2	4	4	4	3	3	3	4	2
3	2	4	4	3	2	3	4	4
1	5	5	5	1	1	3	5	1
3	2	5	4	3	4	4	1	4
4	4	4	5	2	3	3	4	5
5	5	5	5	5	5	5	5	5
2	4	4	4	2	2	2	4	2
4	3	4	4	3	3	4	3	3
5	1	5	5	5	5	5	2	5
2	3	4	4	2	3	3	4	3
2	2	3	4	3	4	4	2	4
1	1	5	5	5	5	5	1	5
3	3	4	4	2	3	3	3	4
5	1	5	5	2	4	4	2	4
4	4	4	4	4	4	4	4	4
2	3	3	4	4	2	2	4	4
4	2	4	4	4	4	4	3	4
2	3	4	4	4	3	3	2	3
2	4	5	5	3	2	5	2	5
2	4	3	5	2	2	2	4	4
4	2	4	4	3	4	4	2	4
3	1	5	5	2	4	5	2	4
3	1	5	5	3	3	4	3	4
3	2	5	5	3	3	3	3	2
2	2	4	4	4	4	4	2	4
2	5	3	3	2	2	2	2	2
3	3	3	3	3	3	3	3	3
4	2	4	4	4	4	4	2	2
3	3	3	3	3	3	3	3	3
4	3	4	4	4	4	4	3	4
4	4	4	4	2	2	4	4	5

3	3	3	3	4	4	4	4	4
5	3	4	5	5	5	5	5	5
3	3	4	4	4	3	2	2	3
4	2	4	1	1	2	2	4	2
3	4	4	4	4	3	4	2	4
2	2	4	4	3	4	4	2	4
4	5	3	4	2	2	2	5	5
2	4	4	3	3	1	2	4	5
1	5	4	5	3	2	1	5	1
1	4	3	3	3	3	3	3	3
3	2	5	5	2	3	4	5	5
3	1	5	5	4	4	4	3	5
3	3	3	3	4	4	4	3	4
3	2	4	5	2	2	3	4	4
4	2	4	5	4	4	4	2	4
2	2	5	5	2	4	4	3	2
2	4	4	4	3	2	3	4	4
3	3	3	3	2	3	2	4	3
4	1	5	4	4	3	4	1	5
2	3	4	5	3	3	4	4	2
2	2	3	3	3	2	2	4	4
4	4	4	4	4	4	4	4	4
1	3	4	1	2	3	1	3	4
3	3	4	3	3	2	3	3	3
4	1	5	5	4	5	5	2	4
1	5	4	5	1	1	5	4	4
4	3	4	4	4	4	3	2	5
4	2	4	4	4	4	4	2	4
2	2	4	4	3	2	3	4	4
3	2	4	4	3	3	4	2	2
5	5	5	5	5	5	5	5	5
2	2	4	5	5	3	4	2	2
2	2	2	2	2	2	2	2	2
4	4	4	4	4	4	4	4	4
4	2	5	4	4	4	4	2	4
3	3	3	3	3	3	3	3	3
5	1	5	4	5	5	5	5	5
5	2	4	5	4	5	5	1	3
3	4	4	4	3	3	4	4	3
3	1	5	5	4	5	5	1	4
4	4	4	4	4	4	4	4	4
2	3	2	4	4	4	4	4	4
5	2	5	5	5	4	4	2	2
4	3	4	4	3	4	4	3	4
4	4	4	4	4	4	4	4	4
2	3	3	4	4	3	3	5	4
4	2	4	4	4	4	4	3	4
4	2	5	5	4	4	4	2	4
4	1	4	4	4	4	4	2	4
4	2	4	4	4	4	4	2	4
3	2	4	4	3	4	4	2	4
3	3	3	4	3	3	3	3	4
1	1	5	5	3	4	5	1	4
4	1	5	4	3	4	4	2	4

5	1	5	5	5	5	5	5	5
5	5	5	5	5	5	5	1	5
2	2	4	3	2	2	4	2	2
3	4	4	4	3	3	4	3	3
3	1	5	5	4	4	5	1	5
4	1	5	5	5	5	5	1	5
2	1	5	5	2	4	3	3	5
3	2	5	5	5	5	5	2	5
3	3	5	5	3	3	3	1	5
3	2	5	4	4	5	5	2	5
2	1	5	4	2	4	4	1	2
2	2	2	3	3	2	3	3	4
2	2	4	4	3	4	4	4	4
3	2	4	4	3	4	4	2	4
4	4	4	4	4	4	4	2	4
2	4	4	4	2	2	2	4	2
2	2	4	4	2	3	4	4	4
1	2	4	4	4	4	4	1	4
2	2	4	4	2	4	3	2	4
2	1	5	5	5	1	5	2	1
2	1	5	5	5	5	5	1	5
1	1	3	4	3	1	1	4	2
3	3	3	3	3	3	3	3	3
3	2	5	5	3	4	4	3	3
4	5	5	5	5	5	5	4	4
1	2	3	5	1	3	3	3	4
3	3	4	4	3	3	3	2	4
2	2	5	5	2	4	4	2	4
4	4	4	4	4	4	4	2	4
1	5	3	5	2	2	2	4	4
3	2	4	4	4	4	4	2	3
4	4	5	5	4	3	4	3	5
5	1	5	5	5	5	5	5	5
1	1	5	5	3	4	4	2	2
5	2	5	5	5	5	5	2	3
5	1	5	5	3	5	5	1	4
3	5	5	5	3	5	5	2	3
4	4	4	4	4	4	4	3	4
2	1	5	5	5	5	5	1	5
3	2	4	4	4	4	3	2	3
5	5	5	5	5	5	5	5	5
3	3	3	3	2	3	3	3	3
3	1	5	5	3	4	4	3	3
4	4	4	4	4	4	4	2	4
4	1	5	5	4	4	4	2	5
2	3	4	2	3	3	3	5	4
1	5	5	5	1	1	4	1	1
3	3	5	5	2	3	3	3	3
3	3	4	4	4	3	4	3	3
5	2	3	4	4	4	4	2	4
3	4	3	3	3	3	3	4	4
4	2	4	4	2	4	4	2	5
4	2	4	4	4	4	4	2	4
4	1	5	5	3	5	5	1	5

5	1	5	5	5	5	5	1	5
1	1	5	5	5	5	5	2	1
3	3	4	4	4	3	4	3	1
2	2	4	4	3	3	4	2	4
2	3	4	4	2	3	2	2	5
5	2	5	5	5	5	5	1	2
1	2	4	1	3	3	3	3	3
4	2	5	5	5	5	5	1	1
5	5	5	5	5	5	5	5	5
1	1	5	4	4	4	4	1	5
2	2	4	4	3	4	4	2	4
4	3	4	4	5	3	1	2	3
5	1	5	5	5	5	5	1	5
3	3	3	3	3	3	3	3	3
3	2	4	4	4	4	4	1	5
2	2	5	4	5	5	5	1	5
3	2	4	5	3	3	2	3	2
3	5	3	4	2	4	2	4	4
4	1	1	2	3	3	4	2	3
2	2	4	4	3	4	4	2	2
2	3	3	4	4	3	3	2	5
4	2	4	5	4	4	4	2	2
2	1	5	5	5	5	5	5	5
1	1	5	5	5	5	5	1	5
3	4	3	3	3	3	3	3	5
3	2	5	4	5	5	5	2	5
2	1	5	5	2	5	5	1	5
5	2	4	5	5	3	4	3	5
2	1	5	5	2	3	3	3	2
3	4	4	4	4	4	4	2	1
3	3	3	3	3	3	3	3	3
4	1	5	5	5	5	5	1	5
4	2	4	4	4	4	4	2	4
3	1	5	5	3	4	5	1	5
2	1	4	4	2	3	3	4	5
2	3	2	5	2	2	2	5	4
2	3	3	3	3	3	3	4	4
1	1	5	5	5	5	5	1	5
4	4	5	3	5	5	5	2	2
2	1	5	3	4	3	4	2	4
2	2	4	4	4	4	4	2	4
1	1	5	5	5	5	5	1	3
5	2	5	5	3	3	5	3	5
4	1	5	5	5	5	5	1	5
3	3	3	3	3	3	3	3	3
2	2	3	4	3	4	4	2	3
3	1	5	4	4	5	4	1	3
1	3	5	5	2	4	5	2	5
4	3	4	4	2	2	2	4	4
4	1	5	5	5	5	5	1	5
3	3	3	3	3	3	3	3	3
4	1	5	5	4	4	5	2	5
5	1	5	5	5	5	5	1	5
2	3	4	5	2	2	5	5	5

3	3	3	3	3	3	3	3	3
3	2	4	4	4	4	4	2	4
4	1	4	5	4	4	4	1	4
3	3	3	3	3	3	3	3	3
5	3	5	5	5	5	5	5	5
1	1	5	5	4	5	5	1	5
5	5	5	5	5	5	5	5	3
2	2	4	4	5	2	2	2	1
4	1	4	4	5	5	4	2	1
5	1	5	5	5	5	5	1	5
5	1	5	5	5	5	5	1	5
3	2	4	4	4	3	3	2	4
5	1	5	5	5	5	5	1	5
5	5	1	5	1	1	1	1	4
2	2	5	5	3	3	3	2	3
4	2	4	4	4	4	5	5	4
2	1	5	5	3	3	3	3	3
4	1	5	5	4	5	5	1	4
4	2	4	4	4	4	4	2	4
3	3	3	4	3	4	4	2	3
3	2	5	5	5	5	5	1	3
3	3	3	3	3	3	3	3	3
5	5	5	5	5	5	5	5	5
3	1	4	5	5	5	5	1	5
3	3	3	3	3	3	3	3	2
2	4	2	2	3	3	3	3	3
3	1	2	4	2	2	3	2	5
2	3	3	5	2	2	3	3	4
2	2	4	4	4	4	4	2	4
4	3	4	4	3	3	3	3	5
2	2	4	4	2	4	4	2	4
2	2	3	4	2	2	3	3	2
4	2	4	4	4	4	4	2	4
2	2	4	4	2	3	3	3	3
3	3	3	3	3	3	3	3	3
2	2	4	4	2	2	4	2	4
2	3	4	4	2	4	3	3	4
2	2	4	4	5	4	3	3	3
4	2	4	4	4	4	4	3	4
3	3	3	3	3	3	3	3	3
2	4	4	4	3	3	4	4	4
2	2	5	5	5	5	5	2	3
4	1	5	5	4	4	4	2	4
2	4	3	3	2	2	3	5	5
2	3	3	4	2	3	3	3	4
5	1	5	5	5	5	5	1	5
2	4	3	4	2	2	3	4	4
4	2	4	4	4	4	4	2	4
4	2	4	4	4	4	4	3	4
2	3	4	4	2	3	4	3	3
3	2	4	5	3	4	3	2	4
4	2	4	4	4	4	4	2	2
3	3	3	3	3	3	3	3	3
4	3	5	4	2	3	3	3	4

2	3	5	5	2	3	4	3	5
3	3	4	5	4	2	2	4	4
1	1	3	5	3	3	3	3	2
3	2	4	4	3	4	4	2	4
2	4	3	3	3	2	2	4	4
1	4	5	5	5	1	4	5	4
4	2	2	4	2	2	3	5	5
2	3	3	3	2	3	3	3	3
3	1	5	5	2	5	3	2	5
5	5	5	5	5	5	5	2	5
4	2	4	4	2	4	4	2	4
1	1	1	1	1	1	1	1	1
2	2	4	5	4	4	5	2	2
2	4	4	5	2	2	3	4	3
3	2	4	4	5	5	5	2	2
3	3	5	4	3	2	2	3	5
3	2	2	4	4	4	4	3	4
4	5	3	4	3	2	1	4	5
3	2	4	4	2	3	4	3	4
1	4	2	1	3	2	3	4	1
5	5	5	5	5	5	5	5	5
3	3	3	3	3	3	3	3	3
2	3	4	4	3	4	3	3	4
4	3	4	5	4	3	3	3	2
4	2	4	4	4	4	4	2	4
2	4	5	5	2	2	4	5	5
3	3	4	4	3	3	3	3	4
1	5	1	5	1	1	1	5	2
2	4	4	4	2	2	2	4	2
1	2	2	5	2	2	4	4	3
3	1	4	5	4	3	4	3	4
3	2	4	4	3	3	4	3	4
2	2	4	4	2	3	4	3	3
5	1	1	5	5	5	5	5	5
1	3	4	4	4	4	4	4	3
2	2	4	4	4	4	4	2	2
3	4	4	4	3	4	4	3	4
3	3	3	3	3	3	3	4	4
2	4	4	4	2	2	2	2	4
2	3	3	4	2	2	3	3	2
3	1	5	5	4	5	5	1	3
3	2	4	4	4	4	4	2	3
4	3	3	5	4	4	4	4	4
2	2	3	4	3	3	3	2	2
4	4	4	5	3	3	4	2	5
2	3	4	4	2	2	2	3	4
4	2	4	5	2	3	4	3	4
2	3	3	4	2	3	3	3	2
5	3	2	2	3	3	3	3	1
2	2	4	4	3	3	3	3	4
4	2	4	4	2	2	4	2	4
4	1	5	5	2	5	5	2	5
3	1	4	5	3	3	3	2	4
1	1	5	5	4	2	5	2	4

2	3	3	4	2	4	3	3	4
4	2	4	4	4	4	4	4	4
3	3	4	4	3	3	4	3	4
3	3	4	4	3	3	3	3	4
4	3	4	4	4	4	3	3	3
2	2	4	4	3	3	4	3	3
3	4	4	5	3	5	3	5	5
3	1	5	5	2	5	5	3	5
2	2	5	3	3	5	5	2	1
1	1	4	5	2	4	4	2	1
1	1	5	5	3	5	5	1	1
4	1	4	4	2	4	3	3	5
1	1	5	5	5	3	3	2	3
4	2	4	4	4	4	4	2	4
2	1	5	4	3	5	5	2	3
3	2	4	4	2	2	4	2	4
2	1	5	5	3	5	5	1	4
4	2	4	4	3	4	4	2	4
2	3	4	4	5	3	4	3	2
3	5	2	5	1	2	2	5	5
2	2	5	5	4	3	4	1	4
2	3	4	4	3	3	3	2	4
1	1	5	5	5	5	5	5	5
1	1	5	5	5	5	5	1	2
5	1	5	5	5	5	5	1	5
1	4	4	5	3	2	2	5	5
4	1	5	5	4	4	4	2	4
1	1	5	5	5	5	5	1	5
4	3	4	5	4	3	3	3	5
1	1	5	5	5	5	5	1	5
5	2	4	5	4	4	3	3	5
1	1	5	3	4	4	5	4	3
2	3	3	3	3	3	3	3	5
1	1	5	5	3	5	5	1	3
2	3	5	5	2	3	3	4	4
5	2	5	5	2	4	4	4	5
2	2	4	4	2	3	4	3	2
1	5	2	3	1	1	1	5	3
3	3	3	3	3	3	3	3	3
3	2	5	5	2	4	4	1	5
4	1	5	5	4	5	5	1	5
4	3	4	4	4	4	4	3	4
3	3	3	3	3	3	3	3	3
3	3	4	3	3	3	4	2	4
3	4	4	3	2	2	2	2	3
2	4	5	5	2	2	3	3	4
2	1	5	5	3	3	4	4	2
3	3	4	4	3	3	3	4	4
4	2	4	5	4	4	4	2	5
2	2	4	5	3	3	4	3	4
1	5	3	5	5	2	4	2	5
4	1	5	5	3	4	5	1	5
2	3	4	5	4	4	4	3	1
3	1	5	5	3	5	5	2	5

2	1	3	3	4	3	4	4	5
3	2	5	5	2	5	5	5	4
1	2	3	4	3	3	3	2	5
1	5	1	1	1	1	1	5	3
4	3	5	5	2	2	3	3	3
4	4	3	3	1	2	2	3	2
3	3	4	4	2	3	2	3	4
5	4	3	5	1	3	3	2	4
2	1	4	4	4	3	4	3	2
3	3	3	4	1	2	3	3	4
3	2	5	5	4	5	5	3	5
1	3	3	3	3	3	3	4	2
1	3	4	5	3	4	4	2	5
4	1	5	5	5	5	5	1	4
4	1	5	5	5	5	5	1	5
3	3	4	3	3	3	3	3	3
2	2	4	5	3	4	4	4	1
3	1	5	5	3	4	5	2	4
2	1	4	4	3	4	4	3	2
3	3	4	4	2	2	4	2	4
4	3	4	4	4	3	3	4	4
2	4	3	3	2	2	4	3	3
5	5	5	5	5	5	5	5	5
2	3	3	5	5	5	5	5	2
4	3	3	5	3	2	4	4	4
3	3	3	3	3	3	3	3	4
2	3	3	3	3	3	3	3	3
3	1	5	4	4	4	4	1	4
3	3	3	3	3	3	3	3	3
2	2	4	4	4	4	4	3	2
3	3	3	3	3	3	3	3	3
2	4	3	4	3	2	3	4	2
2	4	4	4	2	2	3	3	4
3	3	3	4	3	4	4	3	3
3	3	4	4	4	4	4	3	3
3	2	4	4	4	4	4	2	4
4	2	5	5	4	4	4	2	5
3	2	3	3	3	4	4	2	4
3	2	4	4	4	4	4	3	3
3	3	3	3	3	3	3	3	3
1	2	4	4	4	4	4	2	3
3	2	5	4	2	4	4	3	2
3	2	4	3	3	3	3	3	4
2	2	4	5	4	4	4	2	2
3	3	3	3	2	3	2	3	2
5	4	5	4	5	5	4	4	4
2	1	4	5	3	5	4	2	2
2	1	4	5	3	4	4	2	2
2	2	4	4	3	4	4	2	4
3	2	4	5	4	4	4	2	3
3	1	5	5	4	5	4	1	5
2	2	5	4	2	4	4	3	4
4	1	5	5	4	4	5	4	5
2	1	5	5	2	5	4	2	4

3	2	4	4	3	4	3	3	3
2	2	4	5	4	4	4	2	2
3	2	4	4	3	3	4	4	4
2	2	5	5	4	5	5	4	4
2	1	5	5	4	4	5	1	4
2	1	5	5	2	5	4	2	4

C33	C34	C35	C36	C37	C38	C39	C40	C41
5	5	5	5	5	5	1	5	5
3	1	4	5	4	4	5	4	4
5	5	5	5	5	5	5	5	5
5	5	5	5	5	5	5	5	5
3	3	3	3	3	3	3	3	3
5	5	5	5	5	5	5	5	5
5	5	5	5	5	5	5	5	5
5	5	4	5	3	5	4	5	5
4	4	4	4	4	4	4	2	2
5	5	4	5	5	4	3	5	5
3	2	5	4	5	5	4	3	2
2	2	3	4	4	3	3	3	2
2	2	5	5	5	5	2	2	1
5	5	5	5	5	5	5	5	5
3	5	5	3	5	2	5	3	5
5	5	5	5	5	5	5	5	5
1	1	4	3	3	3	4	4	1
5	5	5	5	5	5	5	5	3
2	2	4	4	3	4	3	3	2
2	1	4	3	5	5	3	2	3
5	5	5	5	5	5	5	5	5
5	5	5	5	3	5	5	5	5
1	1	5	5	5	5	1	4	1
1	1	5	4	5	5	1	1	1
3	4	4	4	2	2	4	4	4
2	2	4	4	4	4	2	2	2
1	1	3	3	3	3	1	1	3
3	2	4	4	3	4	3	3	2
5	1	5	4	4	4	3	2	1
3	2	4	3	2	4	4	3	4
1	1	5	5	5	5	2	2	1
1	1	5	5	5	5	1	1	1
1	1	5	4	4	4	2	2	2
5	5	5	4	5	5	5	5	4
1	1	5	4	4	4	3	3	1
3	3	3	3	3	3	3	3	1
2	2	5	1	5	5	5	5	2
5	5	5	5	5	5	5	5	5
1	2	5	5	5	5	2	5	1
1	1	5	5	5	5	2	5	1
1	1	4	1	4	4	2	2	1
1	1	4	4	4	4	2	4	2
1	1	1	5	5	5	2	5	2
1	1	5	3	4	3	2	2	1
2	3	4	4	4	4	4	4	2
4	4	4	4	4	4	4	4	4
1	2	5	4	5	5	1	3	2
5	5	5	5	5	5	5	5	5
3	2	3	2	2	2	2	2	2
4	4	1	4	5	3	2	4	2
5	5	5	5	5	5	5	5	5
1	1	5	5	5	5	3	5	1
3	2	4	3	5	3	3	5	3

3	3	3	3	3	3	3	3	3
3	2	4	3	3	4	3	4	3
5	5	5	4	3	3	3	3	3
5	5	5	5	5	5	5	5	5
5	2	5	3	5	5	2	5	2
2	1	5	4	4	4	1	4	1
1	1	5	5	5	5	1	2	1
1	1	5	5	5	5	1	5	5
3	2	4	4	4	4	4	2	2
2	2	5	4	4	4	5	4	4
2	2	4	2	2	4	2	2	2
4	2	5	3	4	4	4	4	3
1	1	5	5	5	5	3	3	1
2	5	5	5	3	4	4	3	2
2	2	4	4	4	4	2	4	2
3	1	5	4	5	5	2	3	1
1	1	1	5	5	5	1	1	1
1	2	5	3	5	5	1	2	1
1	1	1	4	4	4	3	2	1
3	2	4	3	3	4	3	3	2
2	1	4	4	4	5	2	4	2
2	2	4	3	4	4	4	4	2
3	3	5	3	5	5	3	3	3
3	3	3	4	3	3	3	3	3
3	3	3	3	3	3	3	3	3
3	2	3	1	2	2	4	5	4
2	1	4	3	4	5	3	4	2
3	1	3	3	3	3	3	3	3
3	2	3	3	3	3	3	3	2
3	3	3	3	3	3	3	3	3
3	3	3	3	3	3	3	3	4
3	2	4	3	3	3	4	3	2
1	1	5	5	5	5	2	2	1
3	1	3	3	3	3	3	3	3
2	2	2	3	4	4	3	4	1
1	1	5	4	5	5	2	2	2
1	1	5	5	5	5	2	1	1
2	1	5	2	3	3	2	2	1
4	4	3	3	2	5	3	4	5
1	1	5	3	5	5	3	5	1
4	2	4	2	2	3	3	3	3
2	2	4	4	4	4	2	2	2
2	3	3	3	3	3	3	3	2
1	1	5	5	4	5	1	3	1
1	1	5	4	4	4	4	4	2
3	3	5	5	4	3	1	3	3
4	4	5	3	4	4	4	4	5
4	2	5	3	3	3	3	3	3
1	1	5	4	5	5	2	3	1
5	1	4	3	3	2	5	3	4
4	2	4	4	4	4	4	4	4
2	2	4	4	5	5	1	3	1
2	2	2	2	2	2	2	2	2
4	2	4	2	3	2	4	4	4

3	3	3	3	3	3	3	3	3
4	5	4	1	1	1	5	2	4
3	1	5	4	4	4	4	4	1
1	1	5	5	5	5	5	5	1
1	5	5	5	5	5	3	1	1
4	1	5	4	5	5	5	4	4
3	2	5	3	3	4	5	4	3
2	2	5	3	5	5	2	4	2
1	1	5	5	5	5	2	4	1
3	3	3	3	3	3	3	3	3
3	3	3	3	3	3	3	3	3
1	1	5	5	4	4	2	4	1
1	1	5	2	4	4	1	1	1
2	1	5	1	4	5	1	4	1
2	2	4	2	4	4	2	2	2
2	2	4	3	3	4	4	2	2
2	1	5	4	5	5	3	4	2
2	2	4	2	5	5	2	5	2
1	1	4	2	4	5	1	1	2
2	2	2	3	2	4	4	4	4
2	1	3	4	4	4	4	2	2
1	1	5	5	1	5	1	5	1
2	2	3	3	3	3	3	3	3
3	3	3	3	3	3	3	3	3
4	2	2	2	2	2	4	4	4
4	1	4	2	1	3	3	2	3
4	2	3	3	2	3	5	3	3
4	2	4	2	3	3	4	3	4
3	3	4	2	3	3	2	4	2
1	1	5	4	5	5	2	1	1
2	2	4	4	2	4	2	2	1
2	2	5	3	4	5	2	2	3
2	3	4	3	2	3	4	3	5
1	1	5	1	1	1	5	1	5
2	1	1	3	4	4	2	2	2
1	1	5	4	4	4	2	1	1
1	3	4	3	4	4	2	2	1
2	1	5	2	4	4	4	2	2
1	1	5	3	3	4	3	1	1
3	4	4	4	5	4	5	5	4
2	1	5	3	5	5	3	4	1
4	3	3	1	1	5	5	3	5
3	3	3	3	3	3	3	3	3
5	5	4	3	3	3	4	4	5
1	1	1	3	3	5	3	3	1
1	1	5	5	5	5	4	4	1
5	2	4	1	1	1	4	1	3
1	1	5	5	4	5	1	3	1
1	1	5	4	4	5	4	2	2
4	2	4	2	2	2	4	3	4
1	1	5	5	5	5	2	1	1
3	5	5	3	5	5	5	4	3
3	2	4	2	4	4	3	3	4
2	2	4	3	4	4	2	2	2

1	1	4	3	5	5	4	1	1
3	3	4	3	3	4	3	3	1
1	1	5	5	5	5	1	5	1
3	2	4	3	2	3	3	3	3
1	1	4	5	2	5	3	1	1
2	2	4	4	4	4	4	2	2
2	2	4	4	4	4	3	3	2
2	2	4	4	4	4	2	2	2
4	3	3	2	2	2	4	3	4
2	1	5	5	2	3	2	3	1
3	2	4	3	5	5	2	5	3
1	1	5	1	5	5	1	1	1
3	3	3	3	3	3	3	3	3
1	1	1	5	5	5	1	3	1
2	2	3	3	3	3	3	3	2
3	1	4	4	4	4	4	4	1
1	1	5	4	4	4	4	4	1
2	2	4	4	4	3	3	2	2
4	2	4	2	3	2	5	5	4
1	1	5	1	4	5	3	5	1
4	2	5	3	3	3	4	4	4
1	1	5	5	5	5	2	4	1
2	1	5	3	5	5	4	1	1
1	1	5	3	4	5	2	4	1
3	3	3	3	3	3	3	3	3
3	3	4	2	3	5	2	4	4
2	2	2	3	4	4	3	2	2
3	3	3	3	3	3	3	3	3
4	4	4	1	1	1	5	1	5
5	5	1	1	4	5	1	1	1
2	2	4	3	4	4	4	4	2
1	1	5	5	5	5	1	2	1
1	1	5	5	3	5	1	1	1
2	1	3	2	3	4	1	1	1
1	1	5	4	3	3	3	3	3
1	1	5	5	4	3	2	4	1
3	2	4	3	3	4	4	4	3
1	1	3	4	5	5	2	4	1
2	2	3	4	4	4	2	4	1
1	1	4	5	5	5	4	4	2
3	1	5	3	5	5	4	4	3
2	2	4	4	4	4	4	4	2
2	1	5	5	5	5	5	2	5
2	2	4	3	2	4	2	3	2
3	2	3	3	3	3	3	2	3
2	1	4	3	3	3	4	4	2
3	3	3	3	3	3	3	3	3
1	1	5	5	5	5	1	2	1
1	1	5	5	5	5	4	4	1
1	1	5	5	5	5	1	1	1
1	1	4	4	2	4	2	2	1
1	3	3	3	4	3	2	1	1
3	3	2	3	3	3	3	3	3
2	1	5	5	5	5	3	4	1

3	3	3	3	3	3	3	3	3
3	4	3	4	3	4	2	2	1
1	1	4	4	4	4	4	4	1
2	1	4	2	4	3	4	1	2
5	1	5	3	3	5	2	5	1
5	1	5	5	5	5	2	5	5
5	4	4	4	1	4	2	4	1
1	1	4	4	5	5	1	4	2
2	1	4	4	4	3	2	2	2
2	2	5	2	4	4	4	4	2
5	5	5	4	5	5	2	5	2
1	1	5	5	5	5	5	5	1
1	1	1	1	1	1	1	1	1
3	2	5	4	4	4	2	3	1
2	2	3	3	3	3	3	3	3
4	4	4	4	5	5	4	4	1
3	3	3	3	3	3	3	3	3
3	3	2	1	1	1	3	3	4
3	2	3	4	3	3	3	2	2
3	1	5	4	4	4	4	4	3
2	2	4	4	4	4	4	4	2
3	2	4	4	4	4	4	4	2
2	1	5	5	5	5	2	2	1
5	1	5	5	3	5	1	1	1
3	1	3	3	3	3	3	4	3
3	3	4	3	3	3	3	3	3
3	2	4	2	3	3	3	3	3
1	1	5	5	5	5	3	3	1
5	5	5	5	5	5	5	5	5
1	1	5	4	4	5	4	1	1
3	3	3	3	3	3	3	3	3
5	3	4	1	1	1	5	3	5
4	4	4	2	2	3	4	3	4
5	5	5	5	5	5	5	5	1
2	1	5	3	5	4	3	2	1
2	1	4	3	4	4	4	1	2
2	1	5	4	3	4	3	3	2
1	1	5	3	5	5	2	3	1
4	4	4	4	4	4	4	4	4
4	1	1	5	1	5	1	4	1
2	2	2	4	4	4	2	4	4
3	1	4	3	3	4	4	3	4
2	3	5	3	3	5	5	4	2
1	1	5	5	5	5	3	5	5
3	3	3	3	3	4	1	3	1
3	2	4	3	3	4	3	3	2
3	1	4	3	2	4	4	4	2
2	2	3	3	4	4	2	4	2
1	1	5	3	5	5	2	1	1
2	4	1	4	1	2	4	4	1
2	2	4	3	4	4	2	3	4
1	1	5	1	5	5	1	5	1
3	3	3	3	3	3	3	3	3
2	2	4	3	4	4	2	4	2

1	1	1	5	5	5	2	2	1
1	1	5	1	1	1	1	1	2
2	2	4	4	4	4	4	3	2
3	3	3	3	3	3	3	3	3
3	3	3	3	3	3	3	3	3
1	1	5	3	1	5	1	1	1
1	1	5	5	5	5	1	1	1
4	4	3	3	4	4	4	4	2
3	1	4	2	1	3	1	1	1
5	3	3	1	1	3	5	1	5
2	2	4	3	4	4	3	4	2
1	1	5	2	4	4	5	4	2
4	2	4	2	4	4	4	4	4
1	2	3	3	3	4	2	3	4
1	1	3	2	5	5	2	1	1
2	2	5	5	5	5	2	5	2
3	3	3	3	3	3	3	3	3
2	2	5	3	2	5	5	4	2
1	1	5	5	5	5	1	5	1
3	2	2	2	2	3	3	3	3
2	2	4	4	4	4	4	4	4
2	2	4	3	4	3	4	4	2
1	1	5	1	4	4	5	3	1
3	4	3	4	2	4	4	4	2
2	2	5	3	5	5	2	1	3
5	1	5	1	1	5	5	1	5
2	1	4	4	5	4	4	4	1
2	2	3	3	4	4	3	4	4
5	5	5	2	5	3	3	5	1
1	1	5	4	5	5	2	5	1
1	1	1	5	5	5	1	3	1
3	2	4	3	4	4	4	4	3
2	1	1	1	1	1	1	2	1
5	5	5	4	5	5	5	5	5
3	3	3	3	3	3	3	3	3
4	1	4	3	3	4	4	4	3
1	1	5	5	5	5	5	5	1
1	1	4	3	4	4	3	3	2
4	2	5	1	4	5	2	1	4
5	3	3	3	4	4	4	4	3
2	2	4	4	4	4	4	2	2
4	3	3	3	3	3	4	3	3
2	2	4	3	3	3	3	2	2
2	2	4	3	4	4	4	4	2
2	2	3	3	4	3	3	3	2
3	2	4	2	3	2	4	2	4
2	2	4	4	4	4	2	4	2
1	1	4	2	4	4	2	4	2
1	1	4	4	4	3	2	2	1
2	1	5	2	5	3	4	4	2
2	2	4	2	2	3	3	2	3
1	1	5	4	4	3	1	2	1
3	2	5	2	5	5	5	3	4
4	3	4	2	3	2	4	4	4

1	1	5	3	4	5	2	2	1
1	1	4	4	4	4	4	2	1
3	3	3	3	3	3	3	3	3
4	1	5	5	4	4	4	4	2
4	4	4	4	4	4	4	4	2
4	4	4	2	2	2	2	2	4
2	2	5	4	4	4	2	2	2
2	2	4	2	2	2	2	2	2
2	2	4	4	3	4	4	2	2
3	3	4	3	4	3	3	3	3
3	1	4	3	4	3	4	4	2
1	1	4	4	4	4	2	2	2
4	4	4	4	4	4	4	4	4
3	2	4	4	4	4	3	4	3
2	2	4	4	4	4	4	4	2
4	4	4	4	4	4	4	4	4
4	4	4	4	4	4	4	4	4
2	2	4	4	4	4	2	2	2
3	2	4	3	3	4	3	2	2
3	3	3	3	3	3	3	3	3
2	1	4	1	4	4	2	2	2
3	3	3	3	3	3	3	3	3
1	1	5	5	5	5	3	2	2
3	1	5	4	4	4	3	2	2
2	2	4	3	4	4	2	4	4
2	1	5	5	5	5	4	5	1
3	2	4	4	4	4	1	2	2
4	3	4	2	2	2	4	2	4
2	1	5	4	3	3	4	2	2
3	2	4	3	4	4	3	2	3
1	1	5	4	4	3	2	4	2
2	2	4	4	4	4	3	4	2
3	3	4	3	4	4	4	3	2
3	3	4	3	3	4	4	3	2
2	2	4	4	4	4	4	4	2
3	2	4	2	4	2	3	2	3
4	1	2	1	4	2	4	2	5
3	3	3	3	3	3	3	3	3
2	2	2	2	2	4	2	2	3
3	1	5	1	4	5	5	5	5
4	2	5	2	2	2	4	2	4
3	3	3	3	3	4	4	4	3
4	3	3	2	3	3	4	3	3
4	1	4	2	4	4	4	4	4
3	1	5	3	3	4	1	4	3
3	2	4	4	4	4	4	4	2
3	2	4	3	4	3	4	3	2
1	1	1	1	5	5	2	2	2
1	1	4	5	5	5	2	1	1
4	2	3	4	4	5	3	4	4
1	1	5	5	5	5	1	3	1
4	1	4	1	4	2	3	3	4
4	4	4	4	4	4	4	4	4
3	3	3	3	3	3	3	3	3

2	2	4	4	4	4	3	3	1
2	1	5	5	5	5	5	5	2
4	4	2	4	4	4	2	3	4
4	3	3	3	3	3	3	3	3
4	2	4	2	4	3	4	4	4
1	2	5	5	5	5	2	4	2
1	1	4	4	3	4	3	4	1
4	1	3	1	4	4	2	1	1
2	2	4	4	4	4	2	2	2
3	3	4	3	2	4	5	5	4
2	1	4	4	4	4	3	3	3
2	2	2	3	4	4	4	1	2
2	2	3	3	4	4	2	4	2
4	2	5	3	4	3	5	2	4
1	1	5	4	3	3	3	3	1
2	2	4	2	4	3	3	3	3
4	1	4	2	2	4	3	2	3
3	3	3	3	3	2	3	3	3
1	1	5	5	5	5	5	1	1
1	1	5	5	5	5	5	4	3
2	2	5	5	5	4	4	5	4
1	1	5	4	4	4	1	1	1
2	2	5	2	4	5	3	4	2
1	1	5	4	5	5	5	3	1
5	5	5	1	1	1	1	1	1
3	2	4	2	4	3	3	3	3
1	1	5	5	5	5	3	5	1
1	1	3	2	2	5	5	4	1
3	3	3	3	3	3	3	4	3
3	3	3	3	3	3	3	3	3
1	1	5	3	5	4	3	3	1
3	3	3	3	3	3	3	3	3
1	1	5	5	5	4	2	4	1
5	4	2	1	3	1	5	1	5
3	3	4	2	4	4	4	4	4
4	2	2	2	4	3	4	2	3
2	2	2	3	1	2	2	3	2
2	1	4	4	3	3	3	2	3
2	2	4	4	4	5	3	3	2
5	2	4	2	3	2	4	4	4
3	1	5	3	2	5	1	5	3
3	3	3	3	3	3	3	3	3
5	2	4	2	3	3	5	2	4
2	4	4	5	1	4	4	1	1
4	4	4	4	4	4	4	4	4
2	1	5	2	4	4	2	4	2
2	2	4	4	3	4	2	4	2
4	3	3	2	4	4	4	2	2
1	1	5	4	4	4	2	2	1
2	1	5	4	4	4	2	4	2
3	2	1	1	4	3	5	2	5
4	3	3	3	3	3	3	3	3
3	2	4	2	2	4	4	4	2
2	2	4	3	4	4	2	3	2

2	2	4	2	4	3	4	3	4
3	2	4	3	3	4	3	3	3
4	3	3	3	3	3	3	3	2
2	2	4	4	4	4	4	3	1
4	3	5	1	2	3	5	5	5
1	1	5	2	5	4	1	4	1
2	1	5	4	4	4	3	2	2
3	2	2	3	2	3	3	3	2
1	1	5	5	5	5	1	1	1
1	1	5	3	5	5	3	2	1
4	2	4	4	4	4	3	4	4
3	4	5	3	4	5	4	4	4
1	1	5	5	4	5	1	1	1
2	2	2	4	2	3	3	3	2
2	2	4	4	3	4	3	2	2
1	1	5	5	5	5	1	1	1
4	2	4	4	4	4	4	4	3
3	3	3	3	3	4	4	4	3
3	2	5	3	4	4	2	2	4
3	1	4	3	4	4	3	1	3
3	2	5	3	4	4	3	3	3
2	2	4	4	4	4	2	2	2
3	3	3	2	3	3	4	3	2
3	2	4	3	4	4	4	4	3
3	2	4	2	4	3	3	2	3
3	1	3	3	1	1	5	1	5
1	1	4	4	5	5	1	1	1
4	3	4	3	3	3	4	3	3
5	5	5	5	5	5	5	5	5
4	4	4	2	2	2	4	2	4
1	2	3	3	3	3	3	3	3
1	1	5	5	5	5	1	2	1
3	3	4	3	4	3	4	2	3
2	1	4	3	4	4	3	2	2
1	1	5	5	5	5	1	1	1
3	3	4	4	3	4	4	3	3
1	1	4	2	4	5	2	3	1
2	2	4	4	4	4	4	4	4
5	4	3	2	3	4	5	5	4
4	2	4	4	4	4	3	4	2
2	2	4	3	4	4	4	4	2
2	1	5	2	5	5	4	5	2
4	2	4	2	3	2	4	4	4
3	2	4	4	4	4	2	2	2
1	1	5	4	5	5	2	2	1
1	1	4	4	4	4	3	2	1
3	1	1	2	3	3	3	3	3
2	2	4	4	4	4	4	4	2
5	4	3	2	2	2	5	2	3
3	3	4	3	3	3	3	3	3
2	2	4	4	5	4	4	4	2
3	3	2	3	3	3	3	3	3
2	2	4	3	4	4	3	4	3
3	2	4	3	4	4	4	2	3

4	4	4	4	4	4	4	4	4
3	4	3	3	3	2	3	4	1
4	3	2	3	3	4	3	2	2
5	2	2	2	2	3	3	3	3
2	2	3	3	3	4	3	3	3
2	2	4	4	4	4	2	2	2
5	2	4	2	2	2	4	4	3
5	4	4	2	2	2	5	2	4
4	3	3	1	3	1	2	2	3
3	3	3	3	3	3	3	3	3
2	1	5	3	1	4	3	5	2
2	2	5	4	3	4	4	3	1
3	3	3	4	2	2	5	5	3
2	2	5	3	2	4	2	3	2
2	2	4	4	4	4	4	4	2
1	1	4	4	4	4	2	1	2
4	3	3	2	2	2	3	3	3
2	1	4	3	4	4	4	3	2
1	1	4	4	5	3	1	1	1
3	1	5	3	4	4	4	3	2
4	2	4	3	2	3	4	3	4
4	4	4	4	4	4	4	4	4
3	3	4	3	3	3	2	2	3
3	3	3	3	3	3	3	3	3
1	1	5	5	5	5	2	3	1
4	1	5	1	5	5	3	1	1
3	2	5	3	3	4	2	3	2
2	2	4	4	2	3	3	4	2
2	1	4	4	4	5	3	3	2
1	1	2	3	3	4	4	2	2
2	2	5	2	2	2	2	5	2
2	2	2	4	4	4	3	3	3
2	2	2	2	3	4	4	4	3
4	4	4	4	4	4	4	4	2
2	2	4	4	4	4	3	2	2
3	3	3	3	3	3	3	3	3
5	5	5	5	5	5	5	5	5
1	1	4	5	5	5	2	5	1
4	4	4	4	4	4	4	4	3
1	1	5	5	5	5	1	1	1
4	4	4	4	4	4	4	4	4
4	3	4	4	2	2	3	3	4
1	1	1	1	4	4	2	2	2
2	2	4	3	4	4	4	4	3
4	4	4	4	4	4	4	4	4
4	3	3	2	4	4	4	4	4
2	2	4	4	4	4	2	4	2
2	2	4	4	3	4	4	4	2
1	2	4	4	4	4	2	4	2
2	2	4	4	4	4	2	4	2
2	2	2	2	4	4	2	2	2
3	3	3	3	3	3	3	3	3
1	1	5	4	4	4	2	2	2
1	1	5	3	5	5	1	4	1

1	1	1	1	5	5	3	5	1
1	1	5	5	5	5	5	5	1
1	1	4	4	4	4	2	2	2
3	3	3	3	3	3	3	4	2
1	1	5	4	5	5	2	2	1
1	1	5	5	5	5	1	3	1
2	2	5	3	4	4	4	3	2
3	2	5	4	4	5	3	3	1
3	1	5	3	5	3	2	3	3
1	1	5	5	5	5	4	4	1
1	1	4	3	4	4	2	1	2
5	2	2	4	4	4	5	3	4
2	2	4	4	4	4	3	3	2
2	2	4	4	4	4	3	2	2
2	2	2	2	2	4	4	4	4
3	2	3	2	3	2	4	3	2
3	2	4	2	2	4	4	4	2
1	1	4	4	3	4	2	4	1
2	2	4	4	3	4	2	2	2
1	1	3	3	3	5	3	3	1
1	1	5	1	5	5	1	1	1
3	4	3	3	3	3	3	1	4
3	3	3	3	3	3	3	3	3
2	2	4	3	3	4	3	3	2
5	5	5	4	5	5	1	5	1
4	3	3	3	3	3	3	3	3
2	2	4	3	3	3	4	3	3
1	1	5	3	5	5	2	2	1
1	1	4	4	4	4	4	4	2
5	5	3	1	3	3	3	3	5
2	2	4	3	4	4	2	3	2
3	2	5	3	4	4	3	4	2
1	1	5	5	5	5	5	5	2
2	2	1	4	4	4	2	2	2
2	2	5	3	4	4	4	5	1
1	1	5	1	5	5	1	3	1
1	1	5	3	4	3	2	1	2
2	2	4	3	3	3	3	4	3
2	1	3	1	5	5	2	4	1
2	2	4	4	4	4	2	2	2
5	5	5	5	5	5	5	5	5
3	3	3	3	4	3	3	3	3
2	1	5	3	4	4	3	2	1
2	2	4	4	2	4	4	4	2
1	1	5	5	4	4	2	2	1
3	1	4	4	4	4	4	4	2
1	1	5	1	1	1	5	1	5
3	3	3	3	3	3	3	3	3
1	2	5	3	3	4	4	3	2
2	2	5	5	5	5	3	5	2
4	4	4	3	3	3	3	3	3
2	2	4	3	3	3	2	2	2
2	2	4	4	4	4	2	4	2
1	1	5	5	3	5	2	2	1

1	1	5	5	5	5	5	5	5
1	1	5	4	5	5	2	2	1
1	1	3	5	3	3	1	3	1
2	2	4	4	4	4	1	1	1
3	2	3	3	3	4	5	3	3
1	1	5	1	5	5	2	4	3
2	2	4	3	2	4	5	3	2
1	1	5	5	5	5	5	5	1
2	2	5	5	4	5	3	5	5
1	1	5	4	5	4	2	2	1
2	2	4	2	4	4	2	4	2
2	3	3	3	4	5	4	4	5
1	1	5	5	5	5	1	1	1
3	3	3	3	3	3	3	3	3
2	2	4	2	3	4	4	2	2
1	1	4	3	4	4	4	4	1
2	1	4	3	4	4	2	1	2
2	4	4	3	5	3	3	2	2
2	1	4	2	2	2	3	1	3
2	2	4	3	3	4	2	2	1
3	2	5	4	5	3	3	2	2
2	2	4	4	4	4	2	2	2
1	1	5	5	5	5	1	1	1
1	1	3	5	5	1	1	3	1
3	3	4	2	3	3	4	3	4
1	1	5	5	5	5	1	1	1
1	1	5	5	2	4	2	1	1
4	2	5	2	2	3	4	3	4
1	1	5	2	4	4	1	3	1
2	1	3	2	4	3	2	3	2
3	3	3	3	3	3	3	3	3
1	1	4	5	4	4	1	2	1
2	2	4	2	4	4	2	2	2
1	1	5	5	5	5	1	3	1
5	5	4	1	3	4	4	2	4
4	2	4	3	3	2	4	2	4
4	3	5	3	3	3	3	3	4
1	1	5	1	5	5	1	1	1
2	2	4	2	4	2	4	2	2
1	1	5	5	4	3	1	2	2
2	2	4	4	3	4	2	2	2
1	1	5	5	5	5	1	1	1
5	1	5	3	5	5	4	3	3
1	1	5	5	5	5	1	4	1
3	3	3	3	3	3	3	3	3
2	2	4	4	3	4	4	2	2
1	1	4	3	5	5	1	3	1
2	1	5	2	3	5	5	2	1
3	3	3	3	2	3	4	3	4
1	1	5	1	5	5	1	1	1
3	3	3	3	3	3	3	3	3
1	1	5	5	5	4	3	3	1
1	1	5	5	5	5	1	5	1
3	1	5	2	5	4	4	3	2

3	3	3	3	3	3	3	3	3
2	1	4	4	4	4	3	4	2
1	1	4	3	4	5	1	1	1
3	3	3	3	3	3	3	3	3
1	1	5	1	5	5	5	4	4
1	1	5	4	4	4	2	5	2
3	4	3	3	4	4	3	3	3
2	2	3	1	2	2	2	2	2
2	2	5	4	4	4	2	4	2
1	1	5	5	5	5	3	5	1
1	1	5	5	5	5	1	1	1
2	2	4	4	4	4	4	3	3
1	1	5	5	5	5	1	2	1
4	4	4	2	2	4	4	4	4
2	1	3	3	4	4	3	2	1
2	4	4	4	5	5	5	5	5
1	1	5	3	4	3	2	2	2
1	1	5	4	5	5	1	2	1
2	2	4	3	4	4	4	3	2
3	2	3	3	3	3	3	3	4
1	1	3	3	4	4	4	4	2
3	3	4	3	3	3	3	3	3
1	1	5	5	5	5	5	5	5
1	1	5	1	5	5	1	5	1
3	2	3	4	3	2	4	4	3
4	4	2	2	4	3	2	3	2
1	2	1	3	3	2	2	2	2
3	2	4	2	3	3	2	2	3
1	1	5	3	4	4	4	4	1
4	2	5	3	2	3	2	3	3
4	2	4	4	2	4	4	2	2
3	3	3	3	3	3	3	3	3
2	2	4	4	4	4	2	4	2
4	3	3	2	3	3	3	3	4
3	3	3	3	3	3	3	3	3
4	2	4	2	4	5	4	4	4
5	2	4	3	2	3	4	3	4
4	2	2	3	4	4	2	4	2
3	2	4	4	4	4	3	3	3
3	3	3	3	3	3	3	3	3
5	2	5	3	3	5	5	2	5
2	1	2	4	4	4	2	2	2
1	1	5	1	5	5	4	3	1
5	3	3	2	3	3	4	4	4
4	3	4	3	3	3	4	3	4
1	1	5	5	5	5	1	5	1
4	3	3	2	3	3	4	4	4
2	2	4	3	4	4	2	4	2
3	2	4	4	4	4	3	4	2
3	1	4	2	4	4	2	1	1
2	1	5	3	4	4	3	3	1
2	2	4	4	4	4	2	2	2
3	3	3	3	3	3	3	3	3
3	3	3	3	3	4	4	4	3

2	2	5	3	2	4	4	2	2
3	3	4	3	3	4	4	2	3
3	1	4	3	4	3	4	1	2
1	1	4	4	4	4	4	4	1
4	4	4	2	2	2	4	3	4
3	3	3	3	4	3	5	1	1
3	2	4	4	3	4	5	5	3
3	3	3	3	3	3	3	3	3
1	1	5	3	5	5	4	4	1
2	2	5	5	5	5	2	2	2
2	2	4	4	4	4	2	2	2
1	1	1	1	1	1	1	1	1
2	2	3	4	4	3	2	2	2
4	2	4	3	3	4	4	3	4
2	1	4	2	3	5	2	2	2
5	3	4	4	4	3	3	3	3
2	1	5	4	4	4	4	4	2
5	3	5	1	1	2	4	3	3
2	2	4	4	4	3	4	2	2
4	4	4	2	2	3	4	1	4
5	2	5	3	5	5	5	5	5
3	3	3	3	3	3	2	3	3
3	2	4	3	4	4	4	4	3
3	2	3	3	4	4	3	4	3
2	2	4	4	4	4	4	4	4
3	3	5	3	3	4	4	4	4
3	3	3	2	3	3	3	3	3
5	5	5	1	1	1	5	1	5
4	2	4	2	4	2	4	2	4
2	1	5	4	4	4	2	4	2
4	1	4	4	4	3	4	4	2
3	2	4	3	4	4	4	4	3
3	2	3	3	3	3	3	3	3
1	1	5	1	5	5	1	5	1
3	3	4	4	4	4	4	4	4
4	2	4	3	3	4	3	2	2
3	3	4	3	4	4	4	4	3
4	4	4	4	4	4	4	4	4
3	2	4	2	2	4	4	4	2
4	3	4	2	3	2	3	4	4
1	1	5	5	4	5	3	2	1
2	2	4	3	4	4	3	3	2
4	4	4	4	4	4	4	4	4
3	2	4	3	4	3	2	2	3
2	1	3	3	3	4	2	4	2
2	1	4	2	4	4	3	3	4
3	2	5	4	2	4	3	2	2
3	3	3	3	3	3	3	3	3
2	1	2	2	3	5	3	5	1
1	1	4	3	4	4	3	3	2
2	2	4	4	4	4	2	4	2
1	1	5	3	5	5	4	2	5
1	1	4	4	4	4	2	2	1
1	1	5	2	5	5	2	1	1

3	2	4	2	4	3	3	3	2
3	4	4	3	4	3	3	4	3
3	2	4	3	4	4	3	3	2
3	3	4	3	4	4	3	3	3
3	3	3	3	3	3	3	3	3
2	2	3	3	3	3	3	3	4
1	1	5	2	3	1	3	4	2
2	2	5	2	4	5	2	2	2
1	1	3	1	3	4	4	1	1
1	1	3	4	4	5	2	1	1
1	1	5	5	3	5	2	1	1
1	1	4	4	3	5	1	2	1
2	2	4	3	4	3	3	1	1
2	2	4	4	4	4	3	3	3
1	1	5	5	5	5	1	3	1
2	2	2	4	4	4	4	2	2
1	1	5	1	5	5	1	1	1
4	2	4	2	3	4	2	4	2
3	3	4	3	3	3	3	3	3
4	2	4	1	2	3	3	3	5
2	1	5	3	5	5	2	1	1
3	2	4	3	3	3	3	2	3
5	5	5	5	5	5	5	1	1
1	1	4	5	5	5	1	1	1
1	1	5	4	5	5	2	1	1
4	3	5	2	2	2	4	5	4
1	1	4	2	5	5	2	4	1
1	1	5	1	5	5	1	1	1
3	3	4	3	3	4	3	3	2
1	1	5	5	5	5	1	1	1
2	1	1	3	4	3	1	1	1
1	1	4	4	5	5	2	1	1
4	1	4	3	3	3	3	3	3
1	1	5	5	4	5	1	1	1
1	1	5	3	4	3	4	4	1
2	1	5	4	4	4	2	2	2
2	2	4	3	1	3	5	2	2
5	4	3	1	2	1	4	1	5
3	3	3	3	3	3	3	3	3
2	1	4	3	4	4	2	1	2
1	1	5	5	5	5	1	1	1
3	3	4	2	3	3	3	4	2
3	3	3	3	3	3	3	3	3
1	1	4	2	4	4	2	2	1
3	3	3	2	3	3	2	2	3
3	2	3	2	5	4	4	2	3
1	1	3	4	4	3	2	1	1
3	3	4	3	3	3	3	3	3
2	1	4	4	4	4	2	2	2
3	2	4	3	3	3	2	2	2
5	2	1	2	4	4	1	4	1
1	1	5	5	5	5	1	2	1
2	1	3	2	4	4	3	1	1
1	1	1	4	4	5	1	2	1

2	1	3	4	5	4	3	1	1
2	2	4	4	3	5	1	2	2
3	2	4	3	4	4	4	3	1
5	5	1	5	1	1	5	1	5
3	2	3	3	2	3	3	2	3
3	4	3	2	3	2	4	2	4
2	2	3	3	3	4	3	2	3
3	2	4	4	4	4	4	2	2
2	2	4	2	3	3	3	2	2
4	1	3	3	2	2	4	3	2
2	1	5	5	4	4	2	4	1
2	2	4	2	3	2	2	2	3
3	2	4	3	4	4	3	3	2
4	1	5	5	5	5	1	4	1
1	1	5	5	4	5	2	4	1
3	3	3	3	3	3	3	3	3
2	2	4	2	4	4	3	4	2
2	1	4	4	4	4	2	3	1
2	2	3	3	4	4	2	4	2
2	2	2	3	4	4	3	2	2
4	3	4	3	3	4	2	3	2
4	3	4	2	2	2	4	3	4
5	5	5	5	5	5	5	5	5
5	2	4	2	4	4	4	4	3
3	2	5	2	3	4	4	3	3
3	2	4	2	3	3	3	3	3
3	3	3	3	3	3	3	3	3
1	1	5	4	4	4	1	4	1
3	3	3	3	3	3	3	3	3
2	2	4	3	4	4	3	4	3
3	3	3	3	3	3	3	3	3
4	3	4	2	3	3	4	3	4
3	3	4	2	4	4	4	3	3
2	2	4	3	4	3	3	4	2
3	3	4	3	3	3	4	4	3
2	2	4	4	4	4	2	4	2
2	2	5	4	5	5	3	4	1
2	2	4	4	4	4	2	3	2
2	2	3	3	3	3	4	4	4
3	3	3	3	3	3	3	3	3
1	1	4	4	4	4	2	2	2
1	1	5	5	5	4	3	4	1
2	2	4	2	3	4	3	3	2
2	2	4	4	4	4	2	2	2
4	4	3	2	2	2	3	3	4
5	2	4	5	4	4	4	2	5
1	1	4	4	4	4	1	3	1
1	1	5	4	4	4	2	2	2
1	1	4	4	4	4	3	4	2
1	1	4	3	4	4	1	1	1
1	1	5	3	4	5	2	3	1
1	1	3	4	4	4	4	3	2
1	1	5	5	5	5	4	1	1
2	2	5	3	4	4	4	3	2

2	3	4	4	3	4	3	2	2
2	2	4	4	4	4	2	4	2
2	1	4	3	3	4	4	3	2
1	1	5	5	5	5	2	2	1
1	1	4	4	4	4	2	2	1
2	2	5	3	4	4	4	3	2

C42	C43	C44	C45	C46	C47	C48	C49	C50
5	5	5	5	5	5	5	5	5
4	4	2	2	4	4	3	1	4
5	5	5	5	5	5	5	5	5
5	5	5	5	5	5	5	5	5
3	3	3	3	3	4	3	3	3
5	5	5	5	5	5	5	5	4
5	5	5	5	5	5	5	5	5
4	5	4	2	4	4	2	3	2
2	2	2	2	2	2	3	2	2
5	5	5	5	5	3	3	5	5
5	5	5	5	5	5	5	2	5
4	4	4	3	4	5	3	3	4
5	5	5	5	5	1	1	1	5
5	5	5	5	5	5	5	5	5
5	5	3	5	3	5	3	5	3
5	5	5	5	5	5	5	5	5
4	5	4	3	4	3	4	2	4
5	5	5	5	5	5	5	5	5
4	4	4	4	4	3	3	3	4
4	5	4	2	4	4	2	3	5
5	5	5	5	5	5	5	5	5
5	5	3	5	2	5	2	5	5
5	5	5	5	5	2	4	1	5
5	5	5	5	5	1	1	1	5
2	4	2	2	2	4	4	4	4
4	4	3	3	4	3	2	2	4
3	3	3	3	3	3	3	3	3
3	4	4	2	3	3	4	2	4
5	5	4	3	4	4	2	2	5
4	5	2	2	4	4	2	3	5
5	5	5	5	5	3	5	1	5
5	5	5	5	5	1	1	1	5
5	5	5	3	5	4	2	1	5
5	5	5	5	5	4	4	4	3
5	5	5	3	4	5	2	1	5
4	4	4	4	4	4	4	5	4
5	5	5	5	5	5	5	1	5
5	5	5	5	5	5	5	5	5
5	5	5	5	5	2	2	2	4
5	5	5	5	5	2	1	2	5
4	4	4	4	4	1	2	1	4
4	4	4	4	4	2	4	2	4
5	5	5	5	5	2	2	3	5
5	5	5	4	4	2	1	1	5
4	4	4	4	4	2	4	3	4
4	4	4	4	4	4	4	4	4
5	4	4	4	4	4	3	1	4
5	5	5	5	5	5	5	5	5
3	2	3	3	2	2	2	2	3
4	1	5	2	4	4	1	1	5
5	5	5	5	5	5	5	5	5
5	5	5	4	5	3	5	5	5
4	4	4	4	3	3	4	3	4

3	3	3	3	3	3	3	3	3
4	4	4	3	4	3	4	2	4
3	3	3	1	3	3	5	5	5
5	5	5	5	5	5	5	5	5
5	5	3	3	5	5	3	5	5
5	5	4	4	4	1	5	1	5
5	5	5	5	5	1	1	1	5
5	5	5	5	5	5	5	5	5
4	5	4	4	4	4	2	3	4
4	5	3	3	3	5	4	2	5
4	4	4	4	4	2	3	2	4
4	5	3	4	4	2	4	3	5
5	5	5	5	5	3	3	1	5
3	3	3	2	1	2	2	4	4
4	4	4	4	4	2	4	2	4
5	5	3	3	5	3	3	1	5
5	5	5	5	5	1	5	1	5
5	5	5	5	4	3	4	3	3
4	5	4	4	4	4	4	1	4
3	5	3	3	4	3	3	2	5
4	5	4	4	4	3	4	2	4
4	5	4	4	4	3	3	1	5
3	3	5	1	5	5	3	3	3
3	3	3	3	3	3	3	3	3
3	3	3	3	3	3	3	3	3
4	2	2	1	1	5	1	5	3
4	5	1	4	4	2	3	2	5
3	3	3	3	3	3	3	3	3
3	4	2	3	3	4	3	3	3
3	3	3	2	2	5	2	4	3
3	3	2	2	1	4	3	4	3
4	4	3	3	3	4	3	3	4
5	5	5	5	5	2	1	1	5
3	3	3	3	3	3	3	5	3
4	5	3	3	4	3	2	1	5
5	5	5	5	5	1	1	2	5
5	5	5	5	5	3	1	3	5
4	4	2	2	3	4	2	3	4
5	4	4	3	4	5	2	1	3
5	4	5	5	5	1	3	1	5
3	4	3	3	3	4	2	3	3
4	4	4	4	4	2	2	2	4
2	5	3	3	3	3	5	3	3
5	5	5	5	5	1	3	1	5
4	5	4	3	3	4	3	2	4
5	2	3	3	2	2	3	4	3
5	4	5	4	4	3	3	2	4
3	5	3	3	3	3	3	3	5
5	5	5	5	5	2	1	1	5
4	5	3	3	2	5	2	4	4
2	5	2	2	2	4	4	4	4
5	5	5	5	5	1	1	1	5
2	2	2	2	2	2	2	2	2
2	4	3	2	3	4	4	4	3

3	3	3	3	3	2	4	4	4
2	4	1	3	1	5	1	5	4
5	5	4	4	4	5	5	5	5
5	5	5	5	5	1	1	1	5
5	5	5	5	5	2	1	1	5
5	5	2	2	5	5	4	5	5
4	5	3	3	4	5	4	3	4
4	5	2	4	4	2	2	2	5
5	5	5	5	5	2	1	1	5
3	3	3	3	3	3	3	3	3
3	5	3	3	3	3	3	3	4
4	5	5	4	4	2	3	1	5
5	5	3	3	3	3	1	1	5
4	5	4	4	5	2	4	1	5
4	5	4	4	4	2	2	2	4
4	4	4	4	4	4	1	3	4
2	5	4	5	5	4	3	3	5
4	5	5	4	5	2	2	2	5
4	4	4	3	4	2	1	2	4
4	5	4	3	3	2	1	2	5
4	4	4	4	4	4	2	2	4
5	5	5	5	5	1	5	1	5
3	3	3	3	3	3	3	3	3
3	3	3	3	3	3	3	3	3
2	4	2	2	2	4	2	4	3
2	4	2	2	2	3	2	4	4
3	3	2	3	2	2	2	1	4
3	4	2	1	3	5	4	5	3
3	5	2	3	4	4	1	3	4
5	5	5	5	5	1	1	1	5
4	5	4	4	4	2	4	2	4
4	5	3	3	4	2	2	2	5
4	3	3	5	3	4	3	4	3
1	5	1	1	1	5	1	5	1
4	5	5	2	4	1	1	2	5
5	5	4	4	4	3	4	1	5
4	3	5	5	5	1	3	3	4
4	5	5	5	5	5	5	2	4
4	5	4	3	4	3	4	1	5
4	4	5	4	3	4	4	5	5
5	5	4	3	4	3	1	1	5
2	5	3	1	1	5	4	5	4
4	3	3	3	3	3	3	3	3
1	4	3	2	2	4	2	5	4
5	3	3	5	5	3	1	1	5
5	5	3	4	5	4	1	1	5
2	4	2	2	2	4	1	4	4
5	4	5	5	5	2	4	1	5
4	5	4	5	4	2	2	2	5
2	4	3	2	3	4	4	3	4
5	5	4	3	4	3	1	1	5
5	5	5	5	5	5	5	5	5
4	4	2	2	4	3	3	4	5
4	4	4	4	3	2	3	2	4

4	5	4	3	4	2	2	1	5
4	5	4	3	3	3	3	2	5
5	5	5	5	5	1	3	1	5
3	4	2	2	3	4	3	4	4
4	5	4	3	5	4	4	2	5
4	4	4	4	4	3	3	3	4
4	4	4	4	4	3	3	2	5
2	4	4	4	4	2	2	2	4
2	3	2	2	2	4	3	4	2
5	5	4	5	2	4	1	1	5
4	5	2	5	5	3	4	1	4
5	5	5	4	5	1	1	1	5
3	3	3	3	3	3	3	3	3
5	5	5	5	5	1	1	1	5
3	3	3	3	3	3	3	3	3
3	4	3	4	4	4	4	4	4
4	5	4	4	4	4	2	1	5
4	4	4	3	4	3	2	2	4
3	4	5	1	3	3	2	4	3
5	5	5	5	5	1	5	1	5
3	5	3	2	3	4	2	2	5
5	5	4	5	5	2	2	1	5
4	5	4	4	4	3	1	3	5
5	5	5	3	5	2	4	1	5
3	3	3	3	3	3	3	3	3
3	4	4	4	3	5	4	2	5
4	4	3	3	4	3	2	3	4
3	4	3	3	3	3	2	1	4
1	5	1	1	1	3	1	4	4
5	4	5	5	5	2	1	1	5
4	4	3	3	4	4	3	3	4
5	5	5	5	5	1	1	1	5
5	5	5	4	5	3	1	1	5
5	5	3	5	4	3	1	1	5
3	3	3	3	3	3	3	3	3
5	5	5	5	5	2	2	2	5
3	4	3	2	3	2	3	3	4
4	4	5	5	5	2	4	1	5
4	5	4	4	4	3	3	4	5
5	4	4	5	5	2	2	1	5
4	5	5	3	4	3	3	3	4
4	4	4	4	4	2	2	4	4
5	5	5	1	5	5	1	5	5
4	5	4	4	4	2	4	4	4
3	4	3	3	3	3	3	3	3
4	4	3	3	4	3	4	2	4
3	3	3	3	3	3	3	3	3
5	5	5	5	5	1	5	1	5
5	5	5	5	5	1	4	1	5
5	5	5	5	5	2	3	1	5
4	4	5	2	4	1	2	1	5
4	3	3	4	4	3	1	1	4
3	3	3	3	3	3	3	3	3
5	5	5	5	4	5	3	4	5

3	5	5	5	4	3	3	3	3
1	3	4	2	2	4	4	2	1
4	5	5	4	5	1	4	3	4
3	4	3	3	3	3	2	3	4
5	4	3	3	3	4	4	2	5
5	5	5	5	5	2	5	2	5
3	2	4	5	5	5	5	5	4
4	4	4	3	5	3	1	1	5
4	4	2	4	4	4	4	2	5
4	4	3	3	4	3	3	2	4
5	5	5	5	5	5	5	4	5
5	5	5	5	5	5	5	3	5
1	1	1	1	1	1	1	1	1
3	5	3	3	5	3	1	3	5
3	3	3	3	3	3	3	3	3
5	4	5	5	4	5	5	2	5
3	3	3	3	3	3	3	3	3
3	4	4	4	5	3	5	2	4
3	5	2	3	3	4	3	3	3
3	5	3	3	4	4	4	3	5
4	4	4	4	4	3	4	2	4
4	4	4	4	4	3	4	2	4
5	5	5	5	5	2	5	1	5
5	5	5	5	5	1	5	1	5
4	3	3	3	3	3	3	3	3
3	5	3	3	3	3	3	3	3
3	4	3	3	3	4	2	3	4
5	5	5	5	5	3	5	1	5
5	5	5	5	5	5	5	5	5
5	5	5	2	5	4	2	1	5
3	3	3	3	3	3	3	4	2
1	3	1	1	1	5	2	5	4
3	4	2	2	2	4	2	4	4
5	5	5	5	5	5	5	5	5
3	5	4	3	3	2	2	2	5
4	5	3	3	4	4	3	2	5
4	5	4	3	4	4	3	2	5
5	5	5	1	5	4	3	3	4
4	4	4	4	4	4	4	4	4
5	4	5	5	5	5	3	2	5
4	4	4	4	4	2	2	2	4
3	5	3	3	3	3	4	3	5
3	5	3	3	3	5	4	3	4
5	5	5	5	5	1	1	1	5
4	5	3	3	4	1	5	1	5
3	4	3	3	4	3	3	3	4
4	5	3	3	3	4	4	4	4
2	4	4	4	4	3	4	2	3
5	4	4	4	5	2	1	2	5
1	1	4	2	4	3	4	2	2
3	4	4	4	3	4	3	3	4
5	5	5	5	5	1	1	1	5
3	4	3	3	3	3	3	3	3
4	4	4	4	4	4	4	2	4

5	5	5	5	5	2	5	1	5
5	5	4	3	4	2	1	1	5
2	5	2	1	3	5	1	1	5
3	4	3	3	3	3	3	3	3
3	3	3	3	3	3	3	3	3
5	5	5	3	5	5	5	1	5
5	5	3	3	2	1	1	1	5
4	5	4	4	4	3	4	4	5
3	5	3	2	4	5	1	3	5
1	5	1	1	1	5	5	5	5
4	4	4	4	4	3	3	2	4
4	5	4	4	2	1	2	2	5
4	4	4	4	4	4	4	4	4
4	4	4	2	3	3	4	2	4
5	5	2	4	5	4	2	1	5
5	5	5	5	5	2	2	2	5
3	3	3	3	3	3	3	3	3
4	5	4	4	4	4	2	2	5
5	5	5	5	5	2	2	1	5
3	4	3	2	3	4	2	4	2
4	4	4	4	4	3	2	1	4
3	5	3	4	4	4	4	2	4
4	5	4	3	4	5	4	3	3
4	4	2	2	4	4	3	2	4
3	4	1	4	4	4	3	4	5
3	5	1	1	1	5	1	5	5
5	5	5	5	5	3	2	1	5
4	4	3	4	4	3	2	2	4
5	3	2	5	1	4	2	5	1
5	5	5	5	5	2	4	2	5
5	3	5	5	5	2	1	1	5
3	5	4	3	4	4	4	2	5
1	1	1	1	1	1	1	1	1
5	5	5	5	5	5	5	5	5
3	3	3	3	3	3	3	3	3
2	4	4	3	3	4	4	3	4
1	1	2	4	4	5	5	1	1
5	4	4	4	4	3	4	2	4
3	5	1	3	4	5	2	4	4
3	3	3	3	4	4	3	4	4
4	4	4	4	4	2	2	2	4
3	4	3	3	3	4	3	3	3
2	5	4	3	3	3	2	2	4
4	4	4	4	4	4	4	3	4
3	4	4	3	4	4	4	3	4
2	4	2	2	2	3	2	4	4
4	4	4	4	4	4	4	2	4
4	4	4	4	4	2	2	2	4
4	4	3	3	4	2	3	2	4
4	4	4	4	4	4	2	4	5
4	4	4	2	4	4	2	2	4
5	5	5	5	5	2	1	1	5
4	5	5	5	5	4	3	2	4
2	5	2	2	2	4	2	4	4

5	5	4	4	4	4	4	1	5
4	4	4	4	4	3	2	2	5
3	3	3	3	3	3	3	3	3
4	4	5	5	5	5	5	2	4
1	4	4	4	4	4	5	2	4
2	4	2	2	4	4	4	4	4
4	5	4	4	4	2	2	1	5
4	4	4	4	2	4	2	4	4
4	4	4	4	4	2	4	2	4
3	3	3	3	3	3	3	3	4
3	5	4	3	3	4	2	2	4
4	4	4	4	4	2	3	2	4
4	4	4	4	4	4	4	1	4
4	4	4	4	4	3	3	2	4
4	4	4	3	4	4	4	2	4
4	4	4	4	4	4	4	4	4
4	4	4	4	4	4	4	2	4
4	4	4	4	4	2	2	2	4
3	4	3	3	4	4	2	2	4
3	3	3	3	3	3	3	3	3
4	4	4	3	4	3	1	1	4
4	3	3	3	3	3	3	3	3
5	5	5	5	5	3	2	3	5
3	5	3	3	3	4	3	2	5
4	4	4	4	4	2	4	2	5
5	5	5	5	5	1	5	1	5
4	4	4	4	4	2	3	1	4
3	4	2	2	2	4	2	4	4
4	5	3	2	4	4	4	4	5
2	5	4	4	3	4	2	3	4
4	5	4	3	4	3	3	2	4
2	4	4	3	4	4	2	3	4
4	4	4	4	4	4	3	3	4
2	5	3	4	4	3	3	3	4
4	5	4	4	4	2	4	2	4
3	5	2	2	2	4	2	3	4
5	5	2	2	2	4	2	4	5
3	3	3	3	3	3	3	2	4
4	5	4	3	3	2	4	2	4
5	5	5	5	5	5	5	5	5
2	5	3	2	3	4	2	4	5
3	5	4	4	4	3	3	4	4
3	4	3	2	3	4	3	3	4
2	5	2	2	3	4	3	4	4
5	5	3	2	3	5	4	3	5
4	4	4	5	5	2	3	2	4
2	5	3	2	4	4	3	2	5
5	5	5	5	5	2	3	3	5
5	5	4	5	5	2	2	1	5
4	4	4	4	4	3	3	2	4
5	5	5	5	5	2	2	1	5
5	4	4	4	5	4	4	2	4
4	4	4	4	4	4	4	4	4
3	3	3	3	3	3	3	3	3

4	4	4	4	4	3	2	2	4
5	5	5	5	5	3	4	1	5
4	5	5	5	5	4	4	3	5
3	3	3	3	3	3	2	3	4
2	4	2	2	3	4	4	4	4
4	5	5	5	5	1	5	1	5
1	4	4	3	4	3	4	1	4
4	5	2	2	3	1	1	1	5
4	4	4	3	4	4	4	2	4
2	5	3	2	4	1	1	4	4
3	5	4	4	5	2	2	2	4
4	5	3	3	3	4	2	2	5
4	4	4	3	4	2	3	3	4
4	5	3	3	2	5	2	3	5
3	5	3	3	3	3	3	1	5
3	3	3	3	3	2	3	2	5
2	4	2	2	3	4	3	2	4
3	3	3	3	3	3	3	3	3
5	5	5	5	5	5	5	5	5
2	5	4	3	3	3	3	3	4
3	4	4	4	5	3	3	3	4
5	5	5	5	5	2	2	2	5
5	4	4	4	4	4	3	2	4
5	5	5	5	5	2	2	2	5
1	1	1	1	2	5	1	1	1
2	4	3	3	4	4	2	2	4
5	5	4	4	4	2	3	2	5
3	5	3	1	1	5	1	1	5
3	4	3	3	3	4	4	3	4
3	3	3	3	3	3	3	3	3
5	5	4	3	5	2	1	1	5
3	3	3	3	3	3	3	3	3
5	5	5	5	5	2	4	2	5
1	4	1	2	1	5	2	2	2
2	4	2	2	2	4	2	4	4
3	4	2	2	2	4	2	2	3
2	2	1	1	1	1	1	1	5
2	4	4	2	3	4	3	2	4
4	4	4	4	4	4	4	2	4
2	5	2	2	2	4	4	4	5
5	5	5	3	5	3	4	3	5
3	3	3	3	3	3	3	3	3
2	5	3	3	3	4	4	2	4
4	5	3	4	4	5	2	4	4
4	4	4	4	4	4	4	4	4
4	4	4	2	4	4	2	2	4
2	5	2	4	4	2	2	2	4
2	3	1	5	3	1	1	1	1
4	4	4	4	4	1	1	1	4
4	5	5	5	5	3	4	2	4
5	5	5	5	5	5	4	5	4
3	3	3	3	3	3	3	3	3
3	4	2	2	2	4	4	3	4
4	5	4	3	4	3	2	3	4

4	5	2	2	4	4	2	2	4
3	5	3	2	4	3	3	2	4
2	2	3	3	3	3	3	3	3
4	4	4	4	4	2	2	3	4
3	5	2	1	2	5	1	5	3
5	5	3	3	4	4	1	1	5
4	4	4	5	4	3	3	2	5
3	5	3	4	3	4	4	2	4
5	5	5	5	5	3	4	1	5
5	5	5	3	5	5	3	1	5
4	4	4	4	4	2	3	4	4
3	4	5	5	5	5	4	5	5
5	5	5	5	5	1	1	1	5
4	4	3	3	4	4	3	3	4
4	4	4	2	4	2	3	2	4
5	5	5	5	5	1	5	1	5
3	3	3	3	3	3	3	3	3
3	4	4	3	4	4	4	4	4
4	5	3	3	4	3	3	3	5
5	5	5	4	4	4	2	4	4
4	5	4	3	4	3	3	3	5
4	4	4	4	4	4	2	2	4
3	3	3	2	3	3	2	3	4
3	5	4	3	3	4	3	4	4
4	4	3	2	3	4	2	3	4
1	5	1	1	1	5	1	5	1
5	5	5	5	5	1	1	1	5
3	5	2	2	2	4	2	4	4
5	5	4	5	5	5	5	1	5
2	4	2	2	2	4	2	4	4
3	3	3	3	3	3	3	3	3
5	5	4	5	5	1	5	1	5
4	4	3	3	3	4	2	3	4
4	4	4	3	4	3	3	2	4
5	5	5	5	5	1	1	1	5
3	4	3	3	3	4	3	3	4
4	5	5	4	5	2	4	1	5
4	4	4	4	4	4	4	4	4
3	4	2	2	2	4	4	5	5
4	4	4	4	4	3	3	3	4
4	4	4	3	3	4	4	2	4
4	5	3	3	4	5	5	2	5
2	5	2	2	2	4	2	3	4
4	4	1	2	4	2	4	2	4
5	5	5	5	5	2	3	1	5
4	1	4	4	5	3	3	1	5
1	3	3	3	2	3	2	3	3
4	4	4	4	4	2	2	2	4
2	4	2	2	2	4	2	5	3
3	3	3	3	4	3	3	2	4
4	4	4	4	4	2	2	2	4
3	3	3	3	3	3	3	3	3
3	4	3	3	4	3	4	3	4
4	4	3	2	4	4	2	4	4

4	4	4	4	4	4	4	5	4
1	4	4	4	3	3	4	3	4
4	3	4	4	4	4	3	3	3
4	3	3	4	2	4	2	4	2
3	3	3	4	4	4	3	2	4
4	4	4	4	4	2	2	2	4
1	3	1	1	3	1	1	3	3
2	4	2	2	2	4	2	4	4
2	4	1	2	2	2	3	1	5
3	3	3	3	3	3	3	3	3
4	5	5	3	4	4	3	3	5
4	5	3	3	4	4	4	3	5
1	1	2	1	4	1	4	5	1
4	5	3	3	4	4	2	3	5
2	5	4	2	4	4	4	2	4
4	5	3	4	4	3	2	2	5
2	4	2	2	3	3	2	4	4
3	3	3	4	3	4	3	2	4
5	5	5	4	4	3	1	1	5
3	5	4	3	3	4	2	2	5
2	3	2	2	2	4	2	4	3
4	4	4	4	4	4	4	4	4
3	3	2	3	4	4	4	4	3
3	3	3	3	3	3	3	3	3
5	5	5	5	5	2	4	1	5
3	5	1	1	1	5	3	5	5
3	5	3	3	3	2	3	3	4
4	4	4	4	4	3	2	2	4
4	5	4	3	5	3	4	1	5
3	4	3	3	3	4	1	1	4
2	5	3	2	2	2	1	1	1
3	5	5	4	4	3	4	2	4
4	4	4	4	4	4	3	2	4
4	4	4	4	4	4	4	2	4
4	4	4	4	4	3	3	2	4
3	3	3	3	3	3	3	3	3
5	5	5	5	5	5	5	5	5
5	5	5	5	5	5	1	1	5
4	3	4	4	4	3	4	4	4
5	5	5	5	5	2	1	1	5
4	4	4	4	4	4	3	3	4
5	5	4	3	3	3	3	2	3
4	5	5	5	5	2	2	2	5
4	4	4	4	4	4	2	2	4
4	4	4	4	4	4	4	4	4
3	4	2	2	2	4	2	4	2
4	4	4	4	5	2	2	2	4
4	5	4	4	4	4	4	2	4
5	5	5	4	4	2	4	1	4
4	4	4	4	4	2	4	2	5
4	4	3	4	4	2	3	2	4
3	4	3	3	3	4	3	3	4
5	5	4	4	4	3	2	1	5
4	4	4	4	4	2	2	1	5

5	5	5	5	5	2	2	1	5
5	5	5	5	5	5	1	1	5
4	5	4	3	4	2	2	1	4
4	4	4	4	4	4	4	3	3
5	5	5	4	5	5	1	1	5
5	5	5	5	5	2	1	1	5
5	5	3	3	5	5	1	2	5
5	5	5	5	5	3	1	2	5
4	5	3	4	5	3	3	3	3
5	5	5	5	5	3	3	1	5
4	4	4	3	4	2	2	1	4
3	4	3	4	5	2	4	4	4
3	4	4	3	4	3	4	2	4
4	4	4	4	4	3	2	2	4
4	4	4	4	4	4	4	4	4
3	4	2	3	2	3	2	2	4
4	4	4	2	4	4	4	2	5
4	4	4	4	4	1	1	1	4
4	4	4	3	4	2	2	2	4
5	5	5	5	5	2	1	1	5
5	5	5	5	5	1	4	1	5
3	3	3	4	3	3	3	3	1
3	3	3	3	4	3	3	3	4
4	4	4	4	4	4	3	3	5
5	5	5	3	5	5	5	5	5
3	3	3	3	3	3	3	3	3
3	4	3	3	4	3	3	3	3
4	5	4	4	4	3	2	2	4
4	4	4	4	4	2	2	2	4
1	4	1	2	1	5	3	4	5
4	4	4	4	4	2	4	2	4
4	4	3	3	4	4	4	3	5
5	5	5	5	5	5	5	1	5
5	5	5	5	5	4	2	3	4
5	5	4	4	5	2	4	2	5
5	5	5	5	5	2	2	1	5
4	4	4	4	4	1	3	1	5
3	4	4	4	4	4	4	2	4
5	3	5	4	5	5	5	1	5
4	4	4	4	4	2	2	2	4
5	5	5	5	5	5	5	5	5
3	3	3	2	4	3	3	3	3
5	5	5	3	4	2	2	1	5
4	4	4	4	4	2	3	3	3
4	5	4	4	4	2	4	2	5
1	5	3	3	3	3	1	5	5
5	5	3	1	1	5	1	5	5
3	5	5	5	5	3	3	3	3
4	5	4	3	3	3	3	3	4
3	3	4	4	4	4	4	3	5
3	3	3	3	3	3	3	5	3
3	4	3	3	4	2	4	2	4
4	4	4	4	4	2	4	2	4
5	5	5	5	5	5	2	2	5

5	5	5	5	5	5	5	1	5
5	5	5	5	5	3	2	2	5
5	5	5	5	5	3	3	2	5
4	4	4	4	4	2	3	2	4
3	5	3	3	3	5	3	3	5
5	5	5	5	5	4	5	2	5
4	5	4	4	4	4	3	3	5
1	5	5	5	5	2	5	2	5
5	5	5	5	5	5	5	5	5
4	5	4	5	4	2	2	2	5
4	4	4	4	4	2	2	2	4
3	3	2	2	2	4	4	4	5
5	5	5	5	5	4	1	1	5
3	3	3	3	3	3	3	3	3
4	5	3	3	4	5	4	2	4
5	4	4	4	4	2	2	2	3
5	5	4	3	3	2	2	2	5
3	4	3	4	4	4	3	3	3
3	2	2	3	2	2	3	2	2
4	4	3	2	3	1	2	2	4
3	4	3	3	4	2	2	2	4
4	2	4	4	4	2	2	2	4
5	5	3	5	5	1	1	1	5
5	5	5	5	3	1	5	1	5
3	3	2	1	3	3	3	3	3
4	5	5	5	5	2	5	2	5
5	5	4	5	5	1	1	1	5
2	5	3	2	3	4	5	5	5
4	5	4	3	5	3	4	1	4
2	4	4	3	4	2	2	2	4
3	3	3	3	3	3	3	3	3
5	5	5	5	5	1	1	1	5
4	4	4	4	4	4	2	2	4
5	5	5	5	5	3	3	1	5
2	5	2	2	3	4	4	5	5
3	4	2	1	3	4	4	4	4
2	4	3	1	3	5	2	2	2
5	5	5	5	5	1	5	5	5
4	2	2	4	2	4	2	2	4
4	4	5	5	5	1	1	1	5
4	4	4	4	4	2	2	2	4
5	5	5	5	5	1	1	1	5
5	5	5	5	5	5	5	5	5
5	5	5	5	5	1	4	3	5
3	3	3	3	3	3	3	3	3
4	4	4	4	3	2	3	2	4
5	4	4	4	4	3	1	2	5
5	5	3	2	3	5	4	3	5
2	4	3	2	2	4	3	4	4
5	5	5	5	5	1	4	1	5
3	3	3	3	3	3	3	3	3
5	5	4	3	5	2	3	1	5
5	5	5	5	5	1	1	1	5
4	5	4	4	4	4	2	3	5

3	3	3	3	3	3	3	3	3
5	5	3	3	5	3	3	2	4
5	4	4	5	5	1	2	1	4
3	3	3	3	3	3	3	3	3
5	5	2	3	3	3	2	1	5
5	5	4	4	4	1	1	1	5
3	3	3	3	1	3	3	3	3
4	4	2	2	2	4	2	3	4
4	4	4	4	4	2	4	2	4
5	5	5	5	5	5	5	1	5
5	5	5	5	5	1	4	1	5
4	4	3	3	3	3	4	3	4
5	5	5	5	5	1	4	1	5
4	5	5	5	4	4	4	4	4
4	5	4	3	4	3	3	2	5
5	5	5	5	5	3	5	1	5
4	5	4	3	4	3	3	2	5
5	5	5	3	5	2	2	1	5
4	4	4	4	4	3	5	2	4
3	3	3	3	3	3	3	3	3
4	5	5	5	5	4	3	3	4
3	3	3	3	3	3	3	3	3
5	5	5	5	5	4	4	3	3
5	5	5	5	5	1	5	1	5
3	2	5	4	4	3	3	3	3
2	4	3	3	3	4	4	3	4
3	1	1	1	5	2	1	2	1
3	5	3	2	3	4	3	3	4
4	5	4	4	3	2	3	1	4
3	4	3	3	4	4	5	2	4
2	4	4	4	4	2	2	2	4
3	3	3	3	3	3	3	3	3
4	4	4	4	5	5	4	2	4
3	3	3	3	3	3	3	4	3
3	3	3	3	3	3	3	3	3
4	4	4	4	4	4	4	2	4
2	4	2	2	2	3	3	3	4
4	4	3	3	4	3	3	2	5
3	3	3	3	3	3	3	3	3
3	3	3	3	3	3	3	3	3
2	5	1	2	2	5	2	5	3
4	5	4	4	4	3	2	2	4
4	5	5	5	5	3	4	2	5
3	4	2	2	3	4	3	4	3
3	4	2	2	3	4	3	3	4
5	5	5	5	5	1	1	1	5
2	4	2	2	3	4	4	4	3
4	4	4	4	4	2	4	2	4
4	4	4	4	4	3	4	4	4
4	5	3	3	4	4	2	3	5
4	5	4	3	4	4	3	2	5
4	4	4	4	4	2	2	2	4
3	3	3	3	3	3	3	3	3
3	5	3	3	4	3	4	3	4

4	5	4	3	4	4	2	2	4
2	4	4	3	4	4	2	3	4
2	5	3	2	2	4	3	4	4
4	4	4	4	4	4	4	2	4
4	4	2	2	2	4	2	2	4
1	3	1	1	3	3	3	1	5
2	4	3	3	4	5	5	4	4
3	3	3	3	3	3	3	3	3
5	5	5	4	5	1	4	1	5
5	5	5	2	5	5	5	2	5
4	4	4	4	4	2	2	2	4
1	1	1	1	1	1	1	1	1
4	3	4	3	3	2	2	2	5
3	4	2	2	3	4	3	3	3
5	5	2	4	5	4	2	1	5
2	2	4	5	5	4	4	4	3
4	4	4	4	4	4	3	3	4
3	4	1	3	2	4	4	2	3
4	4	4	3	4	2	4	2	4
3	1	2	2	2	4	1	4	2
5	5	5	5	4	4	4	4	5
3	3	3	3	3	3	3	3	3
4	4	4	3	4	3	3	4	3
3	3	3	3	3	4	3	2	4
4	4	4	4	4	4	3	4	4
4	4	3	3	3	4	3	4	5
3	3	3	3	3	3	3	4	3
1	4	1	1	1	5	1	5	5
2	4	2	4	2	4	2	4	4
4	4	4	4	4	2	2	2	4
3	4	4	4	3	2	5	1	4
4	4	3	4	3	3	3	3	4
3	3	3	3	3	3	3	3	3
1	5	5	5	5	5	5	5	5
4	2	2	2	4	4	4	4	4
4	4	3	4	4	3	2	1	4
3	4	4	4	4	4	4	3	4
4	5	2	4	4	4	4	4	4
2	4	2	2	2	3	2	2	4
2	4	2	2	3	4	3	4	4
5	5	5	5	5	3	3	1	5
4	4	4	4	4	3	3	3	4
4	4	4	4	4	4	4	4	4
3	4	3	2	4	4	3	2	4
4	4	4	4	4	3	4	3	4
3	4	3	2	2	4	2	4	4
4	5	4	3	4	3	4	4	4
3	3	3	3	3	3	3	2	4
2	3	4	2	4	2	3	5	4
3	4	4	2	4	4	4	4	4
4	4	4	4	4	3	4	2	4
5	5	4	4	4	3	2	1	5
1	4	4	3	4	3	3	1	5
5	5	5	2	5	2	1	2	5

3	3	2	2	3	4	2	3	4
2	4	5	4	4	4	2	3	4
4	4	4	3	4	3	3	3	4
4	4	3	3	3	3	3	3	4
4	3	3	4	4	4	3	3	5
4	4	4	3	3	4	3	3	4
5	4	3	2	2	3	4	1	4
4	4	4	4	4	2	2	2	5
5	5	5	5	5	2	4	1	5
4	5	4	4	4	2	1	1	4
5	5	5	5	5	1	1	1	5
5	5	5	4	5	2	2	1	5
4	5	3	3	4	4	1	3	4
3	3	3	3	3	3	3	3	3
5	5	5	5	5	1	3	1	5
4	4	4	4	4	2	2	2	4
5	5	5	5	5	1	4	1	5
3	4	3	2	3	3	4	3	4
3	5	3	3	3	4	3	3	4
2	5	1	2	2	5	3	5	3
4	5	3	3	5	2	3	2	5
3	5	3	3	3	3	2	2	5
5	5	5	5	5	1	1	1	5
5	5	5	5	5	1	1	1	5
5	5	5	5	5	2	1	1	5
2	5	3	2	2	4	2	5	4
5	5	5	5	5	2	2	1	4
5	5	5	5	5	1	1	1	5
1	4	3	2	4	4	4	3	4
5	5	5	5	5	1	1	1	5
4	5	3	3	2	4	2	3	5
5	4	5	4	5	2	1	3	4
3	3	3	3	3	3	3	3	3
5	5	5	5	5	1	1	1	5
4	5	4	3	3	4	4	3	5
4	5	4	3	4	4	3	2	4
4	4	3	3	4	4	4	3	4
1	1	1	1	2	5	1	5	2
3	3	3	3	3	3	3	5	3
4	5	4	3	4	3	1	1	5
5	5	5	5	5	1	3	1	5
3	3	4	3	3	4	4	3	4
3	3	3	3	3	3	3	3	3
3	4	3	3	3	3	4	1	4
4	4	2	3	3	3	3	2	3
4	5	3	3	3	4	2	2	5
4	5	4	4	4	4	2	1	5
3	5	3	3	3	4	3	3	3
4	5	4	4	4	2	4	1	5
3	5	4	4	4	4	3	2	4
2	5	3	4	4	2	5	2	4
5	5	5	5	5	2	2	1	5
3	2	3	4	4	4	1	2	4
5	5	5	5	4	3	1	2	5

3	4	4	2	4	4	1	2	4
4	4	4	3	5	5	3	3	4
3	5	3	3	4	5	1	3	4
1	1	1	1	1	5	1	5	1
4	4	3	3	3	4	4	2	4
2	3	2	2	2	4	2	3	2
4	5	3	2	3	3	2	3	4
3	5	3	3	3	4	4	4	4
4	5	4	2	4	2	1	1	5
3	5	3	2	2	2	4	4	4
4	4	4	4	5	3	4	2	4
3	2	3	2	3	3	1	2	4
3	3	3	3	4	3	2	3	5
5	5	5	5	5	2	4	1	5
5	5	5	5	5	1	4	1	5
3	3	3	3	3	3	3	3	3
4	5	4	3	4	4	4	4	5
5	5	4	4	5	2	2	2	4
4	3	3	3	4	3	2	2	4
2	4	2	3	4	3	2	2	4
4	5	4	3	3	4	3	3	4
2	2	2	2	2	4	2	4	3
5	5	5	5	5	5	5	5	5
4	5	4	3	5	5	5	3	5
4	5	2	4	3	4	2	3	4
3	3	3	3	3	3	3	3	3
3	3	3	3	3	3	3	3	3
5	5	4	4	4	1	4	1	5
3	3	3	3	3	3	3	3	3
3	4	3	3	3	3	3	3	4
3	3	3	3	3	3	3	3	3
2	4	3	2	3	4	3	4	4
3	4	2	2	3	4	4	5	5
4	4	4	4	4	2	4	2	4
3	4	4	3	4	4	3	3	3
4	4	4	4	4	2	4	2	4
4	5	5	5	5	5	5	1	5
4	4	4	4	4	2	4	2	3
4	4	2	3	2	4	2	4	4
3	3	3	3	3	3	3	3	3
4	4	4	3	4	4	3	2	4
4	4	5	4	5	3	1	1	5
3	4	2	2	3	4	3	2	4
4	5	5	5	4	2	2	2	5
2	3	1	1	1	3	3	3	3
4	4	2	2	4	4	4	4	4
5	5	5	5	5	2	4	2	5
4	5	4	4	4	3	4	1	4
4	5	5	5	5	2	4	1	5
5	5	5	4	5	2	3	1	5
5	5	4	4	4	3	3	2	5
4	4	4	4	4	2	2	2	4
5	5	5	5	5	2	4	1	5
5	5	4	4	4	4	4	2	5

3	5	5	4	3	3	3	2	4
4	4	4	4	4	2	4	2	4
4	4	4	3	4	4	2	4	4
5	5	5	5	5	2	2	2	5
5	5	5	5	5	1	2	1	5
5	5	4	4	4	4	4	2	5

C51	C52	C53	C54	C55	C56	C57	C58	C59
5	5	5	5	5	5	5	5	5
4	2	2	3	1	1	3	1	4
5	5	5	5	5	5	5	5	5
5	5	5	5	5	5	5	5	5
3	3	3	3	3	3	3	3	3
5	5	5	4	5	5	5	5	5
5	5	5	5	5	5	5	5	5
1	2	2	5	5	2	3	2	4
5	5	5	5	5	5	5	5	5
5	5	5	5	2	3	1	1	4
5	5	5	5	4	4	3	2	4
3	4	3	4	4	2	3	2	3
5	5	5	5	1	5	1	1	5
5	5	5	5	5	5	5	5	5
4	5	3	5	5	5	4	3	2
5	5	5	5	5	5	5	5	5
5	4	3	4	4	2	2	1	3
5	5	5	5	5	5	5	5	5
4	4	3	4	4	3	3	2	4
5	4	3	4	4	2	2	2	5
5	5	5	5	5	5	5	5	5
5	5	5	5	5	5	5	5	5
5	5	5	5	2	5	2	1	5
5	5	5	5	4	1	1	1	5
4	2	2	2	4	4	4	4	2
4	4	4	4	4	3	3	2	4
3	3	3	3	3	3	2	2	1
4	5	4	4	4	3	4	2	4
5	5	4	5	2	3	2	1	4
5	5	3	4	4	4	4	3	3
5	5	5	5	2	3	2	2	5
5	5	5	5	1	4	1	1	5
5	5	4	4	4	3	2	2	4
4	4	4	5	5	4	4	5	5
5	5	3	4	4	3	2	2	4
5	4	4	4	4	4	4	5	3
5	5	5	4	2	5	4	1	4
5	5	5	5	5	5	5	5	5
5	5	5	5	2	3	1	2	5
5	5	5	5	1	3	1	1	5
4	4	4	4	2	2	2	1	4
4	4	4	4	2	4	2	2	4
5	5	5	5	2	5	2	2	3
5	5	5	4	2	4	2	1	3
4	4	4	4	4	4	4	2	4
4	4	4	4	4	4	4	4	4
5	5	5	4	4	4	4	4	4
5	5	5	5	5	5	5	5	5
2	2	2	3	2	2	2	2	2
2	4	2	4	4	4	4	4	4
5	5	5	5	5	5	2	2	5
5	4	4	5	3	5	1	1	4
3	4	3	4	3	3	3	2	4

3	3	3	3	3	3	3	3	3
4	4	3	4	4	4	2	2	4
5	3	4	3	4	4	3	5	3
5	5	5	5	5	5	5	5	5
5	5	5	5	2	3	5	2	3
5	5	3	5	5	5	1	1	4
5	5	5	5	1	3	1	1	5
5	5	5	5	5	5	5	5	5
4	4	2	4	5	4	3	2	2
5	4	4	3	5	5	2	1	5
4	4	4	4	3	3	2	2	4
4	4	4	4	4	5	4	2	4
5	5	5	5	1	5	1	1	5
3	2	2	2	2	4	3	3	4
4	4	4	4	2	4	2	2	4
5	5	4	4	3	3	3	1	3
5	5	5	5	1	5	1	1	5
3	5	2	3	2	5	2	4	3
5	5	4	3	4	4	3	2	4
5	3	3	3	4	3	3	2	3
4	5	4	4	3	4	2	2	4
5	4	4	4	2	4	2	2	4
3	3	3	3	3	3	3	3	3
3	3	3	3	3	3	3	3	3
3	3	3	3	3	3	3	3	3
3	4	3	2	5	4	4	3	2
5	4	3	3	4	5	2	2	4
3	3	3	3	3	3	3	3	3
4	3	3	3	3	3	3	3	3
3	3	3	3	3	4	3	2	2
3	3	3	3	4	3	4	3	3
4	4	3	3	2	2	3	2	4
4	5	5	5	2	1	1	1	4
3	3	3	3	3	3	3	3	3
5	5	5	5	3	5	5	5	5
5	5	5	5	1	5	1	1	5
5	5	5	5	4	5	1	1	5
4	3	3	3	4	2	3	2	3
3	3	3	3	3	3	3	3	3
5	5	5	4	1	3	1	1	4
3	3	3	3	3	4	4	2	3
5	5	5	5	3	3	2	2	4
3	3	3	3	5	3	3	3	3
5	5	5	5	1	3	1	1	5
4	4	2	4	3	2	4	2	3
4	4	2	4	3	3	1	1	4
3	4	3	4	3	4	3	4	4
5	3	3	4	3	5	3	2	3
5	5	5	5	1	5	1	1	5
5	2	3	4	3	3	4	3	2
4	2	2	2	4	4	4	2	2
5	5	5	5	1	5	5	1	5
2	2	2	2	2	2	2	2	2
4	4	3	2	4	4	3	3	2

3	4	4	5	4	1	2	3	3
4	1	4	1	5	5	5	5	5
5	5	5	5	4	5	5	1	5
5	5	5	5	1	5	5	1	5
5	5	5	5	1	5	1	1	5
5	4	5	5	4	4	4	1	2
5	3	3	3	4	3	3	2	4
5	3	3	4	4	5	2	1	4
5	5	5	5	2	5	1	1	5
3	3	3	3	3	3	3	3	3
4	3	3	3	3	3	3	3	3
5	5	4	4	3	4	4	1	4
5	5	5	5	1	3	1	2	3
5	5	4	4	2	3	2	2	4
5	5	4	4	2	3	3	3	4
4	4	4	4	4	2	4	2	4
5	5	5	5	3	2	3	3	4
5	5	2	5	4	4	3	2	4
5	4	3	3	3	2	1	2	5
4	4	4	3	3	4	4	4	4
4	4	2	3	4	3	3	2	4
5	5	5	5	1	5	1	1	5
3	3	3	3	3	2	3	3	3
3	3	3	3	3	3	3	3	3
4	2	2	2	4	2	4	3	2
4	2	2	2	4	2	4	3	2
4	3	3	1	4	4	5	2	2
4	3	2	2	5	4	5	5	2
5	3	3	3	5	5	4	2	2
5	5	5	5	1	5	1	1	5
4	4	4	4	3	4	2	2	4
5	4	4	4	2	3	2	2	4
4	3	3	3	3	2	2	3	3
5	1	1	1	5	5	5	5	1
5	5	2	3	2	1	3	1	3
5	5	5	5	1	3	1	1	5
5	3	5	5	2	4	2	2	5
5	5	5	5	5	5	5	2	5
5	5	3	4	4	4	3	1	4
4	4	3	4	4	3	4	4	5
5	4	3	4	2	4	1	1	3
5	2	1	1	5	4	4	2	2
3	3	3	3	3	3	3	3	3
4	1	1	1	5	3	4	3	2
3	5	3	4	4	3	1	1	2
5	5	5	5	2	2	2	1	4
4	3	3	3	3	3	3	3	3
5	5	5	5	2	3	1	1	3
5	5	3	4	4	5	2	2	4
4	3	2	3	4	4	2	2	2
5	4	4	4	3	5	2	1	5
5	5	5	5	5	5	5	5	5
5	4	4	4	5	5	4	3	3
3	4	3	4	3	3	2	2	3

4	4	3	3	3	2	1	1	5
5	4	3	3	4	3	3	2	3
5	5	5	5	1	5	1	1	5
4	3	3	3	4	4	2	2	3
5	4	4	3	5	4	3	1	4
4	4	3	4	2	3	2	2	4
4	4	3	4	3	3	2	2	3
4	4	4	4	2	4	2	2	4
2	2	2	2	4	3	4	4	2
4	4	2	3	4	1	2	1	3
5	5	4	1	4	3	4	1	2
5	5	5	5	1	5	1	1	4
3	3	3	3	3	3	3	3	3
5	5	5	5	1	5	1	1	5
4	3	3	3	3	3	3	3	3
5	5	3	4	5	2	1	1	3
5	5	4	4	4	4	1	1	4
4	4	3	4	2	3	2	2	4
2	1	3	1	1	2	4	1	3
5	5	5	5	1	5	1	1	4
5	3	3	4	4	5	5	2	3
5	5	5	5	4	5	1	1	5
5	3	4	4	4	4	2	1	3
5	5	5	5	1	4	1	1	5
3	3	3	3	5	4	5	3	1
3	3	4	3	5	4	4	4	5
4	4	2	4	4	2	2	2	4
3	3	3	3	2	1	1	4	4
4	1	1	1	5	4	5	5	2
3	5	5	5	5	3	5	1	3
4	4	3	3	4	4	4	2	4
5	5	5	5	1	4	1	1	5
5	5	4	5	1	1	1	1	5
5	5	3	3	4	3	1	1	3
3	3	3	3	3	3	3	3	3
5	5	5	4	2	4	2	1	3
4	3	3	3	3	3	3	3	3
5	5	5	5	2	3	2	2	5
5	4	3	3	4	4	3	2	4
5	5	5	5	2	4	2	2	5
4	4	4	4	4	4	4	3	4
4	4	4	4	4	4	4	4	4
5	5	5	5	5	5	5	5	5
5	5	4	5	2	3	3	2	4
4	3	3	3	3	3	3	3	3
4	4	3	3	4	4	3	2	4
3	3	3	3	3	3	3	3	3
5	5	5	5	1	5	1	1	5
5	5	5	5	1	1	1	1	5
5	5	5	5	1	1	1	1	5
4	5	5	5	1	5	1	1	4
3	4	3	4	2	3	3	1	4
3	3	3	3	3	3	3	3	3
4	3	3	4	4	3	4	4	2

5	5	3	3	3	3	3	3	3
3	3	1	1	1	1	2	2	2
4	4	4	4	4	4	1	4	4
5	3	3	3	4	4	3	2	4
5	5	5	5	1	5	5	2	5
5	5	4	5	2	5	2	2	5
4	4	5	5	5	4	3	3	4
4	4	3	5	4	4	2	1	4
4	4	2	3	4	3	4	2	2
4	4	4	4	2	3	2	2	4
5	5	5	5	4	2	2	2	5
5	5	5	5	3	5	1	1	5
1	1	1	1	1	1	1	1	1
5	5	4	4	3	2	3	2	5
3	3	3	3	3	3	3	3	3
5	5	5	5	5	5	5	1	5
3	3	3	3	3	3	3	3	3
3	3	3	3	2	2	4	4	4
3	3	4	4	4	3	4	4	4
5	5	4	5	5	5	1	1	4
4	4	4	4	2	4	4	2	4
4	4	4	3	4	4	4	2	4
5	5	5	5	2	4	1	1	5
5	5	5	5	4	5	1	1	5
3	3	3	3	3	3	3	3	3
4	3	3	3	4	3	4	3	3
4	3	3	3	4	3	3	4	3
5	5	5	5	1	3	1	1	5
5	5	5	5	2	5	5	5	5
5	5	5	4	4	1	3	5	5
3	4	3	3	3	3	3	3	4
4	1	1	1	5	5	5	5	1
2	2	3	3	4	3	4	2	3
5	1	1	4	1	1	1	1	4
5	3	3	3	2	2	3	2	3
5	4	3	4	4	3	2	1	4
5	4	4	4	4	3	2	1	2
4	4	4	4	4	4	2	2	4
4	4	4	4	4	4	4	4	4
4	5	5	5	2	3	1	1	5
4	4	4	4	2	4	2	2	4
3	3	3	3	4	4	3	1	3
5	5	3	3	5	5	4	3	2
5	5	5	5	1	3	3	1	5
5	5	5	1	1	3	1	1	4
4	3	3	3	3	4	3	2	3
4	3	3	4	4	4	3	2	4
4	5	3	4	4	2	3	2	3
4	5	5	5	2	4	4	4	5
4	4	4	4	4	3	3	3	4
3	3	4	3	4	4	4	3	3
5	5	2	3	1	3	1	1	5
3	3	3	3	3	4	3	3	3
4	4	4	4	4	4	2	2	4

5	5	5	5	4	5	1	1	5
5	1	1	1	1	1	1	1	4
4	4	2	3	1	5	2	4	2
3	3	3	3	3	3	3	3	3
3	3	3	3	3	4	2	1	2
5	5	5	5	1	4	1	1	5
5	5	5	5	1	1	1	1	5
5	5	5	5	4	5	2	1	5
5	3	2	3	5	5	5	2	2
5	3	3	3	5	5	5	3	1
4	4	4	4	4	4	2	2	3
5	5	4	4	2	5	1	1	4
4	2	2	4	4	4	2	2	4
4	4	3	4	4	1	1	1	4
5	5	5	5	4	3	1	1	4
5	5	5	5	2	2	2	5	5
3	3	3	3	3	3	3	3	3
5	5	5	5	4	5	2	2	4
5	4	5	5	1	2	1	5	5
4	3	3	4	4	4	3	2	2
4	4	4	4	2	4	1	1	2
5	4	3	3	4	4	2	2	4
5	5	5	2	2	3	1	1	5
4	2	2	2	4	4	3	2	2
3	3	3	4	3	3	4	3	4
5	3	1	1	5	3	5	3	1
5	5	4	5	3	3	2	2	4
4	3	3	4	4	3	2	4	4
5	3	5	1	3	2	5	5	5
5	5	5	5	2	5	1	1	4
4	5	5	4	2	3	3	1	4
4	4	4	4	4	4	3	1	4
1	1	1	3	3	3	3	3	3
5	5	5	5	5	5	5	5	5
3	3	3	3	3	3	3	3	3
4	3	3	3	4	5	4	3	4
3	2	4	4	1	3	1	1	3
4	4	3	4	2	4	2	2	4
4	3	3	3	4	2	4	4	2
4	3	4	2	3	4	4	4	3
4	4	4	4	4	4	2	2	4
3	3	3	3	4	3	3	3	3
5	4	3	3	3	3	2	2	4
4	4	4	4	4	4	4	2	4
4	4	4	4	4	4	4	4	4
4	2	3	2	4	3	3	3	2
4	4	4	4	3	5	4	4	3
4	4	4	4	2	4	2	2	4
4	3	4	4	2	2	2	2	3
5	4	3	3	5	3	4	1	4
4	3	3	3	4	2	3	2	1
5	5	5	5	4	2	2	2	5
5	5	5	5	5	4	2	1	4
4	2	2	2	4	4	4	4	4

5	5	4	4	3	2	2	1	5
5	5	4	5	4	2	3	2	5
3	3	3	3	3	3	3	3	3
5	5	4	4	2	2	2	2	4
4	4	4	4	4	5	4	4	4
4	4	4	2	4	2	2	4	4
5	5	5	5	2	2	2	1	4
4	5	5	5	5	4	2	2	4
4	4	2	4	4	4	2	2	4
5	4	3	3	3	3	4	1	3
4	3	2	2	4	2	4	2	4
4	4	4	4	2	3	2	2	4
4	4	4	4	1	4	1	1	4
4	4	4	4	3	3	2	2	4
3	3	3	3	4	3	2	2	4
4	4	4	4	4	4	4	4	4
4	4	4	4	4	4	1	4	4
5	5	5	5	2	3	2	2	4
4	4	3	4	4	3	3	2	4
3	3	3	3	3	3	3	3	3
4	4	3	3	3	3	3	1	3
3	3	3	3	3	3	3	3	3
5	5	5	5	3	3	2	1	4
5	5	3	3	4	4	4	2	4
5	5	5	5	4	4	2	2	4
5	5	2	4	2	5	1	1	5
4	4	4	4	4	4	2	2	4
4	2	2	4	5	5	4	2	2
5	2	4	4	4	4	2	1	4
4	3	3	3	4	3	3	3	3
4	5	5	5	4	2	4	2	4
4	4	4	4	4	3	2	2	4
4	4	4	4	4	4	2	3	4
4	3	3	3	4	4	3	3	4
4	4	4	4	2	4	2	2	4
3	3	3	3	4	4	3	2	2
5	2	2	2	5	2	3	1	3
4	4	4	4	4	4	4	4	3
5	4	3	4	3	4	2	2	4
5	5	5	5	4	5	3	3	3
5	2	2	2	4	4	4	2	2
4	4	4	4	4	4	4	2	2
4	3	3	3	4	3	4	3	3
4	3	3	4	4	4	4	2	3
5	3	3	4	5	4	4	2	3
4	4	4	4	3	4	4	4	4
5	3	3	3	5	3	4	1	3
5	5	5	5	2	2	1	2	5
5	5	5	5	1	2	1	2	5
4	4	4	3	4	4	4	2	4
5	5	5	5	1	3	1	1	5
5	5	5	5	2	5	5	1	4
4	4	4	4	4	5	5	3	5
3	3	3	4	4	4	2	2	4

4	4	4	4	3	3	2	2	4
5	5	5	5	1	5	2	1	5
5	5	5	5	4	5	4	2	4
2	2	1	2	5	4	4	3	3
4	3	3	3	4	4	4	2	2
5	5	5	5	1	5	2	2	5
4	4	3	4	4	4	2	1	4
5	4	2	1	3	4	2	1	4
4	4	3	4	4	4	2	2	4
4	1	1	4	1	2	4	3	2
5	4	4	4	3	3	3	2	4
5	4	3	3	4	2	3	2	4
4	4	4	4	2	2	2	2	4
5	4	4	4	5	3	4	2	4
5	5	3	3	3	3	3	1	3
4	3	3	4	4	3	3	3	3
4	2	2	4	4	4	4	1	2
3	3	3	3	3	3	3	3	3
5	5	5	5	1	5	5	5	5
3	4	4	5	4	5	3	3	3
4	4	4	3	3	4	4	3	3
5	5	5	5	1	4	1	1	5
5	4	5	5	3	5	3	2	4
5	5	5	5	2	5	2	2	5
1	1	1	1	5	5	5	3	3
3	3	2	3	4	4	3	3	2
5	5	5	5	2	5	3	1	5
5	3	5	3	4	5	3	1	3
4	4	3	3	4	4	3	2	3
3	3	3	3	3	3	3	3	3
5	5	4	3	4	3	1	1	4
3	3	3	3	3	3	3	3	3
5	5	5	5	2	4	2	2	4
2	2	2	1	5	4	5	4	1
4	2	3	2	4	4	3	2	2
4	3	2	3	4	4	4	2	2
5	4	4	4	4	3	2	1	1
4	4	4	4	3	4	2	2	2
4	3	3	3	3	4	2	2	5
5	2	2	2	5	5	5	2	4
2	5	3	3	4	4	1	1	4
3	3	3	3	3	3	3	3	3
5	3	2	3	4	4	4	2	3
5	5	4	4	4	1	4	4	3
4	4	4	4	4	4	4	4	4
4	4	4	4	4	2	4	2	4
4	4	4	4	2	2	2	2	4
1	1	1	1	1	1	2	1	5
4	4	4	4	1	1	1	1	4
4	4	4	4	3	5	1	1	4
3	3	3	3	3	3	3	3	3
3	3	3	5	5	5	3	3	4
4	2	2	3	4	3	4	4	3
5	4	3	3	4	2	3	2	3

4	4	4	4	4	4	2	2	4
4	4	2	3	4	4	4	3	3
3	3	3	3	3	3	3	3	3
4	4	4	4	2	4	3	2	4
5	3	3	2	5	1	5	5	1
5	4	3	4	3	2	3	1	3
5	5	5	5	2	4	2	1	4
3	3	3	3	3	3	2	2	3
5	5	5	5	1	4	1	1	5
5	5	5	5	1	1	1	5	5
4	4	4	4	4	2	4	4	4
4	3	4	4	4	4	5	3	5
5	5	5	5	3	3	1	1	5
4	4	3	4	4	4	4	4	4
4	4	4	4	4	4	4	2	4
5	5	5	5	1	5	1	1	5
3	3	3	3	3	3	3	3	3
4	4	3	3	4	4	4	3	3
5	4	4	4	5	3	3	1	4
4	4	4	4	4	3	3	2	4
5	3	3	3	4	4	3	3	3
4	4	4	4	4	4	2	2	4
3	3	2	2	4	3	3	2	4
4	4	4	4	4	4	4	2	4
4	4	2	3	4	4	3	2	3
5	1	1	1	5	3	3	3	4
5	5	5	5	1	2	1	1	5
5	3	3	4	4	4	4	3	3
5	5	5	5	4	1	1	1	5
4	4	2	4	4	4	4	4	2
3	3	3	3	3	3	3	3	3
5	5	5	5	2	5	2	2	5
4	4	3	3	3	4	3	2	3
4	4	3	4	2	3	2	2	4
5	5	5	5	1	5	1	1	5
3	3	3	3	4	4	3	2	3
5	5	3	5	2	4	2	2	5
4	4	4	4	3	3	3	3	4
5	4	3	3	5	5	5	5	5
4	4	4	4	4	4	3	2	4
4	4	3	2	3	3	3	2	4
5	4	3	5	4	4	4	4	3
4	2	2	2	4	3	4	2	2
4	4	3	4	4	5	2	2	4
5	5	4	5	2	2	2	1	4
5	5	3	4	1	2	1	1	4
3	3	3	3	4	4	3	4	1
4	4	4	4	2	4	2	2	4
3	3	3	3	4	3	4	4	3
4	3	3	3	4	3	3	3	3
4	4	4	4	2	2	2	2	4
3	3	3	3	3	3	3	3	3
4	4	4	4	4	4	3	3	4
4	3	3	4	4	4	3	2	3

4	4	4	4	4	3	2	4	4
4	3	4	3	3	5	4	4	3
4	4	4	4	4	4	3	2	3
4	2	2	2	4	2	4	1	4
4	4	4	4	4	2	3	2	3
3	4	3	4	2	3	2	2	4
2	2	2	2	3	3	2	3	2
4	2	2	2	4	2	3	3	2
5	3	2	3	3	4	4	4	3
3	3	3	3	3	3	3	3	3
5	5	4	4	4	5	3	2	4
5	4	3	3	4	4	1	1	3
2	5	3	1	2	5	2	3	5
5	5	3	5	5	2	2	1	3
4	4	3	4	3	2	4	2	4
5	4	2	3	3	3	1	1	4
4	2	3	3	4	4	4	2	2
4	4	3	3	4	2	3	2	3
4	5	2	4	4	3	2	1	4
5	4	4	4	4	5	2	1	4
4	3	3	3	4	3	3	3	2
4	4	4	4	4	4	4	4	4
3	4	3	2	2	3	3	3	5
3	3	3	3	3	3	3	3	3
5	5	5	5	1	4	2	1	5
5	3	1	3	5	3	3	3	5
4	3	3	3	3	4	3	2	4
4	4	4	4	3	4	2	2	3
3	4	4	4	3	3	2	1	4
4	4	3	3	4	1	3	2	3
1	4	3	4	4	3	3	3	3
4	4	4	4	2	3	2	2	4
4	4	4	4	4	4	4	4	4
4	4	4	4	3	4	2	3	3
4	5	4	4	3	4	2	2	4
3	3	3	3	3	3	3	3	3
5	5	5	5	5	5	5	5	5
5	5	5	5	3	1	1	1	5
4	2	4	3	4	4	4	4	3
5	5	5	5	3	5	5	1	5
4	4	4	4	4	4	4	4	4
3	2	2	3	2	3	3	2	2
4	5	4	4	2	3	2	2	2
4	4	4	4	4	3	2	2	4
4	4	3	4	4	4	4	4	4
4	2	2	3	4	4	3	3	3
4	4	4	4	2	4	2	2	4
5	4	4	4	2	3	3	2	4
4	4	4	4	1	4	1	2	4
5	5	5	5	4	4	2	2	4
4	4	3	4	2	4	2	2	4
4	4	4	4	3	3	3	3	3
5	5	5	5	3	5	2	2	5
5	5	5	5	3	1	1	1	5

5	5	5	5	5	5	1	1	5
5	5	5	5	2	5	1	1	5
4	4	3	3	2	2	1	2	4
4	4	4	4	4	4	2	2	4
5	5	5	5	1	4	1	1	5
5	5	5	5	1	5	1	1	5
5	5	5	5	4	2	2	2	4
5	5	5	5	2	4	2	1	5
5	5	3	3	4	2	3	1	3
4	5	5	5	3	5	1	1	5
3	4	4	4	1	3	1	1	5
2	3	2	2	4	1	1	1	3
4	4	4	4	3	4	4	2	4
4	4	4	3	2	4	2	2	4
4	4	4	4	4	4	2	2	4
2	2	2	2	4	3	2	2	3
5	4	4	4	4	4	2	2	4
5	5	5	5	1	3	1	1	4
5	4	3	4	2	2	2	2	2
5	5	5	5	3	3	2	1	5
5	5	5	5	1	1	1	1	5
1	1	4	3	3	3	4	3	3
4	4	4	4	5	3	3	3	3
5	5	3	4	3	2	3	3	3
4	5	4	4	5	5	5	4	5
3	3	3	3	3	3	3	3	3
3	4	3	3	4	3	4	3	4
5	4	2	4	4	3	2	1	4
4	4	4	4	3	4	2	2	4
2	1	1	1	5	4	5	5	3
4	4	4	4	2	3	2	2	4
4	4	3	3	4	1	4	2	2
5	5	5	5	1	5	5	5	5
4	4	4	4	4	4	2	2	4
4	5	5	5	3	2	2	2	4
5	5	5	5	1	4	1	1	4
5	4	4	2	2	3	2	1	4
4	4	4	4	2	4	4	4	4
3	5	5	5	1	3	1	1	5
4	4	4	4	2	3	2	2	4
5	5	5	5	5	5	5	5	5
3	3	3	3	3	3	3	3	3
4	5	4	3	2	3	2	2	4
3	3	3	3	3	3	3	3	3
5	5	5	5	2	4	1	1	4
3	3	3	3	3	3	3	3	3
5	3	5	5	5	5	3	1	1
3	3	3	3	3	3	3	3	3
4	4	3	3	4	3	3	3	4
5	5	4	3	3	3	3	3	3
3	3	3	3	3	5	3	3	3
4	3	2	3	3	4	2	2	3
4	4	4	4	2	4	2	2	4
5	5	5	5	1	5	1	1	4

5	5	5	5	5	5	1	1	5
5	5	5	5	2	5	1	1	5
5	5	5	5	2	2	2	2	3
4	4	4	4	2	4	2	1	3
3	2	3	3	4	3	4	3	5
5	5	3	5	4	5	1	1	5
5	4	3	4	5	3	3	2	4
5	5	5	5	4	5	1	1	5
5	5	5	5	5	5	5	5	5
5	5	5	4	1	5	1	1	4
4	4	4	3	4	3	2	2	4
4	4	3	2	2	2	3	4	5
5	5	5	5	5	1	1	1	5
3	3	3	3	3	3	3	3	3
4	4	4	4	4	4	2	2	4
3	3	3	1	1	1	1	1	1
5	5	5	3	1	3	2	2	3
3	4	4	4	2	5	5	5	5
2	1	1	3	4	3	3	3	3
3	4	4	3	2	3	1	1	4
4	3	3	2	2	4	3	2	3
5	4	4	4	2	4	2	2	4
5	5	5	5	5	1	1	1	5
5	5	5	5	1	5	1	1	4
3	3	3	3	3	3	5	4	3
5	5	5	5	2	3	3	3	4
5	5	5	5	1	4	1	1	5
5	2	2	2	5	3	4	3	4
4	4	3	4	4	4	4	2	4
4	4	4	3	2	2	2	3	4
3	3	3	3	3	3	3	3	3
5	5	5	5	1	5	1	1	5
4	4	4	4	4	4	2	2	4
5	5	5	5	1	5	1	1	5
4	4	4	4	4	4	4	4	4
4	2	2	2	4	3	4	3	3
4	3	3	3	3	3	3	3	3
5	5	5	5	1	5	1	1	5
4	4	2	4	4	4	2	2	4
4	5	5	5	2	3	1	1	5
4	4	4	4	2	3	3	2	4
5	5	5	5	1	5	1	1	5
5	5	5	5	5	5	1	1	5
5	5	5	5	2	5	1	1	5
3	3	3	3	3	3	3	3	3
4	4	4	3	4	3	2	2	4
5	5	4	4	4	3	3	1	1
5	3	5	5	5	3	1	2	2
4	3	3	3	4	4	3	2	2
5	5	5	5	1	1	1	1	5
3	3	3	3	3	3	2	3	4
5	5	4	4	2	3	1	2	5
5	5	5	5	1	1	1	1	5
5	4	5	3	3	4	2	2	4

3	3	3	3	3	3	3	3	3
4	4	3	3	2	4	1	1	3
5	4	4	4	2	3	2	1	4
3	3	3	3	3	3	3	3	3
5	5	5	5	5	5	5	5	5
5	5	5	5	2	1	1	2	4
3	3	3	1	5	3	3	5	1
3	3	4	2	2	2	4	2	2
4	4	4	4	2	4	2	2	5
4	4	4	5	1	1	5	1	5
5	5	5	5	1	5	1	1	5
4	4	3	4	4	4	4	2	4
5	5	5	5	1	5	1	1	5
5	4	4	4	4	4	4	4	4
5	4	4	5	3	2	1	1	4
5	2	5	5	4	5	5	2	1
5	4	3	4	3	2	1	1	3
5	5	5	5	1	5	1	1	5
4	4	3	3	4	5	3	2	4
3	3	3	3	3	3	3	3	3
4	4	4	4	4	3	2	1	3
3	3	3	3	3	3	3	3	3
5	2	5	2	4	2	5	5	5
5	5	5	5	1	5	1	1	5
4	3	4	4	4	4	3	3	3
5	4	4	5	2	3	3	3	3
2	1	5	3	2	2	4	1	2
5	3	2	3	3	4	3	3	3
3	4	4	4	2	2	2	2	4
4	3	3	4	4	4	4	3	4
4	4	4	4	2	3	3	2	4
3	3	3	3	3	3	3	3	3
4	4	4	4	4	4	2	4	4
3	3	3	3	4	4	3	3	3
3	3	3	3	3	3	3	3	3
4	4	4	4	2	4	2	2	4
4	3	3	3	4	5	3	2	2
5	5	4	4	3	3	3	1	3
3	3	3	3	3	3	3	3	3
3	3	3	3	3	3	3	3	3
5	2	2	3	5	5	5	5	2
4	4	3	4	3	3	2	2	4
5	5	5	5	2	4	2	1	4
4	3	3	3	5	5	3	2	2
4	3	3	2	4	4	4	3	3
5	5	5	5	1	5	1	1	5
4	2	2	3	4	4	4	2	3
4	4	4	4	2	4	2	2	4
4	4	4	4	3	4	3	2	4
5	3	3	4	4	4	3	1	3
5	5	4	4	2	4	2	2	4
4	4	4	4	2	2	2	2	4
3	3	3	3	3	3	3	3	3
4	3	3	4	4	4	3	2	3

5	4	3	3	4	4	3	2	4
4	3	3	3	4	4	3	2	3
4	4	4	3	4	3	4	3	3
4	4	4	4	2	4	2	2	4
4	2	2	2	4	3	4	4	3
3	3	3	5	5	1	3	1	5
4	4	2	2	4	4	4	2	4
3	3	3	3	3	3	3	3	3
5	5	4	5	3	5	1	1	4
5	5	5	5	2	4	2	2	5
4	4	4	4	2	4	2	2	4
1	1	1	1	4	1	1	1	1
3	3	3	3	2	2	2	2	4
4	2	3	3	4	3	3	3	4
5	5	5	4	1	4	1	1	4
5	3	2	4	2	3	2	3	3
4	4	4	4	2	3	2	2	4
4	3	4	2	3	3	2	4	4
4	4	3	4	4	2	2	2	4
1	2	1	2	5	1	4	4	2
1	5	5	5	5	5	5	5	5
3	3	3	3	3	3	3	2	3
4	3	4	3	4	4	3	2	4
4	4	3	4	4	4	2	2	4
4	4	4	4	4	4	3	3	4
5	5	3	3	3	3	3	2	3
3	3	3	3	3	3	3	3	3
5	5	1	2	5	5	5	2	1
4	2	2	2	4	2	3	2	2
4	4	4	4	2	3	2	2	4
4	4	3	3	4	3	4	2	4
4	4	3	4	4	4	2	2	4
3	3	3	3	3	3	3	3	3
5	5	5	5	5	5	5	5	5
4	4	4	2	3	3	2	3	3
3	3	3	3	1	3	2	2	4
4	4	4	4	4	4	3	4	4
4	4	4	4	4	4	4	4	4
4	3	2	2	2	4	4	2	4
2	3	3	3	4	2	4	3	2
5	5	5	5	2	5	1	1	5
4	4	3	4	4	3	3	2	4
4	4	4	4	4	4	4	4	4
4	4	3	4	5	4	3	2	3
4	4	4	4	4	2	3	2	3
3	2	3	4	4	3	4	3	2
4	4	3	4	4	3	2	2	4
4	3	3	3	4	3	3	2	3
4	2	2	4	5	5	2	2	4
4	4	3	4	4	4	3	4	4
4	4	4	4	2	4	2	2	4
5	5	4	5	2	5	1	1	4
3	4	4	4	1	4	1	1	4
4	4	4	5	3	4	2	1	5

4	3	3	3	4	3	3	2	4
4	4	4	4	4	3	2	2	3
4	4	3	3	4	3	3	3	4
4	4	4	3	3	4	3	3	3
4	4	4	2	2	2	3	2	4
4	4	3	4	4	3	2	2	3
4	2	4	4	1	5	3	1	2
5	5	5	5	2	2	2	2	5
5	5	5	5	1	4	1	1	5
5	5	5	5	2	4	1	1	4
5	5	5	5	1	5	1	1	4
5	5	4	5	3	5	1	1	5
4	4	4	4	3	3	2	2	4
3	3	3	3	3	3	3	3	3
5	5	5	5	1	3	1	1	5
4	4	2	2	4	2	2	2	4
5	5	4	5	1	3	1	1	5
4	4	4	4	4	3	4	3	2
5	3	3	3	4	5	3	2	3
3	4	1	3	4	5	4	4	2
5	5	4	4	3	4	2	1	4
5	3	3	5	3	3	3	1	3
5	5	5	5	1	5	1	1	5
5	5	5	5	1	3	1	1	5
5	5	5	5	1	5	1	1	5
4	3	3	2	4	4	4	3	2
4	4	3	4	2	4	2	2	4
5	5	5	5	4	5	1	1	5
4	4	4	4	5	3	3	2	3
5	5	5	5	1	5	1	1	5
5	4	2	3	3	1	1	1	4
5	4	2	4	4	2	2	1	4
3	3	3	4	4	4	4	4	4
5	5	5	5	1	4	1	1	5
5	5	3	3	4	5	2	1	3
5	5	5	5	2	4	2	1	4
4	4	4	3	3	4	3	2	4
1	1	1	1	5	1	5	5	1
3	1	1	3	3	3	3	3	3
5	4	4	4	2	4	1	1	4
5	5	5	5	1	3	2	1	5
3	3	3	3	4	4	4	3	4
3	3	3	3	3	3	3	3	3
4	3	3	4	3	3	2	1	4
4	2	2	3	4	2	4	2	3
5	4	2	3	4	4	3	2	2
5	5	4	4	4	3	2	2	2
4	3	3	3	3	4	4	3	3
5	4	4	4	2	4	2	2	4
5	3	2	4	4	4	4	2	3
4	5	5	5	2	5	2	2	3
5	5	5	5	1	3	1	1	5
3	3	2	2	4	1	2	1	3
5	5	5	5	3	4	1	1	4

4	4	3	3	4	4	4	2	4
4	4	3	5	4	5	1	1	3
3	3	3	4	2	3	3	1	4
1	1	1	1	5	1	5	5	1
3	3	3	3	4	3	3	2	3
2	2	2	2	4	2	4	3	2
5	4	4	3	4	3	2	3	3
5	3	3	3	4	2	4	2	4
5	5	5	4	3	4	2	2	5
4	3	2	3	4	3	4	3	2
4	4	4	4	2	3	2	2	4
4	3	3	3	4	3	4	2	3
5	3	3	3	4	3	2	2	3
5	5	5	5	1	5	1	1	5
5	5	5	5	1	4	1	1	5
3	4	3	3	3	3	3	3	3
5	5	3	4	4	4	2	2	4
5	5	5	5	1	4	2	1	5
3	2	2	2	2	2	2	3	3
4	2	2	2	4	4	2	2	3
4	4	4	3	4	4	4	2	3
2	2	2	2	4	3	4	2	3
5	5	5	5	5	5	5	5	5
5	5	5	5	5	5	5	3	3
5	4	2	3	4	5	3	2	4
2	3	3	3	4	3	3	3	3
3	3	3	3	3	3	3	3	3
5	5	5	5	1	1	1	1	4
3	3	3	3	3	3	3	3	3
4	4	4	4	3	3	3	3	3
3	4	3	3	3	3	3	3	3
5	2	3	3	4	3	3	3	2
5	3	3	2	3	3	3	3	4
3	4	3	3	2	3	3	2	3
3	3	4	3	4	4	3	3	4
4	4	4	4	2	3	2	2	4
5	5	5	5	4	4	2	1	5
4	4	3	3	2	3	2	2	3
3	3	3	3	3	3	3	3	3
3	3	3	3	3	3	3	3	3
4	4	4	4	4	3	2	2	4
5	5	5	5	2	1	3	1	5
4	4	4	3	4	3	3	2	3
5	5	5	4	3	2	2	2	2
3	3	3	3	3	3	3	3	3
5	2	2	2	5	2	2	3	4
5	5	5	5	2	4	1	1	5
5	4	3	4	1	4	2	1	3
5	5	5	4	4	2	1	1	5
4	4	3	4	1	3	2	1	4
5	5	4	4	3	1	3	2	4
4	4	3	4	3	3	2	2	4
4	4	4	5	5	3	1	1	5
5	4	2	4	4	4	2	1	3

4	4	3	4	3	4	2	2	4
4	4	4	4	3	4	2	2	4
4	4	3	3	4	4	3	2	4
5	5	5	5	2	5	2	1	5
5	5	5	5	2	4	2	2	5
5	4	2	4	4	4	2	1	3

C60	C61	C62	C63	C64	C65	C66	C67	C68
5	5	5	5	5	5	5	3	4
1	4	2	1	3	2	5	5	2
5	5	5	5	5	5	5	5	5
5	5	5	5	5	5	5	5	5
3	3	3	3	3	3	3	3	3
5	5	5	5	4	4	5	5	4
5	5	5	5	5	5	5	5	5
3	4	3	2	4	4	1	3	3
1	1	1	1	1	1	1	5	5
5	5	5	5	5	4	5	5	4
3	4	2	2	4	4	2	5	5
2	4	2	2	2	2	2	2	3
5	5	1	1	5	5	1	5	5
5	5	5	5	5	5	5	5	5
2	2	5	5	3	5	5	5	5
5	5	5	5	5	5	5	5	5
2	4	3	1	2	4	2	3	4
5	5	5	5	5	5	5	5	5
3	4	4	4	4	3	3	3	3
2	5	2	2	2	5	4	2	5
5	5	5	5	5	3	2	3	4
5	5	5	5	5	5	5	5	4
5	4	4	1	4	4	1	4	5
4	5	1	1	4	4	1	4	4
2	2	3	2	2	2	4	2	2
2	3	2	2	3	3	2	3	4
1	1	3	3	3	5	3	3	3
3	4	2	2	3	3	2	4	4
3	2	2	1	1	1	1	2	4
3	3	2	2	3	3	2	3	3
5	5	3	1	5	5	1	3	5
5	5	1	1	5	5	1	5	5
3	4	2	2	4	3	2	3	5
5	5	5	5	4	3	4	5	4
2	5	3	2	4	4	1	2	3
3	3	3	3	3	3	3	3	3
5	4	2	1	5	4	1	5	5
5	5	5	5	5	5	5	5	5
5	5	1	1	5	5	1	5	5
5	5	1	1	5	5	1	5	5
4	4	1	1	4	4	1	4	4
4	4	2	2	4	4	2	3	4
5	5	2	1	5	5	2	3	5
3	4	2	1	3	4	1	3	3
3	4	3	3	3	4	2	4	3
4	4	4	4	4	4	4	4	4
4	4	4	4	4	4	4	4	4
5	5	5	5	5	4	4	4	4
3	2	2	2	2	2	2	2	2
4	4	1	1	1	1	1	1	1
1	5	2	3	3	2	3	3	3
5	4	2	1	3	5	1	5	5
5	4	3	1	4	4	2	4	5

3	3	3	3	3	3	3	3	3
3	4	3	2	3	4	2	3	4
3	3	5	4	4	5	4	3	4
5	5	5	5	5	5	5	5	5
5	5	3	5	2	2	4	3	5
3	5	2	1	1	4	1	3	5
5	5	1	1	5	5	1	5	5
5	5	5	5	5	5	5	5	5
2	4	2	2	4	4	2	4	4
3	3	4	2	2	3	2	4	4
3	4	2	2	3	4	2	3	4
4	4	5	1	4	4	2	3	4
5	5	3	1	5	5	5	3	5
2	2	5	1	4	5	4	3	5
4	4	2	2	4	4	2	4	4
3	3	1	1	3	4	2	3	5
5	5	3	1	5	1	1	1	5
3	3	3	3	3	3	3	3	4
3	4	4	2	4	4	2	4	4
3	3	3	2	2	3	2	3	3
3	4	2	2	4	4	2	3	4
4	3	2	1	4	4	1	4	4
3	3	3	3	3	3	3	3	3
3	4	4	3	3	3	3	3	3
3	3	3	3	3	3	3	3	3
1	1	4	2	2	2	3	2	2
3	4	2	1	4	3	2	3	4
3	3	3	3	3	3	3	3	3
3	3	3	2	3	3	3	3	3
2	3	4	3	3	3	3	3	3
2	3	3	3	1	3	3	3	2
3	3	4	2	3	3	2	4	3
4	5	2	1	5	5	1	5	5
2	2	3	3	2	2	3	2	2
5	5	2	5	5	5	5	5	5
5	5	4	1	5	5	1	4	5
5	5	1	1	5	5	1	5	5
2	2	3	1	3	2	2	4	4
3	3	3	3	3	3	3	3	4
4	4	4	1	4	3	1	3	4
3	2	4	3	3	3	4	2	4
3	4	2	2	4	4	2	4	4
3	3	4	3	3	3	4	2	3
5	5	1	1	4	5	1	4	5
3	3	4	2	2	2	4	4	4
2	4	4	2	3	4	4	3	3
5	3	2	4	3	4	4	3	4
3	3	3	2	2	2	3	3	4
5	5	2	1	5	5	1	5	5
1	2	4	2	2	2	2	2	3
2	4	2	2	2	3	3	2	3
5	5	1	1	5	5	1	5	5
2	2	2	2	2	1	2	2	2
3	3	4	2	3	2	4	1	3

4	2	3	1	1	2	1	4	2
1	1	3	5	1	1	5	1	2
5	5	4	1	5	5	1	5	5
5	5	1	1	5	5	5	1	5
5	5	1	1	5	5	1	4	5
2	4	4	1	3	4	4	2	5
2	4	4	1	3	3	1	3	2
4	4	2	1	4	4	2	2	4
5	5	1	1	5	5	1	2	5
3	3	3	3	3	3	3	3	3
3	3	3	3	3	3	3	3	3
4	4	2	2	4	4	1	3	4
3	4	1	1	3	3	1	3	4
3	4	2	2	3	4	2	4	4
4	4	3	2	3	3	2	3	4
4	4	4	2	4	4	2	4	3
3	2	3	2	3	4	2	4	4
2	4	2	2	4	4	2	4	4
3	1	3	1	3	2	2	3	4
3	3	3	2	4	4	4	4	4
3	4	4	2	3	4	2	4	4
5	5	1	1	5	5	1	5	5
3	3	3	3	2	3	3	3	4
3	3	3	3	3	3	3	3	3
2	4	4	2	2	2	4	2	4
2	2	4	2	2	3	4	2	4
2	4	5	2	3	3	3	3	3
1	2	4	3	1	3	3	2	2
2	4	3	2	3	3	4	2	4
5	5	1	1	5	5	1	4	5
4	4	2	2	4	4	2	3	4
3	3	2	1	4	4	4	3	4
3	3	4	3	3	3	4	4	3
1	1	5	5	1	1	5	1	1
1	1	1	2	2	2	1	3	3
4	4	1	1	4	3	2	3	4
3	4	5	1	5	3	1	5	5
5	5	4	2	2	4	2	2	4
2	2	3	1	3	4	1	4	4
1	1	2	3	4	3	4	3	2
2	3	2	2	4	4	2	4	5
1	2	5	2	1	1	4	1	3
3	3	3	2	3	3	4	2	2
2	4	4	3	2	1	5	1	3
1	1	3	1	3	3	3	3	4
3	4	2	1	3	3	1	3	5
3	3	3	3	3	3	3	3	3
3	5	1	1	5	4	1	5	4
3	5	3	2	3	4	2	2	4
2	4	4	2	2	4	2	3	4
3	4	2	1	3	5	1	2	5
5	5	3	3	3	3	3	3	5
2	3	4	1	3	3	5	2	4
4	3	2	2	3	3	2	3	4

3	4	2	1	3	4	1	4	4
2	2	2	1	2	3	1	2	4
5	5	1	1	5	5	1	4	5
2	3	4	2	2	2	3	2	4
4	3	2	1	2	5	1	3	5
3	4	2	2	3	4	2	4	4
3	4	3	2	3	4	2	3	4
4	4	2	2	4	4	2	4	4
2	2	4	3	2	2	4	2	2
2	4	2	1	4	3	1	4	4
2	5	3	1	3	3	1	2	4
5	5	1	1	4	5	1	5	5
3	3	3	3	3	5	1	5	5
5	4	1	1	5	5	1	5	5
3	3	3	2	3	3	3	3	3
3	4	3	1	3	3	1	4	4
3	4	2	1	4	4	1	4	4
3	3	2	2	3	3	2	3	4
4	3	3	3	2	4	2	4	3
4	5	3	1	5	5	1	5	4
2	4	4	2	2	5	2	2	5
5	5	2	1	4	5	1	3	5
3	5	4	1	2	3	3	2	4
5	5	2	1	4	1	1	2	1
1	2	5	3	1	1	1	1	2
5	1	1	1	3	1	5	5	1
3	4	2	2	3	3	2	4	4
3	3	3	3	2	2	3	3	3
1	1	4	2	1	1	5	1	4
3	3	3	4	4	5	4	5	5
3	3	3	3	3	3	3	3	4
5	5	1	1	5	5	1	1	5
5	5	1	1	4	3	1	3	5
4	5	1	1	4	3	3	2	5
3	4	2	1	3	4	1	4	4
2	4	2	5	5	3	1	4	3
3	3	3	3	3	2	3	3	3
4	5	1	1	5	5	1	5	5
3	3	2	1	4	3	2	4	4
5	5	1	2	5	4	1	5	5
4	4	4	1	4	4	3	3	4
2	4	2	2	2	2	2	2	4
2	5	5	5	5	5	5	5	5
4	4	2	2	4	4	3	2	5
3	3	3	3	3	3	2	3	4
3	4	4	2	3	4	2	4	4
3	3	3	3	3	3	3	3	3
5	5	1	1	5	5	1	5	5
5	5	1	1	5	5	1	2	5
5	5	1	1	5	4	1	5	5
5	4	1	1	5	5	1	2	5
4	4	4	4	4	4	4	4	4
3	3	3	3	3	3	3	3	3
2	4	5	2	2	3	2	2	3

3	3	3	3	3	3	3	3	3
4	2	1	4	1	2	1	2	2
4	4	3	1	4	4	1	4	4
2	2	4	2	3	3	2	3	4
5	5	2	1	5	5	5	4	3
5	5	2	2	5	5	2	5	5
4	5	4	4	4	4	4	4	4
3	4	1	1	5	1	4	5	5
2	4	2	2	2	4	2	2	4
4	4	4	2	4	2	2	2	2
3	4	3	1	3	4	2	2	3
5	5	1	1	5	5	1	5	5
1	1	1	1	1	1	1	1	1
4	1	2	1	3	3	2	4	4
3	3	3	3	3	3	3	3	3
4	4	3	2	5	5	3	4	3
3	3	3	3	4	3	3	3	3
2	4	4	4	4	3	3	4	3
3	2	3	2	3	3	4	4	3
3	5	4	1	4	4	1	4	5
4	4	4	2	4	4	2	4	4
4	4	2	2	4	4	2	2	3
4	5	1	1	5	5	1	5	5
3	5	3	1	4	4	1	5	4
3	3	3	3	3	3	3	3	3
3	4	3	3	3	3	3	4	3
3	3	3	2	3	3	3	3	3
3	3	2	1	3	5	3	5	5
5	5	4	5	5	5	5	5	5
2	4	2	1	2	5	1	3	5
3	3	3	3	3	3	3	3	3
1	1	5	5	1	1	5	1	2
3	3	3	3	2	4	4	4	3
4	4	2	4	4	4	2	4	5
3	4	2	1	3	3	1	3	4
3	3	4	1	3	3	1	3	4
2	4	2	2	3	4	2	3	4
2	2	2	1	1	4	1	3	4
4	4	4	4	4	4	4	4	4
4	5	1	1	4	4	4	4	5
4	4	2	2	2	4	2	2	4
3	3	3	1	3	3	3	3	4
1	4	1	3	1	3	2	3	3
5	3	3	1	5	5	1	5	5
5	3	1	1	3	3	3	2	3
3	3	3	2	3	3	2	3	4
2	3	2	1	3	3	2	2	3
2	4	2	3	4	4	2	4	4
5	5	5	5	5	5	5	5	4
3	4	3	4	5	3	3	3	4
4	4	3	3	3	4	3	3	4
5	4	1	1	5	4	1	4	4
3	3	3	3	3	3	2	3	3
3	4	4	2	3	4	2	2	4

5	5	2	1	5	5	1	4	5
4	4	4	1	4	3	1	4	5
1	3	2	1	2	3	4	4	4
3	3	3	3	3	3	3	3	3
3	2	2	3	1	2	4	5	2
3	5	1	1	5	5	1	3	5
5	3	3	1	4	4	1	4	4
4	4	4	1	3	4	1	3	4
2	1	1	1	3	1	4	3	5
1	1	5	3	1	1	3	1	1
2	4	2	2	3	3	2	4	4
4	4	5	1	3	3	1	5	5
2	4	4	2	2	2	4	2	4
3	3	2	2	4	3	2	3	3
1	2	2	1	4	3	2	3	4
5	5	2	2	5	5	2	5	5
3	3	3	3	3	3	3	3	3
2	4	4	2	4	4	2	2	5
5	5	2	1	2	1	1	1	1
2	2	3	2	3	3	3	3	3
4	4	3	3	4	4	4	4	4
4	4	4	2	2	3	2	4	4
4	5	1	1	5	5	2	2	5
2	4	4	2	2	3	2	4	3
3	2	4	3	3	5	4	5	4
1	1	5	4	1	1	5	3	5
4	4	2	2	4	4	4	4	4
3	4	3	2	3	3	3	3	4
5	5	1	5	5	5	1	5	5
4	4	2	1	3	5	2	4	5
3	5	2	1	5	3	3	2	5
3	4	4	2	3	4	2	3	4
3	3	3	3	3	3	3	3	3
5	5	5	5	5	5	5	5	5
3	3	3	3	3	3	3	3	3
3	4	3	3	4	4	2	1	5
4	4	5	5	5	5	4	4	4
4	4	2	1	4	4	2	3	4
3	4	2	3	1	2	5	2	4
3	3	1	3	3	4	4	4	4
4	4	2	2	4	4	2	4	4
3	3	3	3	3	3	3	3	3
3	4	3	2	2	4	2	3	4
3	4	4	2	4	4	2	3	4
4	4	4	3	4	4	3	3	4
3	2	4	3	2	2	4	3	2
3	3	3	3	3	3	3	3	3
4	4	2	2	4	4	2	4	4
3	3	2	2	2	2	2	2	4
2	4	3	1	2	3	2	2	4
2	3	3	3	3	3	2	3	3
4	4	1	1	3	4	1	4	5
4	5	4	2	4	4	3	2	4
2	2	4	4	1	3	4	2	2

4	4	2	1	4	4	2	3	4
4	4	3	2	4	4	2	5	4
3	3	3	3	3	3	3	3	3
3	4	2	1	5	4	1	1	5
4	4	4	3	4	1	2	1	4
2	4	4	4	2	2	4	2	2
3	4	1	1	4	4	2	4	4
2	5	2	2	2	2	2	4	4
4	4	2	2	4	4	2	4	4
3	3	3	3	3	3	3	3	3
2	4	4	2	3	5	2	3	2
4	4	2	2	4	4	2	4	4
4	2	2	1	4	4	3	1	4
4	3	3	2	4	4	3	3	4
3	4	2	2	3	3	2	2	4
4	4	4	4	4	4	4	4	4
4	4	4	4	4	4	4	4	4
4	4	2	2	3	4	2	2	3
3	4	4	2	2	4	2	3	4
3	3	3	3	3	3	3	3	3
4	2	2	1	3	2	2	3	4
3	3	3	3	3	3	3	3	3
4	4	3	1	5	5	2	5	5
3	3	3	1	2	4	1	2	5
2	4	2	4	3	4	2	4	4
5	5	2	1	4	5	1	4	5
4	4	2	2	4	4	2	4	4
1	4	4	2	2	2	3	2	4
2	4	4	2	3	4	2	4	4
3	3	3	3	3	3	3	3	4
3	4	2	2	4	4	2	2	4
4	2	2	2	4	4	2	2	4
4	4	4	3	4	4	2	4	4
3	3	2	2	3	3	3	3	4
4	4	2	2	4	4	2	4	4
2	3	2	2	2	3	2	2	3
2	4	4	1	2	4	3	1	5
1	4	4	1	3	4	5	3	4
3	3	2	2	3	3	2	3	4
3	4	5	1	3	4	3	3	4
2	2	4	2	2	2	3	2	4
3	3	3	3	3	3	3	3	3
3	3	4	3	2	3	3	3	3
3	3	4	2	3	4	2	2	4
1	5	2	2	4	3	3	2	4
4	4	2	2	4	4	3	3	3
2	4	3	2	3	3	2	2	3
5	5	2	2	5	5	2	3	5
5	4	2	2	5	5	2	4	5
4	3	3	3	4	4	3	4	4
4	4	1	1	5	5	1	5	5
5	5	2	1	4	4	1	4	4
4	4	4	5	5	4	4	4	4
3	3	3	3	3	3	3	3	2

4	4	2	2	4	4	2	4	4
5	5	2	1	5	5	1	5	4
4	4	2	2	4	5	2	3	4
2	3	2	2	2	3	4	3	3
2	4	4	2	2	2	2	2	4
4	4	2	1	5	5	2	4	5
3	4	2	1	3	3	2	4	4
2	1	3	1	2	4	4	1	3
4	4	2	2	2	4	2	2	4
1	1	5	3	2	1	5	1	4
4	4	3	2	3	4	2	3	3
4	4	2	2	3	1	2	3	4
4	4	2	2	4	4	1	3	4
2	4	2	1	5	5	4	1	4
3	3	3	1	3	3	1	3	5
3	3	2	2	3	3	3	3	3
2	4	4	2	3	3	2	3	4
3	3	3	3	3	3	3	3	3
5	5	5	1	5	5	5	5	5
4	4	4	4	4	4	4	3	4
4	4	4	3	2	4	4	2	4
5	5	2	1	5	5	1	2	5
4	4	3	2	5	5	3	4	4
5	5	2	1	5	5	1	5	5
4	3	3	5	5	1	1	1	1
2	4	2	2	2	3	2	2	2
4	4	2	1	5	5	2	5	5
3	5	3	1	3	3	1	5	3
3	3	3	2	3	3	3	5	3
3	3	3	3	3	3	3	3	2
2	5	2	1	4	5	2	3	5
3	3	3	3	3	3	3	3	3
4	4	2	2	4	4	2	4	4
1	1	5	3	1	3	4	2	1
2	2	4	2	2	4	4	2	4
2	4	3	2	2	1	1	1	1
1	4	2	4	4	4	4	4	4
2	4	2	2	4	3	2	2	4
5	5	3	2	3	1	1	3	4
2	4	4	1	2	4	5	1	3
1	4	3	1	5	5	1	2	5
3	3	3	3	3	3	3	3	3
2	4	4	2	3	4	4	2	4
2	5	4	2	1	1	2	2	4
4	4	4	4	4	4	4	4	4
2	4	2	2	2	2	2	4	4
4	4	2	2	3	4	2	2	4
1	2	4	1	5	2	4	3	4
4	1	2	1	4	4	1	4	4
4	4	1	1	4	4	1	4	4
3	3	3	3	3	3	3	3	3
4	3	3	3	3	3	3	3	5
3	4	2	2	3	3	2	4	4
3	3	2	2	3	2	2	3	4

2	4	2	2	2	3	2	2	3
3	4	3	3	3	3	3	2	4
3	3	3	3	3	3	3	3	3
3	4	2	1	4	4	1	3	4
1	5	5	5	2	1	5	1	2
2	4	2	1	3	3	1	4	4
4	4	2	1	4	4	2	3	4
3	2	3	2	3	3	2	2	3
5	5	1	1	5	5	1	5	5
3	2	5	1	2	5	1	5	5
4	4	4	4	4	4	4	4	4
3	3	2	3	5	5	5	4	4
5	5	3	1	4	5	1	3	5
3	4	3	3	3	3	3	3	3
4	4	4	2	3	4	3	2	4
5	5	1	1	5	5	1	1	5
3	3	3	3	3	3	3	3	3
3	3	4	3	3	3	3	3	3
3	4	2	1	3	4	3	4	5
4	4	2	2	4	4	2	4	4
3	3	3	2	3	3	3	3	4
4	4	2	2	4	4	2	4	4
3	4	4	3	3	3	4	3	3
3	4	4	2	3	4	2	2	4
3	3	3	3	3	3	3	3	3
3	2	2	2	5	5	5	2	2
5	5	1	1	5	5	1	4	5
2	2	4	2	2	3	3	3	4
5	5	1	1	5	5	5	5	5
2	4	4	2	2	2	2	2	2
3	3	3	3	3	3	3	3	3
5	5	2	2	5	5	1	5	5
3	4	3	3	4	3	3	2	3
3	4	2	2	3	3	2	3	4
5	5	1	1	5	5	1	5	5
3	4	3	2	3	3	3	3	3
4	5	2	1	4	4	2	2	4
4	4	4	3	3	2	4	2	2
3	3	5	3	2	3	4	1	3
4	4	2	2	4	3	3	4	4
3	4	2	2	2	3	2	4	3
2	5	4	2	2	2	1	1	5
2	2	3	2	2	4	2	2	4
4	3	2	2	3	4	2	3	4
4	4	1	1	3	4	2	3	4
4	4	2	1	3	4	1	3	4
3	2	3	3	3	2	4	3	3
4	4	2	2	4	4	2	4	4
2	2	4	4	2	3	3	3	3
3	3	3	2	3	3	3	3	3
2	4	2	2	4	4	2	4	4
3	3	3	3	3	3	3	3	3
4	4	3	2	3	4	2	3	4
2	4	2	2	3	2	3	2	4

4	4	3	3	3	2	2	2	2
4	4	2	3	2	3	4	5	3
3	3	4	3	3	2	4	4	3
1	2	3	3	1	2	3	2	4
3	2	2	2	3	3	2	3	4
4	3	2	2	4	4	2	3	4
1	3	4	2	3	3	4	1	2
2	2	4	1	1	2	3	2	2
1	1	4	2	1	2	2	2	3
3	3	3	3	2	2	3	2	2
3	4	3	2	4	4	2	4	4
3	4	2	1	3	4	2	3	4
4	4	5	5	2	3	1	3	5
2	4	2	1	3	4	2	4	4
4	4	3	3	4	4	2	4	4
4	4	2	1	4	3	2	5	4
2	4	3	3	2	4	4	2	4
3	4	3	2	3	3	2	2	2
2	4	1	1	3	3	1	4	4
3	4	2	1	3	3	1	2	4
2	4	4	3	2	3	3	3	4
4	4	4	4	4	4	4	4	4
4	3	2	2	2	3	1	4	4
3	3	3	3	3	3	3	3	3
5	5	2	1	5	5	2	4	5
2	3	1	1	1	5	1	5	5
3	3	2	1	3	3	2	3	4
4	4	3	2	4	4	2	3	4
3	3	3	2	4	3	3	2	4
2	4	2	1	3	2	1	2	1
4	2	3	4	2	4	3	2	2
4	4	3	2	3	3	2	2	3
4	4	3	4	4	4	4	4	4
3	3	4	4	4	4	4	4	4
4	4	2	2	4	5	2	3	4
3	3	3	3	3	3	3	3	4
5	5	5	5	5	5	5	5	5
5	5	2	1	5	5	1	4	5
3	4	4	5	5	4	4	5	4
5	5	2	1	5	5	1	2	5
4	4	4	4	4	4	4	4	4
2	3	4	3	3	3	3	3	3
2	3	3	1	5	5	2	3	3
4	4	4	2	4	4	2	4	4
4	4	4	4	4	4	4	4	4
3	3	3	2	2	3	4	3	4
4	3	2	2	4	4	2	4	4
3	4	3	1	5	4	2	4	4
4	4	2	2	4	4	2	4	4
4	4	2	2	4	4	2	3	4
4	2	2	2	4	3	2	4	4
3	3	3	3	3	3	3	3	4
5	5	2	2	4	4	1	4	5
5	1	1	1	5	5	1	3	5

5	5	2	1	5	5	1	5	5
5	5	1	1	5	5	1	5	5
4	3	2	2	3	3	1	5	5
4	4	4	2	4	4	2	3	4
5	5	2	1	5	5	1	3	4
5	5	1	1	4	5	1	5	4
2	4	2	1	3	4	4	2	4
5	2	2	1	1	4	1	1	5
2	4	2	1	3	5	1	3	5
5	5	2	1	5	5	2	4	5
5	3	2	1	3	3	1	3	3
2	2	3	4	4	5	3	3	3
4	4	3	2	3	4	3	2	4
4	4	2	2	4	4	2	4	4
4	4	2	2	4	4	2	4	4
3	3	4	3	3	3	3	3	3
2	4	4	2	3	2	2	3	4
5	4	1	1	4	5	1	3	4
3	4	2	2	3	4	2	2	4
1	2	1	1	5	5	2	4	5
5	5	3	1	5	5	1	5	5
4	3	3	3	3	3	4	5	5
3	3	3	3	3	3	3	3	3
3	4	3	3	3	3	3	3	4
4	5	5	4	4	4	4	5	5
3	3	3	3	3	3	3	3	3
3	3	3	3	3	3	3	3	3
3	3	2	1	4	4	2	4	5
4	4	2	2	4	4	1	4	4
3	3	5	2	1	1	5	2	3
4	4	2	2	3	4	2	3	4
2	3	2	2	2	3	2	3	3
4	5	5	5	5	5	5	5	5
4	4	3	2	3	4	2	4	4
4	4	2	2	5	4	2	3	4
5	4	2	1	5	5	1	4	5
4	3	3	2	3	4	1	5	5
4	4	3	3	4	4	4	4	4
3	3	1	1	3	5	1	5	5
4	4	2	2	4	4	2	4	4
5	5	5	5	5	5	5	5	5
3	3	3	3	3	3	3	3	3
3	2	1	1	3	3	2	4	3
3	4	3	3	3	3	3	3	3
4	4	2	1	4	4	1	4	5
3	3	3	3	3	3	3	3	3
1	5	5	1	2	1	1	1	5
3	3	3	3	3	3	3	3	5
3	3	3	2	3	3	2	3	4
3	3	3	3	3	3	3	3	3
3	3	4	3	3	3	3	3	3
4	3	2	2	3	3	2	2	4
4	4	2	2	4	5	2	4	4
4	4	3	2	4	3	2	3	5

5	5	1	1	5	5	1	5	5
5	5	1	1	5	5	1	5	5
3	5	2	1	5	5	1	5	5
4	4	1	1	3	4	1	4	4
3	3	3	2	1	1	5	2	3
5	5	3	1	5	5	1	5	5
1	5	4	2	4	4	2	4	4
5	4	5	4	4	3	4	5	3
5	5	5	5	5	5	5	5	5
3	5	3	1	4	4	1	4	4
4	4	2	2	3	4	2	4	4
5	5	5	2	3	3	2	3	4
2	5	2	1	5	5	1	5	5
3	3	3	3	3	3	3	3	3
2	4	4	2	4	2	4	4	3
2	4	4	2	4	3	2	5	5
4	3	2	1	5	3	1	4	5
5	4	4	5	4	4	4	4	3
3	3	3	3	3	3	2	3	3
4	4	3	1	4	4	1	4	4
2	4	3	3	4	3	2	5	4
4	4	2	2	4	4	2	4	5
3	3	1	1	4	5	1	5	3
5	5	2	1	5	3	1	4	5
3	1	4	3	3	3	3	3	3
3	4	3	1	5	5	1	5	5
5	5	2	1	5	3	1	2	5
2	4	5	2	3	2	3	3	3
2	4	2	1	3	4	2	2	5
3	3	3	3	3	3	3	3	3
3	3	3	2	3	3	3	3	3
5	5	1	1	5	5	1	4	5
4	4	2	2	2	4	2	4	4
5	5	4	1	4	4	1	3	4
1	2	4	2	1	2	4	3	1
3	2	3	2	2	1	3	2	3
3	3	3	3	3	3	3	3	3
5	5	1	1	5	5	1	5	5
2	4	2	2	2	2	2	2	4
5	5	2	1	5	3	1	3	3
4	3	3	2	4	4	2	3	3
5	5	1	1	5	5	1	5	5
3	5	3	1	5	5	1	2	5
5	5	1	1	5	5	1	5	5
3	3	3	3	3	3	3	3	3
4	2	3	2	3	3	2	3	4
4	3	3	1	2	3	1	4	4
3	4	2	1	2	2	1	2	5
2	2	4	2	2	3	3	2	3
5	5	1	1	5	5	1	5	5
4	3	3	3	3	3	3	3	3
4	5	3	1	3	4	1	5	5
5	5	1	1	5	5	1	5	5
3	4	4	2	4	3	2	2	3

3	3	3	3	3	3	3	3	3
3	3	3	2	4	3	3	3	4
4	4	2	2	4	3	2	2	2
3	3	3	3	3	3	3	3	3
5	5	5	5	3	5	5	4	2
4	4	3	1	4	4	1	5	4
2	3	5	3	3	3	4	1	2
2	4	2	2	2	3	2	4	3
5	5	2	2	5	2	2	3	4
5	5	3	1	5	5	1	5	5
5	5	1	1	5	5	1	5	5
3	4	3	2	3	3	2	4	4
5	5	1	1	5	5	1	5	5
5	4	4	4	4	4	4	4	4
3	3	2	1	4	5	1	3	5
5	1	5	3	5	5	1	5	5
3	3	2	1	3	5	1	3	5
5	5	1	1	3	1	2	4	4
3	3	3	1	3	4	1	5	5
3	3	3	3	3	3	3	3	3
3	4	3	2	3	4	1	5	4
3	3	3	3	3	3	3	3	3
5	5	5	1	5	5	1	5	5
3	5	1	1	5	5	1	5	5
2	2	2	2	2	2	2	2	2
3	3	3	4	4	3	2	3	4
2	2	3	2	3	4	2	4	4
2	4	3	2	2	3	2	2	3
4	2	4	2	3	4	2	4	4
3	3	3	2	3	3	2	2	4
2	2	2	2	4	2	2	2	4
3	3	3	3	3	3	3	3	3
4	4	2	2	4	4	2	3	4
3	3	3	3	3	3	3	3	3
3	3	3	3	3	3	3	3	3
4	4	4	2	2	2	2	2	4
2	3	4	2	3	3	3	2	4
2	3	1	3	2	4	3	3	4
3	3	3	3	3	3	3	3	3
3	3	3	3	3	3	3	3	3
2	2	5	3	2	2	5	2	2
4	3	3	2	4	4	3	3	3
4	4	3	2	4	4	2	3	4
2	3	3	3	3	3	4	2	3
2	4	4	2	3	4	3	2	3
5	5	1	1	5	5	1	5	5
3	3	3	3	3	3	3	3	3
4	4	2	2	4	4	2	4	4
4	4	3	2	4	4	3	4	4
2	4	2	1	3	3	2	3	4
4	3	4	1	3	3	2	3	3
4	2	2	2	4	2	2	4	4
3	3	3	3	3	3	3	3	3
3	4	3	1	3	3	3	3	4

3	4	4	2	3	4	2	3	4
2	4	4	2	3	3	3	2	2
2	2	4	2	3	3	2	2	2
4	4	2	2	4	4	2	3	4
2	2	4	3	2	2	3	2	2
1	1	5	1	1	1	1	1	1
4	2	4	2	1	2	2	2	3
3	3	3	3	3	3	3	3	3
4	5	4	1	5	4	2	3	5
5	5	2	2	4	4	2	4	4
4	4	2	2	2	2	2	2	4
2	1	1	1	1	1	1	1	1
5	3	2	2	2	3	2	4	3
2	2	4	2	3	2	3	2	2
4	4	2	1	3	5	1	2	5
1	1	3	2	2	3	3	3	2
3	4	3	2	4	4	2	4	4
2	4	1	3	4	4	3	3	4
4	4	2	2	3	4	2	3	4
2	1	5	4	1	2	4	3	1
5	5	3	4	5	5	5	4	4
3	3	3	3	3	3	3	3	3
3	3	3	2	2	3	3	3	3
4	4	4	4	4	4	4	4	4
2	4	4	4	4	4	2	4	4
3	4	3	4	4	5	4	3	4
3	3	3	3	3	3	3	3	3
1	1	5	2	1	1	5	1	1
2	4	3	2	2	2	4	2	3
4	4	2	2	4	3	2	2	4
3	2	1	1	3	3	1	1	4
3	4	3	2	3	3	2	4	4
3	3	3	3	3	3	3	3	3
5	5	5	1	5	4	3	3	3
2	2	2	2	2	2	2	2	2
4	4	2	2	4	3	2	4	4
3	4	3	2	3	4	3	4	4
4	4	4	4	4	4	4	4	4
2	4	2	2	2	4	2	4	4
2	3	4	3	2	3	3	2	3
5	5	2	1	5	5	1	1	3
2	4	3	2	3	3	2	3	4
4	5	5	2	5	5	1	5	4
3	5	3	2	2	4	3	2	3
3	4	4	2	3	3	4	4	4
2	2	4	4	2	3	3	2	4
3	4	2	2	3	4	2	3	4
3	3	3	3	3	3	3	3	3
2	4	5	2	2	3	2	2	2
3	4	2	2	2	4	2	4	4
4	4	2	2	4	4	2	3	4
3	4	2	1	4	4	1	4	4
3	4	3	1	3	3	1	4	4
2	5	2	1	4	4	2	4	4

3	3	3	2	3	2	2	3	3
4	4	4	2	4	4	2	3	4
3	3	4	2	3	3	3	3	3
3	3	3	2	3	4	3	4	4
4	4	4	3	3	2	1	1	1
3	3	3	3	3	4	2	3	3
2	1	1	2	4	3	1	2	2
4	4	1	1	4	4	1	4	4
5	1	1	1	4	4	1	2	2
4	4	1	1	4	4	1	3	4
4	4	1	1	5	5	1	3	5
4	5	1	1	4	4	1	2	4
3	4	2	2	4	4	2	3	4
3	3	3	3	3	3	3	3	3
5	5	2	1	5	5	1	3	5
4	4	2	2	4	2	2	2	4
5	5	1	1	5	4	1	3	5
2	2	2	2	2	3	3	2	3
3	3	2	1	3	2	3	2	3
2	2	4	3	2	2	3	2	3
3	5	2	1	4	2	1	3	4
3	3	2	1	3	3	2	3	3
5	5	1	1	5	5	1	5	5
5	5	1	1	5	5	1	5	5
5	5	1	1	4	5	1	5	5
2	2	4	4	4	2	2	2	2
4	4	2	2	4	4	2	4	4
5	5	5	1	5	5	1	5	5
4	3	2	3	3	4	3	3	4
5	5	1	1	5	5	1	5	5
2	3	1	2	3	3	1	3	3
4	4	2	2	3	4	2	3	4
4	2	5	3	3	3	3	3	4
5	5	1	2	5	5	1	3	5
3	3	4	2	3	3	4	1	3
4	4	2	1	4	3	2	2	4
3	4	4	2	3	3	3	2	4
1	1	5	4	1	1	5	1	1
3	3	3	3	3	3	3	3	3
3	4	4	1	3	3	2	3	5
5	5	2	1	4	4	1	4	5
3	3	3	2	3	4	2	4	4
3	3	3	3	3	3	3	3	3
4	3	2	2	3	3	2	4	4
2	3	4	2	2	3	3	3	3
2	3	2	1	2	3	2	3	3
2	4	2	2	3	3	2	2	5
3	4	3	3	3	3	2	4	3
4	4	2	1	4	4	1	4	4
3	4	3	2	3	4	2	2	5
4	4	3	2	4	4	1	4	4
5	5	1	1	5	5	1	5	5
1	1	2	2	2	1	3	2	3
4	5	1	1	4	4	2	2	4

1	4	3	2	1	4	3	2	4
2	4	2	1	4	5	2	5	5
1	3	2	1	3	1	1	1	4
1	1	5	5	1	1	5	1	1
3	4	2	2	3	3	3	3	3
2	2	4	2	3	2	3	2	3
3	2	3	2	3	3	3	3	4
4	1	4	2	2	4	2	4	4
4	3	2	2	3	3	2	4	4
2	4	3	2	2	3	4	2	4
4	3	3	2	3	3	2	3	4
2	1	3	2	1	3	2	3	3
4	4	3	1	4	3	2	2	3
5	5	2	1	4	5	1	5	5
5	5	1	1	4	5	1	5	5
3	3	3	3	3	3	3	3	3
3	4	4	2	3	4	3	4	3
5	5	1	1	4	4	1	4	4
3	3	3	3	3	3	3	3	3
3	4	2	2	3	4	2	4	4
3	4	2	2	3	4	2	2	3
2	4	4	3	2	2	3	2	2
5	5	5	5	5	5	4	4	4
3	3	3	3	3	3	3	3	3
2	4	4	2	3	4	2	3	4
3	3	3	3	3	3	3	3	3
3	3	3	3	3	3	3	3	3
5	5	1	1	4	4	1	3	4
3	3	3	3	3	3	3	3	3
4	3	3	2	3	3	3	3	3
3	3	3	3	3	3	3	3	3
2	2	4	3	2	3	3	2	2
4	4	3	2	4	4	2	3	4
3	3	2	2	3	4	2	3	3
3	4	4	2	2	3	2	3	3
4	4	2	2	4	4	2	4	4
4	5	2	2	5	5	1	5	5
3	3	3	3	3	3	3	3	3
3	3	3	2	2	3	3	3	3
3	3	3	3	3	3	3	3	3
4	4	2	2	3	3	2	3	4
2	4	2	1	4	4	1	2	3
3	4	3	2	3	3	2	3	3
5	2	2	2	2	2	2	4	4
3	3	3	3	3	3	3	3	3
2	2	5	2	4	2	4	2	4
4	5	1	1	5	4	2	2	5
3	4	2	1	4	5	2	4	4
5	4	2	2	4	4	2	2	4
4	4	2	1	4	5	1	2	4
3	3	2	1	3	4	1	3	3
4	4	3	1	4	4	2	2	4
3	5	1	1	5	5	2	4	5
2	4	4	1	4	4	2	2	4

4	4	3	2	3	4	2	3	4
4	4	2	2	4	4	2	4	4
4	4	3	2	3	4	3	3	4
5	5	1	1	5	5	1	2	5
5	4	1	1	4	5	1	4	5
2	4	4	1	4	4	2	2	4

C69	C70	Excise at	D1	D2	D3	D4	D5	D6
4	5	337	1	1	1	1	2	1
1	1	207	1	1	2	1	4	3
5	5	345	2	2	3	2	2	2
5	4	321	2	3	1	2	2	2
1	3	212	4	1	4	1	4	1
4	4	339	1	2	2	2	1	2
5	4	309	3	3	2	3	3	3
5	2	228	4	3	3	3	1	3
1	1	217	2	3	1	1	1	1
3	5	302	1	1	1	1	2	2
2	2	287	3	1	1	1	2	3
2	2	230	2	3	1	1	2	1
1	1	323	1	1	1	1	4	1
4	5	332	4	4	4	4	3	4
5	5	282	4	3	1	4	4	4
5	5	349	1	1	1	1	1	1
1	2	208	1	2	1	2	2	2
5	5	333	4	4	4	4	4	4
2	3	233	2	2	1	2	4	2
2	5	224	2	1	1	1	3	2
2	1	301	2	2	2	2	3	2
5	5	321	2	2	2	3	3	3
1	1	305	1	1	1	1	1	1
1	1	287	3	2	1	1	2	1
3	3	244	2	1	2	1	2	2
2	2	207	2	2	3	2	2	3
3	3	257	2	2	2	2	2	2
1	2	273	3	2	2	2	2	2
1	1	232	1	3	1	1	1	1
2	2	320	1	1	1	1	1	1
1	1	324	2	2	1	1	1	1
1	1	324	1	1	1	2	1	1
1	2	268	1	4	4	4	3	1
1	4	257	3	3	3	3	3	3
1	4	239	2	3	2	1	3	1
3	3	301	1	1	1	1	1	1
1	2	258	4	4	4	4	4	4
5	5	320	4	4	4	4	4	4
1	2	274	1	1	1	1	1	1
1	1	302	4	4	4	4	1	1
1	1	279	1	1	1	1	1	1
4	2	320	1	1	1	1	1	1
1	1	235	3	3	3	3	3	3
1	1	266	1	1	1	1	1	1
3	4	257	1	1	1	4	2	2
4	4	324	3	3	3	3	3	3
4	4	226	1	1	1	1	1	1
4	5	263	1	4	4	4	4	4
2	2	322	1	2	2	2	1	2
1	1	266	3	3	2	3	3	2
3	3	258	4	4	4	4	4	4
1	1	240	2	2	1	1	1	1
1	2	339	1	1	1	1	1	1

3	3	258	3	2	2	2	3	3
2	2	287	2	3	2	1	2	2
3	3	329	4	3	3	4	3	4
5	5	334	3	3	2	2	2	2
3	5	242	2	4	4	4	4	2
1	1	252	1	1	1	1	1	1
1	1	256	1	1	1	1	1	1
5	5	268	1	1	1	1	1	1
2	2	323	2	2	2	2	4	2
1	3	326	2	1	1	2	1	2
2	2	283	3	1	2	1	2	2
1	1	286	2	2	1	3	2	1
1	1	312	1	1	1	1	1	1
5	5	272	1	1	1	1	1	1
2	2	264	1	1	1	1	3	2
1	1	248	1	1	1	1	1	1
1	1	276	1	1	1	1	1	1
3	3	282	1	1	1	1	1	1
1	1	241	1	2	1	1	2	1
2	2	215	2	2	2	2	2	2
2	2	307	2	3	2	1	3	2
1	1	179	4	2	1	1	2	2
3	3	279	2	2	3	1	2	1
3	2	223	1	3	2	2	2	1
3	3	216	1	1	1	1	1	1
4	4	304	3	2	2	1	4	2
2	1	183	2	1	1	1	2	2
3	3	229	3	2	2	3	2	2
3	3	306	3	2	2	1	4	4
3	3	198	2	1	1	1	1	1
4	3	260	3	2	2	3	4	3
2	3	319	1	3	4	2	2	4
1	1	302	1	2	3	3	3	4
1	2	229	4	4	2	3	2	2
2	2	218	3	3	2	1	2	2
1	1	305	1	1	1	1	1	1
1	1	203	1	2	1	1	2	1
1	3	270	2	2	2	3	2	3
3	4	209	1	1	1	1	1	1
1	1	318	1	1	1	1	1	1
3	3	237	3	3	3	3	3	3
2	2	223	2	2	3	2	3	2
2	3	336	4	4	4	4	4	4
1	1	238	2	2	1	1	1	1
1	2	330	2	3	2	2	3	4
3	2	284	2	2	2	2	2	2
3	3	281	3	4	2	2	2	2
2	2	293	1	1	1	1	1	1
1	1	287	1	1	1	1	1	1
2	2	197	2	1	1	1	2	1
2	4	218	2	3	2	2	1	1
1	1	144	1	1	1	2	1	1
2	2	277	1	1	1	1	1	1
2	3	307	2	1	1	1	2	1

3	2	217	4	4	2	4	4	4
5	5	248	3	3	2	3	3	1
1	2	226	2	4	2	4	4	2
1	1	268	1	4	1	1	4	1
1	1	310	2	2	1	2	2	2
1	1	211	2	3	1	2	2	1
4	4	214	3	2	1	2	4	2
2	2	293	1	1	1	1	3	1
1	1	191	1	1	1	1	3	1
3	3	178	2	2	1	2	2	1
3	3	271	3	4	3	2	3	3
1	1	242	1	1	1	1	1	1
1	1	272	1	2	3	2	3	3
2	2	272	3	2	2	1	2	1
3	2	250	2	2	1	2	2	1
2	2	257	3	3	1	1	3	3
1	1	255	3	3	1	2	2	1
2	2	230	1	1	1	1	4	1
1	1	212	2	4	2	1	2	2
4	2	215	3	3	3	3	3	3
2	1	285	1	2	2	1	1	2
1	1	281	1	1	1	1	1	1
3	3	298	4	4	4	4	4	4
3	3	270	3	2	1	2	2	3
2	2	228	1	2	2	2	3	2
2	3	317	3	2	3	1	2	1
2	3	274	1	2	3	1	2	2
3	4	292	3	2	2	2	4	2
1	3	208	1	1	1	1	2	2
1	1	211	3	1	1	1	3	2
1	2	255	2	1	3	2	4	1
2	2	300	2	1	2	1	1	1
3	4	264	3	3	2	1	2	3
5	5	274	3	2	1	1	3	2
1	3	253	1	1	1	1	1	1
2	1	231	2	1	1	1	2	1
2	5	195	1	1	1	1	1	1
2	2	156	1	1	1	1	1	1
1	2	206	4	2	1	1	4	2
5	3	180	2	2	2	2	2	2
1	1	346	3	3	2	1	3	2
2	5	289	3	3	2	4	4	3
2	3	165	2	3	2	3	3	1
2	3	300	1	2	2	1	2	2
1	1	261	1	1	1	1	1	1
1	3	220	2	2	1	1	3	2
3	3	300	3	4	1	3	4	1
1	2	262	1	1	2	3	3	1
2	2	226	2	2	2	1	2	4
2	2	254	1	1	1	1	4	3
2	1	279	1	2	1	1	2	2
5	3	242	4	2	1	2	4	3
3	3	233	2	3	2	4	3	2
2	2	216	1	1	1	1	1	1

1	1	278	2	1	1	1	2	1
1	1	259	1	2	1	1	1	2
1	1	256	1	1	1	1	2	1
3	2	268	2	1	2	1	2	2
1	1	162	2	1	2	2	2	1
2	2	243	2	3	2	2	2	2
2	2	261	1	1	1	2	1	1
2	2	319	1	1	1	1	1	2
4	4	227	4	4	4	4	4	4
2	1	322	3	2	3	2	3	2
1	1	224	1	1	1	1	1	2
1	1	257	2	1	2	2	2	3
1	1	290	2	2	2	2	2	3
1	1	253	1	1	1	1	1	1
3	3	101	2	2	1	1	3	2
1	2	320	3	1	1	1	2	1
1	1	242	2	2	1	1	2	1
2	1	317	1	1	3	3	2	2
3	3	254	4	4	4	2	1	1
1	1	307	2	3	1	1	3	1
1	4	263	1	1	1	1	1	1
1	1	224	2	3	2	1	2	1
1	2	238	1	1	2	1	3	2
1	1	121	1	3	2	1	1	2
3	5	334	2	2	1	1	4	1
2	4	266	1	2	2	1	2	1
1	2	232	1	1	2	2	1	1
4	3	326	2	4	4	2	4	3
2	3	302	2	1	4	2	2	2
5	4	262	1	1	1	1	2	1
2	2	226	2	1	1	1	1	1
1	1	284	1	1	2	2	1	1
1	1	329	3	3	1	1	1	1
1	1	305	1	3	3	3	2	2
1	1	258	3	2	2	1	2	1
1	1	114	3	3	2	1	1	1
3	3	179	1	1	4	1	2	1
1	1	254	2	2	2	2	3	4
1	1	258	1	1	1	1	2	1
1	1	268	3	3	3	3	3	3
1	1	230	2	4	2	2	2	2
2	2	245	3	2	2	2	2	2
1	3	210	3	4	4	4	4	4
1	1	330	1	2	1	1	2	2
2	2	314	2	2	1	2	1	3
2	2	324	2	2	2	1	2	2
3	3	299	1	4	1	1	1	1
1	1	243	1	1	1	1	1	1
1	1	216	2	1	1	2	2	1
1	1	268	1	1	1	2	1	2
1	1	229	2	2	1	2	4	3
2	4	131	1	1	1	1	1	1
3	3	293	1	1	1	1	1	1
1	1	235	3	4	3	2	4	4

3	3	266	1	2	1	2	3	2
4	2	198	4	3	1	4	4	4
1	1	137	3	2	2	1	2	2
2	2	280	2	3	1	1	2	1
5	1	237	2	2	2	2	4	2
2	2	250	1	1	1	1	1	1
4	4	258	4	4	4	4	4	4
1	3	214	3	3	2	1	1	2
2	2	162	2	2	2	2	1	1
2	2	263	4	4	3	3	3	2
1	1	221	1	3	3	3	2	1
1	1	195	2	4	1	1	4	1
1	1	120	3	3	2	1	1	2
2	2	224	1	1	1	1	2	1
3	3	225	1	1	3	1	3	3
1	1	280	2	4	4	1	2	2
3	3	256	2	2	2	2	2	2
2	3	251	1	1	1	1	1	1
3	3	322	2	2	2	1	2	2
1	1	304	4	2	4	1	4	3
2	2	222	3	1	2	1	2	2
3	3	222	1	4	2	2	3	3
1	1	220	1	1	1	1	1	1
1	1	311	2	1	2	1	1	2
3	3	160	2	2	2	2	3	2
3	3	282	4	3	1	3	4	1
2	3	214	1	1	1	1	2	2
1	1	119	1	1	1	1	1	1
5	1	180	2	4	3	1	4	2
1	1	174	1	1	2	1	2	1
2	2	266	2	2	2	2	2	2
4	5	251	2	4	4	4	4	2
3	3	252	3	3	3	4	3	3
2	2	269	3	2	2	1	4	2
1	1	134	1	1	1	1	2	2
2	1	279	1	2	2	1	2	1
1	2	166	1	2	1	1	1	1
1	1	238	1	3	1	1	4	4
4	4	237	2	2	1	1	1	1
1	1	280	1	1	1	1	1	1
2	2	208	3	1	1	1	1	1
3	1	219	2	2	2	1	2	2
2	1	245	3	3	2	3	4	4
1	1	244	1	1	1	1	1	1
3	3	244	4	4	4	4	4	4
3	3	180	1	1	1	1	1	1
2	2	234	1	1	1	1	2	1
2	1	233	2	2	3	2	1	1
1	2	310	3	3	2	1	3	3
4	4	313	1	3	3	2	3	2
4	3	252	1	3	3	2	3	2
1	1	310	2	4	1	2	3	1
3	3	265	1	1	1	1	1	1
2	2	329	2	3	1	2	2	1

1	1	221	2	2	1	2	1	1
1	1	112	2	1	1	1	2	2
1	1	315	2	2	1	1	3	1
2	2	304	2	2	2	2	2	2
2	2	262	4	4	4	4	4	4
1	1	213	3	3	2	1	2	2
1	1	154	3	3	3	3	3	3
1	1	257	3	1	1	1	1	1
3	4	274	2	2	1	1	2	2
3	3	209	2	2	2	1	1	1
1	2	295	2	2	2	1	1	1
2	1	274	3	3	2	1	3	3
2	2	311	1	2	2	1	2	1
1	1	112	1	1	1	1	1	1
1	1	292	1	1	1	1	1	1
2	2	300	2	1	1	1	1	1
3	3	207	4	4	3	3	4	4
2	2	252	2	2	1	1	2	1
1	1	254	1	1	1	1	1	1
2	3	262	3	3	2	1	2	3
4	4	224	1	2	1	1	1	4
1	2	259	1	1	1	1	1	1
4	4	154	2	3	1	1	2	2
2	2	271	1	1	1	1	1	3
5	5	239	3	3	3	3	3	3
5	5	254	3	2	1	3	4	3
1	1	311	2	2	4	3	4	2
2	2	300	2	3	3	2	3	2
1	5	253	4	1	4	3	4	4
1	1	279	1	1	1	1	1	1
1	1	262	1	1	1	2	2	3
3	3	219	1	1	1	1	1	1
3	3	246	2	1	1	1	2	2
5	5	269	2	2	2	1	2	2
3	3	279	3	2	2	2	2	2
1	3	207	4	1	3	3	3	3
5	4	222	1	1	1	1	1	1
2	1	264	1	1	1	1	1	2
1	1	207	1	1	1	1	1	1
3	3	289	3	2	2	3	2	3
2	2	257	2	3	1	2	3	3
3	3	235	2	2	2	2	2	2
1	3	186	1	1	1	2	2	2
2	2	251	2	2	1	1	1	1
3	3	273	3	3	2	2	3	2
3	4	265	2	2	1	1	3	1
4	2	242	2	2	2	2	2	2
2	2	206	1	1	1	1	1	1
1	1	294	1	1	1	1	2	1
1	2	250	3	3	3	2	2	3
3	4	170	2	2	1	1	3	3
1	1	287	1	1	1	1	1	1
3	2	263	1	1	1	1	1	1
5	4	233	3	2	2	3	4	2

1	1	282	1	2	1	1	3	2
2	2	243	2	2	1	1	2	1
3	3	187	2	1	1	1	3	2
1	2	281	1	1	1	1	1	1
3	1	224	4	4	4	4	4	4
4	4	244	3	2	2	2	2	2
1	2	229	1	1	2	1	2	2
2	2	239	2	3	3	2	4	4
2	2	273	2	2	1	1	2	2
3	3	252	4	4	4	4	4	4
1	1	256	2	2	1	2	2	1
2	2	255	2	1	2	1	2	2
2	1	234	3	3	3	3	3	3
2	2	242	3	3	3	2	2	2
2	2	275	3	3	2	2	2	2
4	4	238	4	4	4	4	4	4
1	4	215	1	1	1	1	2	1
2	2	252	2	3	2	1	2	1
2	3	223	3	3	3	3	3	3
3	3	291	2	2	2	2	3	2
1	2	255	2	2	1	1	2	2
3	3	260	3	2	2	2	3	2
1	1	307	1	2	1	1	1	1
1	1	197	1	2	1	2	2	1
2	2	258	1	1	1	1	1	1
1	1	236	2	1	1	1	3	1
1	2	270	2	2	2	2	2	2
2	2	249	2	1	2	1	3	2
1	2	248	2	2	1	2	3	2
2	3	237	2	1	2	2	2	2
2	2	278	1	2	1	1	2	1
1	1	217	2	2	2	2	2	2
2	2	199	3	3	2	2	3	2
2	2	222	4	1	1	3	2	3
2	2	251	2	1	1	1	1	1
2	3	264	3	1	1	1	1	3
2	1	206	3	1	1	2	4	2
3	3	228	3	2	2	2	2	2
2	2	197	2	2	2	2	2	1
1	1	221	4	4	4	4	4	4
2	4	239	2	1	1	2	3	1
3	3	256	2	3	3	2	2	3
3	3	229	2	3	2	2	3	2
2	2	291	2	2	1	1	1	1
2	2	291	3	2	4	2	2	2
3	2	255	2	2	1	1	2	2
1	1	314	1	3	1	2	2	1
2	2	255	2	2	2	1	1	4
1	2	242	1	1	1	1	1	1
2	1	229	4	4	4	3	3	3
1	1	258	1	1	1	4	2	2
2	1	317	2	3	2	3	3	3
5	4	252	2	3	2	2	2	3
2	2	195	1	2	2	2	2	2

2	2	208	1	1	1	1	2	1
1	1	327	1	1	1	1	1	1
2	2	264	3	2	2	3	2	3
2	4	217	2	2	3	2	2	2
2	3	254	3	2	1	2	2	2
1	1	182	1	1	1	1	1	1
1	1	257	1	1	1	1	2	2
2	2	246	1	1	1	1	2	1
2	2	253	1	2	2	4	2	3
3	3	234	1	1	1	1	3	1
1	2	254	1	1	1	1	2	1
1	1	234	4	3	3	4	4	3
2	2	223	3	3	3	2	2	2
1	1	216	1	2	1	2	2	2
3	3	302	1	2	2	1	2	2
3	2	287	1	4	1	1	1	1
2	2	252	1	2	1	1	2	1
3	3	310	1	1	3	1	2	1
5	5	276	1	4	4	1	1	1
3	2	310	4	4	4	2	3	1
2	2	153	3	3	2	2	3	2
1	1	214	1	1	1	1	1	1
2	1	304	3	2	2	2	2	2
1	1	251	2	2	1	1	1	2
1	1	234	2	1	2	1	4	4
2	2	225	1	2	2	2	1	2
2	1	293	2	1	2	1	2	2
1	1	220	2	1	1	1	4	1
3	2	301	1	2	2	2	4	2
2	1	113	4	4	4	4	4	4
1	2	205	1	2	1	1	2	1
3	3	199	2	2	2	2	2	2
2	2	206	2	2	1	2	2	2
4	5	240	2	1	1	1	2	1
2	3	266	2	1	1	1	2	1
1	1	200	1	1	1	1	1	1
4	4	282	1	1	2	1	4	4
1	1	226	2	2	3	2	2	2
2	2	194	2	2	2	2	2	2
1	3	213	3	2	1	1	3	2
1	1	234	1	3	1	1	1	1
3	3	253	1	1	1	2	1	1
1	4	259	1	3	1	1	1	3
2	2	180	1	1	1	1	1	1
4	4	289	3	3	3	3	3	3
2	1	281	3	1	1	1	3	3
2	2	229	1	1	1	1	1	1
2	3	219	4	2	1	3	2	2
1	1	215	4	4	4	4	4	4
1	1	247	2	1	1	1	1	1
3	3	229	4	2	1	1	3	2
4	4	227	2	4	1	1	2	1
2	2	232	2	1	1	1	1	1
1	2	261	1	1	1	1	2	2

2	3	158	3	1	1	3	1	1
2	3	257	4	4	4	1	3	2
3	3	287	3	3	3	3	3	3
2	2	212	3	2	1	1	3	2
3	3	324	4	4	1	1	4	4
1	1	292	1	1	1	1	1	1
1	2	247	2	1	1	1	2	2
2	2	256	2	4	1	1	2	1
1	1	298	1	1	1	1	1	1
1	1	223	4	4	4	4	4	4
4	4	243	1	1	1	1	1	1
5	4	331	1	1	1	1	1	1
1	1	237	2	2	2	2	2	2
2	2	218	3	1	1	1	3	3
4	2	259	3	2	2	2	2	2
1	1	248	1	1	1	1	1	1
3	3	235	3	3	1	1	1	1
3	3	262	1	2	1	2	1	2
1	2	215	2	2	2	2	2	2
1	1	230	2	1	3	1	2	1
2	2	223	3	3	2	2	2	2
2	2	179	1	1	1	1	1	1
2	4	305	2	2	1	2	2	1
2	3	212	1	3	1	2	2	1
3	2	274	1	2	2	2	3	2
4	2	180	1	1	4	4	2	2
1	1	227	1	1	1	1	1	1
2	3	331	2	2	1	1	1	1
5	5	213	4	4	4	4	4	4
4	4	253	1	1	1	1	2	1
3	3	326	2	2	2	2	2	2
1	1	230	4	1	1	1	1	1
2	3	296	3	3	2	2	3	3
2	2	237	1	1	1	1	1	1
1	1	192	1	1	1	1	1	1
2	2	263	2	2	2	1	2	1
1	1	231	1	1	1	1	1	1
2	2	261	3	2	3	2	2	2
4	4	198	3	3	3	3	3	3
2	2	254	2	1	1	2	1	3
2	2	301	2	2	2	1	3	3
1	1	277	3	4	1	1	2	2
3	4	218	1	1	1	1	1	3
2	2	266	1	1	1	1	2	2
1	3	171	2	2	1	1	1	1
4	2	220	2	1	1	1	2	2
2	3	265	2	3	3	2	4	4
2	2	209	2	3	1	1	1	1
3	3	246	3	2	2	2	1	2
2	3	231	2	2	2	2	2	2
2	2	231	2	2	2	2	2	2
3	3	240	1	3	1	1	4	4
2	2	221	3	3	1	2	3	3
1	4	185	1	4	2	3	2	2

3	3	239	1	1	2	2	2	2
4	3	266	1	1	4	4	4	4
2	3	174	1	1	1	1	1	1
3	2	174	4	4	3	2	3	2
2	2	179	2	2	2	2	2	2
2	2	195	2	2	1	2	1	1
3	3	262	1	2	3	1	3	1
4	4	271	2	3	1	2	2	1
3	3	209	3	1	2	1	2	1
2	2	257	2	2	2	2	1	1
1	3	269	3	3	2	2	3	3
1	1	270	1	2	1	1	1	1
3	3	203	1	1	1	2	3	2
1	1	220	1	1	1	1	1	1
1	2	283	1	1	1	1	2	2
1	1	254	3	2	2	2	3	3
2	2	198	2	2	1	1	3	1
2	2	234	2	2	2	2	2	2
1	1	208	2	1	1	1	1	1
1	1	219	2	2	1	4	4	1
2	2	324	3	2	2	2	2	2
4	4	225	3	2	2	2	3	2
5	4	252	3	3	3	3	3	3
3	3	260	2	2	2	4	3	2
1	1	253	2	2	1	2	2	2
5	1	230	2	1	1	1	1	2
3	3	236	1	3	2	2	2	2
2	3	262	2	3	1	2	3	3
1	2	215	3	3	3	2	3	3
1	1	239	3	3	2	2	2	3
2	2	271	4	4	4	4	4	4
3	3	202	2	2	2	2	2	2
4	4	280	2	2	2	2	2	2
4	4	315	3	3	3	3	3	3
2	2	225	2	2	1	1	1	1
3	1	319	4	4	4	4	4	4
5	5	237	4	4	4	4	4	4
1	1	213	2	2	2	2	2	2
5	5	262	3	3	3	3	3	3
1	1	258	1	1	1	1	1	1
4	4	234	3	3	3	3	3	3
3	3	201	2	2	2	3	4	2
1	1	268	1	1	1	1	1	1
2	2	269	1	1	1	1	1	1
4	4	284	2	2	2	2	2	2
3	3	281	3	2	1	2	3	1
2	1	258	3	2	1	1	1	1
2	2	228	3	3	1	2	2	2
4	2	307	2	2	2	1	2	2
2	2	290	4	4	4	4	4	4
2	2	300	2	2	2	2	2	2
3	3	325	2	1	1	2	1	2
1	1	270	1	1	1	1	1	1
1	1	237	2	2	2	2	1	1

1	1	292	1	1	1	1	1	1
1	1	325	1	1	4	1	1	4
1	1	255	1	1	1	1	1	1
4	2	293	1	2	2	2	1	1
1	1	251	2	1	3	2	1	3
1	1	306	1	1	1	1	1	1
2	1	280	1	1	1	1	1	1
1	1	195	1	3	1	1	1	1
1	1	252	2	1	1	1	2	1
1	1	263	2	2	2	2	3	2
1	1	253	2	2	2	2	2	1
5	4	192	3	3	2	3	2	2
2	2	234	3	2	1	1	1	1
2	2	284	2	2	2	2	3	2
2	2	254	2	2	2	1	1	2
3	3	288	3	3	2	1	4	2
2	3	319	2	2	2	2	2	2
1	3	188	3	2	2	1	2	1
2	2	217	2	1	1	1	2	1
1	1	249	2	3	4	2	1	1
1	1	262	2	1	1	1	1	2
4	3	210	1	1	1	1	1	1
3	3	221	3	3	3	3	3	3
3	3	270	3	3	3	3	4	3
5	5	280	2	2	3	2	4	1
3	3	137	2	4	2	2	3	1
3	3	264	3	2	2	2	2	2
1	1	243	1	1	1	2	2	2
1	1	308	4	4	4	2	2	1
3	4	267	4	4	4	3	4	4
2	2	285	1	1	1	1	1	1
1	2	328	1	1	1	1	2	1
5	1	284	4	4	4	4	4	4
2	2	253	2	2	1	2	2	1
2	2	300	1	1	1	1	1	1
1	1	263	2	2	2	2	3	2
1	1	256	2	1	1	1	1	1
4	4	247	2	2	2	2	2	2
1	1	276	1	1	1	1	1	1
2	2	244	2	2	2	1	2	2
5	5	302	4	4	4	4	4	4
3	3	226	1	1	1	1	1	1
1	2	216	2	1	1	1	1	1
3	3	239	1	2	2	1	2	1
1	1	241	1	1	1	1	2	2
3	3	255	4	4	4	4	4	4
1	1	203	3	4	1	3	4	4
1	1	249	3	3	3	1	2	2
1	3	280	2	2	1	2	2	1
3	3	305	1	3	2	3	2	2
3	4	322	4	4	3	2	4	4
2	2	308	1	1	1	1	2	1
2	2	269	1	1	1	1	1	1
1	1	273	1	2	1	2	1	1

1	5	205	1	4	1	1	4	1
1	1	309	4	4	4	4	4	3
1	1	227	2	2	2	2	2	2
1	1	292	1	1	1	1	3	2
3	3	264	4	2	2	1	4	4
1	1	286	1	3	1	1	2	1
2	1	259	2	1	2	1	3	1
5	4	223	3	3	3	3	3	3
5	5	313	4	4	4	4	4	4
1	1	214	4	1	1	1	1	1
2	3	247	3	2	1	1	1	1
4	3	272	3	3	3	3	3	3
1	1	272	1	1	1	1	1	1
3	3	205	1	1	1	1	1	1
1	2	190	2	2	1	1	1	1
1	1	261	3	2	2	3	2	2
1	1	243	3	4	1	1	2	1
3	3	274	1	1	1	1	1	1
4	3	298	1	2	2	1	3	2
1	1	308	3	2	1	2	2	2
2	3	207	2	3	3	2	2	3
2	2	307	1	1	1	1	1	1
1	1	300	1	1	1	1	1	1
1	1	222	2	3	1	1	2	2
3	3	269	1	1	1	1	1	1
1	1	241	1	2	2	1	1	4
1	1	212	1	2	1	1	1	1
2	3	321	2	2	1	1	1	1
1	1	264	1	1	1	1	1	2
3	3	310	3	2	1	2	2	2
3	3	196	2	1	2	1	4	4
1	1	199	2	2	3	1	4	2
2	2	217	1	1	1	1	1	1
1	1	316	2	2	1	1	1	2
4	3	233	3	2	1	2	3	3
2	2	290	3	2	3	2	3	1
3	3	255	2	3	1	1	2	2
1	1	308	1	1	1	1	1	1
2	2	287	4	3	1	1	1	1
1	1	332	2	3	2	1	2	2
2	2	213	1	1	1	1	1	2
1	1	253	1	1	1	1	1	1
2	2	281	1	1	1	1	4	1
1	1	258	2	2	1	1	1	2
3	3	316	1	2	1	1	1	1
2	2	203	1	4	1	1	1	1
1	1	320	1	4	1	1	4	1
3	1	221	1	2	1	2	2	2
2	3	307	3	3	3	2	3	2
1	1	334	1	2	2	4	2	3
3	2	239	3	3	3	3	3	3
1	1	210	1	4	1	1	1	1
1	1	272	1	1	1	1	1	1
2	2	280	3	3	1	2	4	4

3	3	210	1	3	3	3	3	3
1	1	246	1	3	1	2	2	1
1	2	302	1	2	1	1	1	1
3	3	220	2	2	2	2	2	2
1	2	212	4	4	4	4	4	4
1	1	286	1	1	1	1	1	1
1	1	322	4	4	4	1	4	4
2	2	341	2	1	3	1	3	1
2	2	245	1	2	2	4	1	4
1	1	334	1	1	1	1	1	1
1	1	180	1	1	1	1	1	1
2	2	269	3	2	2	1	3	1
1	1	262	1	1	1	1	1	1
4	4	269	3	4	4	3	3	3
1	2	317	2	2	2	2	2	2
1	1	271	2	2	4	2	3	4
1	2	224	2	2	2	2	2	2
1	1	267	1	1	1	1	1	1
1	1	213	1	1	3	1	1	1
3	3	274	2	2	2	2	2	1
1	1	336	1	1	1	1	1	1
3	3	207	3	3	3	3	3	3
5	5	225	1	1	1	1	1	1
1	1	226	4	1	1	1	1	1
2	2	217	2	2	2	2	2	2
4	2	262	3	3	3	3	3	2
2	1	242	3	3	3	3	3	3
2	2	238	2	1	1	1	2	1
2	2	241	2	1	1	1	4	1
2	1	219	1	3	1	1	1	2
2	2	268	1	2	2	2	1	1
3	3	206	3	3	2	1	2	2
2	2	212	1	1	1	1	1	2
3	3	249	2	2	1	1	2	2
3	3	244	2	2	1	2	2	1
2	2	211	2	2	2	1	2	3
2	2	253	2	2	2	2	2	2
1	1	319	1	1	1	1	1	2
3	3	239	2	2	1	2	2	1
3	3	224	3	3	3	3	3	3
5	5	179	2	4	2	1	2	3
2	2	273	1	1	2	1	1	1
2	2	289	2	1	1	1	2	1
2	2	194	2	3	2	3	3	2
2	3	201	4	4	4	4	4	4
1	1	342	1	1	1	1	1	1
2	3	201	2	2	2	2	2	2
2	2	275	2	2	2	2	2	2
4	2	259	2	2	1	1	1	1
1	1	245	1	1	1	1	1	1
1	1	265	1	1	1	1	1	1
2	2	256	1	1	1	1	1	1
3	3	210	2	2	2	2	2	2
2	2	243	3	4	2	4	3	2

2	2	249	2	2	1	1	1	1
2	2	220	3	3	3	3	2	2
1	3	211	3	2	2	1	3	2
2	2	265	1	1	1	1	1	1
4	3	182	3	3	2	2	3	3
1	1	211	4	1	1	1	4	3
2	2	213	3	2	1	1	2	2
3	3	206	4	3	2	2	2	3
1	1	301	2	1	2	1	2	2
1	1	304	1	1	1	1	1	1
2	2	256	1	1	1	1	1	1
1	1	160	4	4	4	4	4	4
2	2	260	1	2	2	1	2	1
3	3	199	2	2	2	2	3	2
1	1	284	1	1	1	1	4	1
1	5	207	3	3	4	4	4	3
2	2	260	2	2	1	1	1	1
3	3	195	3	4	3	3	4	2
2	2	245	2	2	2	2	3	2
4	4	143	1	1	1	1	1	1
5	2	250	2	2	2	1	1	1
3	3	213	3	3	3	3	3	3
2	2	234	3	3	2	2	3	1
4	4	237	3	3	4	3	2	4
2	2	256	3	2	4	2	2	4
2	2	227	2	1	1	2	2	2
3	3	211	1	1	1	1	1	1
5	5	142	4	2	2	1	4	2
1	4	195	4	2	2	2	3	2
2	2	248	2	2	3	3	2	1
1	1	248	2	1	1	1	2	2
2	2	249	2	2	1	2	2	2
3	3	225	3	2	2	2	2	2
3	3	291	3	3	3	2	2	2
2	2	224	4	1	1	4	4	4
2	1	252	3	3	4	3	2	4
2	2	242	2	2	2	1	2	1
4	4	217	2	1	1	1	2	1
2	3	217	2	2	2	1	2	1
2	2	194	1	1	1	1	1	1
1	1	314	3	3	3	3	3	3
2	2	251	1	1	1	1	1	1
3	1	252	1	1	1	1	1	1
1	3	232	1	1	1	2	2	2
2	2	257	2	1	1	2	1	1
2	3	205	2	1	1	1	2	1
2	2	251	3	2	2	2	3	3
3	3	210	2	1	1	1	2	2
2	3	217	3	1	2	1	4	1
2	4	235	1	1	2	3	2	4
2	2	270	1	1	1	1	1	1
1	1	303	1	1	1	1	1	1
1	1	274	1	1	1	1	1	1
2	2	285	1	1	1	1	1	2

2	2	216	2	2	1	1	2	2
2	2	253	2	2	2	2	2	3
3	2	241	2	2	2	2	2	2
3	2	244	1	1	1	1	1	1
1	2	228	3	2	2	1	2	2
2	3	240	1	2	1	2	1	2
5	2	233	1	1	1	1	1	1
1	1	287	4	4	4	2	3	3
1	2	276	2	2	1	2	2	2
2	1	278	2	2	3	1	3	2
1	1	307	2	2	3	1	3	2
1	1	290	2	3	1	2	3	3
2	2	252	1	2	2	2	2	2
3	3	236	1	2	3	1	2	1
1	1	314	1	2	1	1	4	1
2	2	241	1	1	1	1	1	1
1	1	314	1	1	1	1	1	1
2	2	234	2	1	2	1	1	1
1	1	230	3	3	3	2	2	2
3	4	169	2	1	1	2	3	2
1	1	285	1	2	1	2	2	1
1	3	230	3	2	2	2	2	2
1	1	306	1	1	1	1	1	1
1	1	317	1	1	1	1	1	1
1	3	329	1	1	1	1	4	1
4	4	180	1	1	1	1	3	3
2	1	299	1	1	1	1	1	1
1	1	317	1	1	1	1	4	1
3	2	254	1	1	1	1	1	2
1	1	322	2	2	1	1	1	1
1	2	258	1	1	1	1	3	2
2	1	275	2	2	1	2	1	1
4	3	205	2	2	2	2	4	2
1	1	312	2	1	1	1	1	1
1	2	259	1	1	1	1	4	1
1	2	280	2	1	1	1	1	1
1	1	240	2	2	2	1	1	2
4	5	110	1	1	1	1	1	1
3	3	204	4	4	4	4	4	1
1	1	286	2	1	1	1	1	1
1	1	322	1	1	1	1	1	1
3	3	236	3	3	1	2	2	2
3	3	210	1	1	1	1	1	1
1	2	253	1	1	1	1	1	1
2	3	200	2	3	2	2	2	3
1	2	228	2	2	1	1	2	1
1	1	260	2	3	1	3	4	1
3	3	229	2	1	3	1	2	1
1	2	284	1	1	1	1	1	1
1	3	246	1	2	1	2	1	1
1	1	263	1	1	1	1	2	1
1	1	319	1	2	1	1	1	1
1	3	220	1	2	2	2	3	2
1	1	301	3	1	1	1	2	1

1	1	237	4	3	2	2	3	2
3	2	274	1	2	3	2	2	1
1	1	234	1	2	2	1	2	1
5	5	80	1	1	1	2	4	2
2	2	232	2	2	1	2	2	2
3	3	173	2	2	3	3	1	2
2	2	226	2	2	1	2	2	1
1	1	244	1	1	1	1	4	1
2	2	269	1	2	2	1	3	3
3	2	204	2	2	1	2	3	2
2	2	280	1	1	1	1	1	1
2	2	204	1	2	1	1	2	3
3	3	237	1	4	2	1	4	3
1	1	327	1	1	1	1	2	1
1	1	335	1	1	1	1	2	1
3	3	211	2	1	1	1	2	1
2	2	252	2	2	2	2	2	2
1	1	303	1	2	1	1	1	1
3	3	234	1	2	1	1	1	1
2	2	239	1	1	1	1	1	1
3	4	238	3	2	2	3	2	2
1	3	180	1	2	2	2	3	1
4	1	262	1	1	1	1	1	1
3	3	245	3	2	2	1	2	2
1	1	238	1	1	1	1	1	1
3	3	206	1	2	2	1	2	1
3	3	204	2	2	2	2	2	2
1	1	305	2	2	2	1	1	2
3	3	210	2	2	2	2	2	2
3	2	245	1	2	1	1	1	1
3	3	228	2	3	2	1	1	2
3	3	214	2	1	2	2	3	2
2	1	182	1	2	1	1	2	1
2	2	222	1	1	1	1	1	1
2	2	248	2	2	1	1	1	1
2	2	234	2	2	2	2	2	3
1	1	269	1	2	1	1	1	2
2	2	297	1	1	1	1	1	1
1	1	255	2	3	1	1	2	1
3	3	226	1	1	1	1	1	1
1	2	213	1	3	3	1	1	1
1	1	258	1	1	1	1	1	1
2	2	272	2	3	2	2	3	3
2	2	229	1	1	1	1	2	1
3	3	264	2	2	1	1	1	1
2	2	192	1	1	1	1	3	1
2	2	226	1	1	1	1	1	1
1	1	304	2	2	1	2	1	1
1	1	278	2	1	1	1	1	1
1	1	279	1	2	2	1	1	1
1	1	282	2	2	2	2	2	2
2	2	281	3	3	2	2	2	2
1	1	261	2	1	1	2	1	1
2	2	306	2	2	1	1	2	2

2	3	262	2	2	2	2	2	2
2	2	249	2	2	2	2	2	2
1	2	273	2	2	1	1	2	2
1	1	250	2	4	4	4	4	4
1	1	312	1	1	1	1	1	1
2	2	304	2	2	1	1	2	2

D7	D8	D9	D10	D11	D12	D13	D14	D15
1	1	1	1	1	1	1	2	1
1	3	4	2	3	4	1	1	1
2	2	2	2	2	2	2	2	2
1	2	2	1	2	2	2	2	1
1	1	1	4	4	4	4	1	4
1	2	3	1	1	1	1	1	1
3	4	3	3	3	2	2	2	1
1	4	4	1	3	2	3	1	2
1	1	4	3	3	3	2	4	1
1	1	2	1	1	1	1	4	1
2	3	4	2	3	3	2	3	2
3	2	3	3	3	1	3	3	1
1	1	3	1	1	1	1	2	1
4	4	4	4	4	4	4	4	4
3	4	4	2	2	2	3	2	2
1	1	2	1	1	1	1	2	1
2	2	3	1	1	1	1	2	1
4	4	4	4	4	4	4	4	4
1	2	3	1	1	1	1	2	1
2	2	4	2	2	3	2	3	2
2	2	3	3	2	3	2	2	2
3	3	3	3	3	3	3	3	4
1	1	1	1	1	1	1	1	1
1	3	3	2	2	2	2	1	1
1	3	3	1	1	3	1	1	1
2	3	3	3	2	2	3	2	3
2	2	3	2	2	2	2	2	2
2	2	1	1	1	2	1	2	1
3	2	4	1	1	2	2	3	1
1	1	1	1	1	2	2	1	2
1	1	3	1	2	1	1	1	1
1	1	2	1	1	1	2	2	1
4	4	4	4	2	4	4	2	4
3	3	3	3	3	3	3	3	3
4	3	4	3	2	2	2	3	1
1	1	1	1	1	1	2	1	1
4	4	4	4	4	4	4	4	4
4	4	4	4	4	4	4	4	4
1	1	1	1	1	1	1	1	1
1	1	1	4	1	4	1	4	1
1	1	1	1	1	1	1	1	1
1	1	1	1	1	1	1	1	1
3	3	3	2	2	2	2	2	2
1	1	1	1	1	1	1	1	1
3	3	4	1	4	2	4	2	4
3	3	3	3	3	3	3	3	3
1	1	1	1	1	1	1	1	1
4	4	4	4	4	4	4	4	4
1	1	1	1	1	1	1	1	1
3	3	2	3	2	3	3	2	3
1	4	4	4	4	4	4	4	4
1	1	1	1	1	1	1	1	1
1	1	1	1	1	1	1	1	1

1	3	4	3	3	3	3	2	2
1	2	4	1	2	2	2	2	1
2	4	2	3	2	3	2	3	4
2	2	2	2	2	2	2	2	2
4	4	4	4	2	2	2	2	2
1	1	1	1	1	1	1	1	1
3	1	1	1	1	1	1	1	1
1	1	1	1	1	1	1	1	1
1	3	4	4	3	1	1	3	2
1	1	2	1	1	1	2	3	1
1	2	3	1	1	2	2	2	1
2	2	3	1	2	1	2	1	2
1	1	1	1	1	1	1	1	1
1	1	1	1	1	1	1	1	1
1	1	1	1	1	1	1	1	1
1	1	1	1	1	1	1	1	1
1	1	1	1	1	1	1	1	1
1	1	1	1	1	1	1	1	1
1	2	2	1	1	1	1	3	1
1	2	3	2	3	3	2	2	2
1	3	3	2	2	3	2	3	2
2	3	4	2	3	3	2	3	2
1	3	3	4	2	2	1	4	3
2	2	2	2	2	2	2	1	2
1	1	1	1	1	1	1	1	1
1	2	4	4	2	2	2	1	3
1	1	2	1	1	1	1	2	1
1	2	4	3	2	2	2	2	1
1	2	4	4	3	2	2	2	2
1	1	1	1	1	1	1	1	1
2	3	4	2	3	3	2	1	2
1	4	4	1	1	4	4	1	2
1	3	4	2	2	2	2	2	3
1	1	2	2	2	2	1	2	1
2	2	3	3	3	2	2	2	1
1	1	1	1	1	1	1	1	1
2	2	2	1	2	4	2	4	1
2	3	3	1	1	1	2	1	2
1	1	1	1	1	1	1	1	1
1	1	1	1	1	1	1	1	1
2	2	3	3	3	3	3	2	2
2	2	3	3	2	3	3	2	2
1	1	4	4	4	4	3	3	2
1	2	1	1	1	1	1	2	1
3	1	4	3	4	4	4	4	3
2	2	2	2	2	2	2	2	2
2	2	2	1	1	1	1	1	1
1	4	4	1	4	1	2	1	1
1	1	1	1	1	1	1	1	1
1	1	2	1	1	1	1	2	1
2	2	2	2	2	2	3	2	2
2	1	3	1	1	1	1	1	1
1	1	1	1	1	1	1	1	1
2	2	4	2	2	2	2	2	2

4	2	3	3	3	3	3	3	3
3	3	3	3	2	3	2	1	2
3	3	4	1	2	2	4	4	3
4	1	4	4	1	1	4	1	4
2	2	2	2	2	2	2	2	3
2	2	4	1	2	1	1	1	1
1	3	4	2	2	2	2	2	1
1	1	4	2	1	1	1	1	1
1	1	1	1	1	1	1	4	1
1	2	3	1	2	2	1	2	2
3	4	3	4	4	3	3	2	3
1	1	1	1	1	1	1	1	1
1	1	4	1	2	4	3	2	2
2	2	3	2	2	2	2	1	2
2	2	2	2	2	2	2	2	1
1	3	4	1	1	1	1	4	1
2	2	4	2	4	4	4	2	2
1	1	4	1	1	1	1	1	1
2	3	4	2	4	3	2	3	2
3	3	3	3	3	3	3	3	3
1	1	2	1	1	1	1	3	1
1	1	1	1	1	1	1	1	1
4	4	4	4	4	4	4	4	4
3	2	4	2	4	3	3	2	2
2	3	3	2	2	2	2	1	1
1	2	3	3	1	3	4	1	3
2	2	4	2	1	2	2	2	2
2	2	3	2	2	3	3	1	3
1	2	4	2	2	2	2	2	2
1	2	1	1	1	1	1	2	1
1	4	4	2	2	2	2	2	2
1	2	2	1	1	2	2	1	1
2	2	2	1	2	3	3	2	2
2	2	4	2	2	1	2	2	2
1	1	1	1	1	1	1	1	1
2	2	3	2	2	2	3	2	1
1	1	1	1	1	1	1	1	1
1	1	1	1	1	1	1	1	1
1	1	1	1	3	1	1	1	1
2	2	2	2	2	2	2	2	2
1	2	3	2	2	3	3	2	2
2	3	4	3	4	3	3	1	3
3	3	1	1	1	4	3	4	4
1	1	4	2	2	2	2	1	2
1	1	4	1	1	1	1	4	1
1	2	3	2	1	3	1	2	1
4	4	4	4	4	3	2	2	2
4	2	3	1	2	2	2	2	1
1	2	4	2	2	3	2	4	1
2	3	4	2	4	4	4	3	1
1	2	4	1	1	1	1	2	1
1	1	4	1	2	1	1	4	1
3	4	4	2	3	3	2	2	2
1	1	1	1	1	1	1	1	1

1	1	3	1	1	1	1	2	1
1	1	2	1	1	2	1	2	1
1	1	2	1	4	1	1	1	1
1	1	3	2	1	2	2	1	1
2	2	2	1	1	2	2	2	2
1	2	2	1	2	1	1	1	1
1	2	2	1	1	1	1	1	1
1	2	2	1	1	1	1	2	1
4	4	4	4	4	4	4	4	4
2	3	4	1	4	3	3	2	3
1	1	4	1	1	1	1	4	1
2	2	4	3	2	2	4	1	4
2	2	2	1	1	1	1	2	1
1	1	2	1	1	3	1	3	1
1	2	3	1	2	1	1	1	1
1	2	4	1	1	1	1	2	1
1	1	3	1	2	1	1	3	1
1	1	3	2	1	2	2	2	1
1	1	1	2	3	1	2	3	2
1	1	1	1	1	1	1	1	1
1	2	2	1	1	1	1	1	1
2	3	4	1	1	2	2	1	2
2	2	4	1	2	1	2	4	2
1	1	1	2	2	1	1	1	1
2	2	4	3	1	1	1	1	1
1	2	1	1	2	1	1	1	1
3	3	2	1	2	2	2	1	3
3	3	4	3	4	4	4	4	4
1	2	4	4	3	1	1	1	2
4	1	1	1	1	1	1	1	1
1	1	3	2	2	1	2	1	2
4	1	2	1	1	3	2	2	1
1	2	1	1	1	1	1	1	1
2	4	4	4	3	2	3	1	3
1	2	4	2	2	2	2	1	2
2	2	3	3	2	4	2	2	1
2	2	1	1	2	1	1	1	1
2	2	4	3	2	2	3	4	4
1	2	2	1	1	2	2	2	1
4	4	4	3	3	3	2	3	2
2	2	2	2	2	2	2	4	2
2	2	2	2	2	2	2	2	2
4	4	4	4	4	4	4	2	1
1	1	1	1	1	1	1	1	1
2	2	3	2	2	3	2	2	2
2	2	2	1	2	2	2	1	1
1	1	1	1	1	1	1	1	1
1	1	1	1	1	1	1	1	1
1	1	1	1	1	1	1	1	1
2	3	4	1	1	1	1	4	1
1	4	1	1	1	2	1	2	1
1	1	1	1	1	1	1	1	1
1	1	1	1	1	1	1	1	1
1	3	4	1	3	2	2	2	2

1	1	2	2	1	1	1	4	1
1	4	4	1	4	4	1	4	4
1	2	3	1	1	1	2	1	1
1	1	2	1	1	1	1	1	1
2	2	4	2	2	2	2	2	2
1	1	1	1	1	1	1	1	1
4	4	4	4	4	4	4	4	4
1	3	2	2	2	2	2	1	2
2	2	3	1	2	2	2	1	2
2	3	3	4	2	3	3	2	2
4	4	4	1	4	4	4	2	4
2	3	4	1	3	4	3	4	1
1	3	2	2	2	2	2	1	2
2	1	1	1	2	1	1	2	1
1	2	4	1	4	4	2	3	1
2	2	2	2	2	2	2	2	1
2	2	2	2	2	2	2	2	2
1	1	1	1	1	1	1	1	1
1	1	2	2	1	1	1	2	1
1	4	3	2	3	4	4	3	4
1	2	1	1	2	2	2	2	1
3	3	3	1	3	2	2	3	1
4	2	3	1	1	1	1	4	1
1	4	4	2	1	2	2	1	1
2	2	2	2	2	2	2	2	1
3	4	4	4	2	2	2	2	3
1	1	2	1	1	1	1	1	1
1	1	4	1	1	1	1	1	1
1	2	4	4	2	3	2	2	1
4	2	2	2	2	2	2	2	2
3	3	3	3	3	3	3	3	3
3	1	2	1	4	4	3	1	3
2	3	4	4	4	4	4	3	3
2	2	2	2	2	2	2	2	2
3	1	3	1	2	2	2	1	1
1	3	4	1	2	2	2	2	1
2	1	2	1	1	1	1	2	1
3	4	1	1	4	1	1	3	1
1	1	1	1	1	1	1	1	1
1	1	2	1	1	1	1	1	1
1	1	4	1	1	1	1	1	1
1	2	3	1	1	2	1	2	1
4	3	4	2	4	4	2	2	3
1	1	1	1	1	1	1	1	1
4	4	4	4	4	4	4	3	4
1	1	1	1	1	1	1	1	1
1	1	1	1	2	1	2	1	1
1	1	2	1	1	1	1	2	1
4	4	4	2	4	3	2	2	2
1	3	3	2	2	3	2	2	1
1	3	3	2	2	3	2	2	1
1	1	3	1	1	1	1	3	1
1	1	2	1	1	1	1	1	1
2	2	3	1	1	2	2	2	1

1	1	4	1	2	2	2	4	1
1	1	1	1	1	1	2	2	1
1	2	4	2	1	1	1	1	1
1	2	3	2	2	2	2	2	2
4	4	4	4	4	4	4	4	4
2	2	2	2	2	2	2	2	2
3	3	3	3	3	1	3	3	3
1	1	1	1	1	1	1	1	1
1	2	4	2	2	1	2	1	2
1	2	4	1	3	2	2	2	2
2	2	2	1	2	2	2	2	2
4	4	4	2	4	3	2	2	2
2	2	4	2	2	2	2	2	2
1	1	4	2	1	1	1	1	1
1	1	1	1	1	1	1	1	1
1	1	1	1	1	1	1	1	1
3	3	4	3	4	4	4	4	4
1	1	1	1	1	1	1	1	1
1	1	1	1	1	1	1	1	1
2	2	3	2	3	3	3	3	2
1	2	4	1	1	3	4	4	3
2	2	3	2	3	2	1	2	1
1	1	3	2	2	1	2	3	1
1	1	4	2	1	1	1	4	1
3	3	3	3	3	3	3	3	3
3	3	3	1	2	1	3	4	1
1	3	3	2	2	4	4	1	3
2	3	3	3	3	3	3	3	3
2	4	4	2	1	4	1	4	2
1	1	1	1	1	1	1	1	1
1	1	2	2	2	1	2	1	3
1	1	1	1	1	1	1	1	1
1	1	1	1	1	1	1	1	1
1	1	2	2	1	1	1	2	1
1	2	3	1	1	2	2	2	2
3	3	3	3	3	3	3	3	2
1	1	1	1	1	1	1	1	1
1	1	3	2	2	1	1	2	1
1	2	2	1	2	1	1	2	1
3	3	2	3	3	2	3	3	2
1	2	2	2	3	3	3	3	2
1	2	2	1	1	3	1	3	1
2	2	2	1	1	1	2	1	1
1	1	1	1	1	1	1	1	1
2	3	3	2	3	3	2	3	2
1	2	2	2	2	2	1	2	1
2	2	2	2	2	2	2	2	2
1	1	1	1	1	1	1	1	1
1	1	2	1	2	2	1	1	1
2	3	3	2	3	3	2	3	2
1	2	2	3	1	1	1	1	1
1	1	2	1	1	1	1	1	1
1	3	3	1	1	1	1	2	1
2	4	4	4	3	3	3	2	3

1	2	3	1	1	1	1	1	1
1	2	1	1	1	1	1	2	1
2	2	4	2	2	3	2	3	2
1	1	1	1	1	1	1	1	1
4	4	4	4	4	4	4	4	4
1	3	3	3	2	2	2	3	2
1	3	2	1	1	2	1	2	1
2	2	4	2	4	2	2	3	4
2	2	2	2	2	2	1	2	1
4	4	4	4	4	4	4	4	4
1	1	4	1	2	2	2	3	1
1	2	2	1	1	2	1	2	1
3	3	3	3	3	3	3	3	3
2	2	2	2	2	2	2	2	2
2	3	3	2	2	2	2	3	2
4	4	4	4	4	4	4	4	4
2	1	2	1	1	2	1	1	1
1	2	3	1	2	2	2	1	1
3	3	3	3	3	3	3	3	3
1	3	4	3	3	2	3	2	2
2	3	4	2	3	3	2	2	2
3	2	3	3	3	3	3	3	3
1	1	1	1	1	1	1	1	1
1	3	2	1	1	1	1	1	1
1	1	2	1	1	1	1	1	1
1	1	4	3	1	3	1	3	1
2	3	3	1	2	2	2	2	2
1	2	2	2	1	2	1	2	1
1	2	2	1	2	2	1	2	1
2	2	3	2	2	1	2	2	2
2	1	2	1	2	1	1	2	1
2	2	2	1	2	2	2	4	2
2	2	3	2	2	3	2	4	2
2	2	4	2	2	3	2	2	2
1	1	1	1	1	1	1	1	1
1	1	3	1	1	1	1	1	1
1	1	4	4	2	1	2	1	1
1	1	3	1	2	1	1	1	1
2	2	2	2	2	1	1	3	1
4	4	4	4	4	4	4	4	4
1	1	3	3	2	1	1	2	1
3	2	3	2	3	3	4	4	2
2	3	4	2	2	2	2	2	3
1	2	3	1	1	1	1	2	1
1	4	4	1	4	4	4	4	4
2	2	2	1	2	2	2	2	2
1	1	2	2	1	1	1	2	1
1	1	1	1	1	1	1	1	1
1	1	1	1	1	1	1	1	1
3	3	3	3	3	3	3	3	3
3	3	4	1	4	2	4	2	4
3	3	3	3	3	3	3	3	3
2	3	2	2	2	3	2	2	3
2	2	2	2	2	2	2	2	2

1	1	3	2	1	1	1	2	1
1	1	1	1	1	1	1	1	1
3	3	2	3	3	2	3	3	2
2	2	2	2	2	2	2	2	2
2	2	3	2	2	2	2	2	2
1	1	1	1	1	1	1	1	1
1	2	2	1	1	2	1	1	1
1	1	2	1	1	1	1	1	1
3	3	4	2	3	2	2	2	2
3	3	4	1	4	4	3	2	1
1	2	2	1	2	2	1	1	1
4	4	4	4	4	4	3	2	3
3	3	3	1	3	2	2	2	1
2	1	4	1	2	1	1	2	1
1	1	2	1	2	1	2	2	1
3	4	4	1	2	1	2	1	1
1	2	2	1	2	2	2	3	1
1	1	3	1	2	1	2	1	1
1	1	4	4	1	1	1	1	1
3	4	2	4	3	2	2	2	2
2	2	2	1	1	2	2	3	2
1	1	1	1	1	1	1	1	1
2	2	2	2	2	2	2	2	2
1	2	2	1	1	1	1	1	1
1	1	3	2	2	2	2	2	2
1	1	1	1	1	1	1	1	1
1	1	2	1	1	2	1	1	1
1	1	4	1	1	1	1	4	1
1	2	2	2	2	1	2	2	1
4	4	4	4	4	4	4	4	4
1	1	2	2	1	1	1	2	1
2	2	2	2	2	2	2	2	2
1	3	2	1	2	2	2	3	2
1	1	2	2	2	2	1	3	3
1	1	3	1	1	1	1	2	3
1	1	2	2	2	2	2	1	1
2	4	4	4	4	4	4	4	4
1	2	3	1	2	2	1	3	2
2	2	2	2	2	2	2	2	2
1	2	3	2	1	1	1	2	1
1	3	4	1	1	1	1	3	1
1	1	2	1	2	2	1	2	2
1	1	4	1	3	3	2	3	1
1	1	1	1	1	1	1	1	1
3	3	3	3	3	3	3	3	3
1	1	3	1	2	1	1	3	1
1	1	1	1	1	1	1	3	1
2	3	3	3	2	1	2	2	2
4	4	4	4	4	4	4	4	4
1	1	2	1	1	1	1	1	1
4	2	3	2	2	2	2	1	1
2	3	3	3	1	2	2	3	1
2	2	3	1	2	1	1	2	1
2	2	2	1	1	2	1	2	1

1	3	4	1	2	3	1	4	1
1	2	4	4	4	4	3	4	4
3	3	3	3	3	3	3	3	3
2	2	4	2	3	3	2	3	1
1	4	2	1	4	4	4	1	1
1	1	4	1	1	2	1	2	1
1	1	2	1	1	1	1	1	1
2	3	3	3	1	2	2	3	1
1	1	2	2	1	1	1	1	1
4	4	4	4	4	4	4	4	4
1	1	2	1	1	1	1	1	1
1	1	1	1	1	2	1	1	1
1	2	4	2	2	2	2	2	2
1	1	1	2	2	2	2	1	2
2	2	2	2	2	2	2	2	2
1	1	1	1	1	1	1	1	1
1	1	1	1	1	1	1	1	1
2	3	3	3	2	2	2	4	1
1	3	3	1	2	2	1	2	1
3	4	4	2	2	2	2	3	2
2	2	3	3	3	3	3	3	2
1	1	2	1	2	1	1	1	1
2	2	2	2	2	2	2	2	1
1	2	3	1	1	2	1	2	1
2	2	3	1	2	2	2	1	1
3	4	4	4	4	4	4	2	2
1	2	1	1	1	1	1	1	1
1	1	2	1	1	1	1	2	1
4	4	4	4	4	4	4	4	4
1	1	1	1	2	1	1	2	1
2	2	2	2	2	2	2	2	2
1	1	4	1	4	4	1	4	1
3	2	4	3	3	3	3	2	2
1	1	3	2	1	1	1	2	1
1	1	1	1	1	1	1	1	1
2	2	2	2	2	2	2	2	2
1	1	2	1	1	1	1	2	1
2	3	2	3	2	3	2	2	2
3	3	3	3	3	3	3	3	3
2	2	2	1	2	1	2	2	1
1	1	4	3	1	2	3	2	2
1	1	4	2	2	1	1	2	1
2	2	3	1	2	2	1	2	1
1	2	2	1	2	1	1	2	1
1	2	2	1	1	1	1	1	1
1	1	2	1	1	1	1	1	1
2	2	4	2	4	2	2	3	4
1	1	2	1	1	1	1	1	1
3	3	4	3	2	2	2	1	2
2	2	3	2	2	3	2	2	1
2	2	2	2	2	2	2	2	2
1	4	4	4	4	4	4	1	2
3	3	4	4	4	4	2	2	3
2	4	4	1	2	3	3	2	4

2	2	2	2	2	2	2	2	2
4	4	4	4	4	4	4	4	4
1	1	1	1	1	1	1	1	1
3	2	3	2	3	2	3	2	3
2	2	2	2	2	2	2	2	2
1	2	2	1	1	1	1	2	1
1	1	1	2	1	2	1	1	1
2	3	1	1	1	1	1	1	2
1	1	4	2	1	1	1	1	1
1	1	1	1	1	1	1	1	1
1	4	4	2	2	2	3	2	1
1	1	1	1	1	1	1	2	1
1	1	1	1	1	1	1	1	1
1	1	1	1	1	1	1	2	1
2	2	2	1	2	1	2	1	1
1	3	4	3	3	3	3	2	2
2	2	4	2	2	2	1	1	1
2	2	3	2	4	2	2	2	2
1	1	2	1	1	1	1	1	1
2	1	4	1	3	2	2	1	3
2	2	2	2	2	2	2	2	2
2	2	2	2	2	2	2	2	2
3	3	3	3	3	3	3	3	3
3	3	3	3	3	3	3	3	3
2	2	2	1	2	2	2	3	1
1	4	4	1	1	1	1	3	1
2	2	4	3	3	3	3	2	2
1	2	2	2	3	3	3	3	2
4	4	4	4	3	4	4	4	4
1	2	3	1	1	2	1	2	1
1	1	1	1	1	1	1	1	1
2	2	2	1	2	2	2	4	2
2	2	2	2	2	2	2	1	2
3	3	3	3	3	3	3	3	3
1	1	1	1	1	1	1	2	1
4	4	4	4	4	4	4	4	4
4	4	4	4	4	4	4	4	4
2	2	2	2	2	2	2	2	2
4	2	3	4	2	3	3	3	3
1	1	1	1	1	1	1	1	1
3	3	3	3	3	3	3	3	3
2	2	2	3	3	3	3	3	3
1	1	2	1	1	1	1	1	1
1	1	2	1	1	2	1	1	1
2	2	2	2	2	2	2	2	2
2	3	4	1	2	3	1	2	2
1	1	1	1	1	1	1	3	1
2	1	3	2	2	2	2	3	1
1	2	3	1	2	2	2	2	1
4	4	4	4	4	4	4	4	4
2	2	2	2	2	2	2	2	2
2	1	2	2	2	3	1	1	2
1	1	2	1	1	1	1	2	1
1	1	1	1	1	1	1	1	1

1	1	1	1	1	1	1	1	1
1	1	1	1	1	1	1	1	1
1	1	2	1	1	1	1	1	1
2	2	3	2	2	3	1	3	2
1	1	3	1	2	3	2	3	2
1	1	1	1	1	1	1	1	1
2	2	3	2	3	4	2	4	1
2	2	3	1	1	1	1	1	1
1	3	3	1	1	1	1	1	1
2	2	4	1	2	2	2	3	2
1	1	2	1	2	1	1	2	1
3	3	3	3	3	2	2	3	2
1	1	2	1	1	1	1	2	1
2	2	3	2	2	2	2	3	1
1	2	1	2	1	2	1	2	1
1	3	4	1	2	2	2	2	2
2	3	2	2	3	2	2	2	2
2	1	2	1	1	2	1	2	1
1	1	2	1	2	2	1	2	1
1	2	1	1	1	1	1	1	1
2	2	2	1	1	1	1	2	1
1	1	3	3	2	2	2	2	1
3	3	3	3	3	3	3	3	4
2	3	3	3	3	3	3	2	3
2	3	1	3	1	2	2	1	2
1	3	3	2	2	2	2	2	1
2	2	2	2	2	2	2	2	2
2	2	3	1	2	2	2	2	2
1	4	4	2	2	2	2	2	2
4	4	4	4	4	4	4	2	3
1	1	1	1	1	1	1	1	1
1	1	3	1	1	2	2	4	1
4	4	4	4	4	4	4	4	4
1	2	2	1	1	1	1	3	1
1	2	3	1	1	1	1	1	1
2	2	3	2	2	2	2	3	1
1	1	1	1	1	1	1	1	1
2	2	2	2	2	2	2	2	2
1	1	1	1	1	1	1	1	1
1	2	2	1	2	1	1	2	1
4	4	4	4	4	4	4	4	4
1	1	1	1	1	1	1	1	1
1	1	2	1	1	1	1	1	1
1	2	1	1	2	1	1	1	1
1	1	2	1	1	1	1	4	1
4	4	4	4	4	4	4	4	4
2	3	4	1	3	3	2	4	2
1	3	3	3	2	2	2	2	2
1	2	3	1	2	2	1	2	2
3	3	4	3	3	4	3	3	3
4	4	4	3	4	4	3	3	4
1	1	1	1	1	1	1	2	1
1	1	1	1	1	1	1	1	1
1	2	2	1	2	1	2	1	1

1	1	1	1	1	1	1	1	4
3	3	4	4	4	3	4	3	3
3	2	3	1	2	2	2	2	2
1	1	1	1	1	1	1	3	1
4	4	4	4	4	4	4	4	4
3	1	4	1	3	1	1	1	1
1	2	2	2	3	3	2	2	2
3	3	3	3	3	3	3	3	3
4	4	4	4	4	4	4	4	4
1	1	1	1	1	1	1	4	1
2	1	1	1	1	4	1	1	1
3	3	3	3	3	3	3	3	3
1	1	1	1	1	1	1	1	1
1	1	1	1	1	1	1	1	1
1	1	1	1	1	2	1	1	1
2	2	3	2	3	2	3	1	2
1	1	1	1	1	1	1	1	1
1	1	1	1	1	1	1	1	1
1	1	2	1	1	2	2	3	1
1	2	2	1	1	1	1	1	1
3	3	4	3	3	4	2	4	2
1	1	1	1	1	1	1	1	1
1	1	1	1	1	1	1	2	1
2	2	2	2	2	2	2	2	2
1	1	3	1	1	1	1	1	1
2	3	2	4	2	1	1	1	1
1	1	4	1	1	1	1	2	1
1	1	1	1	1	1	1	1	1
3	3	3	1	2	2	2	2	2
1	2	2	1	1	1	1	1	1
4	2	2	2	2	2	1	1	2
2	4	3	1	1	1	1	2	1
1	1	1	1	1	1	1	1	1
1	2	2	1	1	1	1	2	1
2	2	2	1	2	2	1	2	2
1	2	3	3	2	4	1	2	4
1	4	4	1	1	1	1	3	3
1	1	1	1	1	1	1	1	1
2	3	3	1	2	3	2	3	2
2	3	3	1	1	2	2	3	2
1	2	3	1	2	1	1	2	1
1	1	1	1	1	1	1	1	1
1	1	1	1	1	1	1	1	1
1	1	1	1	1	1	1	3	1
1	1	1	1	1	1	1	1	1
1	1	2	1	2	3	1	2	1
1	1	1	1	1	1	1	1	4
2	2	4	1	2	2	1	2	2
2	2	3	1	3	3	3	2	2
3	3	4	2	3	2	2	2	2
3	3	3	3	3	3	3	3	3
1	1	2	1	2	3	1	2	1
1	1	1	1	1	1	1	1	1
3	3	4	1	4	4	3	4	1

3	3	3	3	3	3	3	3	3
1	4	4	1	1	2	1	3	1
1	1	1	1	1	1	1	1	1
2	2	2	2	2	2	2	2	2
1	4	4	4	4	4	4	4	1
1	1	1	1	1	1	1	1	1
4	4	4	4	4	4	4	4	4
1	1	4	4	3	3	4	1	3
4	2	4	4	3	3	3	4	1
1	1	1	1	1	1	1	1	1
1	1	1	1	1	1	1	1	1
2	3	4	1	2	2	1	3	2
1	1	1	1	1	1	1	1	1
3	3	3	3	3	3	2	3	3
1	2	2	2	2	1	2	3	1
3	1	4	4	3	3	4	1	2
1	2	2	2	2	1	2	3	1
1	1	1	1	1	1	1	1	1
1	1	3	1	1	1	1	1	1
2	2	1	1	2	2	2	2	2
1	1	1	1	1	1	1	1	1
3	3	3	3	3	3	3	3	3
1	1	1	1	1	1	1	1	1
1	1	4	1	4	1	1	1	1
2	2	2	2	2	2	2	2	2
4	3	2	2	3	2	3	3	2
3	3	3	3	3	3	3	3	3
1	1	2	1	1	1	1	2	1
1	1	4	1	1	1	1	4	1
2	3	3	1	2	2	2	3	2
1	1	2	1	1	2	2	2	1
2	2	2	2	2	2	2	2	2
1	1	3	1	1	2	1	2	1
2	2	3	2	2	2	2	2	1
2	2	2	2	2	2	2	2	1
1	1	3	2	2	2	2	2	2
2	2	2	3	2	2	2	3	2
1	2	2	1	1	1	1	1	1
1	2	2	1	1	1	1	2	1
3	3	3	3	3	3	3	3	2
1	2	4	2	2	1	1	4	2
1	1	1	1	1	2	1	1	1
1	1	2	1	1	1	1	2	1
3	2	3	2	1	2	2	2	1
4	4	4	4	4	4	4	4	4
1	1	1	1	1	1	1	1	1
2	3	3	2	2	2	2	3	2
2	2	2	2	2	2	2	1	1
1	2	2	1	2	1	2	3	1
1	1	2	1	1	2	1	1	1
1	1	2	1	1	1	1	2	1
1	1	1	1	1	1	1	1	1
2	2	2	2	2	2	2	2	2
2	4	2	1	2	2	2	3	2

1	1	2	1	1	1	1	1	1
3	2	3	3	3	3	3	2	2
1	3	4	3	3	3	3	2	4
1	1	1	1	1	1	1	2	1
2	3	3	3	2	2	2	2	1
2	4	4	4	4	4	2	2	1
2	3	2	1	1	2	1	1	1
2	3	3	3	2	2	2	1	2
1	1	3	2	1	2	2	1	1
1	1	1	1	1	1	1	1	1
1	1	1	1	1	1	1	1	1
4	4	4	4	4	4	4	4	4
1	2	2	1	1	1	1	1	1
1	3	4	2	2	2	2	2	2
1	1	3	1	1	1	1	1	2
4	3	4	3	4	4	3	3	3
2	1	2	1	1	2	2	2	1
2	3	3	2	2	3	3	2	3
2	3	3	2	2	2	2	2	2
1	1	3	1	1	1	1	3	1
1	2	2	2	2	2	2	2	1
3	3	3	3	3	3	3	3	3
1	2	2	2	2	2	2	2	2
1	4	4	4	4	4	4	4	4
2	3	2	3	2	2	4	2	4
2	2	2	1	1	1	1	2	1
1	1	2	1	1	1	1	2	1
2	4	4	4	4	4	4	4	4
3	2	2	1	1	3	1	3	2
1	3	2	1	2	2	2	3	1
1	1	2	1	1	1	1	2	1
2	2	3	1	2	2	2	2	2
2	2	2	2	2	2	2	2	2
2	1	1	1	1	1	1	1	1
2	2	1	4	4	4	2	2	2
1	4	4	4	4	4	4	4	4
1	3	2	2	2	1	2	2	2
4	2	1	1	1	1	1	1	2
2	4	3	2	2	2	2	2	2
1	1	1	1	1	1	1	1	1
3	3	3	3	3	3	3	3	3
1	1	2	1	1	1	1	2	1
1	1	1	1	1	1	1	1	1
2	2	2	1	1	1	2	1	1
1	2	3	1	2	2	2	2	1
2	1	2	1	2	2	1	3	1
1	2	3	2	2	3	2	2	2
1	2	3	2	2	2	2	2	1
1	1	4	3	1	3	1	2	1
3	4	4	1	3	2	4	3	3
1	2	4	1	1	1	1	2	1
1	2	2	1	1	1	1	1	1
1	1	1	1	1	1	1	1	1
1	2	2	1	1	1	1	2	1

1	1	2	1	2	1	1	1	1
2	2	3	2	3	2	2	2	3
1	1	1	2	1	1	1	2	1
1	1	1	1	1	1	1	1	1
2	2	3	2	2	2	1	2	1
1	2	2	2	1	1	2	2	2
1	1	1	1	1	1	1	1	1
1	3	4	4	4	3	2	4	2
1	1	2	1	1	1	1	1	1
1	1	2	1	1	1	1	3	1
1	1	2	1	1	1	1	3	1
4	2	4	3	4	4	1	2	1
2	3	3	3	3	3	3	2	3
2	2	2	1	1	1	1	1	1
2	2	3	1	1	1	1	2	1
1	1	1	1	1	1	1	2	1
1	2	1	1	1	1	1	3	1
1	1	1	1	1	1	1	1	1
1	2	3	2	2	2	2	1	2
3	2	2	1	2	1	2	1	2
1	1	2	1	1	1	1	2	1
2	2	2	1	2	2	2	2	2
1	1	1	1	1	1	1	1	1
1	1	1	1	1	1	1	1	1
1	1	4	1	1	1	1	1	2
2	1	2	2	1	1	1	2	1
1	1	1	1	1	1	1	1	1
1	1	4	1	4	1	1	1	1
1	1	2	1	1	1	1	1	1
1	1	1	1	1	1	1	3	1
1	2	4	1	1	3	1	1	1
2	1	2	1	1	1	1	1	1
2	2	4	3	3	2	1	1	2
1	1	2	1	1	1	1	1	1
1	1	3	1	2	2	1	1	1
1	1	1	1	1	1	1	2	1
2	2	2	2	2	2	2	2	1
1	2	4	2	3	2	2	3	1
4	4	1	4	1	4	1	4	1
1	1	2	1	1	1	1	2	1
1	1	2	1	1	1	1	1	1
1	2	4	2	3	3	2	2	2
1	1	1	1	1	1	1	1	1
1	1	2	1	1	1	1	1	1
1	3	1	2	1	2	2	2	1
1	2	4	2	1	2	2	3	1
2	2	2	1	1	2	2	3	3
1	1	2	2	2	2	1	1	1
1	1	2	1	1	1	1	1	1
1	2	4	1	2	2	1	2	1
2	2	2	1	1	1	1	1	1
1	1	2	1	1	1	1	1	1
3	2	3	4	2	4	2	4	2
1	3	3	1	1	1	1	1	1

2	3	4	4	2	2	2	1	2
2	3	3	3	3	1	1	3	2
1	1	2	1	1	1	1	1	1
1	4	4	1	1	1	1	1	1
2	3	2	1	2	2	2	1	1
3	2	4	3	3	4	4	2	4
2	1	3	2	2	2	2	2	1
1	1	4	1	1	1	1	2	1
2	2	4	2	1	1	1	2	1
3	3	4	1	4	2	2	2	2
2	2	3	1	2	2	1	1	2
1	3	2	1	2	1	1	1	1
1	4	3	3	3	2	1	2	1
1	1	1	1	1	1	1	2	1
1	1	1	1	1	1	1	2	1
1	2	1	1	1	2	1	1	1
1	2	2	2	2	2	2	2	1
1	1	1	1	1	1	1	1	1
1	1	1	1	1	1	1	1	1
1	1	2	1	1	1	1	1	1
2	3	4	2	3	3	2	4	2
1	2	3	2	2	1	2	1	2
1	1	1	1	1	1	1	1	1
1	2	2	2	1	2	2	2	1
1	1	2	1	1	1	1	1	1
1	1	2	1	1	1	2	1	1
2	2	2	2	2	2	2	2	2
1	1	2	1	1	1	1	1	1
2	2	2	2	2	2	2	2	2
1	1	1	1	1	1	1	2	1
2	2	3	2	2	2	2	2	2
1	2	3	2	2	2	2	2	1
1	2	2	1	1	2	2	1	1
1	1	3	1	1	2	1	1	1
2	2	2	2	2	2	2	2	1
2	2	2	1	2	2	2	2	2
1	1	2	1	1	1	1	1	1
1	1	1	1	1	1	1	1	1
1	2	2	1	2	2	1	2	1
1	3	3	1	1	1	1	3	1
1	1	1	1	1	2	1	2	1
1	1	1	1	1	1	1	1	1
3	3	3	3	2	3	2	3	2
1	1	2	1	1	1	1	1	1
1	1	2	1	1	1	1	1	1
2	1	3	1	1	1	1	1	1
1	1	1	1	1	1	1	1	1
2	2	3	1	1	1	1	1	1
1	1	2	1	1	1	1	1	1
1	2	2	1	1	2	2	2	1
1	3	2	1	2	2	2	2	1
2	2	3	2	2	2	2	2	3
1	2	1	1	2	3	1	1	1
1	2	3	1	2	3	2	1	1

2	2	2	2	2	2	2	2	2
1	1	3	1	1	1	1	2	1
1	1	2	1	1	1	1	2	1
2	4	4	4	4	2	4	4	2
1	1	1	1	1	1	1	1	1
1	2	3	1	2	3	2	1	1

D16	D17	D18	D19	D20	D21	Negative emotions total scc
1	1	1	1	1	1	23
3	1	1	1	1	1	40
2	2	2	2	2	3	44
1	1	1	1	1	1	33
4	1	4	1	1	1	51
1	1	2	1	1	1	29
1	1	2	2	3	4	53
3	1	2	3	1	1	49
1	2	4	2	2	1	43
1	1	2	1	1	2	29
2	1	1	1	2	1	43
1	1	2	1	1	1	39
1	1	1	1	1	1	27
4	4	4	4	4	4	59
2	4	4	2	2	1	23
1	1	1	1	1	1	30
1	1	1	1	1	1	78
4	1	4	4	4	1	32
1	1	1	1	1	1	41
1	1	2	2	2	1	47
3	1	3	2	2	2	59
3	1	3	3	3	3	21
1	1	1	1	1	1	35
1	1	2	2	1	1	33
3	1	1	1	1	1	46
1	1	2	2	1	2	41
2	1	2	2	2	1	43
2	1	2	1	2	1	35
2	1	3	1	3	1	38
2	1	2	1	1	1	26
1	1	1	1	1	1	26
1	1	2	1	1	1	26
1	4	2	1	1	1	59
3	1	3	3	3	1	48
1	1	4	4	1	1	46
1	1	1	1	1	1	22
4	4	4	4	4	4	84
4	4	4	4	4	4	84
1	1	1	1	1	1	21
1	4	1	4	1	4	51
1	1	1	1	1	1	21
1	1	1	1	1	1	54
2	2	2	2	2	2	63
1	1	1	1	1	1	21
2	2	4	2	3	3	81
3	3	3	3	3	3	26
1	1	1	1	1	1	56
4	4	4	4	4	4	81
1	1	1	2	1	1	23
3	3	2	3	2	3	21
4	4	4	4	4	4	36
1	1	1	1	1	1	64
1	1	1	1	1	1	43

2	1	2	3	3	1	57
1	1	2	1	1	1	21
2	4	3	2	4	3	23
2	1	2	2	2	2	21
2	1	2	2	3	1	56
1	1	1	1	1	1	31
1	1	1	1	1	1	33
1	1	1	1	1	1	35
3	4	2	3	4	4	21
1	1	4	1	1	1	21
1	1	2	1	1	1	25
1	1	1	2	2	1	21
1	1	1	1	1	1	21
1	1	1	1	1	1	21
1	1	2	1	1	1	28
1	1	1	1	1	1	43
1	1	1	1	1	1	44
1	1	1	1	1	1	45
1	1	2	1	1	1	47
2	1	2	1	3	2	40
2	2	3	1	1	1	21
1	1	1	3	2	1	55
4	2	2	2	2	1	26
2	2	2	2	2	2	44
1	1	1	1	1	1	46
1	4	4	4	3	4	22
1	1	1	1	1	1	56
2	2	2	2	2	1	52
2	1	2	1	1	1	45
1	1	1	1	1	1	43
3	2	4	2	4	2	42
2	1	4	3	1	3	21
2	1	1	2	1	1	35
1	1	4	3	2	1	38
1	1	4	1	1	1	21
1	1	1	1	1	1	21
1	1	2	1	1	1	55
1	1	1	1	3	1	51
1	1	1	1	1	1	64
1	1	1	1	1	1	25
3	2	2	2	3	2	58
2	4	2	2	2	3	40
4	2	1	2	4	1	33
1	1	1	1	1	1	33
1	2	3	1	2	3	21
2	1	2	2	2	1	26
1	1	1	1	1	1	42
1	1	3	1	1	1	25
1	1	1	1	1	1	21
1	1	1	2	1	1	36
1	1	3	2	3	2	67
1	1	1	1	1	1	53
1	1	1	1	1	1	59
1	1	3	1	1	1	51

3	3	3	3	4	2	38
3	2	3	4	2	2	33
1	1	4	4	4	1	44
1	1	4	1	4	4	27
2	1	2	1	1	1	26
1	1	1	2	1	1	37
1	1	2	3	3	1	65
1	1	1	1	1	1	21
1	1	1	1	1	1	44
2	1	2	3	2	1	36
2	3	4	4	1	4	35
1	1	1	1	1	1	42
1	1	2	4	1	1	51
1	1	2	1	1	1	27
2	1	2	1	1	1	50
1	1	4	1	3	1	63
1	1	3	2	4	2	29
1	1	1	1	1	1	21
2	1	2	4	2	1	84
3	3	3	3	3	3	51
2	1	2	1	1	1	41
1	1	1	1	1	1	45
4	4	4	4	4	4	40
2	1	3	3	2	2	47
2	2	1	2	2	2	35
1	4	3	1	1	2	29
2	1	2	1	2	2	48
2	2	1	2	2	2	33
2	1	2	1	1	1	45
1	1	2	1	1	1	39
2	2	2	2	2	4	21
2	1	4	2	1	2	38
2	2	2	2	2	2	21
1	1	2	1	2	1	21
1	1	1	1	1	1	31
2	2	2	1	2	2	42
1	1	1	1	1	1	41
1	1	1	1	1	1	55
1	1	1	1	1	1	54
2	2	2	2	2	2	38
2	1	1	1	1	1	28
1	1	2	2	3	1	34
3	1	4	3	3	2	57
2	2	2	1	2	2	40
1	1	2	1	1	1	41
2	1	1	1	1	1	47
1	1	2	3	4	1	30
1	1	2	3	2	1	45
1	1	2	1	1	1	52
2	1	2	1	2	1	21
1	1	2	1	1	1	27
1	3	4	2	2	1	26
1	1	3	2	2	2	27
1	1	1	1	1	1	30

1	1	2	1	1	1	35
1	1	1	1	1	1	32
1	1	2	1	1	1	24
1	1	1	1	1	1	26
1	1	2	1	2	2	84
2	1	1	1	1	1	55
1	1	1	1	1	1	28
1	1	2	1	1	1	49
4	4	4	4	4	4	32
2	4	2	3	3	1	27
1	1	1	1	1	1	33
3	1	4	1	1	3	29
1	1	1	1	1	1	30
1	1	2	1	1	1	38
1	1	2	2	2	1	46
1	1	1	1	1	1	26
1	1	2	1	1	1	23
2	2	3	1	1	2	36
2	3	2	3	2	2	41
1	1	1	1	1	1	27
1	1	1	1	1	1	37
1	1	2	1	1	1	28
2	1	4	2	1	1	35
1	1	1	1	1	1	71
1	1	4	1	1	2	44
1	1	1	2	1	2	28
1	2	1	1	1	2	28
4	3	4	4	3	1	33
2	2	4	1	1	2	26
1	1	1	1	1	4	56
1	1	1	1	1	1	41
1	1	3	1	1	1	40
1	1	1	1	1	1	28
2	2	4	3	3	2	52
3	1	2	2	2	2	28
1	1	2	1	1	2	60
1	1	1	1	1	1	46
1	2	4	1	1	2	43
2	1	1	1	1	1	64
3	2	2	3	2	2	25
1	1	4	4	1	1	40
2	2	2	2	2	2	35
1	1	2	4	1	1	24
1	1	2	1	1	1	21
1	1	2	2	2	1	25
2	1	2	1	2	1	32
1	1	1	1	1	1	36
1	1	1	1	1	1	21
1	1	1	1	2	1	21
1	1	1	1	1	1	54
1	1	1	2	2	1	31
1	1	1	1	1	1	59
1	1	1	1	1	1	33
2	2	4	2	3	1	28

1	1	1	1	1	1	41
4	4	1	1	1	1	21
2	1	2	1	1	1	84
1	1	2	1	2	1	35
1	1	2	1	1	1	34
1	1	1	1	1	1	54
4	4	4	4	4	4	53
1	1	1	1	1	1	44
1	1	2	1	1	1	35
2	2	2	2	2	1	26
2	1	3	1	1	1	42
1	1	1	1	1	1	40
1	1	1	1	1	1	42
1	1	2	1	1	1	21
2	1	2	1	1	1	29
1	1	2	2	1	1	61
2	2	2	2	2	2	35
1	1	1	1	1	1	50
1	1	1	1	1	1	31
4	3	2	1	3	2	37
2	1	3	1	2	1	42
1	1	4	4	3	1	59
1	1	1	2	1	1	24
1	1	4	1	1	2	24
2	2	2	2	2	2	44
4	4	1	2	4	2	37
1	1	1	1	1	1	57
1	1	1	1	1	1	60
1	1	1	1	2	1	70
2	1	2	1	1	2	44
3	3	3	3	3	3	33
4	1	4	3	4	2	35
4	4	4	3	1	4	25
2	2	2	2	2	2	46
1	1	2	2	2	1	23
1	1	2	1	2	1	22
1	1	1	1	1	1	26
1	1	4	2	4	1	34
1	1	1	1	1	1	59
1	1	1	1	1	1	36
1	1	1	1	1	1	21
2	1	2	1	2	1	82
2	1	1	2	4	2	21
1	1	1	1	1	1	25
4	3	4	4	4	4	28
1	1	1	1	1	1	56
1	1	2	1	1	1	46
1	1	1	1	1	1	46
3	2	3	2	2	2	32
2	1	3	2	3	2	22
2	1	3	2	3	2	33
1	1	1	1	1	1	34
1	1	1	1	1	1	26
1	1	1	1	1	1	33

1	1	2	1	1	1	41
1	1	1	1	1	1	84
1	1	4	1	1	1	43
2	2	2	2	2	1	61
4	4	4	4	4	4	23
2	2	2	2	2	2	34
3	3	3	3	3	3	40
1	1	1	1	1	1	35
1	1	2	1	1	1	56
1	1	4	2	3	1	39
1	1	2	2	2	1	25
3	2	3	2	2	2	21
2	1	2	2	2	1	22
1	1	1	1	1	1	72
1	1	1	1	1	1	24
1	1	1	1	1	1	21
4	1	4	4	3	1	50
1	1	1	1	1	1	48
1	1	1	1	1	1	30
2	2	3	2	3	1	35
2	1	3	3	3	3	34
1	1	1	1	1	1	63
1	1	2	2	1	1	45
4	2	1	1	1	1	55
3	3	3	3	3	3	59
1	1	3	1	1	1	62
2	2	3	3	3	2	21
3	3	3	3	3	3	32
4	3	2	4	2	3	21
1	1	1	1	1	1	24
2	1	1	1	1	1	29
1	1	1	1	1	1	38
1	1	1	1	1	1	55
1	1	1	1	1	1	21
1	1	3	1	1	2	29
2	2	2	2	2	2	25
1	1	1	1	1	1	52
1	1	2	1	2	1	47
1	1	1	1	1	1	36
2	3	2	2	2	2	28
2	3	3	2	1	1	23
1	1	3	2	1	1	50
1	1	1	1	1	1	34
1	1	1	1	1	1	41
2	2	2	2	2	2	21
2	1	2	1	2	1	25
2	2	2	2	2	1	54
1	1	1	1	1	1	33
1	1	1	1	1	1	22
2	2	3	3	3	2	26
1	1	1	3	1	1	62
1	1	1	1	1	1	28
1	1	1	1	1	1	26
3	2	3	3	3	4	41

1	1	1	1	1	1	21
1	1	1	1	1	1	84
1	1	2	2	2	1	47
1	1	1	1	1	1	29
4	4	4	4	4	4	60
3	2	2	2	2	2	33
1	1	1	1	1	1	84
3	4	3	3	2	2	34
1	1	2	1	1	1	33
4	4	4	4	4	4	63
1	1	2	1	1	1	42
1	1	3	2	2	1	44
3	3	3	3	3	3	84
1	1	2	2	2	1	25
1	1	2	2	2	1	34
4	4	4	4	4	4	63
1	1	1	1	1	1	49
1	1	1	2	2	1	43
3	3	3	3	3	3	58
2	2	3	1	3	2	22
2	2	2	1	1	2	28
3	3	3	3	3	3	22
1	1	1	1	1	1	35
1	1	1	2	1	1	34
1	1	1	1	1	1	33
1	1	2	1	2	1	40
4	2	2	2	2	2	28
3	1	2	1	1	1	41
1	1	2	1	1	1	45
2	2	2	2	2	1	49
1	1	2	1	1	1	22
1	1	3	2	2	1	29
3	1	1	1	1	1	48
2	2	4	2	3	1	31
1	1	1	1	1	1	33
1	1	3	1	1	1	84
4	4	2	2	2	4	34
1	1	1	1	1	1	53
1	1	1	1	1	1	49
4	4	4	4	4	4	27
3	1	1	1	1	2	63
2	1	3	3	2	1	36
2	2	2	3	2	2	30
1	1	1	1	1	1	27
4	2	3	4	4	1	21
2	1	2	2	1	1	66
1	1	2	2	1	1	54
1	1	1	1	1	1	59
1	1	1	1	1	1	49
3	3	3	3	3	3	41
2	2	4	2	3	3	26
3	3	3	2	2	3	21
2	2	2	3	2	3	52
2	2	2	2	2	2	42

1	1	1	1	1	1	41
1	1	1	1	1	1	21
2	3	2	2	2	2	26
2	1	2	2	2	2	23
2	1	2	2	2	1	54
1	1	1	1	1	1	41
1	1	1	1	1	1	28
1	1	1	1	1	1	74
3	2	4	3	3	2	45
1	1	3	1	1	1	31
2	1	2	1	1	1	29
3	3	4	4	4	3	36
2	1	2	2	2	1	31
1	1	1	1	1	1	29
1	1	1	1	1	1	36
1	1	3	1	1	1	57
1	1	1	1	2	1	40
1	1	1	1	2	1	21
1	1	4	1	1	1	43
3	3	3	2	2	2	26
1	1	3	1	1	1	43
1	1	1	1	1	1	26
2	2	2	2	2	2	27
1	1	1	1	1	1	31
2	2	2	2	2	2	37
1	1	1	2	1	1	84
1	1	1	1	1	1	27
1	1	1	1	1	1	40
1	1	2	1	2	2	36
4	4	4	4	4	4	33
1	1	2	1	1	1	31
2	1	2	2	2	1	28
1	1	2	1	1	1	71
1	1	3	1	1	1	39
1	1	2	2	2	1	40
1	1	1	1	3	1	33
4	4	4	4	4	4	30
2	1	2	2	1	1	32
2	1	2	2	2	1	40
1	1	2	1	1	1	21
1	1	1	1	1	1	63
2	1	3	2	2	1	35
1	1	2	2	4	1	23
1	1	1	1	1	1	45
3	3	3	3	3	3	84
1	1	2	3	1	1	23
1	1	1	1	1	1	40
2	1	1	2	4	1	42
4	4	4	4	4	4	29
1	1	1	1	1	1	30
1	1	3	1	1	1	38
2	2	2	2	1	2	63
1	1	2	1	1	1	63
2	1	2	1	1	1	45

1	1	3	1	1	1	53
2	1	3	2	4	3	26
3	3	3	3	3	3	25
2	1	3	2	2	1	42
1	1	4	4	2	1	23
1	1	1	1	1	1	84
1	1	1	1	1	1	22
2	2	2	2	1	2	22
1	1	1	1	1	1	42
4	4	4	4	4	4	32
1	1	1	1	1	1	42
1	1	1	1	1	1	21
2	2	2	2	2	1	25
1	1	1	1	1	1	40
1	1	3	1	3	2	35
1	1	1	1	1	1	42
1	1	1	1	1	1	50
2	1	2	2	1	1	24
1	1	2	1	1	1	35
1	1	2	1	2	1	30
2	2	2	2	2	2	34
1	1	2	1	1	1	59
2	1	2	1	1	1	22
1	1	1	1	1	1	25
1	1	1	1	1	1	84
1	2	3	3	4	1	24
1	1	1	1	1	1	41
1	1	1	1	1	1	43
4	4	4	4	4	4	53
1	1	1	1	1	1	25
2	2	2	2	2	1	21
1	1	4	2	4	1	37
2	1	3	2	2	2	23
1	1	1	1	1	1	50
1	1	1	1	1	1	63
2	1	2	1	2	1	32
1	1	1	1	1	1	42
3	2	2	3	3	2	35
3	3	3	3	3	3	32
1	1	2	1	1	1	28
2	1	4	1	1	1	25
1	1	2	1	1	1	25
1	1	2	2	1	1	60
1	1	2	1	1	1	25
1	1	1	1	1	1	42
1	1	1	1	1	1	38
3	4	3	3	2	2	42
1	1	1	1	1	1	62
1	1	2	1	1	2	59
1	1	1	1	2	1	58
2	2	2	2	2	2	40
3	4	1	4	4	4	78
2	1	4	3	4	1	21
4	2	4	4	3	2	56

2	2	2	2	2	2	41
4	4	4	4	4	4	27
1	1	1	1	1	1	28
2	3	2	3	2	3	35
2	2	2	2	2	1	30
1	1	1	1	1	1	26
1	1	1	1	1	1	47
2	1	2	3	2	1	23
2	1	1	1	1	1	25
2	1	1	1	1	1	22
2	1	3	2	1	1	28
1	1	1	1	1	1	51
1	1	1	1	1	1	35
1	1	1	1	1	1	41
1	1	1	1	1	1	23
2	1	2	3	3	1	44
1	1	1	1	3	1	42
2	1	2	1	1	1	43
1	1	1	1	1	1	58
1	1	2	3	3	1	60
1	1	3	1	3	2	37
2	2	2	2	2	1	31
3	2	2	2	2	2	49
3	3	3	3	3	3	47
1	1	2	2	2	1	73
1	1	1	1	1	1	38
2	1	3	3	3	1	39
2	3	3	2	1	1	41
3	4	4	3	3	4	40
2	1	2	2	1	1	63
1	1	1	1	1	1	24
1	1	3	2	2	1	84
3	2	2	2	1	1	84
3	3	3	3	3	3	42
1	1	1	1	1	1	66
4	4	4	4	4	4	25
4	4	4	4	4	4	63
2	2	2	2	2	2	57
3	4	4	3	4	3	22
1	1	4	2	1	1	23
3	3	3	3	3	3	42
3	3	3	3	3	3	39
1	1	1	1	1	1	26
1	1	1	1	1	1	40
2	2	2	2	2	2	35
1	1	1	1	2	1	84
1	1	1	1	1	1	42
1	1	2	2	2	1	33
1	1	2	2	1	1	25
4	4	4	4	4	4	25
2	2	2	2	2	2	21
1	1	2	2	1	1	27
2	1	2	1	1	1	22
1	1	1	1	1	1	40

1	1	1	1	1	1	41
1	1	1	1	1	1	21
1	1	1	1	1	1	38
2	1	3	2	2	1	27
2	2	3	2	1	1	27
1	1	1	1	1	1	43
1	1	2	2	2	1	30
1	1	1	1	1	1	52
1	1	1	1	1	1	26
1	1	2	2	3	1	42
1	1	2	1	1	1	29
2	2	2	2	3	2	41
1	1	1	1	1	1	41
3	1	3	1	1	1	31
1	1	1	1	1	1	27
1	1	2	1	1	1	31
1	1	2	1	2	2	28
2	1	1	1	1	1	29
1	1	1	1	1	1	63
1	1	1	3	1	1	61
1	1	1	1	2	1	40
1	1	1	1	1	1	39
3	3	3	3	3	2	43
3	3	3	1	3	4	36
1	1	3	1	2	1	50
1	1	2	1	1	1	75
2	2	2	2	2	2	21
1	1	2	2	2	1	29
2	2	2	2	2	2	84
2	4	4	2	4	3	29
1	1	1	1	1	1	24
1	1	1	1	1	1	42
4	4	4	4	4	4	22
1	1	1	1	1	1	42
1	1	1	1	1	1	21
3	1	3	1	1	1	33
1	1	1	1	1	1	84
2	2	2	2	2	2	21
1	1	1	1	1	1	23
2	1	1	2	2	1	28
4	4	4	4	4	4	29
1	1	1	1	1	1	84
1	1	1	1	1	1	55
1	1	1	2	1	2	41
1	1	2	1	1	2	37
4	4	4	4	4	4	53
1	1	3	4	2	1	77
2	1	1	1	1	1	23
2	1	2	3	2	1	21
3	2	1	3	1	1	27
4	4	4	4	4	3	30
1	1	1	1	1	1	70
1	1	1	1	1	1	39
1	1	1	1	1	1	26

1	1	1	1	1	1	68
3	1	3	3	3	3	34
1	1	3	1	1	1	41
1	1	1	1	1	1	63
4	2	2	1	4	2	84
1	1	4	1	1	1	27
2	1	3	1	3	2	29
3	3	3	3	3	3	63
4	4	4	4	4	4	21
1	1	1	1	1	1	21
1	1	2	1	1	1	24
3	3	3	3	3	3	43
1	1	1	1	1	1	27
1	1	1	1	1	1	21
1	1	1	1	1	1	40
1	1	2	2	2	1	31
1	1	1	1	1	1	55
1	1	1	1	1	1	21
3	2	1	4	3	2	22
2	1	2	1	1	1	37
3	1	3	2	1	2	25
1	1	1	1	1	1	34
1	1	1	1	1	1	27
2	1	1	2	1	1	23
1	1	3	1	1	1	35
1	1	1	1	1	1	31
1	1	2	1	1	1	44
1	1	1	1	1	1	36
1	1	1	2	2	1	21
2	1	2	1	1	1	27
1	1	4	2	2	2	39
1	1	1	1	1	1	46
1	1	1	1	1	1	48
1	1	1	1	1	1	21
1	1	2	2	2	1	41
2	1	2	1	3	1	40
2	3	4	4	4	1	27
1	1	1	1	1	1	21
1	1	2	2	2	1	24
1	1	2	2	2	1	26
1	1	1	1	1	1	22
1	1	1	1	1	1	33
1	1	1	1	1	1	30
1	1	1	1	1	1	37
1	1	1	1	1	1	28
2	1	3	2	1	1	46
1	1	1	1	1	1	54
1	1	3	1	2	1	59
2	1	2	2	1	1	33
3	2	4	3	3	2	21
3	3	3	2	2	1	56
2	1	3	2	1	1	61
1	1	1	1	1	1	35
1	1	4	1	4	1	22

3	3	3	3	3	3	42
1	1	2	1	1	1	64
1	1	1	1	1	1	21
2	2	2	2	2	2	74
1	1	1	1	2	4	50
1	1	1	1	1	1	52
4	3	4	2	3	1	21
3	1	2	1	4	4	21
1	1	1	2	4	1	45
1	1	1	1	1	1	21
1	1	1	1	1	1	64
2	2	3	2	3	1	35
1	1	1	1	1	1	52
3	3	3	3	3	3	35
1	1	1	2	1	1	21
2	1	4	1	1	1	25
1	1	1	2	1	1	35
1	1	1	1	1	1	21
1	1	1	1	1	1	63
1	2	2	1	1	1	21
1	1	1	1	1	1	30
3	3	3	3	3	3	41
1	1	1	1	1	1	57
1	1	1	1	1	1	63
2	2	2	2	2	1	25
3	3	3	2	3	2	31
3	3	3	3	3	3	23
1	1	1	1	1	1	38
1	1	1	1	1	1	29
1	1	3	1	2	1	43
1	1	1	2	1	1	27
2	2	2	2	2	2	37
1	1	2	1	1	1	35
2	1	2	1	2	1	36
2	1	2	1	1	1	49
1	1	2	1	1	1	38
1	1	1	1	1	1	24
1	1	1	1	1	1	23
1	1	1	1	1	1	28
2	1	2	2	3	3	57
1	1	2	1	1	1	40
1	1	1	1	1	1	23
1	1	1	1	1	1	25
1	2	2	3	2	4	47
4	4	4	4	4	4	84
1	1	1	1	1	1	21
1	2	2	2	2	1	43
1	1	1	1	1	1	34
1	1	1	1	1	1	29
1	1	1	1	1	1	23
1	1	1	1	1	1	23
1	1	1	1	1	1	21
2	2	2	2	2	2	42
1	1	1	2	2	1	46

1	1	1	1	1	1	24
2	1	2	1	3	2	51
2	3	4	2	2	3	55
1	1	1	1	1	1	22
2	1	1	2	2	1	45
1	1	1	2	4	1	51
1	1	1	1	1	1	31
2	1	3	1	2	1	46
1	1	1	1	1	1	30
1	1	1	1	1	1	21
1	1	1	1	1	1	21
4	4	4	4	4	4	84
1	1	1	1	1	2	27
2	1	2	2	2	1	43
2	1	3	1	1	3	32
3	3	3	3	3	3	70
1	1	1	1	1	1	28
3	1	3	4	3	2	58
2	1	3	2	2	1	44
1	1	1	1	1	1	25
1	1	2	1	1	1	32
3	3	3	3	3	3	63
2	1	1	1	1	3	40
4	2	4	4	4	1	71
2	4	4	2	4	3	60
1	1	2	1	1	1	30
1	1	1	1	1	1	23
4	2	2	1	4	4	66
2	2	2	3	1	3	46
1	1	1	2	2	1	38
1	1	1	1	1	1	26
2	1	3	3	2	1	41
2	2	3	2	2	2	44
1	1	1	1	1	1	31
1	1	1	1	1	1	47
4	2	4	4	4	1	71
2	1	1	1	2	1	35
1	1	3	2	1	1	31
2	1	1	2	2	2	41
1	1	1	1	1	1	21
3	3	3	3	3	3	63
1	1	1	1	1	1	23
1	1	1	2	2	1	23
1	1	1	1	1	1	28
1	1	1	2	1	1	31
1	1	1	2	1	1	30
2	1	1	1	2	1	42
1	1	2	1	2	1	34
2	1	3	1	1	1	38
2	2	3	3	3	1	54
1	1	1	1	1	1	26
1	1	1	1	1	1	23
1	1	1	1	1	1	21
1	1	1	1	1	1	25

1	1	1	1	1	1	27
2	3	2	2	3	2	48
2	1	1	1	1	1	30
1	1	1	1	1	1	21
1	1	1	1	1	1	35
1	1	1	1	2	1	31
1	1	1	1	1	1	21
2	2	3	2	3	1	60
1	1	2	2	1	1	29
1	1	3	1	1	1	33
1	1	3	1	1	1	33
1	1	1	2	4	1	49
3	2	3	3	3	3	53
1	1	1	1	1	1	28
1	1	1	1	1	1	30
1	1	1	1	1	1	22
1	1	1	1	1	1	24
1	1	2	2	1	1	25
1	1	1	2	3	2	42
1	1	2	1	1	1	34
2	1	1	1	1	1	27
1	1	2	2	2	1	39
1	1	1	1	1	1	21
1	1	1	1	1	1	21
1	1	1	2	1	1	29
1	1	1	1	1	1	29
1	1	1	1	1	1	21
1	1	1	1	1	1	30
1	1	1	1	1	1	23
1	1	1	1	1	1	25
1	1	2	1	1	1	31
1	1	1	1	2	1	27
2	1	1	1	1	1	41
1	1	1	1	1	1	23
1	1	3	1	2	1	31
1	1	1	1	1	1	23
2	1	2	1	1	2	36
1	1	3	1	1	1	34
4	4	1	4	4	4	66
1	1	1	1	1	1	24
1	1	1	1	1	1	22
1	1	2	2	2	1	43
1	1	1	1	1	1	21
1	1	1	1	1	1	22
1	1	2	1	3	1	38
2	1	2	1	1	1	35
1	3	3	2	1	1	43
2	1	1	1	1	1	30
1	1	1	1	1	1	22
1	1	2	1	1	1	31
1	1	2	1	1	1	26
1	1	1	1	1	1	23
1	1	3	2	1	1	47
1	1	1	1	1	1	28

1	3	3	1	2	3	51
1	1	1	2	2	2	41
1	1	1	1	1	1	25
1	1	2	1	1	1	33
1	1	4	2	2	1	38
2	3	3	1	2	2	55
1	2	2	1	1	2	36
1	1	1	1	1	1	28
2	1	3	1	1	1	37
1	2	2	2	2	2	46
1	1	2	1	1	1	29
1	1	2	1	1	1	30
2	1	2	1	1	1	43
1	1	2	1	1	1	24
1	1	2	1	1	1	24
1	1	1	1	1	1	25
1	1	2	2	1	1	36
1	1	1	1	1	1	22
1	1	1	1	1	1	22
1	1	1	1	1	1	22
2	1	3	3	4	2	54
2	1	4	2	2	1	39
1	1	1	1	1	1	21
1	1	1	2	1	1	34
1	1	1	1	1	1	22
1	1	2	1	1	1	27
2	1	2	2	2	1	40
1	1	1	1	1	1	26
2	2	2	2	2	2	42
1	1	1	1	1	1	23
2	2	2	2	2	2	22
3	1	2	1	2	1	42
1	1	2	1	1	1	39
1	1	2	1	1	1	28
1	2	2	1	1	1	25
2	1	2	2	2	1	33
1	1	1	1	1	1	40
1	1	1	1	1	1	24
1	1	3	1	1	1	21
2	1	1	1	1	1	32
1	1	1	1	2	1	28
1	1	1	1	1	1	28
3	1	2	3	2	1	21
1	1	1	1	1	1	51
1	1	1	1	1	1	23
1	1	1	1	1	1	24
1	1	1	1	1	1	26
1	1	3	1	1	1	21
1	1	1	1	1	1	30
1	1	1	1	1	1	23
1	1	2	1	1	2	28
1	2	2	2	2	2	36
1	1	2	1	1	1	45
1	1	3	2	1	1	28

2	2	2	2	2	2	35
1	1	1	1	1	1	42
1	1	1	1	1	1	30
2	1	1	1	1	1	27
1	1	1	1	1	1	59
1	1	3	2	1	1	21

ore
